# Sex-specific personality metatrait profiles and their associations with APOE, PICALM variants, and cognitive performance in healthy middle-aged adults: a cluster-based analysis

**DOI:** 10.3389/fragi.2026.1933176

**Published:** 2026-10-29

**Authors:** Adam Bednorz, Dorota Religa

**Affiliations:** 1 John Paul II Geriatric Hospital, Katowice, Poland; 2 Institute of Psychology, Humanitas University, Sosnowiec, Poland; 3 Department of Neurobiology, Care Sciences and Society (NVS), Karolinska Institutet, Stockholm, Sweden; 4 Theme Inflammation and Aging, Karolinska University Hospital, Huddinge, Sweden; 5 Department of Clinical Sciences, Lund University, Malmö, Sweden

**Keywords:** Alzheimer’s disease, APOE genotype, machine learning, neuropsychological assessments, personality traits, PICALM genotype

## Abstract

**Introduction:**

APOE *ε*4 is the strongest common genetic risk factor for late-onset Alzheimer’s disease, while PICALM rs3851179 has been associated with amyloid-*β* clearance. Personality can be summarized into two higher-order metatraits: Stability, reflecting emotional and behavioral regulation, and Plasticity, reflecting cognitive flexibility. This study examined whether genetic associations with episodic memory and fluid intelligence differ across Stability–Plasticity personality clusters in cognitively healthy middle-aged adults.

**Material and Methods:**

The sample comprised 192 participants. NEO-FFI personality traits were standardized to derive the metatraits Stability and Plasticity. Sex-stratified agglomerative hierarchical clustering (Ward’s linkage) identified two personality profiles characterized by relatively higher or lower levels of both metatraits. Cognitive outcomes included the California Verbal Learning Test (CVLT) and Raven’s Progressive Matrices (RPM). Sex-stratified regression models examined associations between APOE *ε*4 carrier status, PICALM rs3851179 GG genotype, age, education, and cognitive performance.

**Results:**

After false discovery rate correction, significant associations were observed only in females. In the low Stability–low Plasticity cluster, the APOE × age interaction was negatively associated with CVLT Recognition performance (p_FDR_ = 0.019; R^2^ = 0.254). In the high Stability–high Plasticity cluster, models incorporating APOE and/or PICALM status were significantly associated with CVLT Recognition performance (R^2^ = 0.583–0.619, pFDR = 0.010–0.019). For fluid intelligence, significant negative APOE × age and APOE × age × education interactions, as well as a negative APOE × PICALM × age interaction, were observed in the respective models (R^2^ = 0.553–0.696, p_FDR_ = 0.001–0.019).

**Conclusion:**

Personality metatraits were associated with variability in APOE-related cognitive outcomes, particularly among females. These findings suggest that personality structure can contribute to individual differences in cognitive aging pathways associated with genetic risk. However, the results should be interpreted considering the cross-sectional design and limited sample size.

## 1 Introduction

The age-standardized incidence of dementia and Alzheimer’s disease (AD) has remained relatively stable throughout the world; however, the total number of cases has increased substantially, particularly among women and in regions undergoing demographic aging (Qin et al., 2025). AD shows marked sex differences, and women account for approximately two-thirds of diagnosed cases worldwide (Subramaniapillai et al., 2021; Aggarwal and Mielke, 2023; Emrani and Sundermann, 2025; Arenaza-Urquijo et al., 2024). Neuropathological studies indicate that, despite comparable levels of core AD pathology, women exhibit a stronger association between brain changes and clinical symptoms, more extensive tau pathology, and faster cognitive decline (Bonkhoff et al., 2025; Cicero et al., 2026).

The APOE gene, which encodes apolipoprotein E, represents the strongest common genetic risk factor for late-onset AD. It occurs in three major allelic forms: *ε*2, *ε*3, and *ε*4 (Huang et al., 2019; Raulin et al., 2022). The APOE *ε*4 allele substantially increases the risk of AD, with heterozygous carriers showing approximately three-fold higher risk and homozygous carriers up to a 15-fold higher risk compared with the *ε*3/*ε*3 genotype (Huang and Mahley, 2014; Hoogmartens et al., 2021; Liu et al., 2013). Importantly, APOE *ε*4-related effects may be sex dependent, with evidence suggesting greater vulnerability to AD-related pathology and cognitive decline among women (Altmann et al., 2014; Neu et al., 2017; Groot et al., 2025). Sex differences in tau accumulation appear early in the AD continuum, with women—particularly APOE *ε*4 carriers—showing greater tau deposition even in cognitively normal stages, which can accelerate the transition from normal aging to mild cognitive impairment (MCI) and dementia (Arenaza-Urquijo et al., 2024; Buckley et al., 2019). A meta-analysis including more than 40,000 cognitively healthy adults demonstrated that APOE *ε*4 carriers show lower performance in episodic memory, executive functions, and global cognitive ability, with effects becoming more pronounced with increasing age (Wisdom et al., 2011). Episodic memory appears to be one of the cognitive domains most consistently associated with APOE *ε*4 status, making it an important marker of early cognitive vulnerability in genetically at-risk individuals (Fan et al., 2019; Raulin et al., 2022).

PICALM (phosphatidylinositol binding clathrin assembly protein) is another genetic susceptibility locus for AD. The protein is involved in clathrin-mediated endocytosis and intracellular trafficking, processes relevant to amyloid precursor protein (APP) processing and amyloid-*β* (A*β*) transport and clearance (Ando et al., 2022; Xu et al., 2015). PICALM rs3851179 has been associated with variability in cognitive outcomes. The protective A allele has been associated with better preserved cognitive functioning, while the G allele has been associated with poorer cognitive performance in some populations (Mengel-From et al., 2011; Wu et al., 2022; Sweet et al., 2012; Ponomareva et al., 2020). Previous studies have reported associations between PICALM variation and measures of episodic memory, processing speed, and global cognitive performance, suggesting that this polymorphism may contribute to differences in cognitive aging trajectories (Zeng et al., 2019; Sun et al., 2017). In addition, interactions between APOE and PICALM variants have been associated with lower episodic memory performance, reduced processing speed, and altered cognitive efficiency, particularly among individuals carrying the APOE *ε*4 allele (Barral et al., 2012; Chang et al., 2019; Morgen et al., 2014).

Previous studies have shown associations between personality characteristics and risk of dementia, with higher Neuroticism and lower Conscientiousness, Openness, and Extraversion being associated with less favorable cognitive trajectories (D’Iorio et al., 2018; Terracciano and Sutin, 2019; Aschwanden et al., 2021). Personality has also been proposed to contribute to cognitive reserve by influencing behavioral regulation, lifestyle factors, and engagement in cognitively stimulating activities (Botwinick, 2013; Bogg and Roberts, 2013). Moreover, personality characteristics may interact with genetic susceptibility, including APOE *ε*4-related vulnerability, affecting individual differences in cognitive outcomes (Dar-Nimrod et al., 2012). Among various personality models, the Five-Factor Model (FFM) was identified as particularly useful for diagnosing personality traits (Henriques-Calado, 2023). Within the hierarchical Big Five framework, personality traits can be organized into two higher-order metatraits: Stability, reflecting emotional regulation and behavioral control, and Plasticity, reflecting exploration, cognitive flexibility, and adaptation to novelty (Digman, 1997; DeYoung, 2006; 2015).

The primary objective of the study was to determine whether personality metatraits (Plasticity and Stability) can identify meaningful sources of variability in cognitive functioning among cognitively healthy middle-aged adults at different levels of genetic risk for AD. In particular, we investigated the extent to which genetic variables (APOE, PICALM, and their interaction), sociodemographic factors (age and education), and psychological variables (personality metatraits) explain the variance in neuropsychological measures of episodic memory and fluid intelligence. To our knowledge, metatraits have not yet been directly investigated in the context of risk of AD and cognitive functioning (Troisi et al., 2024). Previous analyses conducted in the present dataset (Dzianok and Kublik, 2024) examined individual Big Five personality dimensions in relation to genetic risk groups. Among these traits, only Neuroticism differed significantly, with higher levels observed of carriers of APOE *ε*4 (Dzianok, 2023). Understanding APOE–PICALM interactions can improve the early identification of individuals at increased risk of accelerated cognitive decline in late-onset AD (Bellenguez et al., 2022; Barral et al., 2012). Other person-centered studies have focused on the relationship between cognitive performance and affective symptoms, highlighting the prognostic value of their co-occurrence for subsequent cognitive decline and the risk of dementia (Zhang et al., 2025).

Given the growing evidence for sex-related differences in AD risk and progression, all analyses were conducted separately for women and men. To identify latent patterns in personality traits, the data was reanalyzed using unsupervised machine learning methods, which detect underlying structures in unlabeled data without relying on predefined categories (Dalmaijer et al., 2022). Data-driven techniques such as clustering and reduction in dimensionality are increasingly used in neuropsychology and behavioral genetics to uncover complex relationships between cognitive phenotypes and genetic variation (Wang et al., 2024; Scheltens et al., 2016).

### 1.1 Study objectives

The present study aimed to investigate whether personality metatraits (Stability and Plasticity) are associated with individual variability in cognitive functioning and whether personality-defined profiles may reveal heterogeneous associations between genetic and sociodemographic factors and cognition in cognitively healthy middle-aged adults. Specifically, our goal was to:

- Identify personality-based cognitive profiles using a data-driven clustering approach;
- Examine whether associations between APOE *ε*4 status, PICALM rs3851179 GG genotype, age, education, and cognitive performance differ across personality-defined clusters.

By integrating personality metatraits, genetic factors, demographic variables, and neuropsychological measures, this study aimed to provide a more comprehensive characterization of factors that contribute to cognitive variability during the preclinical stages of the risk of AD.

## 2 Materials and methods

### 2.1 Dataset and participants

Participants were recruited from the PEARL-Neuro Database, described in detail elsewhere (Dzianok and Kublik, 2024). The dataset includes genetic, demographic, health, and psychometric information from 192 cognitively healthy middle-aged adults (50–63 years). Genotyping of APOE (rs429358/rs7412) and PICALM (rs3851179) was performed using Sanger sequencing (Dzianok and Kublik, 2024). The rs3851179 polymorphism was analyzed using a recessive genetic model by comparing GG homozygotes with carriers of at least one A allele (AA/AG). The majority of the participants had higher education (77.6%). For the present analyses, only variables related to cognitive functioning were included, comprising measures derived from the California Verbal Learning Test (CVLT), Raven’s Progressive Matrices (RPM). The NEO-FFI Personality Inventory was used to assess personality traits. The definitions of all selected variables are provided in Table 1.

**TABLE 1:** Selected cognitive function variables from the dataset.

Variable	Description
CVLT_1	CVLT: List A, trials 1–5 (total learning)
CVLT_2	CVLT: List A, trial 1 (initial recall)
CVLT_3	CVLT: List A, trial 5 (final recall)
CVLT_4	CVLT: List B (interference list)
CVLT_5	CVLT: Short-term delay free recall
CVLT_6	CVLT: Short-term delay cued recall
CVLT_7	CVLT: Long-term delay free recall
CVLT_8	CVLT: Long-term delay cued recall
CVLT_9	CVLT: Perseverations
CVLT_10	CVLT: Intrusion errors – free recall
CVLT_11	CVLT: Intrusion errors – cued recall
CVLT_12	CVLT: Recognition – total hit
CVLT_13	CVLT: Recognition – false alarms
RPM	Raven’s progressive matrices – total score

*Abbreviations:* CVLT, California Verbal Learning Test; RPM, Raven’s Progressive Matrices.

The methods used in this study are detailed below:

- CVLT - is a widely used tool in clinical and research settings to assess verbal learning and memory. It enables the evaluation of various cognitive processes, including learning between repetitions, serial position effects, semantic clustering, intrusions, and proactive interference. The test was designed with ecological validity, incorporating tasks that reflected daily activities, such as recalling shopping lists (Delis et al., 2008; Elwood, 1995; Łojek et al., 2010).
- RPM - is a nonverbal test designed to assess general cognitive ability, particularly abstract reasoning and problem-solving skills. The research findings indicate that RPM can be considered a relatively independent test of cultural factors to measure fluid intelligence (Raven and Raven, 2003; Raven, 2008; Jaworowska and Szustrowa, 2010). The allotted time to complete the test was set at 30 min, replacing the unlimited time frame used in the original version (Dzianok and Kublik, 2024).
- NEO-FFI Personality Inventory - to assess the Big Five personality traits: neuroticism, extraversion, openness to experience, agreeableness, and conscientiousness. The instrument consists of 60 items, and the database contains summary scores for each trait (Costa and McCrae, 1989; Costa Jr and McCrae, 2000; Zawadzki et al., 2010).

### 2.2 Machine learning approach and statistical analysis

Step 1. Prior to analyses, the dataset was preprocessed by excluding participants with missing values for personality traits or sex. The Big Five variables (NEO_NEU, NEO_EXT, NEO_OPE, NEO_AGR, NEO_CON) were standardized using z-scores. For clustering analyses and descriptive statistics of the clustering sample, only participants with complete NEO-FFI data were included.
Step 2. Two higher-order personality metatraits were computed from standardized Big Five dimensions (DeYoung et al., 2015, 2006; DeYoung et al., 2002). Stability was defined as:

$$Stability=\frac{-Neuroticism+Agreeableness+Conscientiousness}{3}$$

whereas Plasticity was calculated as:

$$\text{Plasticity}=\frac{\text{Extraversion}+\text{Openness}}{2}$$

Step 3. Agglomerative hierarchical clustering was performed directly on the standardized Stability and Plasticity metatrait scores using Ward’s linkage and Euclidean distance. The number of clusters was fixed at two to identify distinct personality configurations characterized by relatively lower or higher levels of both metatraits. All clustering procedures were performed separately for females and males. Because clustering was conducted independently within each sex, cluster labels represent relative metatrait configurations within each subsample and should not be interpreted as directly equivalent categories across sexes. Clustering was performed using the AgglomerativeClustering algorithm implemented in *scikit-learn* (Li et al., 2022). Euclidean distance was used as the similarity metric:

$$d\left(x,\;y\right)=\sqrt{\sum_{i=1}^{n}{\left(x_{i}-y_{i}\right)}^{2}}$$

where:

$$d\left(x,\;y\right)$$

denotes the Euclidean distance between observations x and y, where *x*_*i*_ and *y*_*i*_ are the values of the *i*th variable, and *n* is the total number of variables.

Cluster assignments were subsequently merged back into the dataset and visualized using scatterplots of Stability and Plasticity scores. For each sex-stratified sample, agglomerative hierarchical clustering was performed a range of *k* = 2 to *k* = 9, and silhouette coefficients were calculated to assess cluster cohesion and separation. In the female subsample, the highest silhouette value was observed for *k* = 2 (silhouette = 0.4025), which was selected as the optimal solution. In the male subsample, the silhouette analysis yielded comparable values for *k* = 2 (silhouette = 0.3805) and *k* = 9 (silhouette = 0.3821). Despite the slightly higher silhouette value at *k* = 9, this solution was not selected due to very small resulting cluster sizes and limited interpretability (male sample: *n* = 88, with the smallest cluster containing *n* = 20, approaching lower bounds for reliable unsupervised learning inference). Therefore, the two-cluster solution was retained for consistency across sexes and to maintain sufficient cluster stability. All detailed silhouette profiles across tested values of *k* and both subsamples are provided in the Supplementary Materials (Supplementary Table S1; Supplementary Figures S1, S2). The hierarchical dendrogram was cut at a distance level of approximately 8.291 for males and 7.732 for females (see Supplementary Figure S3 in Supplementary Materials).

To evaluate the adequacy of cluster sizes, an *a priori* power analysis was conducted. Assuming *α* = 0.05, power = 0.80, and a medium-to-large effect size (Cohen’s *d* = 0.6), the minimum required sample size per group was estimated at approximately 45 participants (Brydges, 2019; Lakens, 2022). In the present study, the resulting clusters comprised 64 and 32 women, and 68 and 20 men, which is consistent with methodological recommendations for unsupervised learning approaches, suggesting a minimum of 20–30 participants per subgroup (Dalmaijer et al., 2022).

To evaluate the robustness of the identified cluster solutions, additional stability analyses were performed. Bootstrap resampling was conducted separately for females and males using 1,000 iterations. For each bootstrap sample, agglomerative hierarchical clustering with Ward’s linkage was repeated using standardized Stability and Plasticity metatrait scores. Similarity between the original cluster assignment and bootstrap-derived solutions was quantified using the Adjusted Rand Index (ARI). In addition, individual cluster assignment stability was calculated as the proportion of bootstrap iterations in which each participant was assigned to the same cluster as in the original solution.

To assess whether the identified clustering structure depended on the selected clustering algorithm, additional analyses were performed using alternative hierarchical linkage strategies (average and complete linkage) and non-hierarchical clustering approaches, including k-means clustering and Gaussian mixture modeling. The agreement between alternative solutions and the original Ward-based clustering was evaluated using ARI values.

The bootstrap analyses indicated moderate stability of the two-cluster solution, with higher individual assignment stability observed in males compared with females. Alternative clustering approaches showed moderate agreement with the original Ward-based solution, supporting the interpretation of clusters as broad personality metatrait profiles rather than strictly discrete categories. Detailed stability analyses are reported in Supplementary Materials (Supplementary Table S2).

To assess whether the derived personality metatrait clusters differed in cognitive performance, independent-samples Welch’s *t*-tests were conducted for each CVLT outcome and RPM score. The effect sizes were quantified using Cohen’s *d*. To account for multiple tests across neuropsychological outcomes, *p*-values were adjusted using the Benjamini–Hochberg False Discovery Rate (FDR) procedure (Benjamini and Hochberg, 1995). Differences in the distribution of genetic variants between clusters were evaluated using Pearson’s *χ* ^2^ tests. Specifically, separate analyses were performed for the full distribution of APOE haplotypes, APOE *ε*4 carrier status, PICALM rs3851179 genotypes, and PICALM GG carrier status. Statistical significance was set at *p <* 0.05 (two-tailed).

Step 4. Prior to regression analyses, participants with missing values for neuropsychological outcomes, demographic variables (age or education), genotype data, or metatrait cluster assignment were excluded using complete-case analysis.

Neuropsychological outcome measures CVLT indices and the total score of RPM. APOE *ε*4 carrier status (presence of at least one *ε*4 allele; e.g., *ε*3/*ε*4 or *ε*4/*ε*4) was coded as 1 and non-carriers as 0. Homozygous carriers of the PICALM rs3851179 GG genotype were coded as 1, while all remaining genotypes were coded as 0. The APOE *ε*2*ε*4 genotype was retained in the dataset but was not classified as an *ε*4 carrier, as the protective *ε*2 allele reduces risk of AD by approximately 40% and partially mitigates the risk associated with *ε*4 (Corder et al., 1994; APOE and Alzheimer Disease Meta Analysis Consortium, 1997; Karagas et al., 2025).

Step 5. Linear regression analyses were performed separately within each metatrait cluster and sex subgroup identified in the previous clustering step. For each neuropsychological outcome variable, a hierarchical set of regression models was evaluated to examine the effects of APOE *ε*4 carrier status, PICALM rs3851179 GG carrier status, age, and education on cognitive performance.

All regression models followed the principle of hierarchical inclusion of lower-order terms. Therefore, higher-order interaction terms were never tested independently, but were evaluated together with the corresponding main effects and lower-order interaction terms required for their interpretation.

The APOE-related models were specified as follows:

- Model 1a:

$$Y=\beta_{0}+\beta_{1}APOE+\epsilon$$

- Model 1b:

$$Y=\beta_{0}+\beta_{1}\text{APOE}+\beta_{2}{\text{Age}}_{c}+\beta_{3}\left(\text{APOE }\times {\text{Age}}_{c}\right)+\epsilon$$

- Model 1c:

$$Y=\beta_{0}+\beta_{1}\text{APOE}+\beta_{2}{\text{Age}}_{c}+\beta_{3}{\text{Education}}_{c}+\beta_{4}\left(\text{APOE}\times {\text{Age}}_{c}\right)+\beta_{5}\left(\text{APOE}\times {\text{Education}}_{c}\right)+\beta_{6}\left({\text{APOE}\times \text{Age}}_{c}\times {\text{Education}}_{c}\right)+\epsilon$$

The PICALM-related models were specified analogously:

- Model 2a:

$$Y=\beta_{0}+\beta_{1}PICALM+\epsilon$$

- Model 2b:

$$Y=\beta_{0}+\beta_{1}PICALM+\beta_{2}Age_{c}+\beta_{3}\left(PICALM\;\times Age_{c}\right)+\epsilon$$

- Model 2c:

$$Y=\beta_{0}+\beta_{1}\text{PICALM}+\beta_{2}{\text{Age}}_{c}+\beta_{3}{\text{Education}}_{c}+\beta_{4}\left(\text{PICALM}\times {\text{Age}}_{c}\right)+\beta_{5}\left(\text{PICALM}\times {\text{Education}}_{c}\right)+\beta_{6}\left({\text{APOE}\times \text{Age}}_{c}\times {\text{Education}}_{c}\right)+\epsilon$$

Combined genetic models were specified as:

- Model 3a:

$$Y=\beta_{0}+\beta_{1}APOE+\beta_{2}PICALM+\beta_{3}\left(APOE\;\times PICALM\;\right)+\epsilon$$

- Model 3b:

$$Y=\beta_{0}+\beta_{1}\text{APOE}+\beta_{2}\text{PICALM}+\beta_{3}{\text{Age}}_{c}+\beta_{4}\left(\text{APOE}\times \text{PICALM}\right)+\beta_{5}\left(\text{APOE}\times {\text{Age}}_{c}\right)+\beta_{6}\left(\text{PICALM}\times {\text{Age}}_{c}\right)+\epsilon$$

- Model 3c:

$$Y=\beta_{0}+\beta_{1}\text{APOE}+\beta_{2}\text{PICALM}+\beta_{3}{\text{Age}}_{c}+\beta_{4}{\text{Education}}_{c}+\beta_{5}\left(\text{APOE}\times \text{PICALM }\right)+\beta_{6}\left({\text{Age}}_{c}\times {\text{Education}}_{c}\right)+\epsilon$$

where *Y* represents each neuropsychological outcome measure, and *β*_0_ represents the intercept. The coefficients *β*_1_ *, β*_2_,…. represent the regression coefficients associated with the corresponding main effects and interaction terms. The binary *APOE* variable represents APOE *ε*4 carrier status, coded as 1 for *ε*3*ε*4 and *ε*4*ε*4 carriers and 0 for non-carriers. The binary *PICALM* variable represents PICALM rs3851179 genotype, coded as 1 for GG carriers and 0 for other genotypes. Continuous predictors were mean-centered prior to the calculation of interaction terms:

$${Age}_{c}=Age-\bar{Age}$$

$${Education}_{c}=Education-\bar{Education}$$

where *Age*_*c*_ and *Education*_*c*_ denote the mean-centered age and education variables, respectively, calculated by subtracting the corresponding sample mean ( $\bar{Age}$ or $\bar{Education}$ ) from each participant’s value. Mean-centering was applied to facilitate the interpretation of main effects and to reduce non-essential multicollinearity between continuous predictors and their interaction terms.

Interaction terms represent the combined effects of the corresponding predictors. For example, *APOE × Age*_*c*_ represents the interaction between APOE carrier status and age, whereas *APOE × Age*_*c*_ *× Education*_*c*_ represents the three-way interaction between *APOE* carrier status, age, and education. In the combined genetic models, *APOE × PICALM* represents the interaction between the two genetic variables, while higher-order interaction terms represent their joint associations with age and/or education. Finally, *ϵ* denotes the residual error term, representing the portion of variance in the outcome not explained by the predictors included in the respective model.

Linear regression models were estimated using Ordinary Least Squares (OLS). For each model, the coefficient of determination (*R* ^2^) and overall model significance were evaluated using the F-test. Because the primary objective was to determine whether combinations of genetic and demographic variables explained variability in cognitive performance, statistical inference initially focused on overall model significance. For models surviving false discovery rate (FDR) correction, individual regression coefficients (*β*), standard errors, and 95% confidence intervals were subsequently examined to facilitate interpretation. As continuous predictors were mean-centered but not standardized prior to analysis, all reported regression coefficients represent unstandardized estimates expressed in the original measurement units.

Due to missing neuropsychological, demographic, or genetic data, regression analyses were performed using complete-case samples separately within each sex-by-cluster subgroup. Consequently, the effective sample size varied across models and is reported for every analysis.

Step 6. To account for multiple testing, the Benjamini–Hochberg False Discovery Rate (FDR) procedure (Benjamini and Hochberg, 1995) was applied separately within females and males across all tested regression models, including all neuropsychological outcomes, metatrait clusters, and genetic model families (APOE, PICALM, and combined *APOE × PICALM* models). Models with adjusted *p*-values (_p_FDR < 0.05) were considered statistically significant.

For FDR-significant models, additional analyses were performed to evaluate model assumptions, estimate stability, and the potential risk of overfitting. Model diagnostics included residual analyses, variance inflation factors (VIF), Cook’s distance, Shapiro–Wilk tests of residual normality, Breusch–Pagan tests for heteroscedasticity, Durbin–Watson statistics for residual autocorrelation, and condition numbers (Supplementary Materials, Supplementary Table S3). Estimate robustness was further assessed using heteroscedasticity-consistent HC3 standard errors together with HC3-based and bootstrap-derived 95% confidence intervals obtained from 1,000 bootstrap resamples (Supplementary Materials, Supplementary Table S4). Predictor-level and model-level VIF summaries are reported in Supplementary Materials (Supplementary Tables S5A,B), whereas model complexity was evaluated using the ratio of observations to estimated parameters for each regression model and subgroup (Supplementary Table S5C).

As an additional sensitivity analysis, FDR-significant associations involving CVLT Recognition Total Hits (CVLT_12) were re-estimated using robust linear regression with the Huber M-estimator because this outcome demonstrated a restricted score distribution and potential ceiling effects. These analyses were performed to determine whether the observed associations remained stable after reducing the influence of potentially influential observations (Supplementary Materials, Supplementary Table S6; Supplementary Figure S4).

Given the relatively small sample sizes within several sex-by-cluster subgroups, particularly for models containing higher-order gene *×* demographic interactions, these analyses should be regarded as exploratory. To improve the transparency and reliability of inference, the supplementary diagnostic and robustness analyses described above were performed, and all higher-order interaction models were interpreted with appropriate caution.

Step 6a. To examine whether personality metatraits showed independent linear associations with cognitive performance, complementary regression analyses were conducted using Stability and Plasticity as continuous predictors. These models were designed to test whether individual metatrait dimensions were associated with neuropsychological outcomes after accounting for demographic and genetic variables.

All analyses were conducted in Python using *pandas*, *NumPy*, *SciPy*, *scikit-learn*, and *statsmodels*. Figure 1 provides an overview of the analytical workflow used in this study.

**FIGURE 1:**
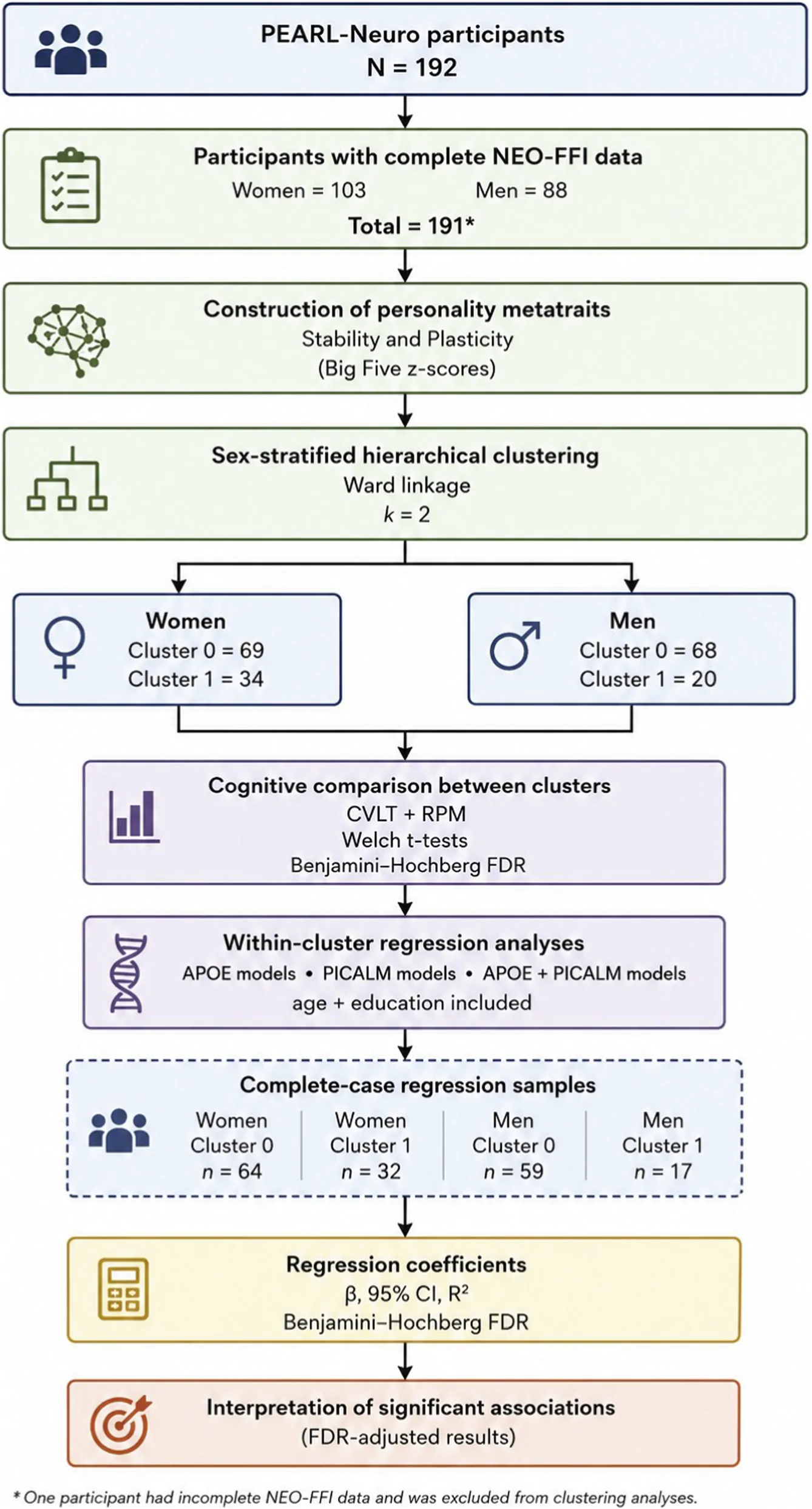
Overview of the study workflow.

## 3 Results

### 3.1 Cluster characteristics for female and male participants

The descriptive statistics for the total sample and sex-stratified groups, calculated from the raw data before data preprocessing and subsequent analytical procedures, are presented in Table 2 ^˜^.

**TABLE 2:** Descriptive characteristics of participants included in the clustering analyses (participants with complete NEO-FFI data).

Variable	Women (N = 103)	Men (N = 88)
Age	54.93 (3.19)	55.17 (2.98)
NEO neuroticism	19.80 (9.51)	16.26 (7.67)
NEO extraversion	25.85 (7.62)	26.51 (6.28)
NEO openness	31.27 (5.66)	31.08 (6.93)
NEO agreeableness	33.18 (5.23)	31.26 (5.99)
NEO conscientiousness	31.43 (7.11)	30.00 (6.95)
CVLT_1	64.85 (8.02)	58.32 (9.38)
CVLT_2	10.08 (1.96)	8.84 (1.91)
CVLT_3	14.50 (1.56)	13.19 (2.37)
CVLT_4	8.71 (2.01)	7.77 (1.92)
CVLT_5	13.71 (2.13)	11.64 (2.89)
CVLT_6	13.98 (1.52)	12.81 (2.26)
CVLT_7	14.25 (1.85)	12.44 (2.88)
CVLT_8	14.18 (1.58)	13.00 (2.33)
CVLT_9	4.65 (5.12)	3.45 (3.59)
CVLT_10	0.97 (1.65)	1.31 (1.62)
CVLT_11	0.43 (0.81)	1.14 (2.02)
CVLT_12	15.55 (0.75)	15.17 (1.32)
CVLT_13	0.38 (0.97)	0.68 (1.25)
RPM	52.29 (5.00)	53.34 (3.90)
APOE ε4 carriers (e3/e4 + e4/e4)	24.3%	26.1%
PICALM rs3851179 GG carriers	42.7%	39.8%

*Abbreviations:* CVLT, California Verbal Learning Test; RPM, Raven’s Progressive Matrices; NEO-FFI–NEO, Five-Factor Inventory; NEU, Neuroticism; EXT, Extraversion; OPE, Openness; AGR, Agreeableness; CON, Conscientiousness.

Continuous variables are presented as mean (SD), whereas genetic variables are presented as percentages of carriers.

Hierarchical clustering based on the Stability and Plasticity metatraits identified two distinct clusters within the female and male subsample. Cluster sizes and descriptive statistics are presented in Table 3 Cluster 1 was characterized by higher levels of both Stability and Plasticity, while Cluster 0 showed lower scores on both metatraits (Table 3). Cluster-specific metatrait profiles are presented in Figure 2.

**TABLE 3:** Descriptive characteristics of the newly identified personality clusters in women and men.

Sex	Variable	Cluster 0 (N)	Mean ± SD	Cluster 1 (N)	Mean ± SD
Women	NEO_NEU	69	23.80 ± 8.62	34	11.68 ± 4.99
	NEO_EXT	69	22.57 ± 6.54	34	32.53 ± 4.80
	NEO_OPE	69	30.25 ± 5.70	34	33.35 ± 5.02
	NEO_AGR	69	31.61 ± 4.90	34	36.38 ± 4.38
	NEO_CON	69	28.38 ± 6.11	34	37.62 ± 4.54
	Stability	69	−0.39 ± 0.49	34	0.78 ± 0.30
	Plasticity	69	−0.31 ± 0.72	34	0.62 ± 0.47
	CVLT_1	69	64.33 ± 8.89	34	65.91 ± 5.87
	CVLT_2	69	10.00 ± 2.10	34	10.24 ± 1.65
	CVLT_3	69	14.39 ± 1.74	34	14.71 ± 1.12
	CVLT_4	69	8.62 ± 2.10	34	8.88 ± 1.82
	CVLT_5	69	13.59 ± 2.31	34	13.94 ± 1.70
	CVLT_6	69	14.00 ± 1.64	34	13.94 ± 1.28
	CVLT_7	69	14.09 ± 2.05	34	14.59 ± 1.31
	CVLT_8	69	14.09 ± 1.74	34	14.38 ± 1.21
	CVLT_9	69	5.17 ± 5.32	34	3.59 ± 4.57
	CVLT_10	69	0.90 ± 1.70	34	1.12 ± 1.57
	CVLT_11	69	0.36 ± 0.79	34	0.56 ± 0.86
	CVLT_12	68	15.47 ± 0.82	34	15.71 ± 0.58
	CVLT_13	68	0.40 ± 0.92	34	0.35 ± 1.07
	RPM	69	52.23 ± 5.11	34	52.41 ± 4.84
Men	NEO_NEU	68	18.41 ± 7.03	20	8.95 ± 4.73
	NEO_EXT	68	24.85 ± 5.90	20	32.15 ± 3.86
	NEO_OPE	68	29.57 ± 6.81	20	36.20 ± 4.54
	NEO_AGR	68	29.99 ± 5.75	20	35.60 ± 4.68
	NEO_CON	68	28.16 ± 6.34	20	36.25 ± 5.14
	Stability	68	−0.25 ± 0.60	20	0.86 ± 0.40
	Plasticity	68	−0.24 ± 0.64	20	0.82 ± 0.48
	CVLT_1	68	58.50 ± 8.79	20	57.70 ± 11.40
	CVLT_2	68	8.79 ± 1.82	20	9.00 ± 2.22
	CVLT_3	68	13.32 ± 2.27	20	12.75 ± 2.71
	CVLT_4	68	7.84 ± 2.00	20	7.55 ± 1.64
	CVLT_5	68	11.65 ± 2.95	20	11.60 ± 2.72
	CVLT_6	68	12.75 ± 2.24	20	13.00 ± 2.38
	CVLT_7	68	12.35 ± 2.93	20	12.75 ± 2.77
	CVLT_8	68	12.94 ± 2.40	20	13.20 ± 2.12
	CVLT_9	68	3.50 ± 3.79	20	3.30 ± 2.92
	CVLT_10	68	1.47 ± 1.68	20	0.75 ± 1.29
​	CVLT_11	68	1.18 ± 2.14	20	1.00 ± 1.59
	CVLT_12	68	15.19 ± 1.35	20	15.10 ± 1.21
	CVLT_13	68	0.72 ± 1.36	20	0.55 ± 0.76
	RPM	68	53.00 ± 4.11	20	54.50 ± 2.89

*Abbreviations:* CVLT, California Verbal Learning Test; RPM, Raven’s Progressive Matrices; NEO-FFI–NEO, Five-Factor Inventory; NEU, Neuroticism; EXT, Extraversion; OPE, Openness; AGR, Agreeableness; CON, Conscientiousness.

Values are presented as mean ± SD.

**FIGURE 2:**
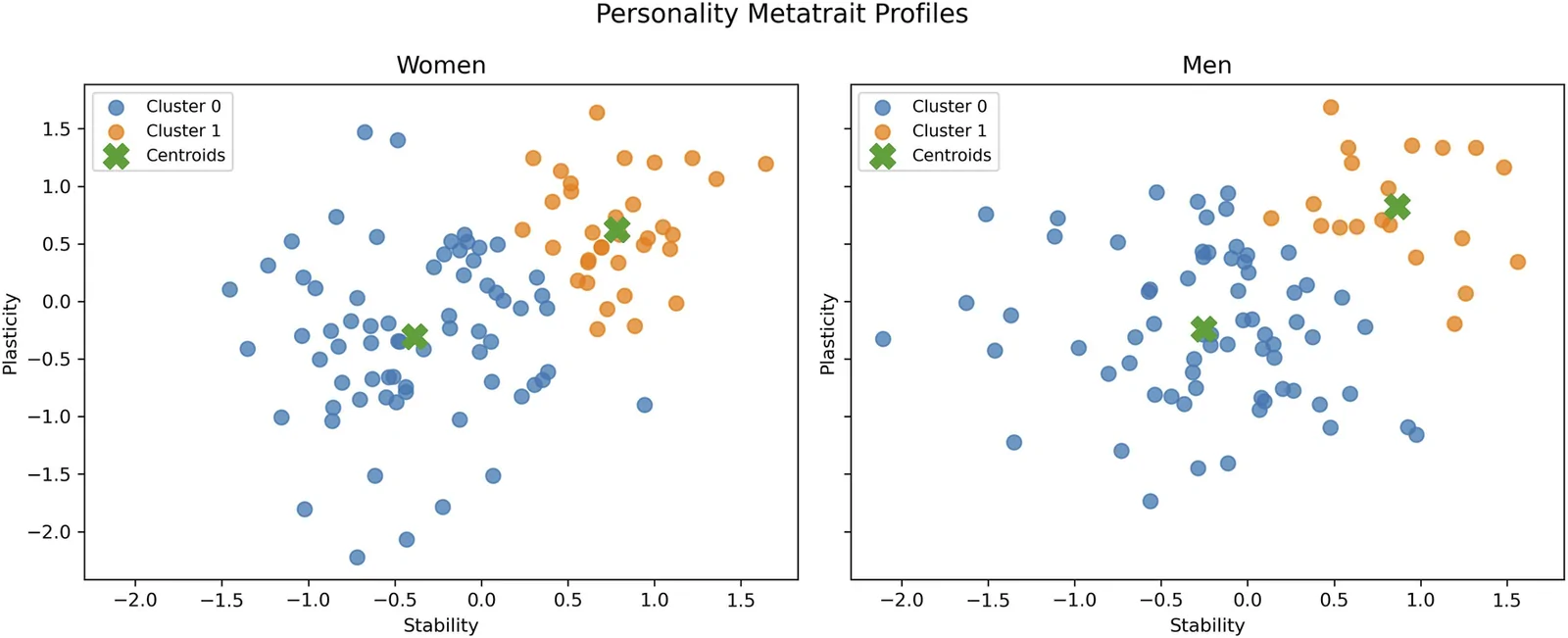
Distribution of personality metatraits (Stability and Plasticity) across identified clusters in women and men. Clusters were derived using hierarchical clustering based directly on Stability and Plasticity scores without prior dimensionality reduction. The figure illustrates the separation of personality profiles between identified clusters within each sex.

#### 3.1.1 Genotype distribution across metatrait clusters

Table 4 presents the distribution of APOE haplotypes, APOE *ε*4 carrier status, PICALM rs3851179 genotypes, and PICALM GG carrier status across the identified personality metatrait clusters. Overall, genotype distributions were comparable between clusters in women and in the total sample. In men, a significant difference was observed only for the full distribution of APOE haplotypes (*χ* ^2^ = 15.77, *p* = 0.008), whereas APOE *ε*4 carrier status and PICALM distributions did not differ significantly between clusters. These findings indicate that the identified metatrait clusters were generally not characterized by systematic differences in genetic risk factor frequencies.

**TABLE 4:** Distribution of APOE and PICALM genotypes across metatrait clusters stratified by sex.

Sex	Genetic marker	Cluster 0	Cluster 1	χ^2^ (df), p
Women	APOE haplotype	​	​	2.086 (4), 0.720
	e2/e4	1 (1.4%)	0 (0.0%)	​
	e3/e2	7 (10.1%)	3 (8.8%)	​
	e3/e3	44 (63.8%)	21 (61.8%)	​
	e3/e4	15 (21.7%)	10 (29.4%)	​
	APOE ε4 carrier status	​	​	0.372 (1), 0.542
	Non-carriers	54 (78.3%)	24 (70.6%)	​
	ε4 carriers	15 (21.7%)	10 (29.4%)	​
	PICALM rs3851179 genotype	​	​	7.195 (3), 0.066
	A/A	7 (10.1%)	1 (2.9%)	​
	G/A	26 (37.7%)	22 (64.7%)	​
	G/G	34 (49.3%)	10 (29.4%)	​
	PICALM GG carrier status	​	​	2.906 (1), 0.088
	Non-GG	35 (50.7%)	24 (70.6%)	​
	GG carriers	34 (49.3%)	10 (29.4%)	​
Men	APOE haplotype	​	​	15.772 (5), 0.008
	e2/e2	1 (1.5%)	0 (0.0%)	​
	e2/e4	0 (0.0%)	2 (10.0%)	​
	e3/e2	6 (8.8%)	3 (15.0%)	​
	e3/e3	43 (63.2%)	10 (50.0%)	​
	e3/e4	18 (26.5%)	3 (15.0%)	​
	e4/e4	0 (0.0%)	2 (10.0%)	​
	APOE ε4 carrier status	​	​	0.000 (1), 1.000
	Non-carriers	50 (73.5%)	15 (75.0%)	​
	ε4 carriers	18 (26.5%)	5 (25.0%)	​
	PICALM rs3851179 genotype	​	​	0.392 (2), 0.822
	A/A	6 (8.8%)	2 (10.0%)	​
	G/A	36 (52.9%)	9 (45.0%)	​
	G/G	26 (38.2%)	9 (45.0%)	​
	PICALM GG carrier status	​	​	0.080 (1), 0.777
	Non-GG	42 (61.8%)	11 (55.0%)	​
	GG carriers	26 (38.2%)	9 (45.0%)	​
Total sample	APOE haplotype	​	​	8.773 (6), 0.187
	e2/e2	1 (0.7%)	0 (0.0%)	​
	e2/e4	1 (0.7%)	2 (3.7%)	​
	e3/e2	13 (9.5%)	6 (11.1%)	​
	e3/e3	87 (63.5%)	31 (57.4%)	​
	e3/e4	33 (24.1%)	13 (24.1%)	​
	e4/e4	0 (0.0%)	2 (3.7%)	​
	APOE ε4 carrier status	​	​	0.119 (1), 0.731
	Non-carriers	104 (75.9%)	39 (72.2%)	​
	ε4 carriers	33 (24.1%)	15 (27.8%)	​
	PICALM rs3851179 genotype	​	​	2.622 (3), 0.454
	A/A	13 (9.5%)	3 (5.6%)	​
	G/A	62 (45.3%)	31 (57.4%)	​
	G/G	60 (43.8%)	19 (35.2%)	​
	PICALM GG carrier status	​	​	0.856 (1), 0.355
	Non-GG	77 (56.2%)	35 (64.8%)	​
	GG carriers	60 (43.8%)	19 (35.2%)	​

Values are presented as n (%).

#### 3.1.2 Cognitive performance across personality metatrait clusters

No statistically significant differences between clusters survived FDR correction in either women or men for any of the examined neuropsychological outcomes (CVLT indices and RPM).

In general, the results indicate a lack of reliable cognitive differentiation between the metatrait-derived clusters. The detailed results are presented in Table 5.

**TABLE 5:** Comparison of neuropsychological performance between personality metatrait clusters.

Sex	Outcome	NC0	NC1	Mean C0	Mean C1	Cohen’s *d*	*p*_FDR_
Women	CVLT-1	69	34	64.33	65.91	0.20	0.684
	CVLT-2	69	34	10.00	10.24	0.12	0.684
	CVLT-3	69	34	14.39	14.71	0.20	0.636
	CVLT-4	69	34	8.62	8.88	0.13	0.684
	CVLT-5	69	34	13.59	13.94	0.16	0.684
	CVLT-6	69	34	14.00	13.94	−0.04	0.862
	CVLT-7	69	34	14.09	14.59	0.27	0.636
	CVLT-8	69	34	14.09	14.38	0.19	0.636
	CVLT-9	69	34	5.17	3.59	−0.31	0.633
	CVLT-10	69	34	0.90	1.12	0.13	0.684
	CVLT-11	69	34	0.36	0.56	0.24	0.636
	CVLT-12	68	34	15.47	15.71	0.31	0.633
	CVLT-13	68	34	0.40	0.35	−0.05	0.862
	RPM	69	34	52.23	52.41	0.04	0.862
Men	CVLT-1	68	20	58.50	57.70	−0.08	0.865
	CVLT-2	68	20	8.79	9.00	0.11	0.865
	CVLT-3	68	20	13.32	12.75	−0.24	0.865
	CVLT-4	68	20	7.84	7.55	−0.15	0.865
	CVLT-5	68	20	11.65	11.60	−0.02	0.947
	CVLT-6	68	20	12.75	13.00	0.11	0.865
	CVLT-7	68	20	12.35	12.75	0.14	0.865
	CVLT-8	68	20	12.94	13.20	0.11	0.865
	CVLT-9	68	20	3.50	3.30	−0.06	0.865
	CVLT-10	68	20	1.47	0.75	−0.45	0.511
	CVLT-11	68	20	1.18	1.00	−0.09	0.865
	CVLT-12	68	20	15.19	15.10	−0.07	0.865
	CVLT-13	68	20	0.72	0.55	−0.14	0.865
	RPM	68	20	53.00	54.50	0.39	0.511

*Abbreviations:* CVLT, California Verbal Learning Test; RPM, Raven’s Progressive Matrices.

### 3.2 Cluster-based genetic regression analyses for female and male participants

After correction for the false discovery rate (*p*_*F*__*DR*_ < 0.05), seven regression models remained statistically significant in the female subsample.

In Cluster 0, the APOE *×* age model (Model 1b) predicting CVLT Recognition–Total Hits performance (CVLT_12) remained significant after FDR correction, explaining 25.4% of the variance in recognition memory performance (*R* ^2^ = 0.254, *p*_*F*__*DR*_ = 0.019).

In Cluster 1, significant associations were observed for both episodic memory and fluid intelligence measures. For CVLT_12, the APOE *×* age *×* education model (Model 1c), the PICALM *×* age *×* education model (Model 2c), and the combined APOE *×* PICALM *×* age *×* education model (Model 3c) remained significant, explaining 58.3–61.9% of the variance in recognition memory performance (*p*_*F*__*DR*_ = 0.010–0.019).

For RPM performance, the APOE *×* age *×* education model (Model 1c), the PICALM *×* age *×* education model (Model 2c), and the combined APOE *×* PICALM *×* age *×* education model (Model 3c) remained significant, explaining 55.3–69.6% of the variance in fluid intelligence performance (*p*_*F*__*DR*_ = 0.001–0.019).

No significant effects survived FDR correction for the simpler genetic models (Models 1a, 2a, and 3a) or for models without age and education interactions. Detailed regression coefficients and confidence intervals for FDR-significant models are presented in Table 6. All tested models, including non-significant findings, are reported in the Supplementary Materials (Supplementary Table S1: All Regression Models).

**TABLE 6:** Regression models surviving false discovery rate correction in women.

Predictor	β	SE	95% CI	*p*	R^2^	*p*_FDR_
Cluster 0 – CVLT-12						
*Model: APOE × Age*						
APOE	−0.382	0.230	[-0.842, 0.078]	0.102	0.254	0.019
APOE × Age	−0.229	0.068	[-0.366, −0.092]	0.001	0.254	0.019
Cluster 1 – CVLT-12						
*Model: APOE × Age × Education*						
Education	0.322	0.136	[0.043, 0.601]	0.025	0.583	0.014
Age × Education	0.163	0.042	[0.076, 0.250]	<0.001	0.583	0.014
*Model: PICALM × Age × Education*						
Age × Education	0.159	0.042	[0.073, 0.244]	<0.001	0.609	0.010
*Model: APOE × PICALM × Age × Education*						
Age × Education	0.157	0.042	[0.071, 0.244]	<0.001	0.619	0.019
Cluster 1 – RPM						
*Model: APOE × Age × Education*						
APOE × Age	−1.594	0.402	[-2.420, −0.768]	<0.001	0.696	0.001
APOE × Age × Education	−0.465	0.117	[-0.706, −0.224]	<0.001	0.696	0.001
*Model: PICALM × Age × Education*						
Age	−0.727	0.282	[-1.307, −0.148]	0.016	0.553	0.019
Age × Education	0.883	0.370	[0.123, 1.643]	0.025	0.553	0.019
*Model: APOE × PICALM × Age × Education*						
APOE × PICALM × Age	−1.433	0.593	[-2.658, −0.209]	0.024	0.625	0.010
Age × Education	0.921	0.345	[0.208, 1.633]	0.013	0.625	0.010

Retression estimates (β), standard errors (SE), 95% confidence intervals (CI), model R2, and FDR-adjusted p-values are presented.

In the male sample, none of the tested regression models remained statistically significant after Benjamini–Hochberg false discovery rate correction. Complete regression results for all models are reported in the Supplementary Materials (Supplementary Table S1: All Regression Models).

#### 3.2.1 Continuous metatrait associations with cognitive performance

Complementary regression analyses were conducted to examine whether Stability and Plasticity, treated as continuous personality dimensions, showed independent linear associations with cognitive performance. After correction for multiple comparisons using the Benjamini–Hochberg False Discovery Rate procedure, no significant main effects of Stability or Plasticity were identified across examined neuropsychological outcomes.

Only one metatrait-related association remained significant after FDR correction: the interaction between PICALM genotype and Plasticity predicting perseverative errors on the CVLT-9. This association was observed both in the PICALM-only model (*β* = −2.97, *p*_*F*__*DR*_ = 0.013) and in the combined APOE + PICALM model (*β* = −2.93, *p*_*F*__*DR*_ = 0.014) (see Supplementary Materials; Supplementary Tables S7, S8, all models in Supplementary Table S2).

Thus, although individual metatraits showed limited linear associations with cognitive performance, these analyses complemented the cluster-based approach by testing whether cognitive differences were better captured by continuous personality dimensions or by combined Stability–Plasticity profiles.

## 4 Discussion

The present study was motivated by theoretical perspectives suggesting that individual differences may not always be optimally represented as continuous trait dimensions, but may also be meaningfully described through qualitatively distinct personality configurations (Kagan, 1998). Rather than focusing on isolated Big Five traits, we examined higher-order personality metatraits, Stability and Plasticity (Digman, 1997; DeYoung et al., 2015; DeYoung et al., 2026), as potential markers of heterogeneity in cognitive aging. Furthermore, we investigate whether these personality configurations interact with genetic susceptibility factors associated with AD, namely, the APOE*ε*3/*ε*4 genotype and the PICALM rs3851179 GG genotype, while adjusting for age and educational attainment.

Among females characterized by lower levels of Stability and Plasticity (Cluster 0), the significant association involved recognition memory performance measured by the CVLT recognition score (CVLT-12). The model related to APOE *ε*4-related which included age explained a substantial proportion of variance in recognition performance, with the interaction between the APOE carrier status and age representing the strongest predictor within this model. These findings are consistent with previous evidence linking the APOE *ε*4 allele to poorer recognition memory in both cognitively healthy individuals and clinical populations (Bednorz et al., 2026; Hirono et al., 2003).

A different pattern was observed among females characterized by higher levels of Stability and Plasticity (Cluster 1). In this subgroup, significant associations were identified primarily for fluid intelligence. Models incorporating APOE, PICALM, age, and education explained a substantial proportion of variance in RPM performance. The observed associations between genetic variants and fluid intelligence, as assessed by the RPM, underscore the potential of cognitive ability measures to capture early gene–cognition interactions. Although intelligence tests are less commonly used in the diagnostic assessment of MCI and AD (Ruchinskas, 2019; Boccardi et al., 2022), they can provide valuable information on interindividual differences in cognitive aging and are highly heritable, reflecting broad genetic influences that extend across cognitive domains (Plomin and Deary, 2015). The observed associations involved interactions between genetic and demographic variables, suggesting that the contribution of genetic susceptibility factors to cognitive performance may depend on the larger developmental context, including educational attainment.

Although this finding should be interpreted cautiously given the limited sample size, it suggests that integrating non-cognitive characteristics, such as personality, with genetic susceptibility may provide a broader perspective on individual differences in cognitive aging. Individuals with lower Stability and Plasticity may represent a personality configuration characterized by reduced emotional regulation capacity, lower behavioral flexibility, and potentially reduced engagement in cognitively stimulating activities (Aschwanden et al., 2021; Bednorz and Religa, 2026; Terracciano et al., 2014). Stability and Plasticity reflect broad dimensions related to affective regulation, goal maintenance, exploratory behavior, and adaptation to changing environmental demands (DeYoung et al., 2015, 2006). Therefore, lower levels of these metatraits may be associated with lifelong differences in health-related behaviors, stress regulation, social engagement, and participation in cognitively enriching experiences. These factors are considered important contributors to cognitive reserve, which influences resilience against age-related brain changes and neuropathological burden (Stern, 2009; 2012; Soldan et al., 2019; Sharp et al., 2010; Soubelet and Salthouse, 2011).

Over time, reduced cognitive reserve can increase vulnerability to subtle changes in memory systems, particularly those involving hippocampal-dependent processes. Recognition memory depends on the coordinated contribution of recollection and familiarity processes, which involve partially distinct but interacting medial temporal lobe structures, including the hippocampus and perirhinal cortex (Tulving, 1985; Yonelinas, 2002; Yonelinas et al., 2010; Squire et al., 2007; Brown and Aggleton, 2001). In addition, recognition memory depends in part on hippocampal computations such as pattern separation and pattern completion, which support the discrimination of similar experiences and the retrieval of complete memory representations from partial cues, respectively (Leal and Yassa, 2018; Ngo et al., 2021). Thus, individual differences in cognitive reserve and environmental enrichment may influence the degree to which genetic susceptibility factors are expressed at the behavioral level.

This finding is consistent with evidence indicating that cognitive abilities are highly polygenic traits whose expression is influenced by demographic and environmental factors such as age, sex, and education (Kang et al., 2023; Raffington et al., 2020; Hindley et al., 2023; Girault et al., 2024). Previous studies suggest that the cognitive consequences of APOE *ε*4 status are strongly age-dependent, with more pronounced differences emerging during later stages of aging as the cumulative neuropathological burden increases. Consequently, carriers and non-carriers can follow different trajectories of cognitive change, indicating that age is an important modifier of the cognitive effects associated with the APOE *ε*4 allele (Hao-Jie et al., 2025; Fan et al., 2019). However, longitudinal studies indicate that subtle memory alterations between APOE *ε*4 carriers may appear as early as midlife, sometimes before the age of 60, even in the absence of clinical impairment, whereas accelerated cognitive decline relative to non-carriers becomes more apparent at older ages (Caselli et al., 2009; Chung et al., 2026). Although APOE remains the strongest common genetic risk factor for late-onset AD, its effects on cognition appear to be context-dependent and may vary according to environmental exposures and individual characteristics (Kang et al., 2023; Sherva et al., 2020). Education represents one of the most important contributors to cognitive reserve and may partially buffer the effects of genetic vulnerability on cognitive aging (Walsemann et al., 2025; Pettigrew et al., 2023; Eid et al., 2019; Groot et al., 2025). At the same time, educational attainment is not exclusively an environmental factor, as genetic studies indicate moderate heritability of educational achievement while also demonstrating its strong association with the risk of dementia (Ayorech et al., 2017; Livingston et al., 2024). The relatively high educational attainment of the present cohort may have further contributed to a reduced variability in cognitive outcomes and potentially attenuated genetic effects. Education is considered one of the main contributors to cognitive reserve and can partially compensate for genetic susceptibility by supporting more efficient cognitive functioning despite biological vulnerability (Stern, 2009, 2012).

The limited number of robust genetic associations observed in the present study may reflect the interaction between a relatively early stage of aging, preserved cognitive functioning, and protective environmental factors, rather than the absence of biological effects. Furthermore, women generally outperform men in episodic memory throughout adulthood and older age (Arenaza-Urquijo et al., 2024; Anstey et al., 2021; Siedlecki et al., 2019; Emrani and Sundermann, 2025; Toopchiani et al., 2025; Ferreira et al., 2014), which can reduce the sensitivity of memory tests to detect early changes related to AD in females. Furthermore, the Big Five model is derived from the lexical hypothesis and based on self-report evaluation, providing a descriptive rather than a mechanistic account of personality (Allport and Odbert, 1936; Cattell, 1945; DeYoung and Gray, 2009; Bednorz and Religa, 2026).

In general, the present findings support a multidimensional view of cognitive aging, in which genetic susceptibility, personality characteristics, and environmental factors jointly contribute to individual variability in cognitive performance. Rather than indicating that personality traits directly modify genetic risk, the observed subgroup patterns suggest that personality metatraits may represent one of the individual characteristics associated with variability in the way genetic and environmental factors are expressed at the cognitive level.

## 5 Limitations

Several limitations should be considered when interpreting the present findings. First, the sample size was relatively modest, particularly after stratification by sex and metatrait cluster, which reduced statistical power and limited the ability to detect small effects. Although an *a priori* power analysis indicated that the obtained cluster sizes were close to the recommended thresholds for exploratory subgroup analyses, the study remained underpowered to detect more subtle associations. Furthermore, the neuropsychological assessment was limited to selected cognitive domains, primarily memory and fluid intelligence, and no biomarker data were available to complement the behavioral measures.

Second, cluster robustness was evaluated using bootstrap resampling and comparisons with alternative clustering algorithms (average linkage, complete linkage, k-means, and Gaussian mixture models; see Supplementary Materials, Table S2). The results indicated moderate overall stability, with mean bootstrap-adjusted Rand indices (ARI) of 0.451 in females and 0.552 in males, and mean individual assignment stability of 63.5% and 76.6%, respectively. Agreement across clustering algorithms varied depending on the method. In particular, average linkage produced a highly similar solution in the male subsample (ARI = 0.950), whereas agreement with k-means and Gaussian mixture models was only moderate in both sexes. Thus, although the identified clusters appear reasonably robust, they remain partly dependent on the clustering algorithm. Replication in larger, independent cohorts is therefore warranted.

Third, the operationalization of personality metatraits was necessarily simplified. The clustering procedure identified primarily concordant Stability–Plasticity profiles (i.e., low–low and high–high) and did not explicitly examine mixed configurations. Consequently, the identified clusters may not fully capture the multidimensional nature of personality organization (Digman, 1997; DeYoung et al., 2010; DeYoung et al., 2015).

Fourth, null findings are reported alongside statistically significant results. Given increasing concerns regarding publication bias and the preferential publication of positive findings, reporting nonsignificant results is essential for cumulative science and helps to avoid inflated estimates of effect sizes (Ioannidis, 2005; Franco et al., 2014; Scheel et al., 2021).

Finally, the decision to perform sex-stratified analyses rather than include sex as an additional covariate was motivated by both biological and statistical considerations. Given previous evidence suggesting sex-dependent effects of APOE, PICALM, and personality-related factors on cognitive ageing, separate analyses allowed us to examine potentially distinct patterns of associations within women and men. From a statistical perspective, adding sex to regression models that already included multiple higher-order interaction terms (e.g., APOE × PICALM × age × education) would have further increased model complexity, reduced interpretability, and potentially contributed to unstable parameter estimates and overfitting, particularly given the moderate sample size (Gelman and Hill, 2007; Harrell, 2015; Babyak, 2004). Although modelling sex as a covariate or formally testing sex-by-predictor interactions could provide a more direct assessment of moderation effects, such approaches would require larger samples to reliably estimate higher-order interactions. Therefore, the analytical strategy adopted here represents a balance between investigating biologically plausible sex-specific patterns and maintaining model interpretability and statistical stability.

Future research should aim to incorporate a broader range of cognitive and non-cognitive variables (Hindley et al., 2023). It may also be important to include affective factors such as anxiety symptoms (Gao et al., 2025), as well as established modifiable risk factors for dementia (Livingston et al., 2024). In addition, future studies could benefit from integrating routinely collected clinical and medical record data (Bednorz et al., 2023; Richter-Laskowska et al., 2025), which can improve participant characterization and support more accurate subgroup classification in aging and dementia.

## 6 Conclusion

In conclusion, our findings indicate that the relationship between genetic risk factors and cognitive performance in healthy middle-aged adults is not uniform but depends on individual cognitive-personality profiles and demographic context. In women, distinct metatrait-based clusters showed different patterns of genetic and environmental associations with episodic memory and fluid intelligence. APOE-related models were associated with recognition memory performance, particularly in interaction with age, whereas education-related factors showed a substantial contribution to cognitive outcomes in the cluster characterized by high Stability and high Plasticity.

Importantly, the effects of APOE and PICALM were not observed as isolated genetic associations but emerged within multivariate models that incorporate age and education, suggesting that genetic susceptibility can influence cognitive performance in the context of developmental and reserve-related factors. The observed associations were robust to false discovery rate correction and sensitivity analyses addressing the restricted distribution of CVLT recognition scores.

These findings support a multidimensional view of cognitive aging, in which genetic risk, personality metatraits, and environmental factors interact to shape individual variability in cognitive performance before the onset of clinical impairment.

## Data Availability

The original dataset has been made publicly available at: https://openneuro.org/datasets/ds004796/versions/1.0.4. Further information about the dataset can be found here: https://doi.org/10.1038/s41597-024-03106-5.
