# Supplementary material for: Sex-specific personality metatrait profiles and their associations with APOE, PICALM variants, and cognitive performance in healthy middle-aged adults: a cluster-based analysis
Source: Front Aging. 2026 Oct 29;7:1933176. doi: 10.3389/fragi.2026.1933176 (PMC13754840; doi:10.3389/fragi.2026.1933176)
Supplement: Supplementary file 1 [file DataSheet1.pdf]

Outcome	Model	N	Predictor	Beta	SE	t	p	CI_low	CI_high	R2	Adj_R2	F	Model_p	pFDR_coef	Significant_FDR
CVLT_1	APOE	173	Intercept	58,59649	1,039496	56,37008	6,5E-108	56,54368	60,6493	0,169284	0,112527	2,982605	0,00124	4,758E-106	TRUE
CVLT_1	APOE	173	sex	6,445966	1,310971	4,91694	2,15E-06	3,857049	9,034882	0,169284	0,112527	2,982605	0,00124	3,011E-05	TRUE
CVLT_1	APOE	173	Plasticity	1,255762	1,057323	1,187681	0,236708	-0,83225	3,343771	0,169284	0,112527	2,982605	0,00124	0,69985965	FALSE
CVLT_1	APOE	173	age_c	-0,26317	0,24216	-1,08674	0,278775	-0,74139	0,215054	0,169284	0,112527	2,982605	0,00124	0,74237363	FALSE
CVLT_1	APOE	173	APOE:Stabi	2,413308	2,374911	1,016168	0,311074	-2,27669	7,103301	0,169284	0,112527	2,982605	0,00124	0,78167367	FALSE
CVLT_1	APOE	173	education_	1,167617	1,157336	1,008883	0,314545	-1,1179	3,453134	0,169284	0,112527	2,982605	0,00124	0,78703081	FALSE
CVLT_1	APOE	173	APOE:age_	-0,37493	0,476871	-0,78623	0,43289	-1,31666	0,5668	0,169284	0,112527	2,982605	0,00124	0,87170999	FALSE
CVLT_1	APOE	173	APOE:Plast	-1,54698	2,221513	-0,69636	0,487207	-5,93404	2,840086	0,169284	0,112527	2,982605	0,00124	0,88419011	FALSE
CVLT_1	APOE	173	APOE	0,561491	1,513071	0,371094	0,711055	-2,42653	3,549516	0,169284	0,112527	2,982605	0,00124	0,9332602	FALSE
CVLT_1	APOE	173	APOE:educ	0,787884	2,47914	0,317805	0,751044	-4,10794	5,68371	0,169284	0,112527	2,982605	0,00124	0,95185346	FALSE
CVLT_1	APOE	173	Stability	-0,27305	1,244947	-0,21933	0,826671	-2,73158	2,185478	0,169284	0,112527	2,982605	0,00124	0,97621258	FALSE
CVLT_1	APOE	173	age_c:educ	-0,05927	0,325144	-0,18228	0,855595	-0,70136	0,58283	0,169284	0,112527	2,982605	0,00124	0,97621258	FALSE
CVLT_1	APOE_PICALM	173	Intercept	58,0604	1,199842	48,39005	1,89E-95	55,69025	60,43056	0,215694	0,129674	2,507471	0,001559	6,9386E-94	TRUE
CVLT_1	APOE_PICALM	173	sex	6,151519	1,322519	4,651366	7,02E-06	3,539033	8,764006	0,215694	0,129674	2,507471	0,001559	8,7814E-05	TRUE
CVLT_1	APOE_PICALM	173	APOE:PICA	-7,59396	3,137317	-2,42053	0,016654	-13,7914	-1,39655	0,215694	0,129674	2,507471	0,001559	0,12554603	FALSE
CVLT_1	APOE_PICALM	173	APOE	4,455856	2,071276	2,151261	0,033005	0,364284	8,547428	0,215694	0,129674	2,507471	0,001559	0,22053195	FALSE
CVLT_1	APOE_PICALM	173	APOE:Stabi	3,809502	2,44026	1,561105	0,120538	-1,01096	8,629959	0,215694	0,129674	2,507471	0,001559	0,50626125	FALSE
CVLT_1	APOE_PICALM	173	PICALM:Pla	2,319487	1,854354	1,250833	0,21288	-1,34358	5,982554	0,215694	0,129674	2,507471	0,001559	0,69007247	FALSE
CVLT_1	APOE_PICALM	173	PICALM:Sta	-2,39589	2,268427	-1,05619	0,292525	-6,87691	2,08513	0,215694	0,129674	2,507471	0,001559	0,76435261	FALSE
CVLT_1	APOE_PICALM	173	education_	1,500086	1,494611	1,003664	0,317105	-1,45235	4,452521	0,215694	0,129674	2,507471	0,001559	0,79007542	FALSE
CVLT_1	APOE_PICALM	173	PICALM	1,275053	1,549507	0,822876	0,411843	-1,78582	4,33593	0,215694	0,129674	2,507471	0,001559	0,86252891	FALSE
CVLT_1	APOE_PICALM	173	APOE:age_	-0,37546	0,47633	-0,78824	0,431758	-1,3164	0,565473	0,215694	0,129674	2,507471	0,001559	0,87170999	FALSE
CVLT_1	APOE_PICALM	173	APOE:Plast	-1,56688	2,212728	-0,70812	0,479934	-5,93787	2,804118	0,215694	0,129674	2,507471	0,001559	0,87971762	FALSE
CVLT_1	APOE_PICALM	173	age_c	-0,17059	0,293963	-0,58032	0,562539	-0,75128	0,410096	0,215694	0,129674	2,507471	0,001559	0,89894588	FALSE
CVLT_1	APOE_PICALM	173	PICALM:ag	-0,24598	0,428977	-0,57342	0,567192	-1,09338	0,601413	0,215694	0,129674	2,507471	0,001559	0,89894588	FALSE
CVLT_1	APOE_PICALM	173	APOE:educ	1,410562	2,579187	0,546902	0,585233	-3,68433	6,505455	0,215694	0,129674	2,507471	0,001559	0,90557047	FALSE
CVLT_1	APOE_PICALM	173	Stability	0,735218	1,466554	0,501323	0,616855	-2,16179	3,632229	0,215694	0,129674	2,507471	0,001559	0,90982023	FALSE
CVLT_1	APOE_PICALM	173	PICALM:ed	-0,70131	2,203915	-0,31821	0,750752	-5,0549	3,652273	0,215694	0,129674	2,507471	0,001559	0,95185346	FALSE
CVLT_1	APOE_PICALM	173	age_c:educ	-0,06655	0,335199	-0,19855	0,842874	-0,7287	0,595593	0,215694	0,129674	2,507471	0,001559	0,97621258	FALSE
CVLT_1	APOE_PICALM	173	Plasticity	0,133099	1,384723	0,096119	0,92355	-2,60227	2,868463	0,215694	0,129674	2,507471	0,001559	0,98995361	FALSE
CVLT_1	PICALM	173	Intercept	58,78123	1,120662	52,45225	3,9E-103	56,56814	60,99433	0,170084	0,113382	2,999596	0,00117	1,903E-101	TRUE
CVLT_1	PICALM	173	sex	6,360546	1,316566	4,831164	3,14E-06	3,760581	8,960512	0,170084	0,113382	2,999596	0,00117	4,2937E-05	TRUE
CVLT_1	PICALM	173	education_	1,845495	1,280838	1,440849	0,151569	-0,68392	4,374905	0,170084	0,113382	2,999596	0,00117	0,58349532	FALSE
CVLT_1	PICALM	173	PICALM:Pla	2,249026	1,851187	1,21491	0,226179	-1,40671	5,904765	0,170084	0,113382	2,999596	0,00117	0,69315053	FALSE
CVLT_1	PICALM	173	Stability	1,180181	1,367371	0,863102	0,389365	-1,52011	3,880477	0,170084	0,113382	2,999596	0,00117	0,85180491	FALSE
CVLT_1	PICALM	173	age_c	-0,22518	0,27272	-0,82569	0,4102	-0,76375	0,313387	0,170084	0,113382	2,999596	0,00117	0,86252891	FALSE
CVLT_1	PICALM	173	PICALM:ag	-0,34701	0,423396	-0,81959	0,413662	-1,18314	0,489115	0,170084	0,113382	2,999596	0,00117	0,86252891	FALSE
CVLT_1	PICALM	173	PICALM:Sta	-1,65488	2,201958	-0,75155	0,453418	-6,00333	2,693562	0,170084	0,113382	2,999596	0,00117	0,87396458	FALSE

CVLT_1	PICALM	173	PICALM:ed	-0,88277	2,13602	-0,41328	0,679954	-5,10099	3,335463	0,170084	0,113382	2,999596	0,00117	0,91438395	FALSE
CVLT_1	PICALM	173	age_c:educ	-0,08776	0,331602	-0,26464	0,791622	-0,74261	0,567094	0,170084	0,113382	2,999596	0,00117	0,96374848	FALSE
CVLT_1	PICALM	173	PICALM	-0,29141	1,316997	-0,22127	0,825163	-2,89223	2,309405	0,170084	0,113382	2,999596	0,00117	0,97621258	FALSE
CVLT_1	PICALM	173	Plasticity	-0,23983	1,279976	-0,18737	0,851607	-2,76753	2,287879	0,170084	0,113382	2,999596	0,00117	0,97621258	FALSE
CVLT_10	APOE	173	Intercept	1,311901	0,209023	6,276357	3,08E-09	0,899121	1,72468	0,038777	-0,0269	0,590446	0,834754	5,4911E-08	TRUE
CVLT_10	APOE	173	sex	-0,30869	0,263611	-1,17099	0,243332	-0,82927	0,211894	0,038777	-0,0269	0,590446	0,834754	0,70915764	FALSE
CVLT_10	APOE	173	APOE:age_	0,111356	0,09589	1,161296	0,24724	-0,07801	0,30072	0,038777	-0,0269	0,590446	0,834754	0,70915764	FALSE
CVLT_10	APOE	173	education_	-0,24862	0,232718	-1,06832	0,286976	-0,70819	0,210957	0,038777	-0,0269	0,590446	0,834754	0,76009946	FALSE
CVLT_10	APOE	173	APOE:Stabi	-0,43958	0,477549	-0,9205	0,35869	-1,38265	0,503485	0,038777	-0,0269	0,590446	0,834754	0,81501357	FALSE
CVLT_10	APOE	173	APOE:Plast	0,251818	0,446703	0,563726	0,573725	-0,63034	1,133972	0,038777	-0,0269	0,590446	0,834754	0,90166392	FALSE
CVLT_10	APOE	173	APOE	-0,15654	0,304249	-0,51452	0,607596	-0,75738	0,444293	0,038777	-0,0269	0,590446	0,834754	0,90982023	FALSE
CVLT_10	APOE	173	Stability	0,120713	0,250335	0,482205	0,630315	-0,37365	0,615076	0,038777	-0,0269	0,590446	0,834754	0,90982023	FALSE
CVLT_10	APOE	173	age_c	0,02502	0,048694	0,513821	0,608082	-0,07114	0,121181	0,038777	-0,0269	0,590446	0,834754	0,90982023	FALSE
CVLT_10	APOE	173	APOE:educ	0,216418	0,498507	0,434132	0,664774	-0,76804	1,200874	0,038777	-0,0269	0,590446	0,834754	0,91328699	FALSE
CVLT_10	APOE	173	Plasticity	-0,07263	0,212607	-0,34161	0,733086	-0,49249	0,347229	0,038777	-0,0269	0,590446	0,834754	0,94625118	FALSE
CVLT_10	APOE	173	age_c:educ	0,00255	0,06538	0,038997	0,968941	-0,12656	0,131663	0,038777	-0,0269	0,590446	0,834754	0,99162101	FALSE
CVLT_10	APOE_PICALM	173	Intercept	1,570438	0,244276	6,42894	1,51E-09	1,087897	2,052978	0,069683	-0,03235	0,682935	0,816272	2,7724E-08	TRUE
CVLT_10	APOE_PICALM	173	PICALM	-0,59415	0,315465	-1,88341	0,061519	-1,21731	0,029017	0,069683	-0,03235	0,682935	0,816272	0,35824532	FALSE
CVLT_10	APOE_PICALM	173	APOE:age_	0,121897	0,096976	1,256972	0,210655	-0,06967	0,313462	0,069683	-0,03235	0,682935	0,816272	0,68813815	FALSE
CVLT_10	APOE_PICALM	173	education_	-0,36594	0,304288	-1,20259	0,230967	-0,96702	0,235152	0,069683	-0,03235	0,682935	0,816272	0,6975188	FALSE
CVLT_10	APOE_PICALM	173	sex	-0,31358	0,269252	-1,16464	0,245956	-0,84546	0,218297	0,069683	-0,03235	0,682935	0,816272	0,70915764	FALSE
CVLT_10	APOE_PICALM	173	APOE	-0,47894	0,421692	-1,13575	0,257814	-1,31194	0,354068	0,069683	-0,03235	0,682935	0,816272	0,72120934	FALSE
CVLT_10	APOE_PICALM	173	APOE:PICA	0,713687	0,638728	1,117357	0,265571	-0,54805	1,975421	0,069683	-0,03235	0,682935	0,816272	0,72294232	FALSE
CVLT_10	APOE_PICALM	173	APOE:Stabi	-0,42491	0,496813	-0,85527	0,393723	-1,40631	0,556491	0,069683	-0,03235	0,682935	0,816272	0,85744166	FALSE
CVLT_10	APOE_PICALM	173	PICALM:ag	0,057243	0,087336	0,655432	0,513161	-0,11528	0,229764	0,069683	-0,03235	0,682935	0,816272	0,89521185	FALSE
CVLT_10	APOE_PICALM	173	PICALM:ed	0,281823	0,448696	0,628094	0,530867	-0,60453	1,168171	0,069683	-0,03235	0,682935	0,816272	0,89687005	FALSE
CVLT_10	APOE_PICALM	173	APOE:educ	0,290733	0,525098	0,553675	0,580599	-0,74654	1,328005	0,069683	-0,03235	0,682935	0,816272	0,90315456	FALSE
CVLT_10	APOE_PICALM	173	APOE:Plast	0,215693	0,45049	0,478795	0,632759	-0,6742	1,105585	0,069683	-0,03235	0,682935	0,816272	0,90982023	FALSE
CVLT_10	APOE_PICALM	173	PICALM:Pla	-0,14908	0,377529	-0,39487	0,693481	-0,89484	0,59669	0,069683	-0,03235	0,682935	0,816272	0,92254883	FALSE
CVLT_10	APOE_PICALM	173	Stability	0,093995	0,298576	0,314812	0,753328	-0,49581	0,683799	0,069683	-0,03235	0,682935	0,816272	0,95185346	FALSE
CVLT_10	APOE_PICALM	173	PICALM:Sta	-0,12221	0,46183	-0,26462	0,791651	-1,0345	0,790082	0,069683	-0,03235	0,682935	0,816272	0,96374848	FALSE
CVLT_10	APOE_PICALM	173	age_c	-0,01462	0,059848	-0,24433	0,807299	-0,13285	0,1036	0,069683	-0,03235	0,682935	0,816272	0,97514073	FALSE
CVLT_10	APOE_PICALM	173	age_c:educ	0,008388	0,068243	0,122913	0,902335	-0,12642	0,143195	0,069683	-0,03235	0,682935	0,816272	0,98158997	FALSE
CVLT_10	APOE_PICALM	173	Plasticity	0,02444	0,281916	0,086693	0,931028	-0,53245	0,581334	0,069683	-0,03235	0,682935	0,816272	0,98995361	FALSE
CVLT_10	PICALM	173	Intercept	1,466685	0,224661	6,528447	8,26E-10	1,023023	1,910346	0,045514	-0,0197	0,69793	0,739264	1,5677E-08	TRUE
CVLT_10	PICALM	173	PICALM	-0,40168	0,26402	-1,52141	0,130118	-0,92307	0,119705	0,045514	-0,0197	0,69793	0,739264	0,5313135	FALSE
CVLT_10	PICALM	173	sex	-0,30178	0,263934	-1,14339	0,254572	-0,823	0,219438	0,045514	-0,0197	0,69793	0,739264	0,71965576	FALSE
CVLT_10	PICALM	173	education_	-0,25607	0,256771	-0,99727	0,320132	-0,76314	0,251005	0,045514	-0,0197	0,69793	0,739264	0,79114198	FALSE
CVLT_10	PICALM	173	PICALM:ag	0,07394	0,084879	0,871123	0,384984	-0,09368	0,241559	0,045514	-0,0197	0,69793	0,739264	0,85023979	FALSE

CVLT_10	PICALM	173	PICALM:ed	0,198167	0,428211	0,46278	0,644147	-0,64747	1,043801	0,045514	-0,0197	0,69793	0,739264	0,90982023	FALSE
CVLT_10	PICALM	173	PICALM:Pla	-0,15234	0,37111	-0,41051	0,681977	-0,88522	0,580526	0,045514	-0,0197	0,69793	0,739264	0,91438395	FALSE
CVLT_10	PICALM	173	PICALM:Sta	-0,15001	0,44143	-0,33983	0,73443	-1,02175	0,721729	0,045514	-0,0197	0,69793	0,739264	0,94625118	FALSE
CVLT_10	PICALM	173	age_c	0,014617	0,054673	0,267346	0,789545	-0,09335	0,122584	0,045514	-0,0197	0,69793	0,739264	0,96374848	FALSE
CVLT_10	PICALM	173	Stability	0,054503	0,274119	0,198831	0,842646	-0,48683	0,595835	0,045514	-0,0197	0,69793	0,739264	0,97621258	FALSE
CVLT_10	PICALM	173	Plasticity	0,05244	0,256598	0,204366	0,838325	-0,45429	0,559173	0,045514	-0,0197	0,69793	0,739264	0,97621258	FALSE
CVLT_10	PICALM	173	age_c:educ	0,008092	0,066477	0,121728	0,903266	-0,12319	0,139371	0,045514	-0,0197	0,69793	0,739264	0,98158997	FALSE
CVLT_11	APOE	173	Intercept	1,015156	0,186773	5,435231	1,99E-07	0,646315	1,383998	0,105418	0,044298	1,724759	0,072154	3,1667E-06	TRUE
CVLT_11	APOE	173	sex	-0,66386	0,235551	-2,81832	0,005433	-1,12903	-0,19869	0,105418	0,044298	1,724759	0,072154	0,04698198	TRUE
CVLT_11	APOE	173	APOE:age_	0,221162	0,085683	2,58117	0,010738	0,051955	0,390369	0,105418	0,044298	1,724759	0,072154	0,08342583	FALSE
CVLT_11	APOE	173	APOE:educ	-0,30558	0,445444	-0,68601	0,493695	-1,18524	0,574088	0,105418	0,044298	1,724759	0,072154	0,8904683	FALSE
CVLT_11	APOE	173	APOE	0,175595	0,271864	0,645893	0,519268	-0,36128	0,712474	0,105418	0,044298	1,724759	0,072154	0,89521185	FALSE
CVLT_11	APOE	173	APOE:Plast	-0,24359	0,399154	-0,61025	0,542554	-1,03184	0,544668	0,105418	0,044298	1,724759	0,072154	0,89687005	FALSE
CVLT_11	APOE	173	Stability	0,134189	0,223688	0,599895	0,54942	-0,30755	0,57593	0,105418	0,044298	1,724759	0,072154	0,89778791	FALSE
CVLT_11	APOE	173	Plasticity	-0,0907	0,189976	-0,47742	0,633712	-0,46587	0,284469	0,105418	0,044298	1,724759	0,072154	0,90982023	FALSE
CVLT_11	APOE	173	age_c	-0,01989	0,043511	-0,45704	0,648262	-0,10581	0,066039	0,105418	0,044298	1,724759	0,072154	0,90982023	FALSE
CVLT_11	APOE	173	age_c:educ	0,021377	0,058421	0,365907	0,714915	-0,09399	0,136746	0,105418	0,044298	1,724759	0,072154	0,93623557	FALSE
CVLT_11	APOE	173	APOE:Stabi	0,116905	0,426716	0,273965	0,784463	-0,72578	0,959588	0,105418	0,044298	1,724759	0,072154	0,96374848	FALSE
CVLT_11	APOE	173	education_	0,044575	0,207947	0,214359	0,830538	-0,36608	0,45523	0,105418	0,044298	1,724759	0,072154	0,97621258	FALSE
CVLT_11	APOE_PICALM	173	Intercept	1,156433	0,215613	5,363456	2,92E-07	0,730513	1,582353	0,155164	0,062505	1,674564	0,052866	4,5142E-06	TRUE
CVLT_11	APOE_PICALM	173	sex	-0,61336	0,237659	-2,58086	0,010783	-1,08283	-0,1439	0,155164	0,062505	1,674564	0,052866	0,08342583	FALSE
CVLT_11	APOE_PICALM	173	APOE:age_	0,221715	0,085597	2,590213	0,010506	0,052627	0,390803	0,155164	0,062505	1,674564	0,052866	0,08342583	FALSE
CVLT_11	APOE_PICALM	173	APOE:PICA	1,227753	0,563781	2,177713	0,030939	0,114067	2,341438	0,155164	0,062505	1,674564	0,052866	0,21329592	FALSE
CVLT_11	APOE_PICALM	173	PICALM:ag	0,119247	0,077088	1,546896	0,123928	-0,03303	0,271525	0,155164	0,062505	1,674564	0,052866	0,51567167	FALSE
CVLT_11	APOE_PICALM	173	age_c	-0,07222	0,052826	-1,36705	0,173587	-0,17657	0,032135	0,155164	0,062505	1,674564	0,052866	0,6223746	FALSE
CVLT_11	APOE_PICALM	173	PICALM	-0,32037	0,278449	-1,15054	0,251694	-0,87041	0,229678	0,155164	0,062505	1,674564	0,052866	0,71495618	FALSE
CVLT_11	APOE_PICALM	173	APOE	-0,41955	0,372212	-1,12719	0,261402	-1,15482	0,315707	0,155164	0,062505	1,674564	0,052866	0,72120934	FALSE
CVLT_11	APOE_PICALM	173	PICALM:Pla	-0,33142	0,33323	-0,99456	0,321499	-0,98968	0,32684	0,155164	0,062505	1,674564	0,052866	0,79114198	FALSE
CVLT_11	APOE_PICALM	173	APOE:educ	-0,44653	0,463484	-0,96343	0,336832	-1,36209	0,469026	0,155164	0,062505	1,674564	0,052866	0,80275452	FALSE
CVLT_11	APOE_PICALM	173	APOE:Plast	-0,25992	0,397631	-0,65367	0,514291	-1,04539	0,525554	0,155164	0,062505	1,674564	0,052866	0,89521185	FALSE
CVLT_11	APOE_PICALM	173	education_	0,083045	0,268584	0,309195	0,757588	-0,44751	0,613602	0,155164	0,062505	1,674564	0,052866	0,95208288	FALSE
CVLT_11	APOE_PICALM	173	Stability	0,079727	0,263542	0,302521	0,762661	-0,44087	0,600324	0,155164	0,062505	1,674564	0,052866	0,95347316	FALSE
CVLT_11	APOE_PICALM	173	Plasticity	0,059074	0,248837	0,237399	0,81266	-0,43248	0,550623	0,155164	0,062505	1,674564	0,052866	0,97519255	FALSE
CVLT_11	APOE_PICALM	173	PICALM:Sta	0,097785	0,40764	0,239882	0,810739	-0,70746	0,903032	0,155164	0,062505	1,674564	0,052866	0,97519255	FALSE
CVLT_11	APOE_PICALM	173	PICALM:ed	-0,09051	0,396047	-0,22852	0,819541	-0,87285	0,69184	0,155164	0,062505	1,674564	0,052866	0,9760277	FALSE
CVLT_11	APOE_PICALM	173	age_c:educ	0,011025	0,060236	0,183035	0,85501	-0,10796	0,130014	0,155164	0,062505	1,674564	0,052866	0,97621258	FALSE
CVLT_11	APOE_PICALM	173	APOE:Stabi	-0,0146	0,438518	-0,0333	0,97348	-0,88085	0,851642	0,155164	0,062505	1,674564	0,052866	0,99162101	FALSE
CVLT_11	PICALM	173	Intercept	1,105804	0,202812	5,45236	1,84E-07	0,705289	1,506318	0,093317	0,03137	1,506402	0,13364	3,0007E-06	TRUE
CVLT_11	PICALM	173	sex	-0,65697	0,238266	-2,7573	0,006502	-1,1275	-0,18644	0,093317	0,03137	1,506402	0,13364	0,05461443	FALSE

CVLT_11	PICALM	173	PICALM:ag	0,162287	0,076624	2,117958	0,035714	0,010969	0,313605	0,093317	0,03137	1,506402	0,13364	0,23333186	FALSE
CVLT_11	PICALM	173	PICALM:Pla	-0,40688	0,335019	-1,2145	0,226335	-1,06848	0,254717	0,093317	0,03137	1,506402	0,13364	0,69315053	FALSE
CVLT_11	PICALM	173	age_c:educ	0,034523	0,060012	0,575279	0,565906	-0,08399	0,153035	0,093317	0,03137	1,506402	0,13364	0,89894588	FALSE
CVLT_11	PICALM	173	age_c	-0,02382	0,049356	-0,48264	0,630008	-0,12129	0,073647	0,093317	0,03137	1,506402	0,13364	0,90982023	FALSE
CVLT_11	PICALM	173	PICALM:Sta	0,189982	0,3985	0,476744	0,634191	-0,59698	0,976943	0,093317	0,03137	1,506402	0,13364	0,90982023	FALSE
CVLT_11	PICALM	173	Stability	0,097815	0,24746	0,395277	0,693162	-0,39087	0,586502	0,093317	0,03137	1,506402	0,13364	0,92254883	FALSE
CVLT_11	PICALM	173	PICALM	0,030765	0,238344	0,12908	0,897456	-0,43992	0,501448	0,093317	0,03137	1,506402	0,13364	0,98158997	FALSE
CVLT_11	PICALM	173	Plasticity	0,027722	0,231644	0,119677	0,904888	-0,42973	0,485174	0,093317	0,03137	1,506402	0,13364	0,98158997	FALSE
CVLT_11	PICALM	173	PICALM:ed	0,0147	0,386566	0,038027	0,969713	-0,74869	0,778095	0,093317	0,03137	1,506402	0,13364	0,99162101	FALSE
CVLT_11	PICALM	173	education_	0,004978	0,2318	0,021475	0,982893	-0,45278	0,462738	0,093317	0,03137	1,506402	0,13364	0,99473515	FALSE
CVLT_12	APOE	172	Intercept	15,28064	0,119088	128,3143	3E-163	15,04545	15,51583	0,083317	0,020295	1,322029	0,216573	1,739E-160	TRUE
CVLT_12	APOE	172	education_	0,249911	0,13248	1,886406	0,061052	-0,01172	0,511546	0,083317	0,020295	1,322029	0,216573	0,35824532	FALSE
CVLT_12	APOE	172	sex	0,267639	0,150317	1,780488	0,076894	-0,02922	0,564501	0,083317	0,020295	1,322029	0,216573	0,39693107	FALSE
CVLT_12	APOE	172	APOE:age_	-0,08978	0,055184	-1,62701	0,105702	-0,19877	0,019198	0,083317	0,020295	1,322029	0,216573	0,4718845	FALSE
CVLT_12	APOE	172	APOE:Plast	0,295459	0,254358	1,161588	0,247133	-0,20687	0,797791	0,083317	0,020295	1,322029	0,216573	0,70915764	FALSE
CVLT_12	APOE	172	age_c:educ	0,04175	0,037223	1,121613	0,263707	-0,03176	0,115261	0,083317	0,020295	1,322029	0,216573	0,72120934	FALSE
CVLT_12	APOE	172	APOE:educ	-0,16552	0,285769	-0,57922	0,563256	-0,72989	0,398842	0,083317	0,020295	1,322029	0,216573	0,89894588	FALSE
CVLT_12	APOE	172	age_c	-0,01263	0,02772	-0,45563	0,649271	-0,06737	0,042114	0,083317	0,020295	1,322029	0,216573	0,90982023	FALSE
CVLT_12	APOE	172	Plasticity	0,04091	0,121031	0,338011	0,735798	-0,19811	0,279933	0,083317	0,020295	1,322029	0,216573	0,94625118	FALSE
CVLT_12	APOE	172	APOE:Stabi	-0,07329	0,275682	-0,26586	0,790692	-0,61774	0,471153	0,083317	0,020295	1,322029	0,216573	0,96374848	FALSE
CVLT_12	APOE	172	APOE	0,02751	0,175526	0,156729	0,875656	-0,31914	0,374156	0,083317	0,020295	1,322029	0,216573	0,98158997	FALSE
CVLT_12	APOE	172	Stability	0,00669	0,142508	0,046943	0,962618	-0,27475	0,288129	0,083317	0,020295	1,322029	0,216573	0,99162101	FALSE
CVLT_12	APOE_PICALM	172	Intercept	15,17892	0,139754	108,6119	2E-147	14,90283	15,455	0,105055	0,006263	1,063393	0,394567	3,826E-145	TRUE
CVLT_12	APOE_PICALM	172	education_	0,357477	0,174203	2,052075	0,041856	0,013342	0,701612	0,105055	0,006263	1,063393	0,394567	0,26751225	FALSE
CVLT_12	APOE_PICALM	172	sex	0,274494	0,154061	1,781716	0,076766	-0,02985	0,57884	0,105055	0,006263	1,063393	0,394567	0,39693107	FALSE
CVLT_12	APOE_PICALM	172	APOE:age_	-0,09107	0,056001	-1,62629	0,105933	-0,2017	0,019556	0,105055	0,006263	1,063393	0,394567	0,4718845	FALSE
CVLT_12	APOE_PICALM	172	PICALM	0,215726	0,180434	1,195595	0,233693	-0,14072	0,57217	0,105055	0,006263	1,063393	0,394567	0,6975188	FALSE
CVLT_12	APOE_PICALM	172	APOE:Plast	0,301753	0,257599	1,171405	0,243245	-0,20713	0,810638	0,105055	0,006263	1,063393	0,394567	0,70915764	FALSE
CVLT_12	APOE_PICALM	172	PICALM:Pla	0,219796	0,215887	1,018109	0,310223	-0,20669	0,646278	0,105055	0,006263	1,063393	0,394567	0,78167367	FALSE
CVLT_12	APOE_PICALM	172	PICALM:ed	-0,2476	0,257658	-0,96095	0,338082	-0,7566	0,261403	0,105055	0,006263	1,063393	0,394567	0,80275452	FALSE
CVLT_12	APOE_PICALM	172	age_c:educ	0,034691	0,039041	0,88858	0,375615	-0,04243	0,111817	0,105055	0,006263	1,063393	0,394567	0,83659659	FALSE
CVLT_12	APOE_PICALM	172	APOE:educ	-0,26071	0,302234	-0,86262	0,389686	-0,85777	0,336346	0,105055	0,006263	1,063393	0,394567	0,85180491	FALSE
CVLT_12	APOE_PICALM	172	Plasticity	-0,07621	0,161255	-0,47259	0,637171	-0,39477	0,242349	0,105055	0,006263	1,063393	0,394567	0,90982023	FALSE
CVLT_12	APOE_PICALM	172	APOE	0,105901	0,241131	0,439185	0,661143	-0,37045	0,582252	0,105055	0,006263	1,063393	0,394567	0,91260662	FALSE
CVLT_12	APOE_PICALM	172	Stability	0,075498	0,171051	0,441379	0,659558	-0,26241	0,413408	0,105055	0,006263	1,063393	0,394567	0,91260662	FALSE
CVLT_12	APOE_PICALM	172	PICALM:Sta	-0,09601	0,267287	-0,35921	0,719927	-0,62404	0,43201	0,105055	0,006263	1,063393	0,394567	0,93861873	FALSE
CVLT_12	APOE_PICALM	172	APOE:PICA	-0,12045	0,373104	-0,32282	0,747268	-0,85751	0,616615	0,105055	0,006263	1,063393	0,394567	0,95185346	FALSE
CVLT_12	APOE_PICALM	172	PICALM:ag	-0,01445	0,050291	-0,28733	0,774245	-0,1138	0,084899	0,105055	0,006263	1,063393	0,394567	0,95843387	FALSE
CVLT_12	APOE_PICALM	172	APOE:Stabi	-0,04645	0,288901	-0,16077	0,872485	-0,61717	0,524273	0,105055	0,006263	1,063393	0,394567	0,98158997	FALSE

CVLT_12	APOE_PICALM	172	age_c	-0,00325	0,034273	-0,09485	0,924558	-0,07096	0,064456	0,105055	0,006263	1,063393	0,394567	0,98995361	FALSE
CVLT_12	PICALM	172	Intercept	15,208	0,129075	117,8229	2,2E-157	14,95309	15,46291	0,0736	0,00991	1,155602	0,322253	6,379E-155	TRUE
CVLT_12	PICALM	172	sex	0,268027	0,151729	1,76649	0,079221	-0,03162	0,567677	0,0736	0,00991	1,155602	0,322253	0,40156629	FALSE
CVLT_12	PICALM	172	education_	0,237485	0,147465	1,610451	0,10927	-0,05374	0,528714	0,0736	0,00991	1,155602	0,322253	0,4759335	FALSE
CVLT_12	PICALM	172	PICALM:Pla	0,224457	0,213127	1,053163	0,293854	-0,19645	0,645361	0,0736	0,00991	1,155602	0,322253	0,76435261	FALSE
CVLT_12	PICALM	172	age_c:educ	0,036575	0,038213	0,957137	0,339942	-0,03889	0,112043	0,0736	0,00991	1,155602	0,322253	0,80275452	FALSE
CVLT_12	PICALM	172	PICALM	0,141781	0,152062	0,932391	0,352539	-0,15853	0,442088	0,0736	0,00991	1,155602	0,322253	0,81110511	FALSE
CVLT_12	PICALM	172	age_c	-0,02576	0,031398	-0,82043	0,413191	-0,08777	0,036248	0,0736	0,00991	1,155602	0,322253	0,86252891	FALSE
CVLT_12	PICALM	172	PICALM:ed	-0,17182	0,246459	-0,69716	0,486713	-0,65855	0,31491	0,0736	0,00991	1,155602	0,322253	0,88419011	FALSE
CVLT_12	PICALM	172	PICALM:Sta	-0,15069	0,255807	-0,58908	0,556636	-0,65588	0,354501	0,0736	0,00991	1,155602	0,322253	0,89894588	FALSE
CVLT_12	PICALM	172	PICALM:ag	-0,02572	0,048899	-0,52597	0,599635	-0,12229	0,070852	0,0736	0,00991	1,155602	0,322253	0,90982023	FALSE
CVLT_12	PICALM	172	Stability	0,048633	0,157425	0,308925	0,75778	-0,26227	0,359531	0,0736	0,00991	1,155602	0,322253	0,95208288	FALSE
CVLT_12	PICALM	172	Plasticity	0,015014	0,147364	0,101886	0,918974	-0,27601	0,306044	0,0736	0,00991	1,155602	0,322253	0,98966478	FALSE
CVLT_13	APOE	172	Intercept	0,630574	0,139968	4,505119	1,27E-05	0,35415	0,906998	0,092439	0,030044	1,481514	0,143029	0,00015605	TRUE
CVLT_13	APOE	172	APOE:educ	-0,63577	0,335875	-1,89288	0,06018	-1,29909	0,027549	0,092439	0,030044	1,481514	0,143029	0,35824532	FALSE
CVLT_13	APOE	172	sex	-0,27281	0,176674	-1,54413	0,124533	-0,62172	0,076107	0,092439	0,030044	1,481514	0,143029	0,51567167	FALSE
CVLT_13	APOE	172	APOE:age_	0,087071	0,06486	1,342459	0,181349	-0,04102	0,215163	0,092439	0,030044	1,481514	0,143029	0,63852151	FALSE
CVLT_13	APOE	172	age_c	0,039996	0,03258	1,227631	0,221389	-0,02435	0,104339	0,092439	0,030044	1,481514	0,143029	0,69257616	FALSE
CVLT_13	APOE	172	Plasticity	-0,12836	0,142252	-0,90234	0,36823	-0,40929	0,152573	0,092439	0,030044	1,481514	0,143029	0,82640959	FALSE
CVLT_13	APOE	172	APOE	0,152058	0,206302	0,737066	0,462162	-0,25537	0,559485	0,092439	0,030044	1,481514	0,143029	0,87396458	FALSE
CVLT_13	APOE	172	APOE:Plast	-0,21081	0,298957	-0,70514	0,48175	-0,80122	0,379605	0,092439	0,030044	1,481514	0,143029	0,87971762	FALSE
CVLT_13	APOE	172	education_	-0,01995	0,155709	-0,12813	0,898204	-0,32746	0,287558	0,092439	0,030044	1,481514	0,143029	0,98158997	FALSE
CVLT_13	APOE	172	APOE:Stabi	0,028937	0,32402	0,089307	0,92895	-0,61097	0,668845	0,092439	0,030044	1,481514	0,143029	0,98995361	FALSE
CVLT_13	APOE	172	Stability	0,005142	0,167495	0,030701	0,975547	-0,32564	0,335928	0,092439	0,030044	1,481514	0,143029	0,99162101	FALSE
CVLT_13	APOE	172	age_c:educ	9,46E-05	0,04375	0,002162	0,998277	-0,08631	0,086496	0,092439	0,030044	1,481514	0,143029	0,99827747	FALSE
CVLT_13	APOE_PICALM	172	Intercept	0,688877	0,161046	4,277518	3,31E-05	0,370733	1,007021	0,148277	0,054255	1,577052	0,076455	0,00037373	TRUE
CVLT_13	APOE_PICALM	172	APOE:PICA	1,016214	0,429947	2,363577	0,019348	0,166858	1,86557	0,148277	0,054255	1,577052	0,076455	0,14220499	FALSE
CVLT_13	APOE_PICALM	172	APOE:educ	-0,64648	0,348281	-1,85619	0,065337	-1,3345	0,041548	0,148277	0,054255	1,577052	0,076455	0,36940355	FALSE
CVLT_13	APOE_PICALM	172	sex	-0,24621	0,177533	-1,38686	0,16749	-0,59693	0,104502	0,148277	0,054255	1,577052	0,076455	0,61552474	FALSE
CVLT_13	APOE_PICALM	172	APOE	-0,36049	0,277868	-1,29734	0,196454	-0,90941	0,188436	0,148277	0,054255	1,577052	0,076455	0,6715999	FALSE
CVLT_13	APOE_PICALM	172	PICALM:Pla	-0,30501	0,248778	-1,22605	0,222052	-0,79647	0,186444	0,148277	0,054255	1,577052	0,076455	0,69257616	FALSE
CVLT_13	APOE_PICALM	172	APOE:age_	0,079947	0,064533	1,238855	0,217284	-0,04754	0,207431	0,148277	0,054255	1,577052	0,076455	0,69257616	FALSE
CVLT_13	APOE_PICALM	172	PICALM:ed	0,28472	0,296913	0,958933	0,339096	-0,30183	0,871268	0,148277	0,054255	1,577052	0,076455	0,80275452	FALSE
CVLT_13	APOE_PICALM	172	PICALM:Sta	0,245991	0,30801	0,798646	0,425726	-0,36248	0,85446	0,148277	0,054255	1,577052	0,076455	0,87170999	FALSE
CVLT_13	APOE_PICALM	172	education_	-0,14696	0,200743	-0,73206	0,465245	-0,54352	0,24961	0,148277	0,054255	1,577052	0,076455	0,87400586	FALSE
CVLT_13	APOE_PICALM	172	age_c	0,025757	0,039495	0,652171	0,515263	-0,05226	0,103779	0,148277	0,054255	1,577052	0,076455	0,89521185	FALSE
CVLT_13	APOE_PICALM	172	APOE:Plast	-0,19987	0,296846	-0,67331	0,501756	-0,78629	0,386545	0,148277	0,054255	1,577052	0,076455	0,89521185	FALSE
CVLT_13	APOE_PICALM	172	PICALM:ag	0,037987	0,057953	0,655486	0,513133	-0,0765	0,152473	0,148277	0,054255	1,577052	0,076455	0,89521185	FALSE
CVLT_13	APOE_PICALM	172	APOE:Stabi	-0,21231	0,332916	-0,63772	0,524606	-0,86998	0,445366	0,148277	0,054255	1,577052	0,076455	0,89687005	FALSE

CVLT_13	APOE_PICALM	172	PICALM	-0,12486	0,207924	-0,6005	0,549053	-0,53561	0,285892	0,148277	0,054255	1,577052	0,076455	0,89778791	FALSE
CVLT_13	APOE_PICALM	172	Stability	-0,10165	0,197112	-0,51567	0,606822	-0,49104	0,287747	0,148277	0,054255	1,577052	0,076455	0,90982023	FALSE
CVLT_13	APOE_PICALM	172	age_c:educ	0,00561	0,04499	0,124691	0,900931	-0,08327	0,094486	0,148277	0,054255	1,577052	0,076455	0,98158997	FALSE
CVLT_13	APOE_PICALM	172	Plasticity	0,016364	0,185823	0,088064	0,92994	-0,35073	0,383455	0,148277	0,054255	1,577052	0,076455	0,98995361	FALSE
CVLT_13	PICALM	172	Intercept	0,671411	0,151021	4,445819	1,63E-05	0,37316	0,969662	0,091098	0,028611	1,457873	0,152382	0,0001917	TRUE
CVLT_13	PICALM	172	education_	-0,3142	0,172537	-1,82104	0,070468	-0,65494	0,026546	0,091098	0,028611	1,457873	0,152382	0,39070826	FALSE
CVLT_13	PICALM	172	sex	-0,31091	0,177526	-1,75134	0,081803	-0,66151	0,039687	0,091098	0,028611	1,457873	0,152382	0,40761667	FALSE
CVLT_13	PICALM	172	PICALM:ed	0,418861	0,288362	1,452554	0,148306	-0,15063	0,988348	0,091098	0,028611	1,457873	0,152382	0,57750908	FALSE
CVLT_13	PICALM	172	PICALM:Pla	-0,32707	0,249363	-1,31161	0,19153	-0,81953	0,1654	0,091098	0,028611	1,457873	0,152382	0,65859474	FALSE
CVLT_13	PICALM	172	PICALM:ag	0,063006	0,057213	1,101238	0,272447	-0,04999	0,175997	0,091098	0,028611	1,457873	0,152382	0,73824396	FALSE
CVLT_13	PICALM	172	age_c	0,037624	0,036737	1,024162	0,307305	-0,03493	0,110175	0,091098	0,028611	1,457873	0,152382	0,78167367	FALSE
CVLT_13	PICALM	172	PICALM:Sta	0,241132	0,299299	0,805654	0,421638	-0,34996	0,832219	0,091098	0,028611	1,457873	0,152382	0,87170999	FALSE
CVLT_13	PICALM	172	Stability	-0,13593	0,184191	-0,73801	0,46159	-0,49969	0,227824	0,091098	0,028611	1,457873	0,152382	0,87396458	FALSE
CVLT_13	PICALM	172	PICALM	0,091129	0,177915	0,512204	0,609215	-0,26024	0,442495	0,091098	0,028611	1,457873	0,152382	0,90982023	FALSE
CVLT_13	PICALM	172	age_c:educ	0,021902	0,04471	0,489873	0,624895	-0,0664	0,110201	0,091098	0,028611	1,457873	0,152382	0,90982023	FALSE
CVLT_13	PICALM	172	Plasticity	0,002571	0,172419	0,014912	0,988121	-0,33794	0,343082	0,091098	0,028611	1,457873	0,152382	0,99488912	FALSE
CVLT_2	APOE	173	Intercept	8,890723	0,235717	37,7178	7,91E-82	8,425227	9,356218	0,150956	0,092947	2,602271	0,004487	2,1139E-80	TRUE
CVLT_2	APOE	173	sex	1,290856	0,297277	4,342272	2,49E-05	0,703792	1,877921	0,150956	0,092947	2,602271	0,004487	0,00028655	TRUE
CVLT_2	APOE	173	education_	0,525923	0,262438	2,003985	0,046749	0,007657	1,044188	0,150956	0,092947	2,602271	0,004487	0,2891053	FALSE
CVLT_2	APOE	173	age_c	-0,05397	0,054912	-0,98282	0,327173	-0,16241	0,054473	0,150956	0,092947	2,602271	0,004487	0,79824682	FALSE
CVLT_2	APOE	173	Plasticity	0,203077	0,239759	0,847003	0,398251	-0,2704	0,676555	0,150956	0,092947	2,602271	0,004487	0,86092433	FALSE
CVLT_2	APOE	173	Stability	0,199212	0,282305	0,705661	0,481419	-0,35829	0,75671	0,150956	0,092947	2,602271	0,004487	0,87971762	FALSE
CVLT_2	APOE	173	APOE:Stabi	-0,33701	0,538536	-0,62578	0,532345	-1,40051	0,726501	0,150956	0,092947	2,602271	0,004487	0,89687005	FALSE
CVLT_2	APOE	173	age_c:educ	0,046114	0,07373	0,625442	0,532567	-0,09949	0,191716	0,150956	0,092947	2,602271	0,004487	0,89687005	FALSE
CVLT_2	APOE	173	APOE:educ	0,106315	0,562171	0,189115	0,850241	-1,00387	1,216496	0,150956	0,092947	2,602271	0,004487	0,97621258	FALSE
CVLT_2	APOE	173	APOE	-0,0539	0,343105	-0,15708	0,875378	-0,73146	0,623671	0,150956	0,092947	2,602271	0,004487	0,98158997	FALSE
CVLT_2	APOE	173	APOE:Plast	0,064666	0,503752	0,128368	0,898018	-0,93015	1,059479	0,150956	0,092947	2,602271	0,004487	0,98158997	FALSE
CVLT_2	APOE	173	APOE:age_	-0,01757	0,108136	-0,16245	0,871152	-0,23111	0,19598	0,150956	0,092947	2,602271	0,004487	0,98158997	FALSE
CVLT_2	APOE_PICALM	173	Intercept	8,77496	0,273238	32,11471	2,16E-70	8,235209	9,314711	0,191534	0,102863	2,160063	0,007218	4,5432E-69	TRUE
CVLT_2	APOE_PICALM	173	sex	1,22576	0,301175	4,069923	7,47E-05	0,630822	1,820697	0,191534	0,102863	2,160063	0,007218	0,00081365	TRUE
CVLT_2	APOE_PICALM	173	APOE:PICA	-1,69052	0,714456	-2,36616	0,01921	-3,10184	-0,27919	0,191534	0,102863	2,160063	0,007218	0,14220499	FALSE
CVLT_2	APOE_PICALM	173	education_	0,593679	0,340365	1,744242	0,083099	-0,07867	1,266033	0,191534	0,102863	2,160063	0,007218	0,40761667	FALSE
CVLT_2	APOE_PICALM	173	APOE	0,804563	0,471688	1,70571	0,090065	-0,1272	1,73633	0,191534	0,102863	2,160063	0,007218	0,42343221	FALSE
CVLT_2	APOE_PICALM	173	Stability	0,420728	0,333976	1,259756	0,209651	-0,239	1,08046	0,191534	0,102863	2,160063	0,007218	0,68813815	FALSE
CVLT_2	APOE_PICALM	173	PICALM:Sta	-0,5284	0,516585	-1,02287	0,307962	-1,54886	0,492055	0,191534	0,102863	2,160063	0,007218	0,78167367	FALSE
CVLT_2	APOE_PICALM	173	PICALM	0,294833	0,352867	0,835536	0,404702	-0,40222	0,991882	0,191534	0,102863	2,160063	0,007218	0,86252891	FALSE
CVLT_2	APOE_PICALM	173	PICALM:Pla	0,348905	0,422289	0,826223	0,409947	-0,48528	1,183089	0,191534	0,102863	2,160063	0,007218	0,86252891	FALSE
CVLT_2	APOE_PICALM	173	age_c	-0,042	0,066944	-0,62738	0,531333	-0,17424	0,09024	0,191534	0,102863	2,160063	0,007218	0,89687005	FALSE
CVLT_2	APOE_PICALM	173	age_c:educ	0,040995	0,076334	0,537046	0,592006	-0,10979	0,191785	0,191534	0,102863	2,160063	0,007218	0,90982023	FALSE

CVLT_2	APOE_PICALM	173	APOE:educ	0,257929	0,587354	0,439137	0,661174	-0,90232	1,41818	0,191534	0,102863	2,160063	0,007218	0,91260662	FALSE
CVLT_2	APOE_PICALM	173	PICALM:ag	-0,02832	0,09769	-0,28987	0,772303	-0,22129	0,164659	0,191534	0,102863	2,160063	0,007218	0,95804687	FALSE
CVLT_2	APOE_PICALM	173	PICALM:ed	-0,14152	0,501894	-0,28197	0,778346	-1,13295	0,849918	0,191534	0,102863	2,160063	0,007218	0,9614867	FALSE
CVLT_2	APOE_PICALM	173	APOE:age_	-0,02091	0,108474	-0,19276	0,847396	-0,23519	0,193368	0,191534	0,102863	2,160063	0,007218	0,97621258	FALSE
CVLT_2	APOE_PICALM	173	APOE:Plast	0,057176	0,503901	0,113467	0,909807	-0,93822	1,052576	0,191534	0,102863	2,160063	0,007218	0,98158997	FALSE
CVLT_2	APOE_PICALM	173	Plasticity	0,028612	0,315341	0,090733	0,927822	-0,59431	0,651532	0,191534	0,102863	2,160063	0,007218	0,98995361	FALSE
CVLT_2	APOE_PICALM	173	APOE:Stabi	-0,04415	0,555716	-0,07945	0,936777	-1,14191	1,053603	0,191534	0,102863	2,160063	0,007218	0,99162101	FALSE
CVLT_2	PICALM	173	Intercept	8,934771	0,252627	35,36747	8,22E-78	8,435882	9,433661	0,161726	0,104453	2,823755	0,002129	2,015E-76	TRUE
CVLT_2	PICALM	173	sex	1,265971	0,296789	4,265563	3,39E-05	0,67987	1,852072	0,161726	0,104453	2,823755	0,002129	0,00037614	TRUE
CVLT_2	PICALM	173	education_	0,636274	0,288735	2,203663	0,028968	0,066078	1,20647	0,161726	0,104453	2,823755	0,002129	0,20454907	FALSE
CVLT_2	PICALM	173	PICALM:St	-0,55935	0,49638	-1,12686	0,261476	-1,53961	0,420902	0,161726	0,104453	2,823755	0,002129	0,72120934	FALSE
CVLT_2	PICALM	173	Stability	0,334866	0,308242	1,086375	0,278937	-0,27385	0,943584	0,161726	0,104453	2,823755	0,002129	0,74237363	FALSE
CVLT_2	PICALM	173	PICALM:Pl	0,395627	0,417307	0,948049	0,344525	-0,42847	1,219727	0,161726	0,104453	2,823755	0,002129	0,80709548	FALSE
CVLT_2	PICALM	173	age_c	-0,041	0,061478	-0,66682	0,505839	-0,1624	0,080413	0,161726	0,104453	2,823755	0,002129	0,89521185	FALSE
CVLT_2	PICALM	173	PICALM:ag	-0,06267	0,095445	-0,65666	0,512336	-0,25116	0,12581	0,161726	0,104453	2,823755	0,002129	0,89521185	FALSE
CVLT_2	PICALM	173	PICALM	-0,13576	0,296886	-0,45729	0,648078	-0,72206	0,450529	0,161726	0,104453	2,823755	0,002129	0,90982023	FALSE
CVLT_2	PICALM	173	age_c:educ	0,034551	0,074752	0,462214	0,644552	-0,11307	0,182172	0,161726	0,104453	2,823755	0,002129	0,90982023	FALSE
CVLT_2	PICALM	173	PICALM:ed	-0,20011	0,481515	-0,41559	0,678262	-1,15101	0,750787	0,161726	0,104453	2,823755	0,002129	0,91438395	FALSE
CVLT_2	PICALM	173	Plasticity	0,044387	0,28854	0,153834	0,877933	-0,52542	0,614199	0,161726	0,104453	2,823755	0,002129	0,98158997	FALSE
CVLT_3	APOE	173	Intercept	13,20701	0,238901	55,28228	1,3E-106	12,73522	13,67879	0,136172	0,077153	2,307252	0,011861	7,525E-105	TRUE
CVLT_3	APOE	173	sex	1,203513	0,301293	3,994498	9,84E-05	0,608518	1,798508	0,136172	0,077153	2,307252	0,011861	0,00105197	TRUE
CVLT_3	APOE	173	Plasticity	0,402576	0,242998	1,656703	0,099527	-0,0773	0,882451	0,136172	0,077153	2,307252	0,011861	0,45365823	FALSE
CVLT_3	APOE	173	APOE	0,512307	0,34774	1,473246	0,142637	-0,17441	1,199027	0,136172	0,077153	2,307252	0,011861	0,5628904	FALSE
CVLT_3	APOE	173	APOE:Stabi	0,531312	0,545812	0,973434	0,331798	-0,54656	1,609185	0,136172	0,077153	2,307252	0,011861	0,80275452	FALSE
CVLT_3	APOE	173	age_c	-0,04896	0,055654	-0,87971	0,38033	-0,15887	0,060947	0,136172	0,077153	2,307252	0,011861	0,84390171	FALSE
CVLT_3	APOE	173	APOE:age_	-0,09048	0,109596	-0,82554	0,410285	-0,30691	0,125955	0,136172	0,077153	2,307252	0,011861	0,86252891	FALSE
CVLT_3	APOE	173	APOE:educ	0,402195	0,569766	0,705895	0,481273	-0,72298	1,527374	0,136172	0,077153	2,307252	0,011861	0,87971762	FALSE
CVLT_3	APOE	173	Stability	-0,15989	0,286119	-0,55881	0,577069	-0,72492	0,405144	0,136172	0,077153	2,307252	0,011861	0,90166392	FALSE
CVLT_3	APOE	173	APOE:Plast	-0,28453	0,510557	-0,55728	0,578108	-1,29278	0,723727	0,136172	0,077153	2,307252	0,011861	0,90166392	FALSE
CVLT_3	APOE	173	age_c:educ	0,018223	0,074726	0,24387	0,807642	-0,12935	0,165793	0,136172	0,077153	2,307252	0,011861	0,97514073	FALSE
CVLT_3	APOE	173	education_	0,018534	0,265984	0,069679	0,944535	-0,50673	0,5438	0,136172	0,077153	2,307252	0,011861	0,99162101	FALSE
CVLT_3	APOE_PICALM	173	Intercept	13,10274	0,27818	47,10164	9,5E-94	12,55323	13,65225	0,17001	0,078979	1,867601	0,02463	3,103E-92	TRUE
CVLT_3	APOE_PICALM	173	sex	1,137756	0,306622	3,710607	0,000288	0,532057	1,743454	0,17001	0,078979	1,867601	0,02463	0,00291663	TRUE
CVLT_3	APOE_PICALM	173	APOE	1,044161	0,48022	2,17434	0,031196	0,095541	1,992781	0,17001	0,078979	1,867601	0,02463	0,21329592	FALSE
CVLT_3	APOE_PICALM	173	APOE:PICA	-1,04394	0,727379	-1,43521	0,153242	-2,4808	0,392913	0,17001	0,078979	1,867601	0,02463	0,58510582	FALSE
CVLT_3	APOE_PICALM	173	PICALM:Pl	0,602024	0,429927	1,400295	0,163424	-0,24725	1,451297	0,17001	0,078979	1,867601	0,02463	0,6120581	FALSE
CVLT_3	APOE_PICALM	173	PICALM:ag	-0,13704	0,099457	-1,37787	0,170228	-0,33351	0,059427	0,17001	0,078979	1,867601	0,02463	0,61980636	FALSE
CVLT_3	APOE_PICALM	173	APOE:Stabi	0,691025	0,565768	1,221393	0,223792	-0,42659	1,808635	0,17001	0,078979	1,867601	0,02463	0,69257616	FALSE
CVLT_3	APOE_PICALM	173	APOE:educ	0,555313	0,597978	0,928652	0,354514	-0,62592	1,73655	0,17001	0,078979	1,867601	0,02463	0,81110511	FALSE

CVLT_3	APOE_PICALM	173	APOE:age_	-0,08515	0,110436	-0,77107	0,441838	-0,30331	0,133	0,17001	0,078979	1,867601	0,02463	0,87396458	FALSE
CVLT_3	APOE_PICALM	173	PICALM	0,218179	0,359249	0,607318	0,544528	-0,49148	0,927835	0,17001	0,078979	1,867601	0,02463	0,89687005	FALSE
CVLT_3	APOE_PICALM	173	PICALM:Sta	-0,27205	0,525929	-0,51728	0,605696	-1,31097	0,766859	0,17001	0,078979	1,867601	0,02463	0,90982023	FALSE
CVLT_3	APOE_PICALM	173	APOE:Plast	-0,27289	0,513015	-0,53194	0,595532	-1,28629	0,740513	0,17001	0,078979	1,867601	0,02463	0,90982023	FALSE
CVLT_3	APOE_PICALM	173	age_c:educ	0,035429	0,077715	0,455888	0,649108	-0,11809	0,188946	0,17001	0,078979	1,867601	0,02463	0,90982023	FALSE
CVLT_3	APOE_PICALM	173	Plasticity	0,132079	0,321044	0,411403	0,681345	-0,50211	0,766266	0,17001	0,078979	1,867601	0,02463	0,91438395	FALSE
CVLT_3	APOE_PICALM	173	PICALM:ed	0,209285	0,510972	0,409582	0,682678	-0,80008	1,218652	0,17001	0,078979	1,867601	0,02463	0,91438395	FALSE
CVLT_3	APOE_PICALM	173	education_	-0,06918	0,346521	-0,19965	0,842015	-0,7537	0,615331	0,17001	0,078979	1,867601	0,02463	0,97621258	FALSE
CVLT_3	APOE_PICALM	173	Stability	-0,05188	0,340017	-0,15257	0,878938	-0,72354	0,619789	0,17001	0,078979	1,867601	0,02463	0,98158997	FALSE
CVLT_3	APOE_PICALM	173	age_c	0,003273	0,068154	0,04802	0,961762	-0,13136	0,137904	0,17001	0,078979	1,867601	0,02463	0,99162101	FALSE
CVLT_3	PICALM	173	Intercept	13,26344	0,258433	51,32255	1,1E-101	12,75308	13,77379	0,131112	0,071747	2,208565	0,016305	4,805E-100	TRUE
CVLT_3	PICALM	173	sex	1,206708	0,30361	3,974533	0,000106	0,607136	1,806279	0,131112	0,071747	2,208565	0,016305	0,0011153	TRUE
CVLT_3	PICALM	173	PICALM:ag	-0,13607	0,097638	-1,39361	0,165356	-0,32889	0,056747	0,131112	0,071747	2,208565	0,016305	0,61365085	FALSE
CVLT_3	PICALM	173	PICALM:Pla	0,551888	0,426897	1,292788	0,197937	-0,29115	1,394928	0,131112	0,071747	2,208565	0,016305	0,67275567	FALSE
CVLT_3	PICALM	173	age_c:educ	0,034712	0,07647	0,453928	0,650492	-0,1163	0,185725	0,131112	0,071747	2,208565	0,016305	0,90982023	FALSE
CVLT_3	PICALM	173	PICALM:ed	0,146548	0,492582	0,29751	0,766461	-0,82621	1,119303	0,131112	0,071747	2,208565	0,016305	0,95482858	FALSE
CVLT_3	PICALM	173	education_	0,074675	0,295371	0,252819	0,80073	-0,50863	0,657976	0,131112	0,071747	2,208565	0,016305	0,97078241	FALSE
CVLT_3	PICALM	173	PICALM	0,055784	0,303709	0,183674	0,8545	-0,54398	0,655551	0,131112	0,071747	2,208565	0,016305	0,97621258	FALSE
CVLT_3	PICALM	173	Plasticity	0,049767	0,295172	0,168603	0,86632	-0,53314	0,632675	0,131112	0,071747	2,208565	0,016305	0,98158997	FALSE
CVLT_3	PICALM	173	age_c	-0,00755	0,062891	-0,12005	0,904595	-0,13175	0,116648	0,131112	0,071747	2,208565	0,016305	0,98158997	FALSE
CVLT_3	PICALM	173	Stability	0,012112	0,315326	0,03841	0,969409	-0,6106	0,63482	0,131112	0,071747	2,208565	0,016305	0,99162101	FALSE
CVLT_3	PICALM	173	PICALM:Sta	0,005094	0,507788	0,010032	0,992009	-0,99769	1,007878	0,131112	0,071747	2,208565	0,016305	0,99709578	FALSE
CVLT_4	APOE	173	Intercept	7,873553	0,238398	33,02698	1,34E-73	7,402763	8,344342	0,078535	0,015577	1,24743	0,260017	3,0214E-72	TRUE
CVLT_4	APOE	173	sex	0,88661	0,300658	2,948902	0,003664	0,292869	1,480351	0,078535	0,015577	1,24743	0,260017	0,03215739	TRUE
CVLT_4	APOE	173	age_c:educ	0,093813	0,074568	1,258077	0,210186	-0,05345	0,241071	0,078535	0,015577	1,24743	0,260017	0,68813815	FALSE
CVLT_4	APOE	173	Stability	0,274659	0,285516	0,961975	0,337505	-0,28918	0,838497	0,078535	0,015577	1,24743	0,260017	0,80275452	FALSE
CVLT_4	APOE	173	Plasticity	-0,19322	0,242486	-0,79683	0,426725	-0,67208	0,285644	0,078535	0,015577	1,24743	0,260017	0,87170999	FALSE
CVLT_4	APOE	173	APOE:Stabi	-0,33613	0,544661	-0,61714	0,538012	-1,41174	0,739467	0,078535	0,015577	1,24743	0,260017	0,89687005	FALSE
CVLT_4	APOE	173	education_	0,124775	0,265423	0,4701	0,638919	-0,39938	0,648935	0,078535	0,015577	1,24743	0,260017	0,90982023	FALSE
CVLT_4	APOE	173	APOE:educ	0,305285	0,568565	0,53694	0,59205	-0,81752	1,428092	0,078535	0,015577	1,24743	0,260017	0,90982023	FALSE
CVLT_4	APOE	173	APOE:age_	-0,03794	0,109365	-0,34689	0,729124	-0,25391	0,178038	0,078535	0,015577	1,24743	0,260017	0,94432864	FALSE
CVLT_4	APOE	173	APOE:Plast	-0,10544	0,509481	-0,20696	0,8363	-1,11157	0,900683	0,078535	0,015577	1,24743	0,260017	0,97621258	FALSE
CVLT_4	APOE	173	APOE	0,041668	0,347007	0,120078	0,904571	-0,6436	0,72694	0,078535	0,015577	1,24743	0,260017	0,98158997	FALSE
CVLT_4	APOE	173	age_c	-0,00828	0,055537	-0,14911	0,88165	-0,11796	0,101393	0,078535	0,015577	1,24743	0,260017	0,98158997	FALSE
CVLT_4	APOE_PICALM	173	Intercept	7,710329	0,278812	27,65424	8,16E-62	7,159568	8,26109	0,106842	0,008883	1,090681	0,367397	1,5991E-60	TRUE
CVLT_4	APOE_PICALM	173	sex	0,807435	0,307319	2,627352	0,00947	0,200361	1,414508	0,106842	0,008883	1,090681	0,367397	0,07733649	FALSE
CVLT_4	APOE_PICALM	173	Stability	0,520507	0,340789	1,52736	0,12871	-0,15268	1,193697	0,106842	0,008883	1,090681	0,367397	0,52923919	FALSE
CVLT_4	APOE_PICALM	173	PICALM:Sta	-0,7236	0,527123	-1,37274	0,171817	-1,76487	0,317671	0,106842	0,008883	1,090681	0,367397	0,61980636	FALSE
CVLT_4	APOE_PICALM	173	PICALM:ag	-0,11921	0,099683	-1,1959	0,233563	-0,31612	0,077702	0,106842	0,008883	1,090681	0,367397	0,6975188	FALSE

CVLT_4	APOE_PICALM	173	PICALM	0,358055	0,360065	0,994417	0,32157	-0,35321	1,069323	0,106842	0,008883	1,090681	0,367397	0,79114198	FALSE
CVLT_4	APOE_PICALM	173	age_c:educ	0,076582	0,077891	0,983187	0,327048	-0,07728	0,230447	0,106842	0,008883	1,090681	0,367397	0,79824682	FALSE
CVLT_4	APOE_PICALM	173	APOE	0,29871	0,48131	0,620618	0,535763	-0,65206	1,249484	0,106842	0,008883	1,090681	0,367397	0,89687005	FALSE
CVLT_4	APOE_PICALM	173	Plasticity	-0,19764	0,321773	-0,61424	0,539961	-0,83327	0,437983	0,106842	0,008883	1,090681	0,367397	0,89687005	FALSE
CVLT_4	APOE_PICALM	173	age_c	0,042146	0,068309	0,616982	0,538152	-0,09279	0,177083	0,106842	0,008883	1,090681	0,367397	0,89687005	FALSE
CVLT_4	APOE_PICALM	173	APOE:educ	0,356065	0,599336	0,5941	0,553312	-0,82785	1,539985	0,106842	0,008883	1,090681	0,367397	0,89894588	FALSE
CVLT_4	APOE_PICALM	173	APOE:PICA	-0,30484	0,72903	-0,41815	0,676419	-1,74496	1,135276	0,106842	0,008883	1,090681	0,367397	0,91438395	FALSE
CVLT_4	APOE_PICALM	173	APOE:Stabi	-0,23794	0,567052	-0,41961	0,675349	-1,35809	0,882206	0,106842	0,008883	1,090681	0,367397	0,91438395	FALSE
CVLT_4	APOE_PICALM	173	APOE:Plast	-0,16317	0,51418	-0,31734	0,751412	-1,17888	0,852534	0,106842	0,008883	1,090681	0,367397	0,95185346	FALSE
CVLT_4	APOE_PICALM	173	APOE:age_	-0,03583	0,110687	-0,32369	0,746608	-0,25448	0,182821	0,106842	0,008883	1,090681	0,367397	0,95185346	FALSE
CVLT_4	APOE_PICALM	173	education_	0,10508	0,347308	0,302557	0,762633	-0,58099	0,791149	0,106842	0,008883	1,090681	0,367397	0,95347316	FALSE
CVLT_4	APOE_PICALM	173	PICALM:Pla	0,06096	0,430903	0,141471	0,887681	-0,79024	0,912161	0,106842	0,008883	1,090681	0,367397	0,98158997	FALSE
CVLT_4	APOE_PICALM	173	PICALM:ed	0,086162	0,512132	0,168242	0,866612	-0,9255	1,097821	0,106842	0,008883	1,090681	0,367397	0,98158997	FALSE
CVLT_4	PICALM	173	Intercept	7,770836	0,254183	30,57177	6,14E-69	7,268873	8,2728	0,099575	0,038055	1,618586	0,097891	1,2454E-67	TRUE
CVLT_4	PICALM	173	sex	0,830228	0,298617	2,780238	0,00608	0,240515	1,41994	0,099575	0,038055	1,618586	0,097891	0,051808	FALSE
CVLT_4	PICALM	173	Stability	0,446582	0,310141	1,439933	0,151828	-0,16589	1,059051	0,099575	0,038055	1,618586	0,097891	0,58349532	FALSE
CVLT_4	PICALM	173	PICALM:Sta	-0,7002	0,499438	-1,40197	0,162849	-1,68649	0,286097	0,099575	0,038055	1,618586	0,097891	0,6120581	FALSE
CVLT_4	PICALM	173	PICALM:ag	-0,12259	0,096033	-1,27658	0,203588	-0,31224	0,067053	0,099575	0,038055	1,618586	0,097891	0,68405654	FALSE
CVLT_4	PICALM	173	PICALM	0,274792	0,298715	0,919914	0,358994	-0,31511	0,864697	0,099575	0,038055	1,618586	0,097891	0,81501357	FALSE
CVLT_4	PICALM	173	Plasticity	-0,26314	0,290318	-0,90639	0,366085	-0,83646	0,310182	0,099575	0,038055	1,618586	0,097891	0,82474404	FALSE
CVLT_4	PICALM	173	age_c:educ	0,068367	0,075212	0,90899	0,364714	-0,08016	0,216897	0,099575	0,038055	1,618586	0,097891	0,82474404	FALSE
CVLT_4	PICALM	173	education_	0,211197	0,290514	0,726979	0,468295	-0,36251	0,784907	0,099575	0,038055	1,618586	0,097891	0,87447583	FALSE
CVLT_4	PICALM	173	age_c	0,035725	0,061857	0,577539	0,564382	-0,08643	0,157881	0,099575	0,038055	1,618586	0,097891	0,89894588	FALSE
CVLT_4	PICALM	173	PICALM:Pla	0,095458	0,419878	0,227347	0,820442	-0,73372	0,924636	0,099575	0,038055	1,618586	0,097891	0,9760277	FALSE
CVLT_4	PICALM	173	PICALM:ed	0,002939	0,484482	0,006067	0,995167	-0,95382	0,959699	0,099575	0,038055	1,618586	0,097891	0,99739738	FALSE
CVLT_5	APOE	173	Intercept	11,69344	0,303598	38,51615	3,79E-83	11,09389	12,29299	0,200857	0,146257	3,678717	0,000112	1,0617E-81	TRUE
CVLT_5	APOE	173	sex	2,010131	0,382886	5,249944	4,75E-07	1,254004	2,766258	0,200857	0,146257	3,678717	0,000112	6,9791E-06	TRUE
CVLT_5	APOE	173	APOE:Stabi	1,02451	0,693624	1,47704	0,141618	-0,34526	2,394284	0,200857	0,146257	3,678717	0,000112	0,56264536	FALSE
CVLT_5	APOE	173	APOE:age_	-0,15629	0,139276	-1,12214	0,263474	-0,43133	0,118757	0,200857	0,146257	3,678717	0,000112	0,72120934	FALSE
CVLT_5	APOE	173	age_c	-0,06618	0,070726	-0,93568	0,350837	-0,20585	0,073493	0,200857	0,146257	3,678717	0,000112	0,81110511	FALSE
CVLT_5	APOE	173	APOE:educ	0,61674	0,724065	0,851775	0,395604	-0,81315	2,04663	0,200857	0,146257	3,678717	0,000112	0,85835942	FALSE
CVLT_5	APOE	173	education_	0,278965	0,338015	0,825303	0,410421	-0,38855	0,94648	0,200857	0,146257	3,678717	0,000112	0,86252891	FALSE
CVLT_5	APOE	173	APOE:Plast	-0,51596	0,648822	-0,79523	0,427651	-1,79726	0,765336	0,200857	0,146257	3,678717	0,000112	0,87170999	FALSE
CVLT_5	APOE	173	Plasticity	0,23752	0,308805	0,769158	0,442927	-0,37231	0,84735	0,200857	0,146257	3,678717	0,000112	0,87396458	FALSE
CVLT_5	APOE	173	APOE	0,296922	0,441912	0,671903	0,502608	-0,57577	1,169614	0,200857	0,146257	3,678717	0,000112	0,89521185	FALSE
CVLT_5	APOE	173	age_c:educ	-0,01128	0,094962	-0,11879	0,905592	-0,19881	0,176252	0,200857	0,146257	3,678717	0,000112	0,98158997	FALSE
CVLT_5	APOE	173	Stability	-0,02505	0,363603	-0,0689	0,945158	-0,7431	0,692996	0,200857	0,146257	3,678717	0,000112	0,99162101	FALSE
CVLT_5	APOE_PICALM	173	Intercept	11,62481	0,358039	32,46802	4,94E-71	10,91755	12,33208	0,212381	0,125997	2,458565	0,001941	1,0748E-69	TRUE
CVLT_5	APOE_PICALM	173	sex	2,005615	0,394646	5,082056	1,06E-06	1,226035	2,785194	0,212381	0,125997	2,458565	0,001941	1,5231E-05	TRUE

CVLT_5	APOE_PICALM	173	APOE:Stabi	1,159098	0,728186	1,591761	0,113476	-0,27935	2,597547	0,212381	0,125997	2,458565	0,001941	0,48703463	FALSE
CVLT_5	APOE_PICALM	173	APOE	0,652853	0,618079	1,056262	0,292492	-0,56809	1,873799	0,212381	0,125997	2,458565	0,001941	0,76435261	FALSE
CVLT_5	APOE_PICALM	173	APOE:age_	-0,14972	0,142139	-1,05334	0,293825	-0,4305	0,13106	0,212381	0,125997	2,458565	0,001941	0,76435261	FALSE
CVLT_5	APOE_PICALM	173	PICALM:Pla	0,495393	0,553349	0,895264	0,372034	-0,59768	1,588471	0,212381	0,125997	2,458565	0,001941	0,83177196	FALSE
CVLT_5	APOE_PICALM	173	education_	0,367195	0,445999	0,823308	0,411598	-0,51383	1,248217	0,212381	0,125997	2,458565	0,001941	0,86252891	FALSE
CVLT_5	APOE_PICALM	173	APOE:educ	0,608	0,769642	0,789978	0,430748	-0,91234	2,128342	0,212381	0,125997	2,458565	0,001941	0,87170999	FALSE
CVLT_5	APOE_PICALM	173	APOE:PICA	-0,70237	0,936191	-0,75024	0,454246	-2,55171	1,146969	0,212381	0,125997	2,458565	0,001941	0,87396458	FALSE
CVLT_5	APOE_PICALM	173	APOE:Plast	-0,50033	0,660289	-0,75774	0,449758	-1,80465	0,804001	0,212381	0,125997	2,458565	0,001941	0,87396458	FALSE
CVLT_5	APOE_PICALM	173	PICALM:ag	-0,09447	0,128009	-0,738	0,46163	-0,34734	0,158397	0,212381	0,125997	2,458565	0,001941	0,87396458	FALSE
CVLT_5	APOE_PICALM	173	age_c	-0,02951	0,08772	-0,33637	0,737046	-0,20279	0,143774	0,212381	0,125997	2,458565	0,001941	0,94625118	FALSE
CVLT_5	APOE_PICALM	173	PICALM:ed	-0,20614	0,657659	-0,31345	0,75436	-1,50528	1,092989	0,212381	0,125997	2,458565	0,001941	0,95185346	FALSE
CVLT_5	APOE_PICALM	173	PICALM	0,106786	0,462381	0,230949	0,817659	-0,8066	1,020168	0,212381	0,125997	2,458565	0,001941	0,9760277	FALSE
CVLT_5	APOE_PICALM	173	Stability	-0,01575	0,437627	-0,036	0,971328	-0,88024	0,848728	0,212381	0,125997	2,458565	0,001941	0,99162101	FALSE
CVLT_5	APOE_PICALM	173	PICALM:Sta	0,028589	0,67691	0,042235	0,966366	-1,30857	1,365749	0,212381	0,125997	2,458565	0,001941	0,99162101	FALSE
CVLT_5	APOE_PICALM	173	age_c:educ	-0,00418	0,100025	-0,04179	0,966721	-0,20177	0,193408	0,212381	0,125997	2,458565	0,001941	0,99162101	FALSE
CVLT_5	APOE_PICALM	173	Plasticity	0,00955	0,413208	0,023112	0,98159	-0,8067	0,825797	0,212381	0,125997	2,458565	0,001941	0,99473515	FALSE
CVLT_5	PICALM	173	Intercept	11,67929	0,330926	35,29279	1,11E-77	11,02578	12,33281	0,18386	0,128099	3,297278	0,00042	2,6156E-76	TRUE
CVLT_5	PICALM	173	sex	2,061294	0,388775	5,302019	3,73E-07	1,293538	2,829051	0,18386	0,128099	3,297278	0,00042	5,6191E-06	TRUE
CVLT_5	PICALM	173	education_	0,553689	0,378225	1,463914	0,145167	-0,19323	1,300611	0,18386	0,128099	3,297278	0,00042	0,56905506	FALSE
CVLT_5	PICALM	173	PICALM:Pla	0,439294	0,546646	0,803617	0,422803	-0,64023	1,518816	0,18386	0,128099	3,297278	0,00042	0,87170999	FALSE
CVLT_5	PICALM	173	PICALM:ag	-0,09186	0,125027	-0,73471	0,463585	-0,33876	0,155046	0,18386	0,128099	3,297278	0,00042	0,87396458	FALSE
CVLT_5	PICALM	173	age_c	-0,05759	0,080533	-0,7151	0,475581	-0,21663	0,101448	0,18386	0,128099	3,297278	0,00042	0,87971762	FALSE
CVLT_5	PICALM	173	Stability	0,200358	0,403778	0,496208	0,620425	-0,59703	0,997742	0,18386	0,128099	3,297278	0,00042	0,90982023	FALSE
CVLT_5	PICALM	173	PICALM:Sta	0,323056	0,650227	0,496836	0,619982	-0,96102	1,60713	0,18386	0,128099	3,297278	0,00042	0,90982023	FALSE
CVLT_5	PICALM	173	PICALM:ed	-0,29614	0,630756	-0,46951	0,639343	-1,54177	0,949478	0,18386	0,128099	3,297278	0,00042	0,90982023	FALSE
CVLT_5	PICALM	173	Plasticity	-0,14368	0,37797	-0,38013	0,704349	-0,8901	0,602741	0,18386	0,128099	3,297278	0,00042	0,93069081	FALSE
CVLT_5	PICALM	173	PICALM	0,053727	0,388903	0,138151	0,890294	-0,71428	0,821735	0,18386	0,128099	3,297278	0,00042	0,98158997	FALSE
CVLT_5	PICALM	173	age_c:educ	-0,01356	0,09792	-0,13846	0,890051	-0,20693	0,179816	0,18386	0,128099	3,297278	0,00042	0,98158997	FALSE
CVLT_6	APOE	173	Intercept	12,90346	0,223777	57,66222	2E-109	12,46155	13,34538	0,138642	0,079792	2,355834	0,010127	1,679E-107	TRUE
CVLT_6	APOE	173	sex	1,034024	0,282218	3,663917	0,000337	0,476698	1,591351	0,138642	0,079792	2,355834	0,010127	0,00324579	TRUE
CVLT_6	APOE	173	APOE:age_	-0,18183	0,102658	-1,77125	0,07841	-0,38456	0,020896	0,138642	0,079792	2,355834	0,010127	0,40091593	FALSE
CVLT_6	APOE	173	APOE:Stabi	0,675704	0,511257	1,321652	0,188159	-0,33393	1,685339	0,138642	0,079792	2,355834	0,010127	0,65080859	FALSE
CVLT_6	APOE	173	Plasticity	0,174731	0,227614	0,767665	0,443811	-0,27476	0,624226	0,138642	0,079792	2,355834	0,010127	0,87396458	FALSE
CVLT_6	APOE	173	age_c	-0,03776	0,052131	-0,72426	0,469956	-0,1407	0,065192	0,138642	0,079792	2,355834	0,010127	0,87447583	FALSE
CVLT_6	APOE	173	APOE	0,222605	0,325725	0,683413	0,495328	-0,42064	0,86585	0,138642	0,079792	2,355834	0,010127	0,89068149	FALSE
CVLT_6	APOE	173	education_	0,139928	0,249145	0,561634	0,575146	-0,35208	0,631941	0,138642	0,079792	2,355834	0,010127	0,90166392	FALSE
CVLT_6	APOE	173	APOE:educ	0,292965	0,533695	0,548937	0,583809	-0,76098	1,34691	0,138642	0,079792	2,355834	0,010127	0,90557047	FALSE
CVLT_6	APOE	173	APOE:Plast	0,139543	0,478234	0,291788	0,770824	-0,80488	1,083964	0,138642	0,079792	2,355834	0,010127	0,95804687	FALSE
CVLT_6	APOE	173	Stability	-0,06051	0,268005	-0,22578	0,821656	-0,58977	0,468747	0,138642	0,079792	2,355834	0,010127	0,9760277	FALSE

CVLT_6	APOE	173	age_c:educ	0,000378	0,069995	0,005396	0,995701	-0,13785	0,138604	0,138642	0,079792	2,355834	0,010127	0,99739738	FALSE
CVLT_6	APOE_PICALM	173	Intercept	12,82107	0,262035	48,92886	3,77E-96	12,30345	13,33869	0,163043	0,071248	1,776161	0,03556	1,4771E-94	TRUE
CVLT_6	APOE_PICALM	173	sex	1,002077	0,288827	3,469476	0,000675	0,431533	1,572622	0,163043	0,071248	1,776161	0,03556	0,00630414	TRUE
CVLT_6	APOE_PICALM	173	APOE:Stabi	0,92497	0,532931	1,735626	0,084617	-0,12778	1,977716	0,163043	0,071248	1,776161	0,03556	0,41119863	FALSE
CVLT_6	APOE_PICALM	173	APOE:age_	-0,17843	0,104026	-1,71521	0,088306	-0,38392	0,027066	0,163043	0,071248	1,776161	0,03556	0,42214563	FALSE
CVLT_6	APOE_PICALM	173	APOE	0,744633	0,452349	1,646147	0,10176	-0,14893	1,638196	0,163043	0,071248	1,776161	0,03556	0,46026642	FALSE
CVLT_6	APOE_PICALM	173	APOE:PICA	-0,92179	0,685163	-1,34536	0,180473	-2,27525	0,431669	0,163043	0,071248	1,776161	0,03556	0,63852151	FALSE
CVLT_6	APOE_PICALM	173	PICALM:Sta	-0,50387	0,495405	-1,0171	0,310693	-1,48249	0,474742	0,163043	0,071248	1,776161	0,03556	0,78167367	FALSE
CVLT_6	APOE_PICALM	173	PICALM:Pla	0,386377	0,404975	0,954076	0,341531	-0,41361	1,186359	0,163043	0,071248	1,776161	0,03556	0,80327985	FALSE
CVLT_6	APOE_PICALM	173	education_	0,303812	0,32641	0,930769	0,353421	-0,34097	0,948598	0,163043	0,071248	1,776161	0,03556	0,81110511	FALSE
CVLT_6	APOE_PICALM	173	PICALM:ed	-0,35818	0,481316	-0,74417	0,457902	-1,30897	0,592605	0,163043	0,071248	1,776161	0,03556	0,87396458	FALSE
CVLT_6	APOE_PICALM	173	PICALM	0,167801	0,338399	0,495868	0,62069	-0,50067	0,83627	0,163043	0,071248	1,776161	0,03556	0,90982023	FALSE
CVLT_6	APOE_PICALM	173	APOE:educ	0,287664	0,563272	0,510702	0,610286	-0,82502	1,400345	0,163043	0,071248	1,776161	0,03556	0,90982023	FALSE
CVLT_6	APOE_PICALM	173	Stability	0,14199	0,320283	0,443327	0,658148	-0,49069	0,774672	0,163043	0,071248	1,776161	0,03556	0,91260662	FALSE
CVLT_6	APOE_PICALM	173	age_c	-0,02711	0,064199	-0,42231	0,673384	-0,15393	0,099706	0,163043	0,071248	1,776161	0,03556	0,91438395	FALSE
CVLT_6	APOE_PICALM	173	PICALM:ag	-0,03314	0,093685	-0,35377	0,723994	-0,21821	0,151921	0,163043	0,071248	1,776161	0,03556	0,94183246	FALSE
CVLT_6	APOE_PICALM	173	APOE:Plast	0,115062	0,483241	0,238105	0,812114	-0,83953	1,069649	0,163043	0,071248	1,776161	0,03556	0,97519255	FALSE
CVLT_6	APOE_PICALM	173	age_c:educ	-0,01324	0,073204	-0,18091	0,856676	-0,15785	0,131364	0,163043	0,071248	1,776161	0,03556	0,97621258	FALSE
CVLT_6	APOE_PICALM	173	Plasticity	-0,00952	0,302412	-0,03147	0,974936	-0,6069	0,587863	0,163043	0,071248	1,776161	0,03556	0,99162101	FALSE
CVLT_6	PICALM	173	Intercept	12,90582	0,244454	52,7945	1,4E-103	12,42307	13,38857	0,116461	0,056095	1,929256	0,039142	7,715E-102	TRUE
CVLT_6	PICALM	173	sex	1,058084	0,287187	3,684301	0,000313	0,490944	1,625223	0,116461	0,056095	1,929256	0,039142	0,00306573	TRUE
CVLT_6	PICALM	173	education_	0,337267	0,279394	1,20714	0,229149	-0,21448	0,889016	0,116461	0,056095	1,929256	0,039142	0,6975188	FALSE
CVLT_6	PICALM	173	age_c	-0,06187	0,059489	-1,03997	0,299913	-0,17935	0,055613	0,116461	0,056095	1,929256	0,039142	0,77008313	FALSE
CVLT_6	PICALM	173	Stability	0,28696	0,298269	0,962082	0,337451	-0,30207	0,875984	0,116461	0,056095	1,929256	0,039142	0,80275452	FALSE
CVLT_6	PICALM	173	PICALM:Pla	0,328711	0,403806	0,814032	0,416829	-0,46873	1,12615	0,116461	0,056095	1,929256	0,039142	0,86606185	FALSE
CVLT_6	PICALM	173	PICALM:ed	-0,35579	0,465937	-0,7636	0,446226	-1,27592	0,564349	0,116461	0,056095	1,929256	0,039142	0,87396458	FALSE
CVLT_6	PICALM	173	PICALM:Sta	-0,30918	0,480321	-0,6437	0,520684	-1,25772	0,639358	0,116461	0,056095	1,929256	0,039142	0,89521185	FALSE
CVLT_6	PICALM	173	PICALM:ag	-0,04461	0,092357	-0,48304	0,629723	-0,227	0,137775	0,116461	0,056095	1,929256	0,039142	0,90982023	FALSE
CVLT_6	PICALM	173	age_c:educ	-0,0155	0,072333	-0,2143	0,830587	-0,15835	0,127344	0,116461	0,056095	1,929256	0,039142	0,97621258	FALSE
CVLT_6	PICALM	173	PICALM	0,01038	0,287281	0,036132	0,971222	-0,55694	0,577705	0,116461	0,056095	1,929256	0,039142	0,99162101	FALSE
CVLT_6	PICALM	173	Plasticity	0,016253	0,279205	0,05821	0,953653	-0,53512	0,56763	0,116461	0,056095	1,929256	0,039142	0,99162101	FALSE
CVLT_7	APOE	173	Intercept	12,58174	0,277708	45,30561	1,52E-93	12,03331	13,13016	0,197747	0,142935	3,607719	0,000143	4,7174E-92	TRUE
CVLT_7	APOE	173	sex	1,653315	0,350234	4,720597	5,08E-06	0,96167	2,344961	0,197747	0,142935	3,607719	0,000143	6,4937E-05	TRUE
CVLT_7	APOE	173	APOE:age_	-0,21705	0,127399	-1,70371	0,090365	-0,46864	0,034538	0,197747	0,142935	3,607719	0,000143	0,42343221	FALSE
CVLT_7	APOE	173	APOE:Stabi	1,000206	0,634473	1,576436	0,116888	-0,25276	2,253168	0,197747	0,142935	3,607719	0,000143	0,49804533	FALSE
CVLT_7	APOE	173	APOE:educ	0,763175	0,662318	1,152279	0,250915	-0,54478	2,071127	0,197747	0,142935	3,607719	0,000143	0,71495618	FALSE
CVLT_7	APOE	173	Plasticity	0,245499	0,282471	0,869115	0,386078	-0,31233	0,803324	0,197747	0,142935	3,607719	0,000143	0,85023979	FALSE
CVLT_7	APOE	173	age_c	-0,04752	0,064695	-0,73446	0,463736	-0,17528	0,080244	0,197747	0,142935	3,607719	0,000143	0,87396458	FALSE
CVLT_7	APOE	173	Stability	0,158533	0,332596	0,476654	0,634255	-0,49828	0,815345	0,197747	0,142935	3,607719	0,000143	0,90982023	FALSE

CVLT_7	APOE	173	APOE	0,109668	0,404227	0,271304	0,786505	-0,6886	0,907938	0,197747	0,142935	3,607719	0,000143	0,96374848	FALSE
CVLT_7	APOE	173	APOE:Plast	-0,1239	0,593491	-0,20876	0,834901	-1,29593	1,048135	0,197747	0,142935	3,607719	0,000143	0,97621258	FALSE
CVLT_7	APOE	173	age_c:educ	-0,00451	0,086864	-0,05197	0,958613	-0,17605	0,167025	0,197747	0,142935	3,607719	0,000143	0,99162101	FALSE
CVLT_7	APOE	173	education_	0,005182	0,30919	0,01676	0,986649	-0,60541	0,615773	0,197747	0,142935	3,607719	0,000143	0,99488912	FALSE
CVLT_7	APOE_PICALM	173	Intercept	12,48274	0,323501	38,58636	2,23E-81	11,8437	13,12178	0,228534	0,143922	2,700956	0,000648	5,6906E-80	TRUE
CVLT_7	APOE_PICALM	173	sex	1,598944	0,356577	4,484141	1,42E-05	0,894565	2,303322	0,228534	0,143922	2,700956	0,000648	0,00017006	TRUE
CVLT_7	APOE_PICALM	173	APOE:Stabi	1,228961	0,657943	1,867885	0,063666	-0,07073	2,528653	0,228534	0,143922	2,700956	0,000648	0,36345223	FALSE
CVLT_7	APOE_PICALM	173	PICALM:Pla	0,934483	0,499971	1,869075	0,063499	-0,05315	1,922119	0,228534	0,143922	2,700956	0,000648	0,36345223	FALSE
CVLT_7	APOE_PICALM	173	APOE:age_	-0,21843	0,128428	-1,70078	0,090989	-0,47212	0,035267	0,228534	0,143922	2,700956	0,000648	0,42343221	FALSE
CVLT_7	APOE_PICALM	173	APOE:PICA	-1,32096	0,845883	-1,56163	0,120414	-2,99191	0,349987	0,228534	0,143922	2,700956	0,000648	0,50626125	FALSE
CVLT_7	APOE_PICALM	173	APOE	0,757368	0,558457	1,35618	0,177014	-0,3458	1,860538	0,228534	0,143922	2,700956	0,000648	0,63081467	FALSE
CVLT_7	APOE_PICALM	173	APOE:educ	0,877173	0,6954	1,261393	0,209062	-0,49651	2,250858	0,228534	0,143922	2,700956	0,000648	0,68813815	FALSE
CVLT_7	APOE_PICALM	173	Stability	0,387125	0,395412	0,979041	0,329085	-0,39397	1,168217	0,228534	0,143922	2,700956	0,000648	0,79959595	FALSE
CVLT_7	APOE_PICALM	173	PICALM:Sta	-0,45108	0,611613	-0,73752	0,461921	-1,65925	0,757096	0,228534	0,143922	2,700956	0,000648	0,87396458	FALSE
CVLT_7	APOE_PICALM	173	age_c	-0,05124	0,079258	-0,6465	0,518909	-0,20781	0,105325	0,228534	0,143922	2,700956	0,000648	0,89521185	FALSE
CVLT_7	APOE_PICALM	173	Plasticity	-0,22752	0,373349	-0,6094	0,543153	-0,96503	0,509991	0,228534	0,143922	2,700956	0,000648	0,89687005	FALSE
CVLT_7	APOE_PICALM	173	PICALM	0,250489	0,417778	0,599573	0,549666	-0,57478	1,075762	0,228534	0,143922	2,700956	0,000648	0,89778791	FALSE
CVLT_7	APOE_PICALM	173	APOE:Plast	-0,08187	0,596596	-0,13723	0,891024	-1,26038	1,096634	0,228534	0,143922	2,700956	0,000648	0,98158997	FALSE
CVLT_7	APOE_PICALM	173	age_c:educ	0,013474	0,090376	0,149089	0,881677	-0,16505	0,192002	0,228534	0,143922	2,700956	0,000648	0,98158997	FALSE
CVLT_7	APOE_PICALM	173	education_	-0,01192	0,402977	-0,02958	0,976443	-0,80795	0,784117	0,228534	0,143922	2,700956	0,000648	0,99162101	FALSE
CVLT_7	APOE_PICALM	173	PICALM:ag	-0,00526	0,115661	-0,0455	0,963767	-0,23374	0,223212	0,228534	0,143922	2,700956	0,000648	0,99162101	FALSE
CVLT_7	APOE_PICALM	173	PICALM:ed	0,033383	0,594219	0,056179	0,955271	-1,14043	1,207196	0,228534	0,143922	2,700956	0,000648	0,99162101	FALSE
CVLT_7	PICALM	173	Intercept	12,52445	0,303252	41,30046	1,4E-87	11,92559	13,12332	0,177719	0,121539	3,163354	0,000667	4,1021E-86	TRUE
CVLT_7	PICALM	173	sex	1,689508	0,356264	4,742291	4,63E-06	0,985955	2,393061	0,177719	0,121539	3,163354	0,000667	6,0437E-05	TRUE
CVLT_7	PICALM	173	PICALM:Pla	0,894153	0,500933	1,784975	0,076148	-0,09509	1,883399	0,177719	0,121539	3,163354	0,000667	0,39693107	FALSE
CVLT_7	PICALM	173	Stability	0,620567	0,370012	1,677154	0,095453	-0,11014	1,35127	0,177719	0,121539	3,163354	0,000667	0,43848604	FALSE
CVLT_7	PICALM	173	age_c	-0,09177	0,073798	-1,2435	0,215491	-0,23751	0,053969	0,177719	0,121539	3,163354	0,000667	0,6923958	FALSE
CVLT_7	PICALM	173	Plasticity	-0,29175	0,346363	-0,84232	0,400856	-0,97575	0,39225	0,177719	0,121539	3,163354	0,000667	0,86252891	FALSE
CVLT_7	PICALM	173	education_	0,217897	0,346596	0,628678	0,530451	-0,46656	0,902358	0,177719	0,121539	3,163354	0,000667	0,89687005	FALSE
CVLT_7	PICALM	173	PICALM:Sta	-0,20801	0,595852	-0,34909	0,727479	-1,3847	0,968688	0,177719	0,121539	3,163354	0,000667	0,9442768	FALSE
CVLT_7	PICALM	173	PICALM:ag	-0,02421	0,114571	-0,21131	0,832911	-0,25047	0,202046	0,177719	0,121539	3,163354	0,000667	0,97621258	FALSE
CVLT_7	PICALM	173	PICALM:ed	-0,11346	0,578009	-0,19629	0,84463	-1,25491	1,027999	0,177719	0,121539	3,163354	0,000667	0,97621258	FALSE
CVLT_7	PICALM	173	PICALM	0,040771	0,356381	0,114404	0,90906	-0,66301	0,744555	0,177719	0,121539	3,163354	0,000667	0,98158997	FALSE
CVLT_7	PICALM	173	age_c:educ	-0,00474	0,089732	-0,05282	0,95794	-0,18194	0,172463	0,177719	0,121539	3,163354	0,000667	0,99162101	FALSE
CVLT_8	APOE	173	Intercept	13,07315	0,234169	55,82788	2,8E-107	12,61071	13,53559	0,138162	0,079279	2,346374	0,010444	1,859E-105	TRUE
CVLT_8	APOE	173	sex	1,090545	0,295324	3,692702	0,000303	0,507336	1,673754	0,138162	0,079279	2,346374	0,010444	0,00302427	TRUE
CVLT_8	APOE	173	APOE:age_	-0,19518	0,107425	-1,81686	0,071098	-0,40732	0,016968	0,138162	0,079279	2,346374	0,010444	0,39070826	FALSE
CVLT_8	APOE	173	APOE:Stabi	0,657265	0,535	1,228534	0,221039	-0,39926	1,713787	0,138162	0,079279	2,346374	0,010444	0,69257616	FALSE
CVLT_8	APOE	173	age_c:educ	-0,05501	0,073246	-0,751	0,453749	-0,19965	0,089638	0,138162	0,079279	2,346374	0,010444	0,87396458	FALSE

CVLT_8	APOE	173	APOE:Plast	0,355689	0,500443	0,710747	0,478269	-0,63259	1,343969	0,138162	0,079279	2,346374	0,010444	0,87971762	FALSE
CVLT_8	APOE	173	Plasticity	0,134648	0,238185	0,56531	0,57265	-0,33572	0,605017	0,138162	0,079279	2,346374	0,010444	0,90166392	FALSE
CVLT_8	APOE	173	age_c	-0,0264	0,054552	-0,48394	0,629085	-0,13413	0,081329	0,138162	0,079279	2,346374	0,010444	0,90982023	FALSE
CVLT_8	APOE	173	education_	0,097422	0,260715	0,373672	0,70914	-0,41744	0,612284	0,138162	0,079279	2,346374	0,010444	0,93282883	FALSE
CVLT_8	APOE	173	APOE	0,089357	0,340852	0,262157	0,793536	-0,58376	0,762473	0,138162	0,079279	2,346374	0,010444	0,9640474	FALSE
CVLT_8	APOE	173	APOE:educ	0,13039	0,558479	0,233473	0,81569	-0,9725	1,23328	0,138162	0,079279	2,346374	0,010444	0,9760277	FALSE
CVLT_8	APOE	173	Stability	-0,03267	0,280451	-0,1165	0,907401	-0,58651	0,521164	0,138162	0,079279	2,346374	0,010444	0,98158997	FALSE
CVLT_8	APOE_PICALM	173	Intercept	12,98912	0,273688	47,45956	3,17E-94	12,44848	13,52976	0,165724	0,074222	1,811163	0,03093	1,0961E-92	TRUE
CVLT_8	APOE_PICALM	173	sex	1,075345	0,301671	3,564626	0,000485	0,479428	1,671263	0,165724	0,074222	1,811163	0,03093	0,00459719	TRUE
CVLT_8	APOE_PICALM	173	APOE:age_	-0,19345	0,108653	-1,78048	0,076956	-0,40809	0,021177	0,165724	0,074222	1,811163	0,03093	0,39693107	FALSE
CVLT_8	APOE_PICALM	173	APOE:Stabi	0,889808	0,556632	1,598558	0,111955	-0,20976	1,989372	0,165724	0,074222	1,811163	0,03093	0,48404253	FALSE
CVLT_8	APOE_PICALM	173	PICALM:Pla	0,637118	0,422985	1,506243	0,13404	-0,19844	1,472677	0,165724	0,074222	1,811163	0,03093	0,54020622	FALSE
CVLT_8	APOE_PICALM	173	APOE:PICA	-0,98374	0,715633	-1,37464	0,171226	-2,39739	0,429912	0,165724	0,074222	1,811163	0,03093	0,61980636	FALSE
CVLT_8	APOE_PICALM	173	APOE	0,605819	0,472465	1,28225	0,201669	-0,32748	1,539121	0,165724	0,074222	1,811163	0,03093	0,6815037	FALSE
CVLT_8	APOE_PICALM	173	education_	0,261133	0,340926	0,765953	0,444869	-0,41233	0,934594	0,165724	0,074222	1,811163	0,03093	0,87396458	FALSE
CVLT_8	APOE_PICALM	173	PICALM:ed	-0,3747	0,502721	-0,74533	0,457198	-1,36776	0,618373	0,165724	0,074222	1,811163	0,03093	0,87396458	FALSE
CVLT_8	APOE_PICALM	173	age_c:educ	-0,05802	0,07646	-0,75885	0,449094	-0,20906	0,093016	0,165724	0,074222	1,811163	0,03093	0,87396458	FALSE
CVLT_8	APOE_PICALM	173	APOE:Plast	0,36631	0,504731	0,725752	0,469085	-0,63073	1,363349	0,165724	0,074222	1,811163	0,03093	0,87447583	FALSE
CVLT_8	APOE_PICALM	173	PICALM:Sta	-0,35173	0,517436	-0,67975	0,497678	-1,37386	0,670411	0,165724	0,074222	1,811163	0,03093	0,89217854	FALSE
CVLT_8	APOE_PICALM	173	Plasticity	-0,18695	0,31586	-0,59188	0,554792	-0,8109	0,436994	0,165724	0,074222	1,811163	0,03093	0,89894588	FALSE
CVLT_8	APOE_PICALM	173	PICALM	0,177324	0,353448	0,501697	0,616592	-0,52087	0,875521	0,165724	0,074222	1,811163	0,03093	0,90982023	FALSE
CVLT_8	APOE_PICALM	173	Stability	0,144373	0,334526	0,431574	0,666651	-0,51645	0,805191	0,165724	0,074222	1,811163	0,03093	0,91373127	FALSE
CVLT_8	APOE_PICALM	173	age_c	-0,02807	0,067054	-0,41859	0,676094	-0,16053	0,104389	0,165724	0,074222	1,811163	0,03093	0,91438395	FALSE
CVLT_8	APOE_PICALM	173	APOE:educ	0,10313	0,588322	0,175295	0,861077	-1,05903	1,265293	0,165724	0,074222	1,811163	0,03093	0,97932887	FALSE
CVLT_8	APOE_PICALM	173	PICALM:ag	-0,00413	0,097851	-0,04218	0,966406	-0,19742	0,189166	0,165724	0,074222	1,811163	0,03093	0,99162101	FALSE
CVLT_8	PICALM	173	Intercept	13,04862	0,255227	51,1256	1,9E-101	12,5446	13,55264	0,11997	0,059844	1,995297	0,031941	7,997E-100	TRUE
CVLT_8	PICALM	173	sex	1,122153	0,299843	3,742465	0,000253	0,53002	1,714285	0,11997	0,059844	1,995297	0,031941	0,00261146	TRUE
CVLT_8	PICALM	173	PICALM:Pla	0,586738	0,421601	1,391689	0,165936	-0,24584	1,419319	0,11997	0,059844	1,995297	0,031941	0,61365085	FALSE
CVLT_8	PICALM	173	age_c	-0,06746	0,062111	-1,08618	0,279021	-0,19012	0,055193	0,11997	0,059844	1,995297	0,031941	0,74237363	FALSE
CVLT_8	PICALM	173	Stability	0,291828	0,311414	0,937107	0,350107	-0,32315	0,906811	0,11997	0,059844	1,995297	0,031941	0,81110511	FALSE
CVLT_8	PICALM	173	age_c:educ	-0,05986	0,075521	-0,79266	0,42914	-0,209	0,089277	0,11997	0,059844	1,995297	0,031941	0,87170999	FALSE
CVLT_8	PICALM	173	education_	0,222405	0,291706	0,762429	0,44692	-0,35366	0,798469	0,11997	0,059844	1,995297	0,031941	0,87396458	FALSE
CVLT_8	PICALM	173	PICALM:ed	-0,3356	0,486471	-0,68987	0,491272	-1,29629	0,625087	0,11997	0,059844	1,995297	0,031941	0,8888249	FALSE
CVLT_8	PICALM	173	PICALM:Sta	-0,23752	0,501488	-0,47364	0,6364	-1,22787	0,752819	0,11997	0,059844	1,995297	0,031941	0,90982023	FALSE
CVLT_8	PICALM	173	Plasticity	-0,08776	0,29151	-0,30107	0,763751	-0,66344	0,487912	0,11997	0,059844	1,995297	0,031941	0,95347316	FALSE
CVLT_8	PICALM	173	PICALM:ag	-0,02554	0,096427	-0,26489	0,791435	-0,21597	0,164882	0,11997	0,059844	1,995297	0,031941	0,96374848	FALSE
CVLT_8	PICALM	173	PICALM	-0,0216	0,299941	-0,072	0,94269	-0,61392	0,57073	0,11997	0,059844	1,995297	0,031941	0,99162101	FALSE
CVLT_9	APOE	173	Intercept	2,904094	0,517709	5,609515	8,65E-08	1,881719	3,92647	0,140092	0,08134	2,38448	0,009222	1,4968E-06	TRUE
CVLT_9	APOE	173	APOE:educ	-3,23324	1,234706	-2,61863	0,009671	-5,67154	-0,79493	0,140092	0,08134	2,38448	0,009222	0,07790027	FALSE

CVLT_9	APOE	173	sex	1,436502	0,652913	2,200142	0,029221	0,147123	2,725881	0,140092	0,08134	2,38448	0,009222	0,20454907	FALSE
CVLT_9	APOE	173	age_c:educ	-0,32766	0,161934	-2,02341	0,044685	-0,64745	-0,00787	0,140092	0,08134	2,38448	0,009222	0,28252688	FALSE
CVLT_9	APOE	173	APOE:age_	0,427096	0,2375	1,798298	0,074003	-0,04192	0,896113	0,140092	0,08134	2,38448	0,009222	0,39693107	FALSE
CVLT_9	APOE	173	Plasticity	-0,92757	0,526587	-1,76148	0,080056	-1,96748	0,112336	0,140092	0,08134	2,38448	0,009222	0,40233055	FALSE
CVLT_9	APOE	173	APOE	0,824212	0,753567	1,093747	0,275699	-0,66394	2,312361	0,140092	0,08134	2,38448	0,009222	0,74237363	FALSE
CVLT_9	APOE	173	Stability	0,278087	0,620031	0,448506	0,654391	-0,94635	1,502529	0,140092	0,08134	2,38448	0,009222	0,91180601	FALSE
CVLT_9	APOE	173	APOE:Stabi	-0,49872	1,182796	-0,42165	0,673846	-2,83452	1,837074	0,140092	0,08134	2,38448	0,009222	0,91438395	FALSE
CVLT_9	APOE	173	age_c	0,048404	0,120605	0,401339	0,688703	-0,18977	0,286575	0,140092	0,08134	2,38448	0,009222	0,92035707	FALSE
CVLT_9	APOE	173	APOE:Plast	0,418028	1,106398	0,377828	0,706056	-1,7669	2,602951	0,140092	0,08134	2,38448	0,009222	0,93085444	FALSE
CVLT_9	APOE	173	education_	-0,183	0,576397	-0,31749	0,751287	-1,32127	0,955277	0,140092	0,08134	2,38448	0,009222	0,95185346	FALSE
CVLT_9	APOE_PICALM	173	Intercept	2,839519	0,58872	4,823211	3,35E-06	1,67657	4,002468	0,211995	0,125568	2,452895	0,001991	4,4748E-05	TRUE
CVLT_9	APOE_PICALM	173	PICALM:Pla	-2,92918	0,909866	-3,21935	0,001565	-4,72651	-1,13184	0,211995	0,125568	2,452895	0,001991	0,01415864	TRUE
CVLT_9	APOE_PICALM	173	sex	1,469377	0,648913	2,264367	0,024938	0,187523	2,751232	0,211995	0,125568	2,452895	0,001991	0,18103254	FALSE
CVLT_9	APOE_PICALM	173	APOE:educ	-2,85141	1,265515	-2,25316	0,025652	-5,3513	-0,35153	0,211995	0,125568	2,452895	0,001991	0,18394237	FALSE
CVLT_9	APOE_PICALM	173	age_c:educ	-0,30974	0,16447	-1,88328	0,061535	-0,63464	0,015148	0,211995	0,125568	2,452895	0,001991	0,35824532	FALSE
CVLT_9	APOE_PICALM	173	PICALM:Sta	1,914641	1,113036	1,720196	0,087393	-0,28404	4,113318	0,211995	0,125568	2,452895	0,001991	0,42120384	FALSE
CVLT_9	APOE_PICALM	173	APOE:age_	0,376462	0,233718	1,610751	0,109269	-0,08522	0,838146	0,211995	0,125568	2,452895	0,001991	0,4759335	FALSE
CVLT_9	APOE_PICALM	173	PICALM:ed	1,443278	1,081383	1,334659	0,183945	-0,69287	3,579427	0,211995	0,125568	2,452895	0,001991	0,64380764	FALSE
CVLT_9	APOE_PICALM	173	APOE:Stabi	-1,39322	1,197349	-1,16359	0,246379	-3,75845	0,972005	0,211995	0,125568	2,452895	0,001991	0,70915764	FALSE
CVLT_9	APOE_PICALM	173	education_	-0,83514	0,733352	-1,1388	0,256542	-2,2838	0,61351	0,211995	0,125568	2,452895	0,001991	0,72120934	FALSE
CVLT_9	APOE_PICALM	173	Stability	-0,46816	0,719586	-0,6506	0,516268	-1,88962	0,953298	0,211995	0,125568	2,452895	0,001991	0,89521185	FALSE
CVLT_9	APOE_PICALM	173	Plasticity	0,452673	0,679435	0,666249	0,506242	-0,88947	1,794819	0,211995	0,125568	2,452895	0,001991	0,89521185	FALSE
CVLT_9	APOE_PICALM	173	PICALM	0,397397	0,760288	0,522692	0,601935	-1,10447	1,89926	0,211995	0,125568	2,452895	0,001991	0,90982023	FALSE
CVLT_9	APOE_PICALM	173	age_c	0,068622	0,144237	0,475757	0,634917	-0,2163	0,353546	0,211995	0,125568	2,452895	0,001991	0,90982023	FALSE
CVLT_9	APOE_PICALM	173	APOE:PICA	0,822335	1,53937	0,534202	0,593967	-2,21852	3,863187	0,211995	0,125568	2,452895	0,001991	0,90982023	FALSE
CVLT_9	APOE_PICALM	173	APOE:Plast	0,491468	1,085707	0,452671	0,651419	-1,65322	2,636159	0,211995	0,125568	2,452895	0,001991	0,90982023	FALSE
CVLT_9	APOE_PICALM	173	PICALM:ag	0,080604	0,210484	0,382946	0,702284	-0,33518	0,496391	0,211995	0,125568	2,452895	0,001991	0,93005221	FALSE
CVLT_9	APOE_PICALM	173	APOE	0,065512	1,016301	0,064462	0,948686	-1,94208	2,073101	0,211995	0,125568	2,452895	0,001991	0,99162101	FALSE
CVLT_9	PICALM	173	Intercept	3,093585	0,552872	5,595481	9,26E-08	2,001769	4,185401	0,157037	0,099443	2,726623	0,002957	1,556E-06	TRUE
CVLT_9	PICALM	173	PICALM:Pla	-2,97256	0,913272	-3,25484	0,001383	-4,77609	-1,16902	0,157037	0,099443	2,726623	0,002957	0,01270602	TRUE
CVLT_9	PICALM	173	education_	-1,73248	0,631894	-2,74172	0,006803	-2,98035	-0,48461	0,157037	0,099443	2,726623	0,002957	0,05634427	FALSE
CVLT_9	PICALM	173	PICALM:ed	2,107402	1,053793	1,999826	0,047201	0,026363	4,188441	0,157037	0,099443	2,726623	0,002957	0,2891053	FALSE
CVLT_9	PICALM	173	sex	1,29008	0,64952	1,986204	0,048708	0,007402	2,572758	0,157037	0,099443	2,726623	0,002957	0,29526078	FALSE
CVLT_9	PICALM	173	PICALM:Sta	1,614149	1,086324	1,485883	0,139266	-0,53113	3,75943	0,157037	0,099443	2,726623	0,002957	0,55706281	FALSE
CVLT_9	PICALM	173	age_c:educ	-0,23414	0,163594	-1,43125	0,154298	-0,55721	0,088923	0,157037	0,099443	2,726623	0,002957	0,58533594	FALSE
CVLT_9	PICALM	173	Stability	-0,89667	0,674585	-1,32922	0,185655	-2,22885	0,435503	0,157037	0,099443	2,726623	0,002957	0,64594894	FALSE
CVLT_9	PICALM	173	Plasticity	0,788504	0,631469	1,248683	0,213594	-0,45853	2,035533	0,157037	0,099443	2,726623	0,002957	0,69007247	FALSE
CVLT_9	PICALM	173	age_c	0,140705	0,134545	1,04578	0,29723	-0,125	0,406405	0,157037	0,099443	2,726623	0,002957	0,76654107	FALSE
CVLT_9	PICALM	173	PICALM	0,396015	0,649733	0,609504	0,543049	-0,88708	1,679113	0,157037	0,099443	2,726623	0,002957	0,89687005	FALSE

CVLT_9	PICALM	173	PICALM:ag	0,098616	0,20888	0,472117	0,637483	-0,31388	0,511114	0,157037	0,099443	2,726623	0,002957	0,90982023	FALSE
RPM	APOE	173	Intercept	53,65509	0,538688	99,60326	1,5E-146	52,59128	54,71889	0,120229	0,060121	2,000202	0,031459	2,154E-144	TRUE
RPM	APOE	173	age_c	-0,25266	0,125492	-2,01334	0,045745	-0,50048	-0,00484	0,120229	0,060121	2,000202	0,031459	0,28614674	FALSE
RPM	APOE	173	sex	-1,24758	0,679372	-1,83637	0,068147	-2,58921	0,094051	0,120229	0,060121	2,000202	0,031459	0,3816248	FALSE
RPM	APOE	173	APOE:Stabi	2,145548	1,230727	1,743318	0,083187	-0,2849	4,575998	0,120229	0,060121	2,000202	0,031459	0,40761667	FALSE
RPM	APOE	173	Plasticity	0,82495	0,547926	1,505586	0,134133	-0,2571	1,906998	0,120229	0,060121	2,000202	0,031459	0,54020622	FALSE
RPM	APOE	173	APOE:age_	-0,31445	0,247124	-1,27244	0,205052	-0,80247	0,173573	0,120229	0,060121	2,000202	0,031459	0,68506061	FALSE
RPM	APOE	173	education_	0,71922	0,599755	1,199189	0,232216	-0,46518	1,903621	0,120229	0,060121	2,000202	0,031459	0,6975188	FALSE
RPM	APOE	173	Stability	-0,40407	0,645157	-0,62631	0,531997	-1,67813	0,869991	0,120229	0,060121	2,000202	0,031459	0,89687005	FALSE
RPM	APOE	173	age_c:educ	0,08675	0,168496	0,514849	0,607365	-0,246	0,419497	0,120229	0,060121	2,000202	0,031459	0,90982023	FALSE
RPM	APOE	173	APOE:Plast	-0,50304	1,151233	-0,43696	0,662728	-2,7765	1,770425	0,120229	0,060121	2,000202	0,031459	0,91260908	FALSE
RPM	APOE	173	APOE:educ	-0,19432	1,28474	-0,15125	0,879968	-2,73143	2,3428	0,120229	0,060121	2,000202	0,031459	0,98158997	FALSE
RPM	APOE	173	APOE	-0,06809	0,784104	-0,08683	0,930911	-1,61654	1,480368	0,120229	0,060121	2,000202	0,031459	0,98995361	FALSE
RPM	APOE_PICALM	173	Intercept	53,81375	0,625024	86,09876	8,7E-133	52,57909	55,04842	0,160698	0,068646	1,745723	0,040099	8,539E-131	TRUE
RPM	APOE_PICALM	173	age_c	-0,37979	0,153131	-2,48019	0,014201	-0,68229	-0,0773	0,160698	0,068646	1,745723	0,040099	0,10844118	FALSE
RPM	APOE_PICALM	173	APOE:Stabi	2,745551	1,271184	2,159838	0,032322	0,23447	5,256631	0,160698	0,068646	1,745723	0,040099	0,21845514	FALSE
RPM	APOE_PICALM	173	education_	1,633801	0,778575	2,098451	0,037487	0,095814	3,171788	0,160698	0,068646	1,745723	0,040099	0,24222289	FALSE
RPM	APOE_PICALM	173	PICALM:ed	-2,04441	1,148067	-1,78074	0,076913	-4,31229	0,223462	0,160698	0,068646	1,745723	0,040099	0,39693107	FALSE
RPM	APOE_PICALM	173	sex	-1,17002	0,688929	-1,69831	0,091456	-2,53092	0,190886	0,160698	0,068646	1,745723	0,040099	0,42343221	FALSE
RPM	APOE_PICALM	173	PICALM:Sta	-1,44594	1,181673	-1,22363	0,222947	-3,7802	0,888325	0,160698	0,068646	1,745723	0,040099	0,69257616	FALSE
RPM	APOE_PICALM	173	APOE:age_	-0,29465	0,248131	-1,18747	0,236857	-0,7848	0,195505	0,160698	0,068646	1,745723	0,040099	0,69985965	FALSE
RPM	APOE_PICALM	173	PICALM:ag	0,251752	0,223464	1,126592	0,261656	-0,18967	0,69318	0,160698	0,068646	1,745723	0,040099	0,72120934	FALSE
RPM	APOE_PICALM	173	Plasticity	0,531768	0,721332	0,737202	0,462114	-0,89314	1,956678	0,160698	0,068646	1,745723	0,040099	0,87396458	FALSE
RPM	APOE_PICALM	173	PICALM	-0,47581	0,807172	-0,58948	0,5564	-2,07029	1,118668	0,160698	0,068646	1,745723	0,040099	0,89894588	FALSE
RPM	APOE_PICALM	173	PICALM:Pla	0,559737	0,965973	0,579454	0,563124	-1,34843	2,467908	0,160698	0,068646	1,745723	0,040099	0,89894588	FALSE
RPM	APOE_PICALM	173	APOE:educ	-0,77207	1,343554	-0,57465	0,566363	-3,42611	1,88197	0,160698	0,068646	1,745723	0,040099	0,89894588	FALSE
RPM	APOE_PICALM	173	APOE:Plast	-0,64581	1,152658	-0,56028	0,576101	-2,92275	1,63114	0,160698	0,068646	1,745723	0,040099	0,90166392	FALSE
RPM	APOE_PICALM	173	Stability	0,144229	0,763959	0,188791	0,850503	-1,36489	1,653345	0,160698	0,068646	1,745723	0,040099	0,97621258	FALSE
RPM	APOE_PICALM	173	APOE:PICA	0,319502	1,634296	0,195499	0,845259	-2,90887	3,54787	0,160698	0,068646	1,745723	0,040099	0,97621258	FALSE
RPM	APOE_PICALM	173	APOE	0,072015	1,078972	0,066744	0,946871	-2,05937	2,203403	0,160698	0,068646	1,745723	0,040099	0,99162101	FALSE
RPM	APOE_PICALM	173	age_c:educ	-0,00328	0,174612	-0,01877	0,985051	-0,3482	0,34165	0,160698	0,068646	1,745723	0,040099	0,99488912	FALSE
RPM	PICALM	173	Intercept	53,74249	0,579118	92,80058	1,1E-141	52,59884	54,88614	0,126009	0,066295	2,110213	0,022295	1,255E-139	TRUE
RPM	PICALM	173	age_c	-0,45174	0,140932	-3,20539	0,001627	-0,73006	-0,17343	0,126009	0,066295	2,110213	0,022295	0,01449265	TRUE
RPM	PICALM	173	education_	1,406102	0,661892	2,124369	0,035166	0,098993	2,713211	0,126009	0,066295	2,110213	0,022295	0,2323346	FALSE
RPM	PICALM	173	sex	-1,21207	0,680354	-1,78153	0,076711	-2,55564	0,131498	0,126009	0,066295	2,110213	0,022295	0,39693107	FALSE
RPM	PICALM	173	PICALM:ed	-1,77827	1,103819	-1,61102	0,109134	-3,9581	0,401558	0,126009	0,066295	2,110213	0,022295	0,4759335	FALSE
RPM	PICALM	173	PICALM:ag	0,267284	0,218796	1,221613	0,22364	-0,1648	0,699365	0,126009	0,066295	2,110213	0,022295	0,69257616	FALSE
RPM	PICALM	173	Stability	0,742263	0,706609	1,050458	0,295082	-0,65315	2,137679	0,126009	0,066295	2,110213	0,022295	0,76435261	FALSE
RPM	PICALM	173	PICALM:Sta	-1,06855	1,137893	-0,93906	0,349105	-3,31568	1,178567	0,126009	0,066295	2,110213	0,022295	0,81110511	FALSE

RPM	PICALM	173	Plasticity	0,430127	0,661446	0,650284	0,516436	-0,8761	1,736356	0,126009	0,066295	2,110213	0,022295	0,89521185	FALSE
RPM	PICALM	173	PICALM:Pla	0,374323	0,956627	0,391295	0,696097	-1,51483	2,263478	0,126009	0,066295	2,110213	0,022295	0,92393898	FALSE
RPM	PICALM	173	PICALM	-0,24452	0,680577	-0,35929	0,719851	-1,58853	1,099486	0,126009	0,066295	2,110213	0,022295	0,93861873	FALSE
RPM	PICALM	173	age_c:educ	0,008871	0,17136	0,051769	0,958777	-0,32953	0,347274	0,126009	0,066295	2,110213	0,022295	0,99162101	FALSE
