# Supplementary material for: Sex-specific personality metatrait profiles and their associations with APOE, PICALM variants, and cognitive performance in healthy middle-aged adults: a cluster-based analysis
Source: Front Aging. 2026 Oct 29;7:1933176. doi: 10.3389/fragi.2026.1933176 (PMC13754840; doi:10.3389/fragi.2026.1933176)
Supplement: Supplementary file 2 [file DataSheet2.pdf]

Supplementary Table 1: All Regression Models

Sex	Cluster	Outcome	Model	N	R2	F	F_pvalue	p_FDR	Significant_FDR
Men	0	CVLT_1	Model 1a APOE	59	0.002619262427583924	0.14969003585899426	0.7002724761634047	0.9909019975283825	False
Men	0	CVLT_1	Model 1b APOE x age	59	0.007113602578579181	0.13135042197440705	0.9410290483911878	0.9909019975283825	False
Men	0	CVLT_1	Model 1c APOE x age x education	59	0.125306078335357	1.0437299978914978	0.4130013589458404	0.9909019975283825	False
Men	0	CVLT_1	Model 2a PICALM	59	0.0034051931602049335	0.19475920283706688	0.6606543092715946	0.9909019975283825	False
Men	0	CVLT_1	Model 2b PICALM x age	59	0.023862636493516054	0.4481763380885985	0.7195819451423138	0.9909019975283825	False
Men	0	CVLT_1	Model 2c PICALM x age x education	59	0.10756110465901991	1.0445490948957377	0.40768252050681936	0.9909019975283825	False
Men	0	CVLT_1	Model 3a APOE x PICALM	59	0.09221896714830013	1.8624326827741757	0.1466880161627691	0.9909019975283825	False
Men	0	CVLT_1	Model 3b APOE x PICALM x age	59	0.10814723420052874	1.285369879968908	0.2840853508906652	0.9909019975283825	False
Men	0	CVLT_1	Model 3c APOE x PICALM x age x education	59	0.21766590985820378	1.5147875678760865	0.16928851632709377	0.9909019975283825	False
Men	0	CVLT_10	Model 1a APOE	59	6.713984270101392e-05	0.0038272279934403746	0.9508870839525299	0.9909019975283825	False
Men	0	CVLT_10	Model 1b APOE x age	59	0.08002663932565834	1.594779932788826	0.20109523378719255	0.9909019975283825	False
Men	0	CVLT_10	Model 1c APOE x age x education	59	0.12230901228731594	1.0152873056331417	0.4318504513880959	0.9909019975283825	False
Men	0	CVLT_10	Model 2a PICALM	59	0.012510048139724006	0.722106328900817	0.39900908309609906	0.9909019975283825	False
Men	0	CVLT_10	Model 2b PICALM x age	59	0.11488545678098572	2.3796167292179518	0.07952961428252657	0.9909019975283825	False
Men	0	CVLT_10	Model 2c PICALM x age x education	59	0.16749965084986318	1.7437393776239907	0.12939442813055319	0.9909019975283825	False
Men	0	CVLT_10	Model 3a APOE x PICALM	59	0.012552450483358402	0.2330536533046432	0.8729648842006696	0.9909019975283825	False
Men	0	CVLT_10	Model 3b APOE x PICALM x age	59	0.11609150755036035	1.3921916018969926	0.24218936727439544	0.9909019975283825	False
Men	0	CVLT_10	Model 3c APOE x PICALM x age x education	59	0.1664470031830111	1.0871671810126489	0.38922866468128636	0.9909019975283825	False
Men	0	CVLT_11	Model 1a APOE	59	0.008244832705264438	0.4738623802505587	0.49400785062791897	0.9909019975283825	False
Men	0	CVLT_11	Model 1b APOE x age	59	0.10487934937727561	2.1480769889277602	0.10461415907715049	0.9909019975283825	False
Men	0	CVLT_11	Model 1c APOE x age x education	59	0.1674004219021512	1.4648477820196157	0.20091393276551975	0.9909019975283825	False

Sex	Cluster	Outcome	Model	N	R2	F	F_pvalue	p_FDR	Significant_FDR
Men	0	CVLT_11	Model 2a PICALM	59	0.011803057553956942	0.6808099192355912	0.41274785712956563	0.9909019975283825	False
Men	0	CVLT_11	Model 2b PICALM x age	59	0.10862229709698079	2.234079642811054	0.0944839577004482	0.9909019975283825	False
Men	0	CVLT_11	Model 2c PICALM x age x education	59	0.1253301973107599	1.2418343928421087	0.3005589939539685	0.9909019975283825	False
Men	0	CVLT_11	Model 3a APOE x PICALM	59	0.06959789311408038	1.3714085171505215	0.2611194009558209	0.9909019975283825	False
Men	0	CVLT_11	Model 3b APOE x PICALM x age	59	0.22173323029887793	3.0200084761048185	0.017988663319416398	0.7452589081642306	False
Men	0	CVLT_11	Model 3c APOE x PICALM x age x education	59	0.2464832922124407	1.7809354153202697	0.0960850788112887	0.9909019975283825	False
Men	0	CVLT_12	Model 1a APOE	59	0.009351887478090015	0.538089740961718	0.4662319596831688	0.9909019975283825	False
Men	0	CVLT_12	Model 1b APOE x age	59	0.017810417965855696	0.3324453194644759	0.8019038675353298	0.9909019975283825	False
Men	0	CVLT_12	Model 1c APOE x age x education	59	0.041797735342530506	0.3178100999421444	0.9425743029672888	0.9909019975283825	False
Men	0	CVLT_12	Model 2a PICALM	59	0.0206123234210962	1.1996295880570296	0.2780021968686464	0.9909019975283825	False
Men	0	CVLT_12	Model 2b PICALM x age	59	0.04156743991196987	0.795120870736541	0.5018630662066379	0.9909019975283825	False
Men	0	CVLT_12	Model 2c PICALM x age x education	59	0.04702034170526581	0.4276162922947408	0.8572095872718448	0.9909019975283825	False
Men	0	CVLT_12	Model 3a APOE x PICALM	59	0.058193034813001865	1.1327929653750266	0.3438852004891202	0.9909019975283825	False
Men	0	CVLT_12	Model 3b APOE x PICALM x age	59	0.06192972571868349	0.6997930865264599	0.6260114725399012	0.9909019975283825	False
Men	0	CVLT_12	Model 3c APOE x PICALM x age x education	59	0.09169448573085359	0.5496229248649116	0.8307567440860837	0.9909019975283825	False
Men	0	CVLT_13	Model 1a APOE	59	0.016064158064158174	0.9306064182553881	0.33878046898227343	0.9909019975283825	False
Men	0	CVLT_13	Model 1b APOE x age	59	0.06797568810148724	1.3371120609383478	0.2717358803873311	0.9909019975283825	False
Men	0	CVLT_13	Model 1c APOE x age x education	59	0.16289947090969725	1.4177974581282928	0.21871704368944364	0.9909019975283825	False
Men	0	CVLT_13	Model 2a PICALM	59	0.005575924075924221	0.3196097922632617	0.5740608764518571	0.9909019975283825	False
Men	0	CVLT_13	Model 2b PICALM x age	59	0.05437185562435953	1.0541324928219178	0.37609967213836487	0.9909019975283825	False
Men	0	CVLT_13	Model 2c PICALM x age x education	59	0.08764589416566748	0.8325690042696151	0.5502940500770492	0.9909019975283825	False
Men	0	CVLT_13	Model 3a APOE x PICALM	59	0.1278803549391785	2.688247177050386	0.05522389834151345	0.9909019975283825	False
Men	0	CVLT_13	Model 3b APOE x PICALM x age	59	0.21626763135854044	2.9250251541534427	0.02097872966552182	0.7452589081642306	False

Sex	Cluster	Outcome	Model	N	R2	F	F_pvalue	p_FDR	Significant_FDR
Men	0	CVLT_13	Model 3c APOE x PICALM x age x education	59	0.26655598665526337	1.9786776281130174	0.062265486444689246	0.9909019975283825	False
Men	0	CVLT_2	Model 1a APOE	59	0.004208767293642168	0.24091368537720878	0.6254313945043342	0.9909019975283825	False
Men	0	CVLT_2	Model 1b APOE x age	59	0.005525805539624673	0.10186934508450578	0.9586098062669399	0.9909019975283825	False
Men	0	CVLT_2	Model 1c APOE x age x education	59	0.06647595710393905	0.5188134510453354	0.8161608445929133	0.9909019975283825	False
Men	0	CVLT_2	Model 2a PICALM	59	0.0005911359828661977	0.03371468098445106	0.8549666649865646	0.9909019975283825	False
Men	0	CVLT_2	Model 2b PICALM x age	59	0.011703664244053669	0.2171081385660791	0.8841213002714751	0.9909019975283825	False
Men	0	CVLT_2	Model 2c PICALM x age x education	59	0.06340795072233152	0.5867395237346864	0.7392980711801711	0.9909019975283825	False
Men	0	CVLT_2	Model 3a APOE x PICALM	59	0.07116491863339469	1.4046521294458036	0.25120646564360033	0.9909019975283825	False
Men	0	CVLT_2	Model 3b APOE x PICALM x age	59	0.07400939547294305	0.8472003799799611	0.5225946803138621	0.9909019975283825	False
Men	0	CVLT_2	Model 3c APOE x PICALM x age x education	59	0.13032189998427268	0.8158539859127037	0.6042083181003366	0.9909019975283825	False
Men	0	CVLT_3	Model 1a APOE	59	0.0026368986161695585	0.1507006033340531	0.6993128543198699	0.9909019975283825	False
Men	0	CVLT_3	Model 1b APOE x age	59	0.0322345888551262	0.610651564458025	0.6109561893554254	0.9909019975283825	False
Men	0	CVLT_3	Model 1c APOE x age x education	59	0.17328503855832456	1.5271349132514824	0.17932813564618	0.9909019975283825	False
Men	0	CVLT_3	Model 2a PICALM	59	0.0028935066997970438	0.16540849246959405	0.6857482059147244	0.9909019975283825	False
Men	0	CVLT_3	Model 2b PICALM x age	59	0.06915984533925457	1.36213558422224	0.26395029602279707	0.9909019975283825	False
Men	0	CVLT_3	Model 2c PICALM x age x education	59	0.1695079109396096	1.7689133717561403	0.12383503019819112	0.9909019975283825	False
Men	0	CVLT_3	Model 3a APOE x PICALM	59	0.029312654296886897	0.5536269371270431	0.6478766194560036	0.9909019975283825	False
Men	0	CVLT_3	Model 3b APOE x PICALM x age	59	0.06484002737129979	0.7349590554050243	0.600535746798666	0.9909019975283825	False
Men	0	CVLT_3	Model 3c APOE x PICALM x age x education	59	0.22130545089458953	1.5473143275916494	0.1581942503497689	0.9909019975283825	False
Men	0	CVLT_4	Model 1a APOE	59	0.0018009768009767946	0.10284089171585126	0.7496195069526448	0.9909019975283825	False
Men	0	CVLT_4	Model 1b APOE x age	59	0.0337676523018714	0.6407088595296334	0.5920804561957498	0.9909019975283825	False
Men	0	CVLT_4	Model 1c APOE x age x education	59	0.0614301379565626	0.47685574807303394	0.8468665804284748	0.9909019975283825	False
Men	0	CVLT_4	Model 2a PICALM	59	0.021611721611721646	1.259078996630479	0.2665301212938694	0.9909019975283825	False

Sex	Cluster	Outcome	Model	N	R2	F	F_pvalue	p_FDR	Significant_FDR
Men	0	CVLT_4	Model 2b PICALM x age	59	0.034216677688901354	0.6495305345807065	0.5866197112499807	0.9909019975283825	False
Men	0	CVLT_4	Model 2c PICALM x age x education	59	0.05682613919785062	0.5221658772037183	0.7888692258186658	0.9909019975283825	False
Men	0	CVLT_4	Model 3a APOE x PICALM	59	0.05055147058823528	0.9761213294611152	0.41070815233147173	0.9909019975283825	False
Men	0	CVLT_4	Model 3b APOE x PICALM x age	59	0.0544834255904173	0.6108029482392223	0.6919677484691742	0.9909019975283825	False
Men	0	CVLT_4	Model 3c APOE x PICALM x age x education	59	0.08354565341270281	0.4963255074015227	0.8698548929913691	0.9909019975283825	False
Men	0	CVLT_5	Model 1a APOE	59	0.0023982378351634637	0.1370281827767332	0.7126254230547767	0.9909019975283825	False
Men	0	CVLT_5	Model 1b APOE x age	59	0.006808006040747383	0.1256689993873331	0.9445234992100984	0.9909019975283825	False
Men	0	CVLT_5	Model 1c APOE x age x education	59	0.14578506091084154	1.2434204230312385	0.29706486092255185	0.9909019975283825	False
Men	0	CVLT_5	Model 2a PICALM	59	0.0008450842705539641	0.04821054539516121	0.8269912568026802	0.9909019975283825	False
Men	0	CVLT_5	Model 2b PICALM x age	59	0.02573992200921449	0.48436611622445697	0.6945214236477678	0.9909019975283825	False
Men	0	CVLT_5	Model 2c PICALM x age x education	59	0.08833148914741507	0.8397126406121062	0.5450774227187899	0.9909019975283825	False
Men	0	CVLT_5	Model 3a APOE x PICALM	59	0.021694877498088516	0.4065596833236569	0.7488781407942418	0.9909019975283825	False
Men	0	CVLT_5	Model 3b APOE x PICALM x age	59	0.031485362339065515	0.34459452425016945	0.8834507373359881	0.9909019975283825	False
Men	0	CVLT_5	Model 3c APOE x PICALM x age x education	59	0.17111884757469475	1.1239814734498241	0.3643087453487341	0.9909019975283825	False
Men	0	CVLT_6	Model 1a APOE	59	0.004545056024128025	0.26025104897322116	0.6119176041027464	0.9909019975283825	False
Men	0	CVLT_6	Model 1b APOE x age	59	0.019763719543045566	0.369640326024518	0.7751965051881415	0.9909019975283825	False
Men	0	CVLT_6	Model 1c APOE x age x education	59	0.14517113235145596	1.2372948936034023	0.3001937514949357	0.9909019975283825	False
Men	0	CVLT_6	Model 2a PICALM	59	0.00018265560796537628	0.010413271696500961	0.919078750311864	0.9909019975283825	False
Men	0	CVLT_6	Model 2b PICALM x age	59	0.0475263620875771	0.9147934426636203	0.4398583833220102	0.9909019975283825	False
Men	0	CVLT_6	Model 2c PICALM x age x education	59	0.09721766571776214	0.9332848804104579	0.47922162174048666	0.9909019975283825	False
Men	0	CVLT_6	Model 3a APOE x PICALM	59	0.05581061441392576	1.0836751749263995	0.3636925038241449	0.9909019975283825	False
Men	0	CVLT_6	Model 3b APOE x PICALM x age	59	0.08754382848988207	1.016996334691852	0.41703949958238784	0.9909019975283825	False
Men	0	CVLT_6	Model 3c APOE x PICALM x age x education	59	0.1944451973804603	1.3141825623587584	0.2540941379761907	0.9909019975283825	False

Sex	Cluster	Outcome	Model	N	R2	F	F_pvalue	p_FDR	Significant_FDR
Men	0	CVLT_7	Model 1a APOE	59	2.5098601649453656e-05	0.0014306562014862157	0.9699601583104138	0.9909019975283825	False
Men	0	CVLT_7	Model 1b APOE x age	59	0.0037046239492395383	0.06817065236745484	0.9766191905353543	0.9909019975283825	False
Men	0	CVLT_7	Model 1c APOE x age x education	59	0.1691723063505447	1.4835098764117913	0.19421536892814664	0.9909019975283825	False
Men	0	CVLT_7	Model 2a PICALM	59	0.00038522938755991554	0.021966537246608672	0.8826992283243945	0.9909019975283825	False
Men	0	CVLT_7	Model 2b PICALM x age	59	0.02394046927146376	0.4496740101319077	0.7185365945853128	0.9909019975283825	False
Men	0	CVLT_7	Model 2c PICALM x age x education	59	0.09592915175834005	0.9196026876904478	0.48854525590552134	0.9909019975283825	False
Men	0	CVLT_7	Model 3a APOE x PICALM	59	0.02829573564814969	0.5338611474504871	0.6609967980258848	0.9909019975283825	False
Men	0	CVLT_7	Model 3b APOE x PICALM x age	59	0.04535566312099115	0.5036116703465442	0.7721696331889205	0.9909019975283825	False
Men	0	CVLT_7	Model 3c APOE x PICALM x age x education	59	0.21480463988259213	1.4894279661494663	0.1784156781562447	0.9909019975283825	False
Men	0	CVLT_8	Model 1a APOE	59	0.0001686757826555585	0.009616141621578367	0.9222271469517875	0.9909019975283825	False
Men	0	CVLT_8	Model 1b APOE x age	59	0.02841303101672754	0.536138899830153	0.659476756165877	0.9909019975283825	False
Men	0	CVLT_8	Model 1c APOE x age x education	59	0.13820434405290483	1.1683945687883979	0.33723618470021327	0.9909019975283825	False
Men	0	CVLT_8	Model 2a PICALM	59	0.0005520071168685359	0.03148178383023122	0.8597988265991314	0.9909019975283825	False
Men	0	CVLT_8	Model 2b PICALM x age	59	0.05454471988540621	1.0576772400184216	0.3745910477719652	0.9909019975283825	False
Men	0	CVLT_8	Model 2c PICALM x age x education	59	0.1200284811506479	1.1821369377945008	0.3303013162909932	0.9909019975283825	False
Men	0	CVLT_8	Model 3a APOE x PICALM	59	0.04486103320421353	0.8610812708957618	0.466838617548893	0.9909019975283825	False
Men	0	CVLT_8	Model 3b APOE x PICALM x age	59	0.09867784500577326	1.1605008833582882	0.34081240589107686	0.9909019975283825	False
Men	0	CVLT_8	Model 3c APOE x PICALM x age x education	59	0.1867148359416695	1.249941098353872	0.28787697004472573	0.9909019975283825	False
Men	0	CVLT_9	Model 1a APOE	59	0.001213027747435702	0.06922655533631665	0.7934150173326344	0.9909019975283825	False
Men	0	CVLT_9	Model 1b APOE x age	59	0.020844632816512054	0.39028699064919853	0.7604468721789363	0.9909019975283825	False
Men	0	CVLT_9	Model 1c APOE x age x education	59	0.29249856578392575	3.0120942180762436	0.010001931618266107	0.7452589081642306	False
Men	0	CVLT_9	Model 2a PICALM	59	0.007353478434151128	0.4222533012914111	0.518425137423852	0.9909019975283825	False
Men	0	CVLT_9	Model 2b PICALM x age	59	0.021772125042705315	0.408039513084163	0.7478289120080752	0.9909019975283825	False

Sex	Cluster	Outcome	Model	N	R2	F	F_pvalue	p_FDR	Significant_FDR
Men	0	CVLT_9	Model 2c PICALM x age x education	59	0.12549095821763134	1.2436558715689798	0.2996871887771013	0.9909019975283825	False
Men	0	CVLT_9	Model 3a APOE x PICALM	59	0.011288218015042317	0.20931343934592514	0.889528976019452	0.9909019975283825	False
Men	0	CVLT_9	Model 3b APOE x PICALM x age	59	0.02303932570322842	0.24997613402403535	0.9379990926685867	0.9909019975283825	False
Men	0	CVLT_9	Model 3c APOE x PICALM x age x education	59	0.2892047684333794	2.215208016144908	0.03674654091502133	0.8405261361982522	False
Men	0	RPM	Model 1a APOE	59	0.00035712097679274635	0.020363167791561394	0.8870306927856706	0.9909019975283825	False
Men	0	RPM	Model 1b APOE x age	59	0.017383343455078193	0.32433261525441887	0.8077409991002766	0.9909019975283825	False
Men	0	RPM	Model 1c APOE x age x education	59	0.07848229763872216	0.6204976807466108	0.7364919286261528	0.9909019975283825	False
Men	0	RPM	Model 2a PICALM	59	0.0013124170650574696	0.07490608072689942	0.7853123805897968	0.9909019975283825	False
Men	0	RPM	Model 2b PICALM x age	59	0.031136663082754534	0.5891840483923604	0.6246882303006498	0.9909019975283825	False
Men	0	RPM	Model 2c PICALM x age x education	59	0.04404699501458165	0.39932990583101974	0.8761018035504609	0.9909019975283825	False
Men	0	RPM	Model 3a APOE x PICALM	59	0.022022201169563216	0.41283182016826764	0.744434534102356	0.9909019975283825	False
Men	0	RPM	Model 3b APOE x PICALM x age	59	0.041557140213140054	0.4596055797809839	0.8044492628250156	0.9909019975283825	False
Men	0	RPM	Model 3c APOE x PICALM x age x education	59	0.10526936379337948	0.640565081469628	0.756874171685451	0.9909019975283825	False
Men	1	CVLT_1	Model 1a APOE	17	7.009319591322338e-05	0.0010514716397063027	0.9745595516341979	0.9909019975283825	False
Men	1	CVLT_1	Model 1b APOE x age	17	0.12909216761358222	0.6423175589767649	0.6013152610800578	0.9909019975283825	False
Men	1	CVLT_1	Model 1c APOE x age x education	17	0.21508306855509396	0.602844367174184	0.6995423595536998	0.9909019975283825	False
Men	1	CVLT_1	Model 2a PICALM	17	0.007534707035363186	0.11387864777904651	0.740446796840984	0.9909019975283825	False
Men	1	CVLT_1	Model 2b PICALM x age	17	0.12638816570512157	0.626916932542372	0.610318212904573	0.9909019975283825	False
Men	1	CVLT_1	Model 2c PICALM x age x education	17	0.18427152676519898	0.4969758592290149	0.7725101715824091	0.9909019975283825	False
Men	1	CVLT_1	Model 3a APOE x PICALM	17	0.007952707252896984	0.0347379925142394	0.9909019975283825	0.9909019975283825	False
Men	1	CVLT_1	Model 3b APOE x PICALM x age	17	0.17153340414748786	0.45550839468204135	0.8011742496122183	0.9909019975283825	False
Men	1	CVLT_1	Model 3c APOE x PICALM x age x education	17	0.24786381010359326	0.4237025765315926	0.8647625615313032	0.9909019975283825	False
Men	1	CVLT_10	Model 1a APOE	17	0.00567542213883665	0.08561724609651201	0.7738332929895784	0.9909019975283825	False

Sex	Cluster	Outcome	Model	N	R2	F	F_pvalue	p_FDR	Significant_FDR
Men	1	CVLT_10	Model 1b APOE x age	17	0.1362510738984387	0.6835566475218343	0.5777710358995265	0.9909019975283825	False
Men	1	CVLT_10	Model 1c APOE x age x education	17	0.33691062095569924	1.1178031039658967	0.4055128256510748	0.9909019975283825	False
Men	1	CVLT_10	Model 2a PICALM	17	0.0008468834688344362	0.01271401932530518	0.9117188259278022	0.9909019975283825	False
Men	1	CVLT_10	Model 2b PICALM x age	17	0.10787746806039478	0.5239964371770375	0.6733567918246817	0.9909019975283825	False
Men	1	CVLT_10	Model 2c PICALM x age x education	17	0.15437143542440612	0.4016150496336903	0.8378783338667317	0.9909019975283825	False
Men	1	CVLT_10	Model 3a APOE x PICALM	17	0.11840882694541244	0.582021386382101	0.6372097327155617	0.9909019975283825	False
Men	1	CVLT_10	Model 3b APOE x PICALM x age	17	0.3764732106195522	1.3283167258715158	0.32173564130053484	0.9909019975283825	False
Men	1	CVLT_10	Model 3c APOE x PICALM x age x education	17	0.3925632364284384	0.8309081560270377	0.5872967650808125	0.9909019975283825	False
Men	1	CVLT_11	Model 1a APOE	17	0.09732170663344752	1.6172158012765225	0.22284476874114462	0.9909019975283825	False
Men	1	CVLT_11	Model 1b APOE x age	17	0.11105747792727194	0.5413725399208716	0.662371728881947	0.9909019975283825	False
Men	1	CVLT_11	Model 1c APOE x age x education	17	0.3237415310253656	1.0531940092901362	0.43531398290508877	0.9909019975283825	False
Men	1	CVLT_11	Model 2a PICALM	17	0.02878992352676568	0.4446503010653083	0.5150134419320139	0.9909019975283825	False
Men	1	CVLT_11	Model 2b PICALM x age	17	0.08253325621746677	0.3898169745109167	0.7623150142351972	0.9909019975283825	False
Men	1	CVLT_11	Model 2c PICALM x age x education	17	0.17173515100711956	0.4561552173499405	0.8007289524813622	0.9909019975283825	False
Men	1	CVLT_11	Model 3a APOE x PICALM	17	0.4231733179101602	3.1790329768270964	0.06000013063266596	0.9909019975283825	False
Men	1	CVLT_11	Model 3b APOE x PICALM x age	17	0.5033543454596088	2.2297176062799475	0.12437498616069406	0.9909019975283825	False
Men	1	CVLT_11	Model 3c APOE x PICALM x age x education	17	0.5061628307409747	1.3178043753930322	0.34201104134510857	0.9909019975283825	False
Men	1	CVLT_12	Model 1a APOE	17	0.021485411140583444	0.3293575494714003	0.5745428845875511	0.9909019975283825	False
Men	1	CVLT_12	Model 1b APOE x age	17	0.05197479952697093	0.2375718822360019	0.8685820531819788	0.9909019975283825	False
Men	1	CVLT_12	Model 1c APOE x age x education	17	0.12094013898662914	0.3026737058200602	0.9012324576656147	0.9909019975283825	False
Men	1	CVLT_12	Model 2a PICALM	17	0.00019157088122589983	0.002874113814905498	0.9579527798804355	0.9909019975283825	False
Men	1	CVLT_12	Model 2b PICALM x age	17	0.09266009852216783	0.44253216787013616	0.7265648025009763	0.9909019975283825	False
Men	1	CVLT_12	Model 2c PICALM x age x education	17	0.22397311256266084	0.6349533187761367	0.6778338041504781	0.9909019975283825	False

Sex	Cluster	Outcome	Model	N	R2	F	F_pvalue	p_FDR	Significant_FDR
Men	1	CVLT_12	Model 3a APOE x PICALM	17	0.02159277504105095	0.09563368856815208	0.9610895779175452	0.9909019975283825	False
Men	1	CVLT_12	Model 3b APOE x PICALM x age	17	0.05889685027616043	0.13768211342781644	0.9798710049214447	0.9909019975283825	False
Men	1	CVLT_12	Model 3c APOE x PICALM x age x education	17	0.13801376302034385	0.20585742454798617	0.9754693641910877	0.9909019975283825	False
Men	1	CVLT_13	Model 1a APOE	17	0.26536251105216635	5.418233789679557	0.0343322630567567	0.8405261361982522	False
Men	1	CVLT_13	Model 1b APOE x age	17	0.460661219813709	3.7011961544898355	0.04002505410467867	0.8405261361982522	False
Men	1	CVLT_13	Model 1c APOE x age x education	17	0.6430917194463994	3.9640486362142644	0.026616389577293946	0.7452589081642306	False
Men	1	CVLT_13	Model 2a PICALM	17	0.17632503192848026	3.211066963853088	0.09333277147041459	0.9909019975283825	False
Men	1	CVLT_13	Model 2b PICALM x age	17	0.49633962780514507	4.270347981616067	0.026395864406158955	0.7452589081642306	False
Men	1	CVLT_13	Model 2c PICALM x age x education	17	0.7013119830642238	5.165544900561053	0.011064471453993552	0.7452589081642306	False
Men	1	CVLT_13	Model 3a APOE x PICALM	17	0.5254515599343187	4.798154555940027	0.018326401791691087	0.7452589081642306	False
Men	1	CVLT_13	Model 3b APOE x PICALM x age	17	0.6835168875398762	4.751397763055035	0.014756227773761564	0.7452589081642306	False
Men	1	CVLT_13	Model 3c APOE x PICALM x age x education	17	0.7969265094824454	5.045561561455645	0.014134062005220629	0.7452589081642306	False
Men	1	CVLT_2	Model 1a APOE	17	0.019230769230769162	0.29411764705882326	0.5955598372302722	0.9909019975283825	False
Men	1	CVLT_2	Model 1b APOE x age	17	0.3110686359800908	1.9566014259848763	0.1704269936207533	0.9909019975283825	False
Men	1	CVLT_2	Model 1c APOE x age x education	17	0.3126378676470589	1.000641839941165	0.46106962161448944	0.9909019975283825	False
Men	1	CVLT_2	Model 2a PICALM	17	0.003472222222222321	0.05226480836237004	0.8222556864200737	0.9909019975283825	False
Men	1	CVLT_2	Model 2b PICALM x age	17	0.202316073488219	1.0990606128286677	0.3845915180646992	0.9909019975283825	False
Men	1	CVLT_2	Model 2c PICALM x age x education	17	0.28498642815823405	0.8768646731182115	0.5272417723081062	0.9909019975283825	False
Men	1	CVLT_2	Model 3a APOE x PICALM	17	0.08333333333333337	0.39393939393939426	0.7594877485296153	0.9909019975283825	False
Men	1	CVLT_2	Model 3b APOE x PICALM x age	17	0.36363636363636376	1.2571428571428578	0.34789454108977014	0.9909019975283825	False
Men	1	CVLT_2	Model 3c APOE x PICALM x age x education	17	0.3829422990570276	0.7979065551722129	0.608383825400498	0.9909019975283825	False
Men	1	CVLT_3	Model 1a APOE	17	0.08754048582995944	1.4390855342702786	0.24889345025164178	0.9909019975283825	False
Men	1	CVLT_3	Model 1b APOE x age	17	0.19972899833342606	1.0814990475840247	0.39126015803984654	0.9909019975283825	False

Sex	Cluster	Outcome	Model	N	R2	F	F_pvalue	p_FDR	Significant_FDR
Men	1	CVLT_3	Model 1c APOE x age x education	17	0.33469183002148206	1.1067382895223363	0.41047322981847995	0.9909019975283825	False
Men	1	CVLT_3	Model 2a PICALM	17	0.032814327485380046	0.5089146027163267	0.4865608369858824	0.9909019975283825	False
Men	1	CVLT_3	Model 2b PICALM x age	17	0.0866372180451126	0.4110392412296672	0.7478142145213158	0.9909019975283825	False
Men	1	CVLT_3	Model 2c PICALM x age x education	17	0.2446772758889616	0.7126622697460147	0.6267063716440251	0.9909019975283825	False
Men	1	CVLT_3	Model 3a APOE x PICALM	17	0.13934837092731822	0.701611337604348	0.5677222522572031	0.9909019975283825	False
Men	1	CVLT_3	Model 3b APOE x PICALM x age	17	0.24655109997215274	0.7199060479332954	0.6220589156671366	0.9909019975283825	False
Men	1	CVLT_3	Model 3c APOE x PICALM x age x education	17	0.34921310910864933	0.6899159915847948	0.6806187529996088	0.9909019975283825	False
Men	1	CVLT_4	Model 1a APOE	17	0.031968031968031885	0.495356037151701	0.4923297330361903	0.9909019975283825	False
Men	1	CVLT_4	Model 1b APOE x age	17	0.08541339379679569	0.4046907137524232	0.7521377023416258	0.9909019975283825	False
Men	1	CVLT_4	Model 1c APOE x age x education	17	0.40249335326663127	1.4819674091118986	0.2720189627916354	0.9909019975283825	False
Men	1	CVLT_4	Model 2a PICALM	17	0.14793019480519465	2.604191474160512	0.12741662216222063	0.9909019975283825	False
Men	1	CVLT_4	Model 2b PICALM x age	17	0.17410134508348807	0.9134766808703823	0.46138529908065196	0.9909019975283825	False
Men	1	CVLT_4	Model 2c PICALM x age x education	17	0.2936275767573322	0.9145043713636172	0.5062966862688261	0.9909019975283825	False
Men	1	CVLT_4	Model 3a APOE x PICALM	17	0.17602040816326514	0.9256965944272437	0.45588140920084885	0.9909019975283825	False
Men	1	CVLT_4	Model 3b APOE x PICALM x age	17	0.3508039579468152	1.1888068587758998	0.37505177963113967	0.9909019975283825	False
Men	1	CVLT_4	Model 3c APOE x PICALM x age x education	17	0.4495190945913409	1.04990584766675	0.46158180080693767	0.9909019975283825	False
Men	1	CVLT_5	Model 1a APOE	17	0.012833716232274361	0.19500842629002418	0.6650799594528725	0.9909019975283825	False
Men	1	CVLT_5	Model 1b APOE x age	17	0.07743634025331603	0.3637228400294596	0.780316572031811	0.9909019975283825	False
Men	1	CVLT_5	Model 1c APOE x age x education	17	0.0783003990731207	0.18689481669259328	0.9615402772633259	0.9909019975283825	False
Men	1	CVLT_5	Model 2a PICALM	17	0.09458319029637263	1.566955505177768	0.22981874616041023	0.9909019975283825	False
Men	1	CVLT_5	Model 2b PICALM x age	17	0.18975099308518462	1.0148167987271353	0.41768910413717575	0.9909019975283825	False
Men	1	CVLT_5	Model 2c PICALM x age x education	17	0.20059202866302583	0.552036605690331	0.7343870843428526	0.9909019975283825	False
Men	1	CVLT_5	Model 3a APOE x PICALM	17	0.1748442940512972	0.9182007736158369	0.45924974051657186	0.9909019975283825	False

Sex	Cluster	Outcome	Model	N	R2	F	F_pvalue	p_FDR	Significant_FDR
Men	1	CVLT_5	Model 3b APOE x PICALM x age	17	0.30506167555601127	0.965748560147682	0.47893705819855603	0.9909019975283825	False
Men	1	CVLT_5	Model 3c APOE x PICALM x age x education	17	0.3814733037813045	0.7929579746333735	0.6115895544717378	0.9909019975283825	False
Men	1	CVLT_6	Model 1a APOE	17	0.011710211304257423	0.1777344778556031	0.6793068715264283	0.9909019975283825	False
Men	1	CVLT_6	Model 1b APOE x age	17	0.048455803904392525	0.2206677851790187	0.8803147922264702	0.9909019975283825	False
Men	1	CVLT_6	Model 1c APOE x age x education	17	0.2332459404600814	0.6692381509138445	0.6550056985254837	0.9909019975283825	False
Men	1	CVLT_6	Model 2a PICALM	17	0.01657645466847102	0.2528379772961835	0.622385930390671	0.9909019975283825	False
Men	1	CVLT_6	Model 2b PICALM x age	17	0.018427411560023255	0.0813512089686717	0.9690013456022905	0.9909019975283825	False
Men	1	CVLT_6	Model 2c PICALM x age x education	17	0.2578251441949857	0.7642610266198356	0.5940950560118614	0.9909019975283825	False
Men	1	CVLT_6	Model 3a APOE x PICALM	17	0.034651072878407185	0.15554443053817316	0.9242420093166761	0.9909019975283825	False
Men	1	CVLT_6	Model 3b APOE x PICALM x age	17	0.05064547791205454	0.11736400766435541	0.9858168918735766	0.9909019975283825	False
Men	1	CVLT_6	Model 3c APOE x PICALM x age x education	17	0.3660587932349856	0.7424143041201761	0.6449404466632552	0.9909019975283825	False
Men	1	CVLT_7	Model 1a APOE	17	0.0006845212629417174	0.010274852298998167	0.9206033114184279	0.9909019975283825	False
Men	1	CVLT_7	Model 1b APOE x age	17	0.015828420173024438	0.0696929500462012	0.9751095789831675	0.9909019975283825	False
Men	1	CVLT_7	Model 1c APOE x age x education	17	0.25369330435177395	0.7478497417058908	0.6043404578654858	0.9909019975283825	False
Men	1	CVLT_7	Model 2a PICALM	17	0.019311580768755277	0.2953779262106528	0.5947803473957782	0.9909019975283825	False
Men	1	CVLT_7	Model 2b PICALM x age	17	0.03230441230997394	0.14465890767437736	0.9312747271240334	0.9909019975283825	False
Men	1	CVLT_7	Model 2c PICALM x age x education	17	0.23376619487569972	0.6711862949496344	0.6537207383277488	0.9909019975283825	False
Men	1	CVLT_7	Model 3a APOE x PICALM	17	0.02096244504475875	0.0927822037069598	0.9627032572399696	0.9909019975283825	False
Men	1	CVLT_7	Model 3b APOE x PICALM x age	17	0.09867130973916272	0.24084097596331655	0.9359662099215385	0.9909019975283825	False
Men	1	CVLT_7	Model 3c APOE x PICALM x age x education	17	0.4005618444339585	0.8591513251512799	0.5696693998286075	0.9909019975283825	False
Men	1	CVLT_8	Model 1a APOE	17	0.017111823361823264	0.2611460352542603	0.616772702025242	0.9909019975283825	False
Men	1	CVLT_8	Model 1b APOE x age	17	0.04040532795753826	0.1824621994930356	0.9064064635950015	0.9909019975283825	False
Men	1	CVLT_8	Model 1c APOE x age x education	17	0.28304138321995453	0.868517413013999	0.5319835965816606	0.9909019975283825	False

Sex	Cluster	Outcome	Model	N	R2	F	F_pvalue	p_FDR	Significant_FDR
Men	1	CVLT_8	Model 2a PICALM	17	0.0004629629629628873	0.006947660954144154	0.9346733501331933	0.9909019975283825	False
Men	1	CVLT_8	Model 2b PICALM x age	17	0.008055555555555594	0.03519088957341572	0.9907280878517286	0.9909019975283825	False
Men	1	CVLT_8	Model 2c PICALM x age x education	17	0.26721990874646273	0.802264971796039	0.5708465520211194	0.9909019975283825	False
Men	1	CVLT_8	Model 3a APOE x PICALM	17	0.02519841269841261	0.11201574055227576	0.9515284343160666	0.9909019975283825	False
Men	1	CVLT_8	Model 3b APOE x PICALM x age	17	0.068366201699535	0.1614428802533301	0.9716818509073947	0.9909019975283825	False
Men	1	CVLT_8	Model 3c APOE x PICALM x age x education	17	0.43166399087200624	0.9765289384779943	0.5008442676275657	0.9909019975283825	False
Men	1	CVLT_9	Model 1a APOE	17	0.11747834566626536	1.9967500812479742	0.17805104471091632	0.9909019975283825	False
Men	1	CVLT_9	Model 1b APOE x age	17	0.14713110170089383	0.7475569910475747	0.5428605642984755	0.9909019975283825	False
Men	1	CVLT_9	Model 1c APOE x age x education	17	0.1906067614405943	0.5180854684623489	0.7578779352995976	0.9909019975283825	False
Men	1	CVLT_9	Model 2a PICALM	17	0.054939307316265173	0.871996493054611	0.36519261353903176	0.9909019975283825	False
Men	1	CVLT_9	Model 2b PICALM x age	17	0.21767382195234564	1.2057032666529373	0.3465668089401514	0.9909019975283825	False
Men	1	CVLT_9	Model 2c PICALM x age x education	17	0.33093952282054073	1.0881930334227519	0.41891907504900383	0.9909019975283825	False
Men	1	CVLT_9	Model 3a APOE x PICALM	17	0.2771030858279183	1.6610667649105593	0.22406835187993943	0.9909019975283825	False
Men	1	CVLT_9	Model 3b APOE x PICALM x age	17	0.32356446823262663	1.0523424579011935	0.4357203769921534	0.9909019975283825	False
Men	1	CVLT_9	Model 3c APOE x PICALM x age x education	17	0.40749906277725323	0.8842641986082296	0.554334070802748	0.9909019975283825	False
Men	1	RPM	Model 1a APOE	17	0.0009497645211932193	0.014260011470864373	0.9065310133674619	0.9909019975283825	False
Men	1	RPM	Model 1b APOE x age	17	0.06347320597591422	0.2936921409873594	0.8292627093775577	0.9909019975283825	False
Men	1	RPM	Model 1c APOE x age x education	17	0.1482653061224486	0.3829639391398097	0.8503194282845982	0.9909019975283825	False
Men	1	RPM	Model 2a PICALM	17	0.022500000000000075	0.34526854219948916	0.5655451666774496	0.9909019975283825	False
Men	1	RPM	Model 2b PICALM x age	17	0.2229081632653055	1.243013152999361	0.3342170826140771	0.9909019975283825	False
Men	1	RPM	Model 2c PICALM x age x education	17	0.31106718720060444	0.9933447777883666	0.46475528074164085	0.9909019975283825	False
Men	1	RPM	Model 3a APOE x PICALM	17	0.07532555879494662	0.35300073218495936	0.7877605552681572	0.9909019975283825	False
Men	1	RPM	Model 3b APOE x PICALM x age	17	0.0804770739464622	0.19254502271312415	0.9591097040513874	0.9909019975283825	False

Sex	Cluster	Outcome	Model	N	R2	F	F_pvalue	p_FDR	Significant_FDR
Men	1	RPM	Model 3c APOE x PICALM x age x education	17	0.3622739412013445	0.7303775267111097	0.6530352099256946	0.9909019975283825	False
Women	0	CVLT_1	Model 1a APOE	64	0.00360142608023839	0.22409548028193066	0.637599517717847	0.8828301014554805	False
Women	0	CVLT_1	Model 1b APOE x age	64	0.07474269586664228	1.615608880529726	0.19518503857285271	0.5810027418406003	False
Women	0	CVLT_1	Model 1c APOE x age x education	64	0.11507851397969371	1.0403500495595654	0.41394418484510415	0.7924186384559482	False
Women	0	CVLT_1	Model 2a PICALM	64	0.003627825559700093	0.22574414508088253	0.6363671518876534	0.8828301014554805	False
Women	0	CVLT_1	Model 2b PICALM x age	64	0.05599645843815315	1.1863611940588148	0.3225827326081189	0.739007714702236	False
Women	0	CVLT_1	Model 2c PICALM x age x education	64	0.08374718012453641	0.8683173398486599	0.5238325132960495	0.8374587597448505	False
Women	0	CVLT_1	Model 3a APOE x PICALM	64	0.019305364753896104	0.393707971065866	0.7579700806695968	0.9246743820217322	False
Women	0	CVLT_1	Model 3b APOE x PICALM x age	64	0.058620350718089465	0.7223398857714228	0.6093680639210799	0.8817651745440186	False
Women	0	CVLT_1	Model 3c APOE x PICALM x age x education	64	0.0725909422575165	0.5381257858697794	0.8226613587901364	0.9276818767163478	False
Women	0	CVLT_10	Model 1a APOE	64	7.373271889488109e-06	0.00045714622781236865	0.9830104100054423	0.9908744932854858	False
Women	0	CVLT_10	Model 1b APOE x age	64	0.03653154497327005	0.7583340125496167	0.5219042243166245	0.8374587597448505	False
Women	0	CVLT_10	Model 1c APOE x age x education	64	0.07803408131730993	0.677110333352065	0.6905663264688288	0.9022146030567919	False
Women	0	CVLT_10	Model 2a PICALM	64	0.11733358559581242	8.241711917690763	0.005594176316780505	0.1084409562945144	False
Women	0	CVLT_10	Model 2b PICALM x age	64	0.13502198083732353	3.1219748443556683	0.03244835045257004	0.32962218890848494	False
Women	0	CVLT_10	Model 2c PICALM x age x education	64	0.19526887267350435	2.305185207090489	0.046242528639339274	0.32962218890848494	False
Women	0	CVLT_10	Model 3a APOE x PICALM	64	0.1301284808381582	2.9919011709578096	0.03786127155185016	0.32962218890848494	False
Women	0	CVLT_10	Model 3b APOE x PICALM x age	64	0.13315417754719672	1.781849112656454	0.13086306573281214	0.5216042312623503	False
Women	0	CVLT_10	Model 3c APOE x PICALM x age x education	64	0.1905577199493661	1.6185024639063095	0.140750348118412	0.5216042312623503	False
Women	0	CVLT_11	Model 1a APOE	64	0.03135888501742157	2.007194244604315	0.1615598682024763	0.5524041466076356	False
Women	0	CVLT_11	Model 1b APOE x age	64	0.11971806764804171	2.7199937485522634	0.052320982366426176	0.32962218890848494	False
Women	0	CVLT_11	Model 1c APOE x age x education	64	0.21601894186966353	2.204328174814147	0.047453087988663455	0.32962218890848494	False
Women	0	CVLT_11	Model 2a PICALM	64	0.06697184273895518	4.450298972760254	0.03893927327763848	0.32962218890848494	False

Sex	Cluster	Outcome	Model	N	R2	F	F_pvalue	p_FDR	Significant_FDR
Women	0	CVLT_11	Model 2b PICALM x age	64	0.06927637350942306	1.4886561711265303	0.22672834074983939	0.6078249134995694	False
Women	0	CVLT_11	Model 2c PICALM x age x education	64	0.1178990047976557	1.2697418455137377	0.28577033233510996	0.7145867283413682	False
Women	0	CVLT_11	Model 3a APOE x PICALM	64	0.08757556806337297	1.9196234778039272	0.13602107727285723	0.5216042312623503	False
Women	0	CVLT_11	Model 3b APOE x PICALM x age	64	0.09349009450925072	1.1963301114952638	0.3223559536517681	0.739007714702236	False
Women	0	CVLT_11	Model 3c APOE x PICALM x age x education	64	0.14255661265800978	1.1430220659371824	0.3501282833964326	0.7541224565461625	False
Women	0	CVLT_12	Model 1a APOE	64	0.08389027431421447	5.677482578397213	0.020263214481520048	0.2636477453525185	False
Women	0	CVLT_12	Model 1b APOE x age	64	0.25438775571718775	6.8235938362808835	0.0004940662317712465	0.018648362109577262	True
Women	0	CVLT_12	Model 1c APOE x age x education	64	0.3256952931639012	3.8640726053014722	0.0016989071813756284	0.0535155762133323	False
Women	0	CVLT_12	Model 2a PICALM	64	0.0012958108847417327	0.08044451573309253	0.7776408978719145	0.9246743820217322	False
Women	0	CVLT_12	Model 2b PICALM x age	64	0.052059781672853855	1.09837689479459	0.35689785636698507	0.7621886424108495	False
Women	0	CVLT_12	Model 2c PICALM x age x education	64	0.1041873248740165	1.1048957151270022	0.3709087124815652	0.7724710375649126	False
Women	0	CVLT_12	Model 3a APOE x PICALM	64	0.08390196669498406	1.831724633056795	0.15103050337715385	0.5437098121577538	False
Women	0	CVLT_12	Model 3b APOE x PICALM x age	64	0.183522699478738	2.6073759951369553	0.034030140475404355	0.32962218890848494	False
Women	0	CVLT_12	Model 3c APOE x PICALM x age x education	64	0.23655182484553683	2.1301954064976694	0.04807393112001646	0.32962218890848494	False
Women	0	CVLT_13	Model 1a APOE	64	0.018096311805443577	1.1426490657148776	0.28923748528103	0.7145867283413682	False
Women	0	CVLT_13	Model 1b APOE x age	64	0.024872027110793393	0.510128471386173	0.6768172915538181	0.9022146030567919	False
Women	0	CVLT_13	Model 1c APOE x age x education	64	0.296743217679751	3.375645711664051	0.0044844617426378665	0.1084409562945144	False
Women	0	CVLT_13	Model 2a PICALM	64	0.0003978395439282467	0.024675868759923794	0.8756878677110655	0.9485554664635422	False
Women	0	CVLT_13	Model 2b PICALM x age	64	0.009795528171897105	0.19784859492328402	0.8974587568925061	0.9487978794513342	False
Women	0	CVLT_13	Model 2c PICALM x age x education	64	0.09516735835514789	0.9991791440353022	0.43489684610515844	0.800362408716167	False
Women	0	CVLT_13	Model 3a APOE x PICALM	64	0.01839100396033888	0.37471139801159375	0.7715403021576475	0.9246743820217322	False
Women	0	CVLT_13	Model 3b APOE x PICALM x age	64	0.025980714595396637	0.30941511510360936	0.9053521421345704	0.950619749241299	False
Women	0	CVLT_13	Model 3c APOE x PICALM x age x education	64	0.06113367999728092	0.44766122825674404	0.8868288483108516	0.9485554664635422	False

Sex	Cluster	Outcome	Model	N	R2	F	F_pvalue	p_FDR	Significant_FDR
Women	0	CVLT_2	Model 1a APOE	64	0.005874695375882921	0.36638350478610826	0.5471914945621985	0.8493622603305039	False
Women	0	CVLT_2	Model 1b APOE x age	64	0.019412153889636352	0.3959289107373148	0.7563878636449118	0.9246743820217322	False
Women	0	CVLT_2	Model 1c APOE x age x education	64	0.05933846567848866	0.5046530639421866	0.8271312986634689	0.9276818767163478	False
Women	0	CVLT_2	Model 2a PICALM	64	0.0007854332009221698	0.04873513665155143	0.8260044131626647	0.9276818767163478	False
Women	0	CVLT_2	Model 2b PICALM x age	64	0.03882759032550309	0.807921449569117	0.4944378392892168	0.8208670527723201	False
Women	0	CVLT_2	Model 2c PICALM x age x education	64	0.08534766133035576	0.8864600770798736	0.5109095720125991	0.8374587597448505	False
Women	0	CVLT_2	Model 3a APOE x PICALM	64	0.015121800870019353	0.30707961417721635	0.8201653434820392	0.9276818767163478	False
Women	0	CVLT_2	Model 3b APOE x PICALM x age	64	0.019124099490941626	0.22616475130013078	0.9497147666577851	0.9616127543964292	False
Women	0	CVLT_2	Model 3c APOE x PICALM x age x education	64	0.05530763186396093	0.40250136646597257	0.914382588680736	0.9521669931716754	False
Women	0	CVLT_3	Model 1a APOE	64	0.0016091543000178454	0.0999283667621726	0.7529773931923246	0.9246743820217322	False
Women	0	CVLT_3	Model 1b APOE x age	64	0.08706609154540135	1.9073909017748198	0.1380182604131174	0.5216042312623503	False
Women	0	CVLT_3	Model 1c APOE x age x education	64	0.10422734903625963	0.9308375193113918	0.49019848262205845	0.8208670527723201	False
Women	0	CVLT_3	Model 2a PICALM	64	0.0007646410407926574	0.04744402217564305	0.8282873899253106	0.9276818767163478	False
Women	0	CVLT_3	Model 2b PICALM x age	64	0.07423036578622522	1.6036465885871605	0.1979660106659478	0.5810027418406003	False
Women	0	CVLT_3	Model 2c PICALM x age x education	64	0.08277809390152213	0.8573627459569515	0.5317198474570479	0.8374587597448505	False
Women	0	CVLT_3	Model 3a APOE x PICALM	64	0.015350384561898966	0.3117938466886845	0.8167720798417353	0.9276818767163478	False
Women	0	CVLT_3	Model 3b APOE x PICALM x age	64	0.09201680814033131	1.1755668871377207	0.3322506953530186	0.7475640645442919	False
Women	0	CVLT_3	Model 3c APOE x PICALM x age x education	64	0.1026352934077096	0.7863220349478199	0.6166379904558819	0.8817651745440186	False
Women	0	CVLT_4	Model 1a APOE	64	0.008287905448026378	0.5181444701546926	0.47433913222678536	0.8208670527723201	False
Women	0	CVLT_4	Model 1b APOE x age	64	0.028225114386211336	0.5808982060363475	0.6298322675314418	0.8817651745440186	False
Women	0	CVLT_4	Model 1c APOE x age x education	64	0.11563610448556083	1.0460499807563461	0.41020143078213916	0.7924186384559482	False
Women	0	CVLT_4	Model 2a PICALM	64	0.026726449004979336	1.7025427605781078	0.19677817053865468	0.5810027418406003	False
Women	0	CVLT_4	Model 2b PICALM x age	64	0.034184911773232574	0.7078976543221326	0.5510647029692708	0.8493622603305039	False

Sex	Cluster	Outcome	Model	N	R2	F	F_pvalue	p_FDR	Significant_FDR
Women	0	CVLT_4	Model 2c PICALM x age x education	64	0.0828454346784292	0.8581232206690589	0.5311702977600808	0.8374587597448505	False
Women	0	CVLT_4	Model 3a APOE x PICALM	64	0.04732734665339822	0.9935699631377893	0.40206904867297955	0.7924186384559482	False
Women	0	CVLT_4	Model 3b APOE x PICALM x age	64	0.057155133910375744	0.7031905005858472	0.623314102677024	0.8817651745440186	False
Women	0	CVLT_4	Model 3c APOE x PICALM x age x education	64	0.10082272025702288	0.7708782432371576	0.6297013351074721	0.8817651745440186	False
Women	0	CVLT_5	Model 1a APOE	64	0.0012927054478301114	0.08025147928994084	0.7779006705897112	0.9246743820217322	False
Women	0	CVLT_5	Model 1b APOE x age	64	0.08123453097450906	1.7683409686842568	0.16285318429822124	0.5524041466076356	False
Women	0	CVLT_5	Model 1c APOE x age x education	64	0.14433369272035257	1.3494390651348314	0.2449515665468821	0.6298754568348397	False
Women	0	CVLT_5	Model 2a PICALM	64	0.003487831581835743	0.2170024259885118	0.6429658605709383	0.8853956112780135	False
Women	0	CVLT_5	Model 2b PICALM x age	64	0.05537106906737366	1.1723348132627291	0.3278420460661354	0.7442900505285236	False
Women	0	CVLT_5	Model 2c PICALM x age x education	64	0.08949575835538515	0.9337789605905101	0.47807151250982394	0.8208670527723201	False
Women	0	CVLT_5	Model 3a APOE x PICALM	64	0.005785448777138491	0.1163822993743657	0.9501649835107575	0.9616127543964292	False
Women	0	CVLT_5	Model 3b APOE x PICALM x age	64	0.051069289539443674	0.6242855796816059	0.6818232401493148	0.9022146030567919	False
Women	0	CVLT_5	Model 3c APOE x PICALM x age x education	64	0.07369954338819185	0.5469978527778696	0.8158492493189518	0.9276818767163478	False
Women	0	CVLT_6	Model 1a APOE	64	0.029909148760442372	1.9115397498883364	0.17175233084758498	0.5694945707051502	False
Women	0	CVLT_6	Model 1b APOE x age	64	0.11477444538407544	2.5931118862440194	0.06086726149084363	0.3567104626905254	False
Women	0	CVLT_6	Model 1c APOE x age x education	64	0.17386511092288148	1.6836486459697397	0.13180658605365297	0.5216042312623503	False
Women	0	CVLT_6	Model 2a PICALM	64	0.002283652654661572	0.14191053896805658	0.7076752320651973	0.9022146030567919	False
Women	0	CVLT_6	Model 2b PICALM x age	64	0.043222097151150995	0.9034927964463911	0.44485266438870075	0.8062684896547795	False
Women	0	CVLT_6	Model 2c PICALM x age x education	64	0.051632591854491094	0.5172147612883871	0.7928595014097033	0.9276818767163478	False
Women	0	CVLT_6	Model 3a APOE x PICALM	64	0.031373378146195896	0.6477909534667131	0.5874103185613255	0.8817651745440186	False
Women	0	CVLT_6	Model 3b APOE x PICALM x age	64	0.06655102134109392	0.8270316484419072	0.5356648523234924	0.8384319427672056	False
Women	0	CVLT_6	Model 3c APOE x PICALM x age x education	64	0.07373984986293636	0.5473208231323238	0.8155998197397555	0.9276818767163478	False
Women	0	CVLT_7	Model 1a APOE	64	0.01765402173819941	1.114219808488555	0.29526246040261866	0.7188789006482053	False

Sex	Cluster	Outcome	Model	N	R2	F	F_pvalue	p_FDR	Significant_FDR
Women	0	CVLT_7	Model 1b APOE x age	64	0.15046784670238456	3.542369670607896	0.01975795686041704	0.2636477453525185	False
Women	0	CVLT_7	Model 1c APOE x age x education	64	0.20004850020469267	2.0006062893151033	0.07114181843831895	0.38144123928630586	False
Women	0	CVLT_7	Model 2a PICALM	64	0.0014080489528657258	0.087422129715881	0.7684683334052025	0.9246743820217322	False
Women	0	CVLT_7	Model 2b PICALM x age	64	0.06030344366058993	1.2834663116251521	0.2882641054044382	0.7145867283413682	False
Women	0	CVLT_7	Model 2c PICALM x age x education	64	0.0632227121628135	0.6411510754422948	0.6968202923168644	0.9022146030567919	False
Women	0	CVLT_7	Model 3a APOE x PICALM	64	0.022680959679580615	0.46414648121752716	0.7083739685997998	0.9022146030567919	False
Women	0	CVLT_7	Model 3b APOE x PICALM x age	64	0.07194573866422704	0.8992691518961555	0.487846146908951	0.8208670527723201	False
Women	0	CVLT_7	Model 3c APOE x PICALM x age x education	64	0.07337055196322328	0.5443627394055579	0.8178805928688646	0.9276818767163478	False
Women	0	CVLT_8	Model 1a APOE	64	0.021912892280570362	1.3890371426765442	0.2430712989856361	0.6298754568348397	False
Women	0	CVLT_8	Model 1b APOE x age	64	0.14743046494646506	3.4584971403466227	0.021806198655865337	0.2636477453525185	False
Women	0	CVLT_8	Model 1c APOE x age x education	64	0.1754743686740987	1.7025485880109974	0.12712750547308996	0.5216042312623503	False
Women	0	CVLT_8	Model 2a PICALM	64	0.0021952203536395043	0.1364030967800013	0.7131417348211091	0.9022146030567919	False
Women	0	CVLT_8	Model 2b PICALM x age	64	0.02381908012416889	0.4880054432368681	0.6919073649651117	0.9022146030567919	False
Women	0	CVLT_8	Model 2c PICALM x age x education	64	0.03919967153197257	0.38759029167643716	0.88399177538805	0.9485554664635422	False
Women	0	CVLT_8	Model 3a APOE x PICALM	64	0.022702490270743225	0.46459732158802974	0.708061064353904	0.9022146030567919	False
Women	0	CVLT_8	Model 3b APOE x PICALM x age	64	0.03934590628393142	0.47510598859583	0.7933206987138097	0.9276818767163478	False
Women	0	CVLT_8	Model 3c APOE x PICALM x age x education	64	0.052970220083735975	0.38453939970914636	0.924355161959346	0.9546618885809639	False
Women	0	CVLT_9	Model 1a APOE	64	0.06410827067669167	4.246979279140312	0.04352150048102025	0.32962218890848494	False
Women	0	CVLT_9	Model 1b APOE x age	64	0.09978975359430009	2.2170321653799	0.09537721742839003	0.4534916753198922	False
Women	0	CVLT_9	Model 1c APOE x age x education	64	0.23961301654794454	2.520958635668732	0.02511293582700475	0.2751504273219651	False
Women	0	CVLT_9	Model 2a PICALM	64	0.010966712970108539	0.6874755511906023	0.41020521796466125	0.7924186384559482	False
Women	0	CVLT_9	Model 2b PICALM x age	64	0.0389467962845117	0.8105023974519007	0.49304094397424825	0.8208670527723201	False
Women	0	CVLT_9	Model 2c PICALM x age x education	64	0.05179891061328812	0.5189718260548687	0.7915353640110996	0.9276818767163478	False

Sex	Cluster	Outcome	Model	N	R2	F	F_pvalue	p_FDR	Significant_FDR
Women	0	CVLT_9	Model 3a APOE x PICALM	64	0.08840260908681963	1.9395099189185596	0.13283500431115208	0.5216042312623503	False
Women	0	CVLT_9	Model 3b APOE x PICALM x age	64	0.13800294891039622	1.8571226030727925	0.11599725338814368	0.5216042312623503	False
Women	0	CVLT_9	Model 3c APOE x PICALM x age x education	64	0.17448432207468012	1.4531277192435659	0.19588301274651912	0.5810027418406003	False
Women	0	RPM	Model 1a APOE	64	0.00932002992886205	0.5832780242321349	0.44792693869709976	0.8062684896547795	False
Women	0	RPM	Model 1b APOE x age	64	0.12069369892778092	2.745202639412637	0.05077281387008309	0.32962218890848494	False
Women	0	RPM	Model 1c APOE x age x education	64	0.2288264684798974	2.37380002427049	0.03377981756796084	0.32962218890848494	False
Women	0	RPM	Model 2a PICALM	64	3.8203550545290454e-06	0.0002368629182791826	0.9877701799492602	0.9917055193116079	False
Women	0	RPM	Model 2b PICALM x age	64	0.11746167863924539	2.661905456029046	0.056072231969579854	0.3446390843008323	False
Women	0	RPM	Model 2c PICALM x age x education	64	0.15598594874745286	1.7557367805685933	0.12478020886594303	0.5216042312623503	False
Women	0	RPM	Model 3a APOE x PICALM	64	0.0293348143027774	0.6044270410647592	0.614680705012944	0.8817651745440186	False
Women	0	RPM	Model 3b APOE x PICALM x age	64	0.16925863087515858	2.3634312568546045	0.05085779807889061	0.32962218890848494	False
Women	0	RPM	Model 3c APOE x PICALM x age x education	64	0.18569332810935968	1.5677651612355914	0.1559572661237392	0.5458504314330872	False
Women	1	CVLT_1	Model 1a APOE	32	0.02303175033921301	0.7072415203015007	0.4070145004618906	0.7924186384559482	False
Women	1	CVLT_1	Model 1b APOE x age	32	0.05144747603415578	0.5062201942996926	0.6811596902192516	0.9022146030567919	False
Women	1	CVLT_1	Model 1c APOE x age x education	32	0.0985861896435043	0.5687156999996237	0.7231600932337648	0.9022146030567919	False
Women	1	CVLT_1	Model 2a PICALM	32	0.02303175033921301	0.7072415203015007	0.4070145004618906	0.7924186384559482	False
Women	1	CVLT_1	Model 2b PICALM x age	32	0.06091405101688585	0.6054090612684597	0.6169931002527212	0.8817651745440186	False
Women	1	CVLT_1	Model 2c PICALM x age x education	32	0.09988801086797727	0.5770589246504267	0.7170542497074182	0.9022146030567919	False
Women	1	CVLT_1	Model 3a APOE x PICALM	32	0.08359192273924498	0.8513579247704772	0.47766140920956524	0.8208670527723201	False
Women	1	CVLT_1	Model 3b APOE x PICALM x age	32	0.10703980965759441	0.6233279111872387	0.6833152719920368	0.9022146030567919	False
Women	1	CVLT_1	Model 3c APOE x PICALM x age x education	32	0.14799263449992806	0.4993839741485864	0.8440629395177549	0.9324372000339155	False
Women	1	CVLT_10	Model 1a APOE	32	0.02939455656359924	0.9085429129532394	0.34811875796466607	0.7541224565461625	False
Women	1	CVLT_10	Model 1b APOE x age	32	0.12020400069117265	1.275186528845686	0.30203868453588156	0.7188789006482053	False

Sex	Cluster	Outcome	Model	N	R2	F	F_pvalue	p_FDR	Significant_FDR
Women	1	CVLT_10	Model 1c APOE x age x education	32	0.1260094088798479	0.7497208011534858	0.5937657393831473	0.8817651745440186	False
Women	1	CVLT_10	Model 2a PICALM	32	0.046221070172190126	1.4538296682817684	0.23733853048569464	0.6295716808673163	False
Women	1	CVLT_10	Model 2b PICALM x age	32	0.13697736523848125	1.4813694999387472	0.24101461681332867	0.6298754568348397	False
Women	1	CVLT_10	Model 2c PICALM x age x education	32	0.14164468171420885	0.8580972578871467	0.5220477011589625	0.8374587597448505	False
Women	1	CVLT_10	Model 3a APOE x PICALM	32	0.06886785671498741	0.6903065985946417	0.5656150676081331	0.8638484668924215	False
Women	1	CVLT_10	Model 3b APOE x PICALM x age	32	0.14098878479293964	0.853471605427836	0.5249920124036425	0.8374587597448505	False
Women	1	CVLT_10	Model 3c APOE x PICALM x age x education	32	0.1551414888137752	0.5279366597293932	0.8232846441957808	0.9276818767163478	False
Women	1	CVLT_11	Model 1a APOE	32	0.03540669856459344	1.1011904761904807	0.3023855693202768	0.7188789006482053	False
Women	1	CVLT_11	Model 1b APOE x age	32	0.05376786671974032	0.5303491659894212	0.6651653300460041	0.9022146030567919	False
Women	1	CVLT_11	Model 1c APOE x age x education	32	0.06853401282296123	0.38259783135985176	0.8560892505434787	0.9339155460474314	False
Women	1	CVLT_11	Model 2a PICALM	32	0.03540669856459344	1.1011904761904807	0.3023855693202768	0.7188789006482053	False
Women	1	CVLT_11	Model 2b PICALM x age	32	0.05964942836827902	0.5920430261853173	0.6253882849523353	0.8817651745440186	False
Women	1	CVLT_11	Model 2c PICALM x age x education	32	0.07162699596467104	0.4011969083518454	0.8434727993419755	0.9324372000339155	False
Women	1	CVLT_11	Model 3a APOE x PICALM	32	0.09604217220316291	0.9916321090047391	0.4110520240017924	0.7924186384559482	False
Women	1	CVLT_11	Model 3b APOE x PICALM x age	32	0.12297110980949977	0.7291091298833993	0.6079991640478303	0.8817651745440186	False
Women	1	CVLT_11	Model 3c APOE x PICALM x age x education	32	0.14397400654944192	0.48354287369376797	0.8552448797072634	0.9339155460474314	False
Women	1	CVLT_12	Model 1a APOE	32	0.009172320217096464	0.27771691498685797	0.6020770498759573	0.8817651745440186	False
Women	1	CVLT_12	Model 1b APOE x age	32	0.08292825332429499	0.8439874348243921	0.48141442298760884	0.8208670527723201	False
Women	1	CVLT_12	Model 1c APOE x age x education	32	0.5825472052457418	7.256498232479394	0.00022680957944517082	0.014289003505045762	True
Women	1	CVLT_12	Model 2a PICALM	32	0.10990502035278149	3.7042682926829262	0.06380320784381847	0.36541837219641493	False
Women	1	CVLT_12	Model 2b PICALM x age	32	0.17511457628827398	1.9813693696629888	0.13962233438417737	0.5216042312623503	False
Women	1	CVLT_12	Model 2c PICALM x age x education	32	0.6091012401134991	8.10267714717192	0.00010238947906394063	0.010128707708018804	True
Women	1	CVLT_12	Model 3a APOE x PICALM	32	0.10995610184372262	1.1530408249158262	0.3450910378160835	0.7541224565461625	False

Sex	Cluster	Outcome	Model	N	R2	F	F_pvalue	p_FDR	Significant_FDR
Women	1	CVLT_12	Model 3b APOE x PICALM x age	32	0.16596656716417924	1.034762055424244	0.41822094807397264	0.7924186384559482	False
Women	1	CVLT_12	Model 3c APOE x PICALM x age x education	32	0.6616270690982538	5.621542534706528	0.0005180100585993684	0.018648362109577262	True
Women	1	CVLT_13	Model 1a APOE	32	0.02933333333333321	0.9065934065934017	0.3486288531915386	0.7541224565461625	False
Women	1	CVLT_13	Model 1b APOE x age	32	0.08574498832148703	0.8753428173152015	0.4656263964499886	0.8208670527723201	False
Women	1	CVLT_13	Model 1c APOE x age x education	32	0.08776891472879755	0.5003100244649763	0.7731445383567357	0.9246743820217322	False
Women	1	CVLT_13	Model 2a PICALM	32	0.05454545454545434	1.7307692307692255	0.19827871348528422	0.5810027418406003	False
Women	1	CVLT_13	Model 2b PICALM x age	32	0.09681583638239999	1.0004764358168952	0.40715101970011075	0.7924186384559482	False
Women	1	CVLT_13	Model 2c PICALM x age x education	32	0.1000869752075827	0.5783361910996706	0.716119837400284	0.9022146030567919	False
Women	1	CVLT_13	Model 3a APOE x PICALM	32	0.07513725490196099	0.7582541836273208	0.5269806541479224	0.8374587597448505	False
Women	1	CVLT_13	Model 3b APOE x PICALM x age	32	0.11242433333333335	0.6586554310674737	0.6578067393501366	0.9009092299795349	False
Women	1	CVLT_13	Model 3c APOE x PICALM x age x education	32	0.11990263209212704	0.391684011150173	0.9138303660011812	0.9521669931716754	False
Women	1	CVLT_2	Model 1a APOE	32	0.020653398422831426	0.6326687116564417	0.43262669027550205	0.800362408716167	False
Women	1	CVLT_2	Model 1b APOE x age	32	0.06475954015841201	0.6462748360076542	0.5918372876536371	0.8817651745440186	False
Women	1	CVLT_2	Model 1c APOE x age x education	32	0.17030171346586387	1.0673384824280425	0.40096352463054313	0.7924186384559482	False
Women	1	CVLT_2	Model 2a PICALM	32	0.0003345509166012839	0.010039886351219565	0.9208527794740761	0.9546618885809639	False
Women	1	CVLT_2	Model 2b PICALM x age	32	0.049104545497732466	0.48197631941785357	0.6974600317262833	0.9022146030567919	False
Women	1	CVLT_2	Model 2c PICALM x age x education	32	0.13486903828594954	0.8106506761675033	0.5527595662468359	0.8493622603305039	False
Women	1	CVLT_2	Model 3a APOE x PICALM	32	0.3250955357734532	4.495784457478006	0.010709668681177042	0.17992243384377432	False
Women	1	CVLT_2	Model 3b APOE x PICALM x age	32	0.3811886991672373	3.203207880984271	0.021970645446043206	0.2636477453525185	False
Women	1	CVLT_2	Model 3c APOE x PICALM x age x education	32	0.4644191359821056	2.493003588537366	0.04128751844405061	0.32962218890848494	False
Women	1	CVLT_3	Model 1a APOE	32	0.06807187808021842	2.1913238739911516	0.1492163828348579	0.5437098121577538	False
Women	1	CVLT_3	Model 1b APOE x age	32	0.0913859548312912	0.9387215429484298	0.4351176587068051	0.800362408716167	False
Women	1	CVLT_3	Model 1c APOE x age x education	32	0.09964281204096959	0.5754856289730861	0.7182053652499434	0.9022146030567919	False

Sex	Cluster	Outcome	Model	N	R2	F	F_pvalue	p_FDR	Significant_FDR
Women	1	CVLT_3	Model 2a PICALM	32	0.03944195920843119	1.2318451629199283	0.2758660541645672	0.7022045015098075	False
Women	1	CVLT_3	Model 2b PICALM x age	32	0.06396344356343253	0.6377872058737555	0.596997803401627	0.8817651745440186	False
Women	1	CVLT_3	Model 2c PICALM x age x education	32	0.07098615417249354	0.39733315424181936	0.8461138346162584	0.9324372000339155	False
Women	1	CVLT_3	Model 3a APOE x PICALM	32	0.08629740469999514	0.8815148911798399	0.46257323969972153	0.8208670527723201	False
Women	1	CVLT_3	Model 3b APOE x PICALM x age	32	0.09059073410848939	0.5179976002359558	0.7602633522083755	0.9246743820217322	False
Women	1	CVLT_3	Model 3c APOE x PICALM x age x education	32	0.12973736644399747	0.42860041801678694	0.8917152747752985	0.9485554664635422	False
Women	1	CVLT_4	Model 1a APOE	32	0.01564881399592133	0.47692777390090285	0.4951261588150502	0.8208670527723201	False
Women	1	CVLT_4	Model 1b APOE x age	32	0.08244911495036367	0.8386729122150346	0.48413657226219176	0.8208670527723201	False
Women	1	CVLT_4	Model 1c APOE x age x education	32	0.09397002534877019	0.5393244655086661	0.7446773327233658	0.9244270337255575	False
Women	1	CVLT_4	Model 2a PICALM	32	0.0005366534292154945	0.016108247422681878	0.8998519570986859	0.9487978794513342	False
Women	1	CVLT_4	Model 2b PICALM x age	32	0.05941551537881884	0.5895746945322529	0.6269475175706127	0.8817651745440186	False
Women	1	CVLT_4	Model 2c PICALM x age x education	32	0.0706889930090222	0.39554332282915067	0.8473338047927248	0.9324372000339155	False
Women	1	CVLT_4	Model 3a APOE x PICALM	32	0.02148760330578514	0.20495495495495547	0.892093831554998	0.9485554664635422	False
Women	1	CVLT_4	Model 3b APOE x PICALM x age	32	0.059270153019885696	0.3276230648924234	0.8916824314687498	0.9485554664635422	False
Women	1	CVLT_4	Model 3c APOE x PICALM x age x education	32	0.1119568455537151	0.36245528086709694	0.9297504247472769	0.9563147225971991	False
Women	1	CVLT_5	Model 1a APOE	32	0.09212650522694188	3.0442513992537292	0.0912614744027732	0.4509390499901734	False
Women	1	CVLT_5	Model 1b APOE x age	32	0.20871661537894837	2.4618509391167946	0.08327343056647149	0.41969809005501635	False
Women	1	CVLT_5	Model 1c APOE x age x education	32	0.2666056487911187	1.8903191324403386	0.13050810632108795	0.5216042312623503	False
Women	1	CVLT_5	Model 2a PICALM	32	0.04893476247188022	1.5435774710596564	0.22371045253692137	0.6078249134995694	False
Women	1	CVLT_5	Model 2b PICALM x age	32	0.18289795108861218	2.089148528960349	0.12423446615363211	0.5216042312623503	False
Women	1	CVLT_5	Model 2c PICALM x age x education	32	0.23824273646706406	1.626321518593788	0.18825139079023442	0.5810027418406003	False
Women	1	CVLT_5	Model 3a APOE x PICALM	32	0.11636270228615453	1.2290697674418596	0.3176429448806	0.739007714702236	False
Women	1	CVLT_5	Model 3b APOE x PICALM x age	32	0.22775838970231466	1.5336438889837767	0.21395847278459434	0.6058150015923345	False

Sex	Cluster	Outcome	Model	N	R2	F	F_pvalue	p_FDR	Significant_FDR
Women	1	CVLT_5	Model 3c APOE x PICALM x age x education	32	0.28661835752363285	1.1551009008586073	0.36645250887542935	0.7695502686384017	False
Women	1	CVLT_6	Model 1a APOE	32	0.13129142538198557	4.534020817270623	0.04154920007565369	0.32962218890848494	False
Women	1	CVLT_6	Model 1b APOE x age	32	0.22079127544901544	2.6446297454445955	0.06860511506933487	0.37583671733635626	False
Women	1	CVLT_6	Model 1c APOE x age x education	32	0.32981764284486637	2.5590822027508326	0.05191916246298379	0.32962218890848494	False
Women	1	CVLT_6	Model 2a PICALM	32	0.05895485941533518	1.879448398576511	0.1805666541085458	0.578151615606707	False
Women	1	CVLT_6	Model 2b PICALM x age	32	0.18503435618068953	2.1190921819227024	0.12027824403603157	0.5216042312623503	False
Women	1	CVLT_6	Model 2c PICALM x age x education	32	0.3016899709864449	2.246549217323443	0.0796728844620492	0.4097462629476816	False
Women	1	CVLT_6	Model 3a APOE x PICALM	32	0.15691390907859692	1.737105894448253	0.18217812744817477	0.578151615606707	False
Women	1	CVLT_6	Model 3b APOE x PICALM x age	32	0.2330789460220004	1.5803589079054943	0.2006027436646085	0.5810562230285212	False
Women	1	CVLT_6	Model 3c APOE x PICALM x age x education	32	0.3457632101517877	1.519433398750047	0.20480128489565522	0.586476406746649	False
Women	1	CVLT_7	Model 1a APOE	32	0.07509492964038422	2.435761205564142	0.12908471571654023	0.5216042312623503	False
Women	1	CVLT_7	Model 1b APOE x age	32	0.28727086442466787	3.7618705351029442	0.021843871291629645	0.2636477453525185	False
Women	1	CVLT_7	Model 1c APOE x age x education	32	0.3034574516129115	2.2654448777624596	0.07762431950051915	0.40752767737772555	False
Women	1	CVLT_7	Model 2a PICALM	32	0.10725932544114358	3.6043835068054415	0.06728422801904672	0.37583671733635626	False
Women	1	CVLT_7	Model 2b PICALM x age	32	0.36254877932139506	5.308309870985017	0.005039671009092421	0.1084409562945144	False
Women	1	CVLT_7	Model 2c PICALM x age x education	32	0.37777012567577517	3.1570400820877986	0.023340281636844172	0.26735231693112416	False
Women	1	CVLT_7	Model 3a APOE x PICALM	32	0.14438502673796794	1.5749999999999995	0.21752337659980545	0.6078249134995694	False
Women	1	CVLT_7	Model 3b APOE x PICALM x age	32	0.31959783687430754	2.4425389009814045	0.060866531996936236	0.3567104626905254	False
Women	1	CVLT_7	Model 3c APOE x PICALM x age x education	32	0.33666488334775	1.4591592022290054	0.22598576092451048	0.6078249134995694	False
Women	1	CVLT_8	Model 1a APOE	32	0.09090909090909105	3.0000000000000036	0.09353647034332341	0.4532921255099519	False
Women	1	CVLT_8	Model 1b APOE x age	32	0.16401673640167336	1.8311644978311599	0.16440599601417727	0.5524041466076356	False
Women	1	CVLT_8	Model 1c APOE x age x education	32	0.16672588540535016	1.0404434614287341	0.41516860159288876	0.7924186384559482	False
Women	1	CVLT_8	Model 2a PICALM	32	0.05818181818181822	1.853281853281854	0.18354019543070063	0.578151615606707	False

Sex	Cluster	Outcome	Model	N	R2	F	F_pvalue	p_FDR	Significant_FDR
Women	1	CVLT_8	Model 2b PICALM x age	32	0.1582383997981308	1.7545249523083932	0.1787448692960024	0.578151615606707	False
Women	1	CVLT_8	Model 2c PICALM x age x education	32	0.1604751556599482	0.9939798864293355	0.4406605104419634	0.8046844103722809	False
Women	1	CVLT_8	Model 3a APOE x PICALM	32	0.118235294117647	1.2515010006671106	0.3099572845494671	0.7299928570697729	False
Women	1	CVLT_8	Model 3b APOE x PICALM x age	32	0.16316735294117668	1.0139067091564815	0.4295800984856457	0.800362408716167	False
Women	1	CVLT_8	Model 3c APOE x PICALM x age x education	32	0.18590304218705145	0.6565203826871148	0.7232037691169523	0.9022146030567919	False
Women	1	CVLT_9	Model 1a APOE	32	0.02994148306846156	0.925969388831458	0.34360508536294765	0.7541224565461625	False
Women	1	CVLT_9	Model 1b APOE x age	32	0.14231899584143304	1.5487233848166226	0.22387357768307098	0.6078249134995694	False
Women	1	CVLT_9	Model 1c APOE x age x education	32	0.43871075302873375	4.064385569578176	0.007371424033699089	0.13268563260658361	False
Women	1	CVLT_9	Model 2a PICALM	32	0.0793029130559908	2.5840066460690387	0.11842352420732201	0.5216042312623503	False
Women	1	CVLT_9	Model 2b PICALM x age	32	0.16830333704541844	1.888706803483978	0.15441745282650426	0.5458504314330872	False
Women	1	CVLT_9	Model 2c PICALM x age x education	32	0.4526664419287283	4.30060511239999	0.00552972114434847	0.1084409562945144	False
Women	1	CVLT_9	Model 3a APOE x PICALM	32	0.10346237404564362	1.0770867797077477	0.374776283435752	0.774128060867291	False
Women	1	CVLT_9	Model 3b APOE x PICALM x age	32	0.18077056023394233	1.14742814111384	0.36103249396673104	0.7645393989883716	False
Women	1	CVLT_9	Model 3c APOE x PICALM x age x education	32	0.45445919103185073	2.3949998840377367	0.048386168941549235	0.32962218890848494	False
Women	1	RPM	Model 1a APOE	32	0.0001329091774180391	0.003987805338467842	0.950066683688123	0.9616127543964292	False
Women	1	RPM	Model 1b APOE x age	32	0.38640854666731994	5.877656459043942	0.003034987988947951	0.08497966369054263	False
Women	1	RPM	Model 1c APOE x age x education	32	0.6963675637106174	11.925970016734448	4.5899723128648e-06	0.0011566730228419297	True
Women	1	RPM	Model 2a PICALM	32	0.0001329091774180391	0.003987805338467842	0.950066683688123	0.9616127543964292	False
Women	1	RPM	Model 2b PICALM x age	32	0.30022952959705496	4.004373426368844	0.017201191316622413	0.2636477453525185	False
Women	1	RPM	Model 2c PICALM x age x education	32	0.5530631642719226	6.434753692944102	0.0005143436044788799	0.018648362109577262	True
Women	1	RPM	Model 3a APOE x PICALM	32	0.001593290977881634	0.014894446981558127	0.9974592161470038	0.9974592161470038	False
Women	1	RPM	Model 3b APOE x PICALM x age	32	0.3529975938643116	2.8370643921677536	0.03567313835622828	0.32962218890848494	False
Women	1	RPM	Model 3c APOE x PICALM x age x education	32	0.7063554351936847	6.915748219352596	0.00012057985366689053	0.010128707708018804	True

Supplementary Table 2: All Regression Coefficients

Sex	Cluster	Outcome	Model	Predictor	Beta	SE	CI_low	CI_high	p	N
Men	0	CVLT_1	Model 1a APOE	APOE	1.0428571428571622	2.695431962232146	-4.354652259381692	6.440366545096016	0.7002724761633933	59
Men	0	CVLT_1	Model 1b APOE x age	APOE	0.9990490623793313	2.7395867397448956	-4.491205451774546	6.489303576533208	0.7167551226030685	59
Men	0	CVLT_1	Model 1b APOE x age	APOE_age	-0.0640391076029907	0.8901654430571652	-1.8479705200259737	1.7198923048199923	0.9429100889583418	59
Men	0	CVLT_1	Model 1b APOE x age	age_c	0.21726491405460013	0.47708541275239824	-0.7388356185551925	1.1733654466643928	0.650613956025329	59
Men	0	CVLT_1	Model 1c APOE x age x education	APOE	1.1960937563380547	2.6792101420808154	-4.182645042168975	6.574832554845084	0.657172795792478	59
Men	0	CVLT_1	Model 1c APOE x age x education	APOE_age	0.1897784440543706	0.8762685590676512	-1.5694040935678895	1.9489609816766307	0.8294030537554439	59
Men	0	CVLT_1	Model 1c APOE x age x education	APOE_age_education	-2.2510490151205227	1.5190463901071234	-5.300661894256441	0.7985638640153963	0.14452425767424118	59
Men	0	CVLT_1	Model 1c APOE x age x education	APOE_education	1.824767403932599	4.055835414743628	-6.3176619497789055	9.967196757644103	0.6546807550994609	59
Men	0	CVLT_1	Model 1c APOE x age x education	age_c	0.1605977529360625	0.4670456308967913	-0.777035475649256	1.098230981521381	0.7323661663081442	59
Men	0	CVLT_1	Model 1c APOE x age x education	age_education	0.49675037258659627	0.7995191391382565	-1.108351275204256	2.1018520203774482	0.5371620672600287	59
Men	0	CVLT_1	Model 1c APOE x age x education	education_c	3.7083616079583805	2.244874557731274	-0.7984121205378649	8.215135336454626	0.10469478086862413	59
Men	0	CVLT_1	Model 2a PICALM	PICALM	1.029761904761905	2.333392659254673	-3.6427762983587675	5.702300107882578	0.6606543092715944	59
Men	0	CVLT_1	Model 2b PICALM x age	PICALM	1.0686789132446446	2.377351877937173	-3.6956407157780014	5.8329985422672905	0.6548196130119373	59
Men	0	CVLT_1	Model 2b PICALM x age	PICALM_age	-0.73575480338378	0.8111152299055546	-2.361266048522383	0.8897564417548229	0.3683162202277295	59
Men	0	CVLT_1	Model 2b PICALM x age	age_c	0.5558482613277107	0.5387094846455709	-0.5237496710846299	1.6354461937400513	0.3066750429101951	59
Men	0	CVLT_1	Model 2c PICALM x age x education	PICALM	1.2570819987642956	2.351762467327032	-3.4620746425614852	5.976238640090076	0.5952547551048459	59
Men	0	CVLT_1	Model 2c PICALM x age x education	PICALM_age	-0.841664669749641	0.8015491101190994	-2.4500906306701706	0.7667612911708888	0.2985519712429915	59
Men	0	CVLT_1	Model 2c PICALM x age x education	PICALM_education	0.5706125333031302	3.958238114554194	-7.372173332940442	8.513398399546702	0.8859327888432007	59
Men	0	CVLT_1	Model 2c PICALM x age x education	age_c	0.6013549605475812	0.5360579462259052	-0.4743240045745616	1.677033925669724	0.2670969700899716	59

Sex	Cluster	Outcome	Model	Predictor	Beta	SE	CI_low	CI_high	p	N
Men	0	CVLT_1	Model 2c PICALM x age x education	age_education	-0.1691882581044556	0.7222752825907147	-1.6185396462901411	1.2801631300812297	0.8157158284247763	59
Men	0	CVLT_1	Model 2c PICALM x age x education	education_c	3.7870014720595897	2.6437356020646465	-1.5180421272512712	9.09204507137045	0.15800138644281078	59
Men	0	CVLT_1	Model 3a APOE x PICALM	APOE	6.607142857142847	3.6148874194546137	-0.637253417992734	13.851539132278429	0.07301203734610297	59
Men	0	CVLT_1	Model 3a APOE x PICALM	APOE_PICALM	-12.094537815126053	5.275025955904862	-22.665926063772034	-1.5231495664800736	0.02570839764728862	59
Men	0	CVLT_1	Model 3a APOE x PICALM	PICALM	3.951680672268889	2.630216939140203	-1.3193918635337791	9.222753208071557	0.13870987630846904	59
Men	0	CVLT_1	Model 3b APOE x PICALM x age	APOE	6.607142857142861	3.6500113577926934	-0.71385280661457	13.928138520900292	0.07594024654293355	59
Men	0	CVLT_1	Model 3b APOE x PICALM x age	APOE_PICALM	-12.405594776670325	5.336341305544446	-23.108939979915426	-1.7022495734252256	0.02394870339858856	59
Men	0	CVLT_1	Model 3b APOE x PICALM x age	APOE_PICALM_age	-0.6897834274952928	1.0094342417727151	-2.7144521154676453	1.3348852604770598	0.4973706820481405	59
Men	0	CVLT_1	Model 3b APOE x PICALM x age	PICALM	4.34920634920633	2.690715350541716	-1.0476851896830324	9.746097888095694	0.11195072478686686	59
Men	0	CVLT_1	Model 3b APOE x PICALM x age	age_c	0.40089453860639246	0.435886193791266	-0.47338244900478765	1.2751715262175725	0.36188474632773304	59
Men	0	CVLT_1	Model 3c APOE x PICALM x age x education	APOE	7.128428234118919	3.8065647677934633	-0.5211500617674387	14.778006530005278	0.0670872011801105	59
Men	0	CVLT_1	Model 3c APOE x PICALM x age x education	APOE_PICALM	-12.73083545427908	5.707513405620795	-24.200513059797803	-1.2611578487603552	0.030322797444014754	59
Men	0	CVLT_1	Model 3c APOE x PICALM x age x education	APOE_PICALM_age	-1.07452348689905	1.1170462583147711	-3.3193119863362863	1.1702650125381862	0.34080647159354793	59
Men	0	CVLT_1	Model 3c APOE x PICALM x age x education	APOE_PICALM_education	4.6045852389110085	6.844137649584968	-9.14922430119886	18.358394779020877	0.504249139593469	59
Men	0	CVLT_1	Model 3c APOE x PICALM x age x education	APOE_age_education	-0.4785166519444566	2.0120576861778487	-4.521897953763016	3.564864649874103	0.8130088758964032	59
Men	0	CVLT_1	Model 3c APOE x PICALM x age x education	PICALM	4.6774780557678	2.628242144866089	-0.6041722757345322	9.959128387270132	0.08132735969576567	59
Men	0	CVLT_1	Model 3c APOE x PICALM x age x education	age_c	0.4078789345170195	0.42570377393690445	-0.44760482793904754	1.2633626969730867	0.3427017670794834	59
Men	0	CVLT_1	Model 3c APOE x PICALM x age x education	age_education	0.5481080521602721	0.7719619596356383	-1.0032075859292682	2.0994236902498127	0.481057545187059	59
Men	0	CVLT_1	Model 3c APOE x PICALM x age x education	education_c	3.6272735196688854	2.1384808288079222	-0.6701645989791283	7.924711638316899	0.09619487984415692	59
Men	0	CVLT_10	Model 1a APOE	APOE	0.03333333333333388	0.5388111545963579	-1.0456173928265797	1.1122840594932475	0.9508870839525561	59
Men	0	CVLT_10	Model 1b APOE x age	APOE	-0.008046998794694282	0.5264730842953725	-1.0631226369189823	1.0470286393295938	0.9878603364538636	59
Men	0	CVLT_10	Model 1b APOE x age	APOE_age	0.13497260184611354	0.1710652703710714	-0.20784986084297935	0.4777950645352064	0.4334929186031933	59
Men	0	CVLT_10	Model 1b APOE x age	age_c	0.11905965621840248	0.09168267063063483	-0.06467652157693823	0.3027958340137432	0.19949775588793411	59

Sex	Cluster	Outcome	Model	Predictor	Beta	SE	CI_low	CI_high	p	N
Men	0	CVLT_10	Model 1c APOE x age x education	APOE	-0.06550471779833661	0.5358001314149127	-1.1411683657600074	1.0101589301633342	0.9031768500288216	59
Men	0	CVLT_10	Model 1c APOE x age x education	APOE_age	0.12706524001775535	0.175240008885067	-0.22474375772990804	0.4788742377654187	0.4717091516905235	59
Men	0	CVLT_10	Model 1c APOE x age x education	APOE_age_education	0.0871602840784112	0.3037855234500657	-0.5227146024568414	0.6970351706136639	0.775340731582545	59
Men	0	CVLT_10	Model 1c APOE x age x education	APOE_education	0.30323965398348407	0.8111036585316656	-1.3251188869284853	1.9315981948954535	0.7100578533796147	59
Men	0	CVLT_10	Model 1c APOE x age x education	age_c	0.13146026320679052	0.09340182260466871	-0.05605171997226116	0.3189722463858422	0.1653539515409003	59
Men	0	CVLT_10	Model 1c APOE x age x education	age_education	-0.1685436066382044	0.15989132509266754	-0.4895388359085369	0.15245162263212814	0.29679942539355914	59
Men	0	CVLT_10	Model 1c APOE x age x education	education_c	-0.5081031557348104	0.44893980660593114	-1.4093874253256078	0.3931811138559871	0.2630186656228463	59
Men	0	CVLT_10	Model 2a PICALM	PICALM	0.3940476190476189	0.4637117835168901	-0.5345192105111825	1.3226144486064202	0.3990090830961004	59
Men	0	CVLT_10	Model 2b PICALM x age	PICALM	0.5634135244724288	0.4519506440666052	-0.34231580607342094	1.4691428550182786	0.2178178141354443	59
Men	0	CVLT_10	Model 2b PICALM x age	PICALM_age	0.18537713087066587	0.15419848192019966	-0.12364353241261594	0.4943977941539477	0.23443726684829994	59
Men	0	CVLT_10	Model 2b PICALM x age	age_c	0.08798735511064264	0.10241231044081434	-0.11725150137285988	0.29322621159414514	0.3939879075331235	59
Men	0	CVLT_10	Model 2c PICALM x age x education	PICALM	0.6078818581770962	0.4534711833537871	-0.30207464308732357	1.517838359441516	0.1859075731271588	59
Men	0	CVLT_10	Model 2c PICALM x age x education	PICALM_age	0.17941199393079957	0.15455618011244424	-0.13072767109437547	0.48955165895597463	0.2510162906747408	59
Men	0	CVLT_10	Model 2c PICALM x age x education	PICALM_education	-0.7329082307533574	0.7632347852897983	-2.264450874167075	0.7986344126603597	0.3413654430133576	59
Men	0	CVLT_10	Model 2c PICALM x age x education	age_c	0.10588749233228544	0.10336368344952364	-0.10152691282110883	0.3133018974856797	0.3103805905817456	59
Men	0	CVLT_10	Model 2c PICALM x age x education	age_education	-0.1771172468618266	0.13927045424611606	-0.4565838589142853	0.10234936519063217	0.20912038539096117	59
Men	0	CVLT_10	Model 2c PICALM x age x education	education_c	-0.06731080139625867	0.5097699825549946	-1.090239108206591	0.9556175054140736	0.895461049279848	59
Men	0	CVLT_10	Model 3a APOE x PICALM	APOE	-0.03571428571428593	0.7526871719565142	-1.5441330861223987	1.4727045146938267	0.9623272207788581	59
Men	0	CVLT_10	Model 3a APOE x PICALM	APOE_PICALM	0.044117647058823595	1.0983590657288766	-2.157043108793472	2.2452784029111186	0.9681055426108653	59
Men	0	CVLT_10	Model 3a APOE x PICALM	PICALM	0.3844537815126048	0.5476603611219087	-0.7130821082080063	1.481989671233216	0.48564370785888666	59
Men	0	CVLT_10	Model 3b APOE x PICALM x age	APOE	-0.0357142857142852	0.7254451130729375	-1.490772916083248	1.4193443446546776	0.9609203807706753	59
Men	0	CVLT_10	Model 3b APOE x PICALM x age	APOE_PICALM	-0.03474571294338746	1.060605664563619	-2.162051277253313	2.092559851366538	0.9739888372537482	59
Men	0	CVLT_10	Model 3b APOE x PICALM x age	APOE_PICALM_age	0.24104605375791827	0.20062653670901084	-0.16135981880067363	0.6434519263165102	0.23491178018823053	59

Sex	Cluster	Outcome	Model	Predictor	Beta	SE	CI_low	CI_high	p	N
Men	0	CVLT_10	Model 3b APOE x PICALM x age	PICALM	0.5090187590187578	0.53478362404364	-0.5636213532683498	1.5816588713058655	0.3455080408165333	59
Men	0	CVLT_10	Model 3b APOE x PICALM x age	age_c	0.12562061290874837	0.08663302059779424	-0.04814322121756784	0.2993844470350646	0.15294205258066704	59
Men	0	CVLT_10	Model 3c APOE x PICALM x age x education	APOE	-0.2613406194046937	0.7844350252035529	-1.8377218211906046	1.3150405823812172	0.7404365678416311	59
Men	0	CVLT_10	Model 3c APOE x PICALM x age x education	APOE_PICALM	0.2943204350046107	1.1761716128064283	-2.069284912705548	2.657925782714769	0.8034510996687874	59
Men	0	CVLT_10	Model 3c APOE x PICALM x age x education	APOE_PICALM_age	0.2922544081491923	0.2301944832801609	-0.17033872517437987	0.7548475414727644	0.21022469330572585	59
Men	0	CVLT_10	Model 3c APOE x PICALM x age x education	APOE_PICALM_education	0.036528202149631955	1.4104006150303523	-2.797777948247214	2.8708343525464777	0.9794429545616901	59
Men	0	CVLT_10	Model 3c APOE x PICALM x age x education	APOE_age_education	-0.03971098887163023	0.41463330274105037	-0.8729478065491564	0.7935258288058958	0.9240908859149453	59
Men	0	CVLT_10	Model 3c APOE x PICALM x age x education	PICALM	0.45109399860670796	0.5416130603090092	-0.6373181954960625	1.5395061927094784	0.4089585661918299	59
Men	0	CVLT_10	Model 3c APOE x PICALM x age x education	age_c	0.12669924746680478	0.08772659103630993	-0.04959393751752758	0.30299243245113716	0.15503248582305304	59
Men	0	CVLT_10	Model 3c APOE x PICALM x age x education	age_education	-0.15815076148284757	0.15908149110884173	-0.47783698670077124	0.1615354637350761	0.3250343285889885	59
Men	0	CVLT_10	Model 3c APOE x PICALM x age x education	education_c	-0.4520229659544094	0.440685858555783	-1.3376143546611496	0.4335684227523309	0.31005792045277786	59
Men	0	CVLT_11	Model 1a APOE	APOE	0.48253968253968293	0.7009820842123877	-0.9211527286774434	1.8862320937568091	0.49400785062792074	59
Men	0	CVLT_11	Model 1b APOE x age	APOE	0.4323214291470987	0.6783953461677364	-0.9272132253479879	1.7918560836421853	0.5265925537599067	59
Men	0	CVLT_11	Model 1b APOE x age	APOE_age	0.41831211879056707	0.22042890087340486	-0.02343727009094021	0.8600615076720743	0.06298680646534499	59
Men	0	CVLT_11	Model 1b APOE x age	age_c	0.03229271991911012	0.11813917735850861	-0.20446348216828025	0.2690489220065005	0.7856120823370043	59
Men	0	CVLT_11	Model 1c APOE x age x education	APOE	0.5095164071298004	0.6817161995326505	-0.8590859710133407	1.8781187852729415	0.4582529302513373	59
Men	0	CVLT_11	Model 1c APOE x age x education	APOE_age	0.36890834122615107	0.22296364979926692	-0.07870986348124087	0.816526545933543	0.10415465945482169	59
Men	0	CVLT_11	Model 1c APOE x age x education	APOE_age_education	0.6860166398481676	0.3865163526043355	-0.0899473166019692	1.4619805962983046	0.08188744331690138	59
Men	0	CVLT_11	Model 1c APOE x age x education	APOE_education	-0.46094585203100913	1.0319939677152643	-2.5327601926800183	1.6108684886180002	0.6570147191934788	59
Men	0	CVLT_11	Model 1c APOE x age x education	age_c	0.0341110060476426	0.11883822306525356	-0.2044666818713335	0.27268869396661866	0.7752467446763779	59
Men	0	CVLT_11	Model 1c APOE x age x education	age_education	-0.10902892168397915	0.20343501259055324	-0.5174417512747715	0.2993839079068133	0.5943299911997126	59
Men	0	CVLT_11	Model 1c APOE x age x education	education_c	0.35301226714803907	0.5712009401157089	-0.7937214698174058	1.499746004113484	0.5393138875975378	59
Men	0	CVLT_11	Model 2a PICALM	PICALM	0.49999999999999994	0.6059782924279561	-0.7134505996671272	1.7134505996671272	0.4127478571295703	59

Sex	Cluster	Outcome	Model	Predictor	Beta	SE	CI_low	CI_high	p	N
Men	0	CVLT_11	Model 2b PICALM x age	PICALM	0.7023142790464059	0.5924827072474975	-0.48504759960197186	1.8896761576947836	0.24096550804942976	59
Men	0	CVLT_11	Model 2b PICALM x age	PICALM_age	0.3580946003171073	0.20214582105578377	-0.047014677833437324	0.763203878467652	0.08202328830195077	59
Men	0	CVLT_11	Model 2b PICALM x age	age_c	0.011064278187565724	0.13425696752962835	-0.25799269721040624	0.2801212535855377	0.9346189221701737	59
Men	0	CVLT_11	Model 2c PICALM x age x education	PICALM	0.7315632320078476	0.6072018321130609	-0.4868763844294295	1.9500028484451246	0.23373119969843378	59
Men	0	CVLT_11	Model 2c PICALM x age x education	PICALM_age	0.3610443801769342	0.20695206040348407	-0.05423531063265058	0.7763240709865189	0.08696629598489043	59
Men	0	CVLT_11	Model 2c PICALM x age x education	PICALM_education	-0.7150880914669162	1.0219779711973924	-2.7658369222135892	1.335660739279757	0.48722701385683387	59
Men	0	CVLT_11	Model 2c PICALM x age x education	age_c	0.010065312867767712	0.13840486511254094	-0.2676643675153064	0.2877949932508419	0.9423050053656141	59
Men	0	CVLT_11	Model 2c PICALM x age x education	age_education	0.049502676985124214	0.18648434141290102	-0.3247055309051066	0.4237108848753551	0.7917106980011437	59
Men	0	CVLT_11	Model 2c PICALM x age x education	education_c	0.5616356099550031	0.6825864106168473	-0.808074230087819	1.931345449997825	0.4143761911197973	59
Men	0	CVLT_11	Model 3a APOE x PICALM	APOE	-0.7142857142857145	0.9544350143726303	-2.6270162258276852	1.1984447972562564	0.4574158679658894	59
Men	0	CVLT_11	Model 3a APOE x PICALM	APOE_PICALM	2.4285714285714275	1.3927596878797535	-0.3625813582994115	5.219724215442266	0.08679678199903727	59
Men	0	CVLT_11	Model 3a APOE x PICALM	PICALM	-0.14285714285714285	0.6944534783022817	-1.5345730132858324	1.2488587275715468	0.8377757222105372	59
Men	0	CVLT_11	Model 3b APOE x PICALM x age	APOE	-0.7142857142857121	0.8892393540638976	-2.4978739875784233	1.0693025590069989	0.4254153212436904	59
Men	0	CVLT_11	Model 3b APOE x PICALM x age	APOE_PICALM	2.434696634115235	1.3000739533250387	-0.17292149138353086	5.042314759614001	0.06662513063938764	59
Men	0	CVLT_11	Model 3b APOE x PICALM x age	APOE_PICALM_age	0.6805512754665289	0.2459248931397247	0.1872884059025513	1.1738141450305066	0.007766762725295828	59
Men	0	CVLT_11	Model 3b APOE x PICALM x age	PICALM	-0.11255411255411257	0.6555294616213152	-1.427379705013944	1.202271479905719	0.8643276528290137	59
Men	0	CVLT_11	Model 3b APOE x PICALM x age	age_c	0.030559835644581493	0.10619341131220965	-0.18243717382402624	0.24355684511318926	0.774641095241782	59
Men	0	CVLT_11	Model 3c APOE x PICALM x age x education	APOE	-0.6860151466240412	0.9742946909157953	-2.64393363115491	1.2719033379068276	0.4846943456096564	59
Men	0	CVLT_11	Model 3c APOE x PICALM x age x education	APOE_PICALM	2.3889471480947324	1.4608447113459937	-0.5467302091173885	5.324624505306853	0.10839085152520969	59
Men	0	CVLT_11	Model 3c APOE x PICALM x age x education	APOE_PICALM_age	0.7179898697901993	0.28590929233401824	0.14343363585061153	1.2925461037297872	0.015377286867361636	59
Men	0	CVLT_11	Model 3c APOE x PICALM x age x education	APOE_PICALM_education	-1.3643684197400785	1.751765011935651	-4.88467200899537	2.1559351695152125	0.43981124957061946	59
Men	0	CVLT_11	Model 3c APOE x PICALM x age x education	APOE_age_education	0.12256613076255432	0.5149885109128823	-0.9123420281740353	1.157474289699144	0.8128752375302783	59
Men	0	CVLT_11	Model 3c APOE x PICALM x age x education	PICALM	-0.11071500372430165	0.6727016416086135	-1.4625595646771599	1.2411295572285566	0.8699495784951139	59

Sex	Cluster	Outcome	Model	Predictor	Beta	SE	CI_low	CI_high	p	N
Men	0	CVLT_11	Model 3c APOE x PICALM x age x education	age_c	0.03703563856301778	0.10895937732591551	-0.1819264279641632	0.25599770509019876	0.735382200854626	59
Men	0	CVLT_11	Model 3c APOE x PICALM x age x education	age_education	-0.11191362935707296	0.19758456370569874	-0.5089746758190299	0.28514741710488395	0.5737012148963175	59
Men	0	CVLT_11	Model 3c APOE x PICALM x age x education	education_c	0.33532570458498595	0.5473466616832344	-0.7646085928589976	1.4352600020289696	0.5429482623557824	59
Men	0	CVLT_12	Model 1a APOE	APOE	0.2777777777777799	0.3786780597410374	-0.48051195704498395	1.0360675126005439	0.46623195968316633	59
Men	0	CVLT_12	Model 1b APOE x age	APOE	0.28443586147737343	0.38410104259466904	-0.48531982919039507	1.054191552145142	0.46213213783838103	59
Men	0	CVLT_12	Model 1b APOE x age	APOE_age	0.03876002968133263	0.1248047633607107	-0.21135430526133597	0.28887436462400123	0.7573050010654495	59
Men	0	CVLT_12	Model 1b APOE x age	age_c	-0.045816481294236544	0.06688928727329434	-0.17986560851221112	0.08823264592373803	0.4962458515768785	59
Men	0	CVLT_12	Model 1c APOE x age x education	APOE	0.29713684844035493	0.39529367929235404	-0.49644832661540805	1.090722023496118	0.45569475383002966	59
Men	0	CVLT_12	Model 1c APOE x age x education	APOE_age	0.0422562014405683	0.12928564927461858	-0.21729556977790127	0.30180797265903786	0.7451237689533876	59
Men	0	CVLT_12	Model 1c APOE x age x education	APOE_age_education	-0.09095690506420917	0.22412181378757295	-0.5409002209978886	0.35898641086947025	0.686560165500835	59
Men	0	CVLT_12	Model 1c APOE x age x education	APOE_education	-0.5164706830440084	0.5984025211449351	-1.7178138726106589	0.684872506522642	0.3921328927554537	59
Men	0	CVLT_12	Model 1c APOE x age x education	age_c	-0.05180769645717698	0.06890843795150225	-0.1901471581264287	0.08653176521207476	0.45560679387104475	59
Men	0	CVLT_12	Model 1c APOE x age x education	age_education	0.08546239956970594	0.11796195349169585	-0.15135610377497408	0.32228090291438594	0.47207497581292523	59
Men	0	CVLT_12	Model 1c APOE x age x education	education_c	0.22503827823951017	0.3312113184172264	-0.4398961891598241	0.8899727456388444	0.49993126367990026	59
Men	0	CVLT_12	Model 2a PICALM	PICALM	0.3571428571428572	0.32607566169894503	-0.2958123925247398	1.0100981068104542	0.2780021968686483	59
Men	0	CVLT_12	Model 2b PICALM x age	PICALM	0.315075220739351	0.3320712891915788	-0.3504105140451338	0.9805609555238358	0.34686377805288804	59
Men	0	CVLT_12	Model 2b PICALM x age	PICALM_age	-0.11302108466363797	0.11329752342399331	-0.34007439544107154	0.11403222611379563	0.322861478141785	59
Men	0	CVLT_12	Model 2b PICALM x age	age_c	0.024236037934667665	0.07524757051160995	-0.12656346320430642	0.17503553907364175	0.7486101160305141	59
Men	0	CVLT_12	Model 2c PICALM x age x education	PICALM	0.32199078387653834	0.3425772940607048	-0.3654408487370533	1.00942241649013	0.3516110478845459	59
Men	0	CVLT_12	Model 2c PICALM x age x education	PICALM_age	-0.11135606186537589	0.11676031445193677	-0.3456527738183528	0.12294065008760105	0.3446404664628836	59
Men	0	CVLT_12	Model 2c PICALM x age x education	PICALM_education	-0.20697409587415194	0.5765899070891833	-1.3639863907654772	0.9500381990171733	0.7210766746876434	59
Men	0	CVLT_12	Model 2c PICALM x age x education	age_c	0.023176694241839102	0.0780866619095816	-0.13351565639695503	0.17986904488063324	0.7677959548264026	59
Men	0	CVLT_12	Model 2c PICALM x age x education	age_education	0.02139056421511476	0.10521262895996801	-0.18973402153954586	0.23251514996977538	0.839687835432871	59

Sex	Cluster	Outcome	Model	Predictor	Beta	SE	CI_low	CI_high	p	N
Men	0	CVLT_12	Model 2c PICALM x age x education	education_c	0.15759225366319995	0.3851085308783917	-0.6151845494259252	0.9303690567523251	0.6840624408685756	59
Men	0	CVLT_12	Model 3a APOE x PICALM	APOE	0.7142857142857104	0.5190362882866021	-0.3258862515928158	1.7544576801642366	0.1743445530476972	59
Men	0	CVLT_12	Model 3a APOE x PICALM	APOE_PICALM	-1.0168067226890751	0.757403917486709	-2.534678092371077	0.5010646469929267	0.18495241082455113	59
Men	0	CVLT_12	Model 3a APOE x PICALM	PICALM	0.5882352941176432	0.3776543717883878	-0.168600979551215	1.3450715677865013	0.12506367818289554	59
Men	0	CVLT_12	Model 3b APOE x PICALM x age	APOE	0.714285714285716	0.5276887927308331	-0.34412396850827354	1.7726953970797055	0.18160505595928433	59
Men	0	CVLT_12	Model 3b APOE x PICALM x age	APOE_PICALM	-0.9951896456256852	0.7714845859617625	-2.5425917643679545	0.5522124731165838	0.2026623809525629	59
Men	0	CVLT_12	Model 3b APOE x PICALM x age	APOE_PICALM_age	0.028954802259886225	0.14593574763677802	-0.2637552391363004	0.32166484365607284	0.8434850635313323	59
Men	0	CVLT_12	Model 3b APOE x PICALM x age	PICALM	0.5595238095238049	0.38900162101641367	-0.22071463400202607	1.3397622530496358	0.1562115092717543	59
Men	0	CVLT_12	Model 3b APOE x PICALM x age	age_c	-0.028954802259889556	0.06301686127049443	-0.15535061939068545	0.09744101487090634	0.647770788986584	59
Men	0	CVLT_12	Model 3c APOE x PICALM x age x education	APOE	0.6216506284336314	0.5781834029029735	-0.5402524205593024	1.7835536774265652	0.28756044613077064	59
Men	0	CVLT_12	Model 3c APOE x PICALM x age x education	APOE_PICALM	-0.8203732426481762	0.8669206290397794	-2.5625154713230205	0.921768986026668	0.3486353506443274	59
Men	0	CVLT_12	Model 3c APOE x PICALM x age x education	APOE_PICALM_age	0.09534791109937735	0.16966941224721369	-0.24561553825089583	0.4363113604496505	0.5767030117549916	59
Men	0	CVLT_12	Model 3c APOE x PICALM x age x education	APOE_PICALM_education	-1.0231023643796862	1.0395637635419066	-3.112183961010378	1.0659792322510055	0.3298691098795582	59
Men	0	CVLT_12	Model 3c APOE x PICALM x age x education	APOE_age_education	-0.262644333184624	0.3056137044282422	-0.8767980657309542	0.3515093993617062	0.39430506661368947	59
Men	0	CVLT_12	Model 3c APOE x PICALM x age x education	PICALM	0.5902204339452348	0.3992066547320432	-0.2120153739014814	1.392456241791951	0.1456783955952089	59
Men	0	CVLT_12	Model 3c APOE x PICALM x age x education	age_c	-0.03170880151367006	0.06466062490935985	-0.1616491921488217	0.09823158912148158	0.626048109126262	59
Men	0	CVLT_12	Model 3c APOE x PICALM x age x education	age_education	0.09405429210149047	0.11725417008798378	-0.14157678455746173	0.32968536876044263	0.42634510530205394	59
Men	0	CVLT_12	Model 3c APOE x PICALM x age x education	education_c	0.23960793461118746	0.3248162577198587	-0.4131347735196299	0.8923506427420048	0.464231752381001	59
Men	0	CVLT_13	Model 1a APOE	APOE	0.42698412698412724	0.44261763040551	-0.4593423895761395	1.313310643544394	0.3387804689822733	59
Men	0	CVLT_13	Model 1b APOE x age	APOE	0.4056162055396302	0.43883001855333775	-0.47381880389286546	1.2850512149721258	0.35936115495998366	59
Men	0	CVLT_13	Model 1b APOE x age	APOE_age	0.2118861916566098	0.14258768018737406	-0.07386590498419829	0.49763828829741785	0.1429875038424025	59
Men	0	CVLT_13	Model 1b APOE x age	age_c	-0.0012007077856421161	0.07642006638897544	-0.15434994317107853	0.15194852759979433	0.9875210562474592	59
Men	0	CVLT_13	Model 1c APOE x age x education	APOE	0.4213213674024563	0.4333262167150296	-0.44861731252699977	1.2912600473319125	0.33549236620090406	59

Sex	Cluster	Outcome	Model	Predictor	Beta	SE	CI_low	CI_high	p	N
Men	0	CVLT_13	Model 1c APOE x age x education	APOE_age	0.1650709472841621	0.14172465741422316	-0.1194531747942042	0.4495950693625284	0.2495476490208753	59
Men	0	CVLT_13	Model 1c APOE x age x education	APOE_age_education	0.5390285942077615	0.24568532900839085	0.04579471508580957	1.0322624733297134	0.03281881182930126	59
Men	0	CVLT_13	Model 1c APOE x age x education	APOE_education	-0.0976137816313205	0.6559768449236769	-1.4145422492030821	1.2193146859404411	0.8822929764212504	59
Men	0	CVLT_13	Model 1c APOE x age x education	age_c	0.00590325635078047	0.07553835105767752	-0.14574631126903298	0.1575528239705939	0.938015240382909	59
Men	0	CVLT_13	Model 1c APOE x age x education	age_education	-0.09552503893950073	0.129311470687762	-0.3551286488079236	0.16407857092892217	0.46346295945218596	59
Men	0	CVLT_13	Model 1c APOE x age x education	education_c	-0.29629528467856703	0.3630782758779867	-1.0252053386854687	0.43261476932833476	0.4182585954436706	59
Men	0	CVLT_13	Model 2a PICALM	PICALM	0.2178571428571429	0.3853556804789361	-0.5538042968435084	0.9895185825577941	0.574060876451862	59
Men	0	CVLT_13	Model 2b PICALM x age	PICALM	0.30522866186372155	0.38685269495037244	-0.47004146335291963	1.0804987870803626	0.43349722042386474	59
Men	0	CVLT_13	Model 2b PICALM x age	PICALM_age	0.17537151748520347	0.13198808115714164	-0.08913850801411066	0.4398815429845176	0.1894360796256136	59
Men	0	CVLT_13	Model 2b PICALM x age	age_c	-0.009483667017913651	0.0876610727526356	-0.1851603825653633	0.166193048529536	0.9142421697984102	59
Men	0	CVLT_13	Model 2c PICALM x age x education	PICALM	0.252455878376363	0.39312743981712167	-0.5364120427147452	1.0413237994674711	0.5235819227597288	59
Men	0	CVLT_13	Model 2c PICALM x age x education	PICALM_age	0.1894216465404257	0.13398927567160415	-0.07944750539852852	0.4582907984793799	0.16340679041792383	59
Men	0	CVLT_13	Model 2c PICALM x age x education	PICALM_education	0.5282464499316595	0.6616705716584826	-0.7994926886901754	1.8559855885534944	0.4282970292795667	59
Men	0	CVLT_13	Model 2c PICALM x age x education	age_c	-0.02355957976803158	0.08960900215102777	-0.20337319763915737	0.1562540381030942	0.793654774995142	59
Men	0	CVLT_13	Model 2c PICALM x age x education	age_education	0.09616349880874042	0.12073763257681526	-0.1461142858522379	0.33844128346971875	0.4293822368817216	59
Men	0	CVLT_13	Model 2c PICALM x age x education	education_c	-0.5541753551269575	0.44193451644558646	-1.4409818405989756	0.33263113034506053	0.21545876260427202	59
Men	0	CVLT_13	Model 3a APOE x PICALM	APOE	-0.6428571428571432	0.5857869488550174	-1.8168004218289064	0.53108613611462	0.2772372081518764	59
Men	0	CVLT_13	Model 3a APOE x PICALM	APOE_PICALM	2.231092436974789	0.8548098464174944	0.5180152235576134	3.9441696503919648	0.011640695669684713	59
Men	0	CVLT_13	Model 3a APOE x PICALM	PICALM	-0.3739495798319328	0.42622261133603395	-1.2281187805997889	0.4802196209359233	0.3841089229762302	59
Men	0	CVLT_13	Model 3b APOE x PICALM x age	APOE	-0.642857142857141	0.5656905954050176	-1.777488789179736	0.4917745034654539	0.2608968479058313	59
Men	0	CVLT_13	Model 3b APOE x PICALM x age	APOE_PICALM	2.252012651431253	0.8270434786382048	0.5931735061989145	3.9108517966635916	0.008739624811861657	59
Men	0	CVLT_13	Model 3b APOE x PICALM x age	APOE_PICALM_age	0.3536894367402835	0.15644539188391532	0.0398997184831858	0.6674791549973813	0.027903520655830977	59
Men	0	CVLT_13	Model 3b APOE x PICALM x age	PICALM	-0.38311688311688286	0.4170157896806781	-1.2195446332532214	0.4533108670194558	0.36240918949569856	59

Sex	Cluster	Outcome	Model	Predictor	Beta	SE	CI_low	CI_high	p	N
Men	0	CVLT_13	Model 3b APOE x PICALM x age	age_c	-0.0092449922958397	0.06755505567624395	-0.14474327468194165	0.12625329009026226	0.8916669302983122	59
Men	0	CVLT_13	Model 3c APOE x PICALM x age x education	APOE	-0.5749979257995261	0.6093516799382721	-1.7995359725065798	0.6495401209075276	0.3499922587729404	59
Men	0	CVLT_13	Model 3c APOE x PICALM x age x education	APOE_PICALM	2.0789848201750734	0.9136539358034491	0.24292849547879403	3.9150411448713527	0.02728818959319455	59
Men	0	CVLT_13	Model 3c APOE x PICALM x age x education	APOE_PICALM_age	0.35876099839974784	0.1788158236087049	-0.0005828527311755871	0.7181048495306712	0.05035839457286566	59
Men	0	CVLT_13	Model 3c APOE x PICALM x age x education	APOE_PICALM_education	-0.4269662907914218	1.0956037868550093	-2.62866453056027	1.7747319489774265	0.6984407531608537	59
Men	0	CVLT_13	Model 3c APOE x PICALM x age x education	APOE_age_education	0.24991714695831763	0.32208849868483513	-0.39734392416286074	0.897178218079496	0.44152126635886035	59
Men	0	CVLT_13	Model 3c APOE x PICALM x age x education	PICALM	-0.4198324479686685	0.4207267875247707	-1.2653145817753828	0.42564968583804585	0.32324279497356323	59
Men	0	CVLT_13	Model 3c APOE x PICALM x age x education	age_c	-0.006853738430228573	0.06814630135792552	-0.14379885814106236	0.1300913812806052	0.9202991183427034	59
Men	0	CVLT_13	Model 3c APOE x PICALM x age x education	age_education	-0.10192934061959097	0.12357501990570084	-0.35026264054984024	0.1464039593106583	0.41346120447683454	59
Men	0	CVLT_13	Model 3c APOE x PICALM x age x education	education_c	-0.31009783697082705	0.3423262088103787	-0.9980281092164974	0.37783243527484334	0.36944562900641353	59
Men	0	CVLT_2	Model 1a APOE	APOE	0.2714285714285749	0.5529996103449366	-0.8359340472885464	1.3787911901456962	0.6254313945043302	59
Men	0	CVLT_2	Model 1b APOE x age	APOE	0.26676403562783824	0.5629564826393242	-0.861425966624306	1.3949540378799825	0.6374736104607719	59
Men	0	CVLT_2	Model 1b APOE x age	APOE_age	0.03791810724420186	0.18291970811524694	-0.328661179564932	0.40449739405333573	0.8365460794251588	59
Men	0	CVLT_2	Model 1b APOE x age	age_c	0.0034125379170878434	0.09803607310007263	-0.1930561429532661	0.19988121878744178	0.9723580680837322	59
Men	0	CVLT_2	Model 1c APOE x age x education	APOE	0.30144125967296187	0.5683086529424379	-0.8394859685043314	1.4423684878502552	0.5981240961550175	59
Men	0	CVLT_2	Model 1c APOE x age x education	APOE_age	0.06550984590812392	0.185872319829598	-0.30764440673273136	0.4386640985489792	0.7259566256912395	59
Men	0	CVLT_2	Model 1c APOE x age x education	APOE_age_education	-0.16584968630117958	0.3222170572437361	-0.8127274209426328	0.4810280483402737	0.6089763627226217	59
Men	0	CVLT_2	Model 1c APOE x age x education	APOE_education	0.050593893532583145	0.860315629933764	-1.6765618024714861	1.7777495895366524	0.9533344467480278	59
Men	0	CVLT_2	Model 1c APOE x age x education	age_c	-0.0013853589040185854	0.09906877746866945	-0.20027422869515052	0.19750351088711335	0.9888974594388631	59
Men	0	CVLT_2	Model 1c APOE x age x education	age_education	-0.02736298338207701	0.16959238763274825	-0.3678339083626945	0.3131079415985405	0.8724586967836097	59
Men	0	CVLT_2	Model 1c APOE x age x education	education_c	0.6659771892833699	0.47617826459038876	-0.2899905664854544	1.6219449450521943	0.16798841604046147	59
Men	0	CVLT_2	Model 2a PICALM	PICALM	0.088095238095238	0.47978084649096353	-0.8726493350323173	1.0488398112227935	0.8549666649865736	59
Men	0	CVLT_2	Model 2b PICALM x age	PICALM	0.12680648256320248	0.4911615738359181	-0.8575033072347558	1.1111162723611607	0.7972341879351703	59

Sex	Cluster	Outcome	Model	Predictor	Beta	SE	CI_low	CI_high	p	N
Men	0	CVLT_2	Model 2b PICALM x age	PICALM_age	0.12671971480062738	0.16757663708932152	-0.20911137055936585	0.4625508001606206	0.45276235754369765	59
Men	0	CVLT_2	Model 2b PICALM x age	age_c	-0.03793466807165463	0.11129753267675398	-0.26097990782545666	0.18511057168214742	0.7345225019301929	59
Men	0	CVLT_2	Model 2c PICALM x age x education	PICALM	0.1693765895752033	0.49467758723877664	-0.8232666103931122	1.162019789543519	0.7334315823284484	59
Men	0	CVLT_2	Model 2c PICALM x age x education	PICALM_age	0.10604871547341833	0.16860052210024778	-0.2322729835307764	0.44437041447761305	0.5321065571030135	59
Men	0	CVLT_2	Model 2c PICALM x age x education	PICALM_education	-0.023519908938014478	0.8325890507342422	-1.6942320675232252	1.6471922496471962	0.9775715992657832	59
Men	0	CVLT_2	Model 2c PICALM x age x education	age_c	-0.02568699906945915	0.11275622225598252	-0.25194891221025206	0.20057491407133377	0.820687319721969	59
Men	0	CVLT_2	Model 2c PICALM x age x education	age_education	-0.07209733240277595	0.15192579993857674	-0.37695875345596214	0.23276408865041026	0.6370902616437042	59
Men	0	CVLT_2	Model 2c PICALM x age x education	education_c	0.6452029421378582	0.5560921934488607	-0.47067768116604425	1.7610835654417607	0.25124856197213513	59
Men	0	CVLT_2	Model 3a APOE x PICALM	APOE	1.2857142857142851	0.7507862661646991	-0.21889501435821623	2.7903235857867865	0.09243950228435623	59
Men	0	CVLT_2	Model 3a APOE x PICALM	APOE_PICALM	-2.1764705882352953	1.0955851681691382	-4.372072329153618	0.019131152683027786	0.05195990435819696	59
Men	0	CVLT_2	Model 3a APOE x PICALM	PICALM	0.6050420168067205	0.5462772489988496	-0.48972205427896975	1.6998060878924108	0.27286656608225657	59
Men	0	CVLT_2	Model 3b APOE x PICALM x age	APOE	1.2857142857142867	0.7636488721621769	-0.24597138145400677	2.81739995288258	0.09813198638887255	59
Men	0	CVLT_2	Model 3b APOE x PICALM x age	APOE_PICALM	-2.203673361522194	1.1164598188855566	-4.443008172185213	0.03566144914082381	0.053626775819940777	59
Men	0	CVLT_2	Model 3b APOE x PICALM x age	APOE_PICALM_age	-0.057447354904982095	0.21119203330857078	-0.48104492995668585	0.36615022014672166	0.7866675278744781	59
Men	0	CVLT_2	Model 3b APOE x PICALM x age	PICALM	0.639971139971137	0.5629466709367266	-0.4891568908377284	1.7690991707800023	0.2607258725905713	59
Men	0	CVLT_2	Model 3b APOE x PICALM x age	age_c	0.035225132682758176	0.09119533274029602	-0.14768954075277108	0.21813980611828743	0.7008502597855621	59
Men	0	CVLT_2	Model 3c APOE x PICALM x age x education	APOE	1.389839704642636	0.8240606064644577	-0.2661720840020416	3.045851493287314	0.09804286719664564	59
Men	0	CVLT_2	Model 3c APOE x PICALM x age x education	APOE_PICALM	-2.301897198048997	1.2355856908659038	-4.784899605764341	0.18110520966634702	0.06846192982840861	59
Men	0	CVLT_2	Model 3c APOE x PICALM x age x education	APOE_PICALM_age	-0.11242663688857846	0.24182271240042877	-0.598387571503906	0.3735342977267491	0.644052872434234	59
Men	0	CVLT_2	Model 3c APOE x PICALM x age x education	APOE_PICALM_education	0.2794099468317768	1.481646725142293	-2.698070622187808	3.2568905158513615	0.8512003553132501	59
Men	0	CVLT_2	Model 3c APOE x PICALM x age x education	APOE_age_education	0.07055639798470725	0.43557842260866386	-0.8047712139174786	0.9458840098868931	0.8719851751764299	59
Men	0	CVLT_2	Model 3c APOE x PICALM x age x education	PICALM	0.6774336297373149	0.5689725376955178	-0.4659594926221806	1.8208267520968104	0.23953650157123044	59
Men	0	CVLT_2	Model 3c APOE x PICALM x age x education	age_c	0.040908676280014045	0.09215808255589043	-0.14428992432561552	0.2261072768856436	0.6590713546992211	59

Sex	Cluster	Outcome	Model	Predictor	Beta	SE	CI_low	CI_high	p	N
Men	0	CVLT_2	Model 3c APOE x PICALM x age x education	age_education	-0.02115719299196571	0.1671174613938294	-0.356992305100907	0.31467791911697557	0.8997744962631129	59
Men	0	CVLT_2	Model 3c APOE x PICALM x age x education	education_c	0.6397582709150296	0.46294701816451217	-0.2905685928911944	1.5700851347212534	0.17326259001265176	59
Men	0	CVLT_3	Model 1a APOE	APOE	0.2650793650793686	0.6828391706283068	-1.102282488354734	1.6324412185134711	0.6993128543198681	59
Men	0	CVLT_3	Model 1b APOE x age	APOE	0.24989093740826274	0.6851950470773331	-1.1232706222226274	1.6230524970391529	0.716733598288627	59
Men	0	CVLT_3	Model 1b APOE x age	APOE_age	-0.19652875175315665	0.22263830665166873	-0.6427058887587624	0.2496483852524492	0.3812260025140236	59
Men	0	CVLT_3	Model 1b APOE x age	age_c	0.15217391304347805	0.11932331147187122	-0.08695534683651138	0.3913031729234675	0.2075610688921107	59
Men	0	CVLT_3	Model 1c APOE x age x education	APOE	0.33658448753464976	0.659858043412146	-0.9881358111319369	1.6613047862012364	0.6121916474224836	59
Men	0	CVLT_3	Model 1c APOE x age x education	APOE_age	-0.1386163621658253	0.2158146715736491	-0.5718823942131256	0.294649669881475	0.5235595232675727	59
Men	0	CVLT_3	Model 1c APOE x age x education	APOE_age_education	-0.554441497786253	0.3741233145862491	-1.3055253922463725	0.19664239667386652	0.14449977778082201	59
Men	0	CVLT_3	Model 1c APOE x age x education	APOE_education	0.7839332939809356	0.9989047067042992	-1.2214515832907156	2.789318171252587	0.4362054412392885	59
Men	0	CVLT_3	Model 1c APOE x age x education	age_c	0.12970000492525657	0.11502786263282667	-0.10122806523052566	0.3606280750810388	0.26478572802135175	59
Men	0	CVLT_3	Model 1c APOE x age x education	age_education	0.3192120213803085	0.19691218935614935	-0.07610569414845558	0.7145297369090726	0.11116590683577042	59
Men	0	CVLT_3	Model 1c APOE x age x education	education_c	0.8510958697417985	0.5528862817083138	-0.2588696562460815	1.9610613957296785	0.12989638023822447	59
Men	0	CVLT_3	Model 2a PICALM	PICALM	0.2404761904761914	0.5912799895962897	-0.9435415654603247	1.4244939464127075	0.6857482059147304	59
Men	0	CVLT_3	Model 2b PICALM x age	PICALM	0.2569611056438008	0.5881247363859645	-0.9216672042337954	1.435589415521397	0.663882910214081	59
Men	0	CVLT_3	Model 2b PICALM x age	PICALM_age	-0.33700993667707085	0.20065894964643177	-0.7391394579362819	0.06511958458214018	0.09872421008754878	59
Men	0	CVLT_3	Model 2b PICALM x age	age_c	0.25289778714436184	0.13326944849270989	-0.014180155879274958	0.5199757301679986	0.06299708158214003	59
Men	0	CVLT_3	Model 2c PICALM x age x education	PICALM	0.27881590473168494	0.5747326266077739	-0.8744694842155165	1.4321012936788864	0.6296268160860726	59
Men	0	CVLT_3	Model 2c PICALM x age x education	PICALM_age	-0.346147916061774	0.19588560996870985	-0.7392211494630455	0.04692531733949756	0.08308101880551105	59
Men	0	CVLT_3	Model 2c PICALM x age x education	PICALM_education	-0.01020810849737952	0.9673292349555924	-1.951296227263786	1.930880010269027	0.9916205602208505	59
Men	0	CVLT_3	Model 2c PICALM x age x education	age_c	0.2465301963219772	0.13100387293728372	-0.01634830675833049	0.5094086994022848	0.06545808324072763	59
Men	0	CVLT_3	Model 2c PICALM x age x education	age_education	0.13775324591922655	0.1765123714934721	-0.216444740392011	0.4919512322304641	0.4386836794238693	59
Men	0	CVLT_3	Model 2c PICALM x age x education	education_c	1.0764945320255257	0.6460861280595512	-0.21997213263984938	2.372961196690901	0.10169149532402892	59

Sex	Cluster	Outcome	Model	Predictor	Beta	SE	CI_low	CI_high	p	N
Men	0	CVLT_3	Model 3a APOE x PICALM	APOE	0.9999999999999987	0.9469734439156111	-0.8977771901924383	2.8977771901924356	0.29558668741798055	59
Men	0	CVLT_3	Model 3a APOE x PICALM	APOE_PICALM	-1.6218487394957997	1.3818713881167342	-4.391180886027671	1.1474834070360722	0.2455882256286559	59
Men	0	CVLT_3	Model 3a APOE x PICALM	PICALM	0.6218487394957943	0.6890243883386443	-0.7589869915132605	2.002684470504849	0.3707237040497221	59
Men	0	CVLT_3	Model 3b APOE x PICALM x age	APOE	1.0000000000000013	0.9468571890773851	-0.8991550151298979	2.8991550151299004	0.2957013591255357	59
Men	0	CVLT_3	Model 3b APOE x PICALM x age	APOE_PICALM	-1.740965091110434	1.384311618027665	-4.517542575241421	1.0356123930205539	0.2140353727484349	59
Men	0	CVLT_3	Model 3b APOE x PICALM x age	APOE_PICALM_age	-0.24359270672830036	0.2618594779664956	-0.7688163059956266	0.28163089253902585	0.3564636415254955	59
Men	0	CVLT_3	Model 3b APOE x PICALM x age	PICALM	0.7752525252525198	0.6980041768861808	-0.6247665572520826	2.1752716077571224	0.27172381063031753	59
Men	0	CVLT_3	Model 3b APOE x PICALM x age	age_c	0.1547038178394089	0.1130741621747796	-0.0720942301165785	0.3815018657953963	0.17703387336746843	59
Men	0	CVLT_3	Model 3c APOE x PICALM x age x education	APOE	1.313215481572732	0.962087463158322	-0.6201716603427219	3.2466026234881857	0.17849910260177856	59
Men	0	CVLT_3	Model 3c APOE x PICALM x age x education	APOE_PICALM	-2.1208047426666132	1.4425413537725895	-5.019700125742899	0.7780906404096726	0.1479036866587295	59
Men	0	CVLT_3	Model 3c APOE x PICALM x age x education	APOE_PICALM_age	-0.3871682184108134	0.2823270498338371	-0.9545256665286452	0.18018922970701845	0.17651470044698472	59
Men	0	CVLT_3	Model 3c APOE x PICALM x age x education	APOE_PICALM_education	1.5919972423295035	1.7298166274502842	-1.8841994169690033	5.06819390162801	0.36191068845351104	59
Men	0	CVLT_3	Model 3c APOE x PICALM x age x education	APOE_age_education	-0.11680604482985357	0.5085360667973483	-1.1387475318529057	0.9051354421931985	0.8192881974750602	59
Men	0	CVLT_3	Model 3c APOE x PICALM x age x education	PICALM	0.8924019298496577	0.6642731628038857	-0.4425049688105509	2.2273088285098663	0.18532292096925826	59
Men	0	CVLT_3	Model 3c APOE x PICALM x age x education	age_c	0.15065771814355688	0.1075941929030386	-0.06556090757329605	0.3668763438604098	0.16774178577452167	59
Men	0	CVLT_3	Model 3c APOE x PICALM x age x education	age_education	0.3339961791847128	0.1951089679819354	-0.05808997141362626	0.7260823297830519	0.0932486451622439	59
Men	0	CVLT_3	Model 3c APOE x PICALM x age x education	education_c	0.8941909097551085	0.5404887926793711	-0.19196198395923514	1.9803438034694523	0.10443658984462609	59
Men	0	CVLT_4	Model 1a APOE	APOE	0.18730158730158986	0.584061448262643	-0.982261288947834	1.3568644635510139	0.7496195069526411	59
Men	0	CVLT_4	Model 1b APOE x age	APOE	0.1842431743552646	0.5853668800085879	-0.9888582678361895	1.3573446165467187	0.7541438215047379	59
Men	0	CVLT_4	Model 1b APOE x age	APOE_age	0.2504199419420069	0.19020144919461698	-0.13075228009050077	0.6315921639745146	0.19343160910272209	59
Men	0	CVLT_4	Model 1b APOE x age	age_c	-0.09719413549039452	0.10193873240402886	-0.30148392037979677	0.10709564939900773	0.3445306292606124	59
Men	0	CVLT_4	Model 1c APOE x age x education	APOE	0.20830441072972106	0.6011240609638249	-0.9985024980076008	1.415111319467043	0.730374010217804	59
Men	0	CVLT_4	Model 1c APOE x age x education	APOE_age	0.2722397590119442	0.19660500176838203	-0.12246125170117578	0.6669407697250642	0.17217128737657664	59

Sex	Cluster	Outcome	Model	Predictor	Beta	SE	CI_low	CI_high	p	N
Men	0	CVLT_4	Model 1c APOE x age x education	APOE_age_education	-0.145255342970782	0.3408225881469841	-0.8294852394917056	0.5389745535501416	0.6717625234948015	59
Men	0	CVLT_4	Model 1c APOE x age x education	APOE_education	0.4290509178083415	0.9099921715054659	-1.3978345968204964	2.2559364324371796	0.6393027207767362	59
Men	0	CVLT_4	Model 1c APOE x age x education	age_c	-0.10080141623297079	0.10478922944134714	-0.3111745725633217	0.1095717400973801	0.34061638010410566	59
Men	0	CVLT_4	Model 1c APOE x age x education	age_education	0.013775058549322317	0.17938503000881556	-0.34635541633399497	0.3739055334326396	0.9390907072349859	59
Men	0	CVLT_4	Model 1c APOE x age x education	education_c	0.32655483075226377	0.5036738586879719	-0.6846126334820603	1.337722294986588	0.5196699322462417	59
Men	0	CVLT_4	Model 2a PICALM	PICALM	0.5619047619047619	0.5007676011056051	-0.44086506244122936	1.5646745862507532	0.26653012129387177	59
Men	0	CVLT_4	Model 2b PICALM x age	PICALM	0.524281468720591	0.5121886319573422	-0.5021674872135249	1.550730424654707	0.3105007065166938	59
Men	0	CVLT_4	Model 2b PICALM x age	PICALM_age	-0.1461449829136415	0.17475074002321084	-0.49635329183307497	0.20406332600579194	0.4066019717167596	59
Men	0	CVLT_4	Model 2b PICALM x age	age_c	0.05268703898840865	0.11606227774849896	-0.17990696327012662	0.2852810412469439	0.651647623288329	59
Men	0	CVLT_4	Model 2c PICALM x age x education	PICALM	0.5592128615914198	0.5236633421551371	-0.4915945108721116	1.6100202340549512	0.29050449219844676	59
Men	0	CVLT_4	Model 2c PICALM x age x education	PICALM_age	-0.15983478345387497	0.1784797111689235	-0.5179805256393282	0.19831095873157822	0.3746281901271693	59
Men	0	CVLT_4	Model 2c PICALM x age x education	PICALM_education	-0.1554721831707646	0.8813748109813081	-1.9240801316882556	1.6131357653467264	0.8606669991037782	59
Men	0	CVLT_4	Model 2c PICALM x age x education	age_c	0.06148498483170106	0.11936320083742567	-0.17803480077065575	0.3010047704340579	0.6086601804877995	59
Men	0	CVLT_4	Model 2c PICALM x age x education	age_education	-0.04789966425045111	0.1608279295601599	-0.37062451526703216	0.27482518676612994	0.7670183118464113	59
Men	0	CVLT_4	Model 2c PICALM x age x education	education_c	0.5182559770822444	0.5886765523242702	-0.663009945853982	1.6995219000184707	0.3827071411005901	59
Men	0	CVLT_4	Model 3a APOE x PICALM	APOE	0.8214285714285707	0.800740919313164	-0.7832920906871306	2.426149233544272	0.3094597750297078	59
Men	0	CVLT_4	Model 3a APOE x PICALM	APOE_PICALM	-1.493697478991597	1.1684815163536504	-3.835386766209921	0.8479918082267268	0.20650374766879118	59
Men	0	CVLT_4	Model 3a APOE x PICALM	PICALM	0.9222689075630215	0.582624598073358	-0.24533687882182686	2.08987469394787	0.11916553582274861	59
Men	0	CVLT_4	Model 3b APOE x PICALM x age	APOE	0.8214285714285718	0.8140185321878486	-0.8112858396217351	2.4541429824788787	0.31751015024454654	59
Men	0	CVLT_4	Model 3b APOE x PICALM x age	APOE_PICALM	-1.4708322846404203	1.1901006027059582	-3.857871802543278	0.9162072332624374	0.22194956715087386	59
Men	0	CVLT_4	Model 3b APOE x PICALM x age	APOE_PICALM_age	0.10416452662215359	0.22512208847615559	-0.34737320079649037	0.5557022540407975	0.6454718838305924	59
Men	0	CVLT_4	Model 3b APOE x PICALM x age	PICALM	0.896103896103893	0.6000781766081508	-0.30750050350555425	2.09970829571334	0.14128525448350487	59
Men	0	CVLT_4	Model 3b APOE x PICALM x age	age_c	-0.026386748844377195	0.09721050289703423	-0.22136632573293968	0.1685928280441853	0.7871076920380939	59

Sex	Cluster	Outcome	Model	Predictor	Beta	SE	CI_low	CI_high	p	N
Men	0	CVLT_4	Model 3c APOE x PICALM x age x education	APOE	0.9753024167826903	0.8923692546208887	-0.8179807396789249	2.768585573244305	0.27976622235883325	59
Men	0	CVLT_4	Model 3c APOE x PICALM x age x education	APOE_PICALM	-1.6829410216073257	1.338006784123345	-4.371766322092528	1.0058842788778768	0.21442646070846821	59
Men	0	CVLT_4	Model 3c APOE x PICALM x age x education	APOE_PICALM_age	0.013706919358539293	0.26186806155073683	-0.512536652528857	0.5399504912459356	0.9584683262410627	59
Men	0	CVLT_4	Model 3c APOE x PICALM x age x education	APOE_PICALM_education	1.1247303200822754	1.6044644936970844	-2.0995617953044885	4.349022435469039	0.4866185355671744	59
Men	0	CVLT_4	Model 3c APOE x PICALM x age x education	APOE_age_education	0.14108692887418656	0.47168471501131093	-0.8067989941449065	1.0889728518932795	0.7661189931923045	59
Men	0	CVLT_4	Model 3c APOE x PICALM x age x education	PICALM	0.9162200455567273	0.6161362348595699	-0.3219520747151099	2.1543921658285647	0.14341090691745262	59
Men	0	CVLT_4	Model 3c APOE x PICALM x age x education	age_c	-0.024204168532564352	0.09979731926578544	-0.2247543900609676	0.1763460529958389	0.8093793176692649	59
Men	0	CVLT_4	Model 3c APOE x PICALM x age x education	age_education	0.018480334635426643	0.18097028700106751	-0.3451930727780902	0.3821537420489435	0.9190797225866986	59
Men	0	CVLT_4	Model 3c APOE x PICALM x age x education	education_c	0.2477732234751251	0.5013219686606228	-0.7596709905741432	1.2552174375243934	0.6233454387539796	59
Men	0	CVLT_5	Model 1a APOE	APOE	0.33809523809524133	0.9133434210622577	-1.4908434150526122	2.167033891243095	0.712625423054773	59
Men	0	CVLT_5	Model 1b APOE x age	APOE	0.32359519275040904	0.9283451982204678	-1.5368501588348475	2.184040544335666	0.7287414254774082	59
Men	0	CVLT_5	Model 1b APOE x age	APOE_age	-0.02536163932287494	0.30164433295543885	-0.6298703911909563	0.5791471125452065	0.9332997373876584	59
Men	0	CVLT_5	Model 1b APOE x age	age_c	0.07374873609706778	0.16166670163944533	-0.25023857395502547	0.397736046149161	0.6500584974831332	59
Men	0	CVLT_5	Model 1c APOE x age x education	APOE	0.36491050820580107	0.8970567548822314	-1.4360060739479577	2.16582709035956	0.6858670301072234	59
Men	0	CVLT_5	Model 1c APOE x age x education	APOE_age	0.07683943809894722	0.2933934213133646	-0.5121724328471998	0.6658513090450943	0.7944542322689232	59
Men	0	CVLT_5	Model 1c APOE x age x education	APOE_age_education	-0.9863563669065543	0.5086091620147207	-2.0074318660012453	0.0347191321881366	0.05800375978627368	59
Men	0	CVLT_5	Model 1c APOE x age x education	APOE_education	1.6041167480085594	1.3579802861826442	-1.1221424349405713	4.33037593095769	0.24298198221214728	59
Men	0	CVLT_5	Model 1c APOE x age x education	age_c	0.0546096531654674	0.15637684833068327	-0.25932996959635335	0.36854927592728814	0.7283632589573676	59
Men	0	CVLT_5	Model 1c APOE x age x education	age_education	0.2566213959951152	0.26769607697310877	-0.2808009035133167	0.794043695503547	0.3422680454657493	59
Men	0	CVLT_5	Model 1c APOE x age x education	education_c	0.8019959833093708	0.7516319284728049	-0.7069680775438256	2.3109600441625675	0.290994046572547	59
Men	0	CVLT_5	Model 2a PICALM	PICALM	0.17380952380952386	0.7915940978416635	-1.411330314897033	1.7589493625160808	0.8269912568026935	59
Men	0	CVLT_5	Model 2b PICALM x age	PICALM	0.17511679857321694	0.8046982707978272	-1.4375345731409652	1.787768170287399	0.8285321272257405	59
Men	0	CVLT_5	Model 2b PICALM x age	PICALM_age	-0.2900495356646921	0.27455044790808664	-0.8402609285445914	0.2601618572152073	0.2953799173452593	59

Sex	Cluster	Outcome	Model	Predictor	Beta	SE	CI_low	CI_high	p	N
Men	0	CVLT_5	Model 2b PICALM x age	age_c	0.20126448893572124	0.18234515251180486	-0.16416336271362497	0.5666923405850675	0.27450496785473044	59
Men	0	CVLT_5	Model 2c PICALM x age x education	PICALM	0.243525152212281	0.8053419141771195	-1.3725116268535	1.859561931278062	0.7635631034231292	59
Men	0	CVLT_5	Model 2c PICALM x age x education	PICALM_age	-0.3186572541007988	0.27448396835075317	-0.8694496322324912	0.23213512403089354	0.25097133124482046	59
Men	0	CVLT_5	Model 2c PICALM x age x education	PICALM_education	-0.20658391419757782	1.3554664232595843	-2.9265262817998163	2.5133584534046607	0.8794549839387376	59
Men	0	CVLT_5	Model 2c PICALM x age x education	age_c	0.21477261607564635	0.18356868030728424	-0.15358488977235596	0.5831301219236487	0.2473415476736779	59
Men	0	CVLT_5	Model 2c PICALM x age x education	age_education	-0.03810870508231498	0.24733729138281163	-0.5344272906082457	0.45820988044361577	0.8781463530976514	59
Men	0	CVLT_5	Model 2c PICALM x age x education	education_c	1.2946923511881012	0.9053257375796327	-0.5219766476661849	3.1113613500423876	0.1586741477391785	59
Men	0	CVLT_5	Model 3a APOE x PICALM	APOE	1.2142857142857135	1.2714491719432197	-1.3337553659644001	3.762326794535827	0.34373606365198883	59
Men	0	CVLT_5	Model 3a APOE x PICALM	APOE_PICALM	-1.9033613445378155	1.8553627279009752	-5.6215913404968845	1.814868651421253	0.30944362496818556	59
Men	0	CVLT_5	Model 3a APOE x PICALM	PICALM	0.6176470588235259	0.9251151588575299	-1.2363251492266165	2.4716192668736685	0.5071531183995115	59
Men	0	CVLT_5	Model 3b APOE x PICALM x age	APOE	1.2142857142857153	1.2887193334570286	-1.3705579278844326	3.7991293564558632	0.350343933404353	59
Men	0	CVLT_5	Model 3b APOE x PICALM x age	APOE_PICALM	-1.987345422665184	1.8841163865690738	-5.766404319738877	1.791713474408509	0.2963065770029175	59
Men	0	CVLT_5	Model 3b APOE x PICALM x age	APOE_PICALM_age	-0.1650188323917139	0.35640366445673927	-0.8798740550924321	0.5498363903090043	0.6452520624149483	59
Men	0	CVLT_5	Model 3b APOE x PICALM x age	PICALM	0.7261904761904718	0.9500181104010856	-1.1793045442259544	2.631685496606898	0.4480210430663666	59
Men	0	CVLT_5	Model 3b APOE x PICALM x age	age_c	0.1094632768361562	0.15389951155261744	-0.19922005214188232	0.4181466058141947	0.4800390718040578	59
Men	0	CVLT_5	Model 3c APOE x PICALM x age x education	APOE	1.3515114081266706	1.327519299918413	-1.3162385038005167	4.019261320053857	0.31364389093458045	59
Men	0	CVLT_5	Model 3c APOE x PICALM x age x education	APOE_PICALM	-2.0414109565465157	1.9904650682974483	-6.041400268167829	1.9585783550747973	0.31011910584852803	59
Men	0	CVLT_5	Model 3c APOE x PICALM x age x education	APOE_PICALM_age	-0.34576434211134854	0.3895639657470205	-1.1286224409544243	0.43709375673172723	0.3791094258934664	59
Men	0	CVLT_5	Model 3c APOE x PICALM x age x education	APOE_PICALM_education	3.297014923696363	2.386856752837803	-1.4995533013811926	8.093583148773918	0.17344721268693564	59
Men	0	CVLT_5	Model 3c APOE x PICALM x age x education	APOE_age_education	-0.24252191412572133	0.7016944604619442	-1.6526297259008067	1.167585897649364	0.7311053567146591	59
Men	0	CVLT_5	Model 3c APOE x PICALM x age x education	PICALM	0.8219100813625113	0.9165855265852152	-1.0200374955742015	2.663857658299224	0.37425902968913716	59
Men	0	CVLT_5	Model 3c APOE x PICALM x age x education	age_c	0.10572053960181504	0.14846193626620796	-0.1926248911750159	0.404065970378646	0.479776549136253	59
Men	0	CVLT_5	Model 3c APOE x PICALM x age x education	age_education	0.26418933705106024	0.2692176444466982	-0.27682377462728863	0.8052024487294092	0.33125433287182904	59

Sex	Cluster	Outcome	Model	Predictor	Beta	SE	CI_low	CI_high	p	N
Men	0	CVLT_5	Model 3c APOE x PICALM x age x education	education_c	0.7706392358780073	0.7457838618081993	-0.728069545062365	2.2693480168183795	0.3065237464580983	59
Men	0	CVLT_6	Model 1a APOE	APOE	0.34285714285714786	0.6720737877602834	-1.0029474032276946	1.6886616889419903	0.6119176041027437	59
Men	0	CVLT_6	Model 1b APOE x age	APOE	0.36176467018675496	0.6793740107321972	-0.9997312719233291	1.723260612296839	0.5965266216951027	59
Men	0	CVLT_6	Model 1b APOE x age	APOE_age	-0.16056459767115772	0.2207468953223445	-0.6029512616691775	0.28182206632686196	0.4700845376935352	59
Men	0	CVLT_6	Model 1b APOE x age	age_c	-0.01080637007077867	0.11830960692765062	-0.2479041206471263	0.22629138050556896	0.927554495265172	59
Men	0	CVLT_6	Model 1c APOE x age x education	APOE	0.37188761445144847	0.6610382377186765	-0.9552020231507479	1.6989772520536448	0.5761848922787678	59
Men	0	CVLT_6	Model 1c APOE x age x education	APOE_age	-0.08213710868263013	0.21620066860619247	-0.5161780621078547	0.35190384474259434	0.7055886962448679	59
Men	0	CVLT_6	Model 1c APOE x age x education	APOE_age_education	-0.8350100191714457	0.3747924557905175	-1.5874372706533042	-0.08258276768958728	0.030323288581825356	59
Men	0	CVLT_6	Model 1c APOE x age x education	APOE_education	0.5443712277298105	1.0006913055938464	-1.46460039647656	2.553342851936181	0.5888152528177317	59
Men	0	CVLT_6	Model 1c APOE x age x education	age_c	-0.02583631324427793	0.11523359662353419	-0.2571774116208069	0.205504785132251	0.8234911396190085	59
Men	0	CVLT_6	Model 1c APOE x age x education	age_education	0.19764738180881095	0.1972643782050764	-0.19837738233714533	0.5936721459547672	0.32110213046489133	59
Men	0	CVLT_6	Model 1c APOE x age x education	education_c	0.6494698149484412	0.5538751508269757	-0.4624809486330308	1.7614205785299133	0.24640739955252697	59
Men	0	CVLT_6	Model 2a PICALM	PICALM	0.05952380952380887	0.5833069061239625	-1.108528122155328	1.2275757412029458	0.9190787503118292	59
Men	0	CVLT_6	Model 2b PICALM x age	PICALM	-0.044013354559296225	0.5861013870420272	-1.2185867817394034	1.130560072620811	0.9404115084833261	59
Men	0	CVLT_6	Model 2b PICALM x age	PICALM_age	-0.31169061382862234	0.19996861453722153	-0.7124366726134992	0.08905544495625456	0.12480409271892816	59
Men	0	CVLT_6	Model 2b PICALM x age	age_c	0.08271865121180166	0.13281095621294006	-0.18344045275038406	0.3488777551739874	0.5359702826596615	59
Men	0	CVLT_6	Model 2c PICALM x age x education	PICALM	0.023663879865833515	0.5903424371243587	-1.1609448854820914	1.2082726452137584	0.968178865685833	59
Men	0	CVLT_6	Model 2c PICALM x age x education	PICALM_age	-0.3346759702384502	0.20120588780396134	-0.7384251221598697	0.06907318168296928	0.10225854988937455	59
Men	0	CVLT_6	Model 2c PICALM x age x education	PICALM_education	-0.45139444083413105	0.9936020187958505	-2.445202757353668	1.5424138756854056	0.6515028782289327	59
Men	0	CVLT_6	Model 2c PICALM x age x education	age_c	0.09897417120598007	0.134561954623997	-0.17104414512311933	0.36899248753507946	0.4653223734201184	59
Men	0	CVLT_6	Model 2c PICALM x age x education	age_education	-0.08853961277386141	0.18130646973200223	-0.4523576609985973	0.2752784354508745	0.6273604963396231	59
Men	0	CVLT_6	Model 2c PICALM x age x education	education_c	0.9570227762808425	0.663633908661341	-0.37465608626503744	2.2887016388267227	0.15527189197152078	59
Men	0	CVLT_6	Model 3a APOE x PICALM	APOE	1.428571428571427	0.9201148688188102	-0.4153799743116864	3.2725228314545403	0.12625427865347363	59

Sex	Cluster	Outcome	Model	Predictor	Beta	SE	CI_low	CI_high	p	N
Men	0	CVLT_6	Model 3a APOE x PICALM	APOE_PICALM	-2.319327731092439	1.342678001342985	-5.010114575320945	0.3714591131360674	0.08971008572481529	59
Men	0	CVLT_6	Model 3a APOE x PICALM	PICALM	0.6050420168067197	0.6694819044425806	-0.7366297012979164	1.9467137349113557	0.37007124752210707	59
Men	0	CVLT_6	Model 3b APOE x PICALM x age	APOE	1.4285714285714288	0.9214290672798681	-0.41958123309464557	3.2767240902375034	0.12700006405133174	59
Men	0	CVLT_6	Model 3b APOE x PICALM x age	APOE_PICALM	-2.3406328651968136	1.3471355318818496	-5.042644563419241	0.3613788330256136	0.08810925328033133	59
Men	0	CVLT_6	Model 3b APOE x PICALM x age	APOE_PICALM_age	-0.3226887519260404	0.2548271664665382	-0.8338073205644992	0.18842981671241843	0.21094157522707288	59
Men	0	CVLT_6	Model 3b APOE x PICALM x age	PICALM	0.6165223665223625	0.6792590742141169	-0.7458988013659256	1.9789435344106505	0.36817726036434795	59
Men	0	CVLT_6	Model 3b APOE x PICALM x age	age_c	0.011577640814926737	0.11003752306900892	-0.2091296804154355	0.23228496204528898	0.9166020947071685	59
Men	0	CVLT_6	Model 3c APOE x PICALM x age x education	APOE	1.3690566786642346	0.9640352394796251	-0.5682446663139764	3.3063580236424457	0.16190114198196473	59
Men	0	CVLT_6	Model 3c APOE x PICALM x age x education	APOE_PICALM	-2.1136237937221196	1.4454618241029638	-5.018388081655097	0.7911404942108571	0.15005776292654804	59
Men	0	CVLT_6	Model 3c APOE x PICALM x age x education	APOE_PICALM_age	-0.37259443012332705	0.28289862982379405	-0.9411005112350149	0.19591165098836083	0.19394482561094895	59
Men	0	CVLT_6	Model 3c APOE x PICALM x age x education	APOE_PICALM_education	1.346972917854965	1.7333186956053803	-2.1362614108867617	4.830207246596691	0.44083168878654566	59
Men	0	CVLT_6	Model 3c APOE x PICALM x age x education	APOE_age_education	-0.4080544102227224	0.5095656140551256	-1.432064849920458	0.6159560294750133	0.4271198228603862	59
Men	0	CVLT_6	Model 3c APOE x PICALM x age x education	PICALM	0.6924905879593197	0.6656180047095676	-0.6451188716924008	2.0301000476110405	0.3032733083495742	59
Men	0	CVLT_6	Model 3c APOE x PICALM x age x education	age_c	0.007523085626587767	0.10781202072979176	-0.20913328149686602	0.22417945275004156	0.944653044240571	59
Men	0	CVLT_6	Model 3c APOE x PICALM x age x education	age_education	0.20586877141027135	0.19550397222267413	-0.1870111699288173	0.59874871274936	0.2974988854998837	59
Men	0	CVLT_6	Model 3c APOE x PICALM x age x education	education_c	0.6447241942196156	0.5415830292354267	-0.443627650181339	1.7330760386205701	0.23960747129204082	59
Men	0	CVLT_7	Model 1a APOE	APOE	0.033333333333336546	0.8812742982463814	-1.7313880090659635	1.7980546757326366	0.9699601583104145	59
Men	0	CVLT_7	Model 1b APOE x age	APOE	0.037594729904035784	0.8960825110940416	-1.7581947518506165	1.833384211658688	0.9666868756381323	59
Men	0	CVLT_7	Model 1b APOE x age	APOE_age	-0.1311676424540924	0.2911613178482829	-0.7146679625835367	0.4523326776753519	0.6541221824732717	59
Men	0	CVLT_7	Model 1b APOE x age	age_c	0.039433771486349745	0.15604831504817215	-0.27329404022700077	0.35216158319970026	0.8014403302668516	59
Men	0	CVLT_7	Model 1c APOE x age x education	APOE	0.06691963631540832	0.8526148162180105	-1.6447760310546886	1.7786153036855052	0.9377473896342444	59
Men	0	CVLT_7	Model 1c APOE x age x education	APOE_age	-0.023411126480921696	0.27885813983476265	-0.5832422022336551	0.5364199492718117	0.9334220543038355	59
Men	0	CVLT_7	Model 1c APOE x age x education	APOE_age_education	-1.0137111626588553	0.4834116736068813	-1.9842005929732465	-0.04322173234446425	0.04097186692520977	59

Sex	Cluster	Outcome	Model	Predictor	Beta	SE	CI_low	CI_high	p	N
Men	0	CVLT_7	Model 1c APOE x age x education	APOE_education	1.9523422650109494	1.2907032981244322	-0.6388527285967824	4.543537258618681	0.13654906413000495	59
Men	0	CVLT_7	Model 1c APOE x age x education	age_c	0.023386698104921666	0.14862963471884355	-0.27499974434459973	0.32177314055444306	0.8755912548142137	59
Men	0	CVLT_7	Model 1c APOE x age x education	age_education	0.20834593964657594	0.2544338919789683	-0.3024514125086934	0.7191432918018452	0.41667586969216486	59
Men	0	CVLT_7	Model 1c APOE x age x education	education_c	0.7069950119391875	0.7143946189252681	-0.7272120306161471	2.141202054494522	0.32702126817595845	59
Men	0	CVLT_7	Model 2a PICALM	PICALM	0.11309523809523903	0.7630684871828994	-1.4149230505619594	1.6411135267524375	0.8826992283243851	59
Men	0	CVLT_7	Model 2b PICALM x age	PICALM	0.05748035047894051	0.7762378605561024	-1.498135084560044	1.613095785517925	0.9412393304723585	59
Men	0	CVLT_7	Model 2b PICALM x age	PICALM_age	-0.30498163339668743	0.26484020164178573	-0.8357332579021537	0.22576999110877877	0.2544798582920249	59
Men	0	CVLT_7	Model 2b PICALM x age	age_c	0.139093782929399	0.17589600500595712	-0.21340968830419088	0.4915972541629889	0.4324732932717821	59
Men	0	CVLT_7	Model 2c PICALM x age x education	PICALM	0.12373312674344561	0.7729014316502572	-1.4272070617051476	1.6746733151920388	0.8734311160718401	59
Men	0	CVLT_7	Model 2c PICALM x age x education	PICALM_age	-0.33847304605005096	0.2634273075431696	-0.867078611097555	0.19013251899745304	0.20452752791505746	59
Men	0	CVLT_7	Model 2c PICALM x age x education	PICALM_education	0.035333197746986844	1.3008660305004034	-2.575045466169928	2.6457118616639015	0.9784350634958415	59
Men	0	CVLT_7	Model 2c PICALM x age x education	age_c	0.15541388088212993	0.17617423521364672	-0.19810558534352057	0.5089333471077804	0.3817500398623356	59
Men	0	CVLT_7	Model 2c PICALM x age x education	age_education	-0.07118817253910867	0.23737414288886535	-0.5475142379713069	0.40513789289308955	0.7654500875291412	59
Men	0	CVLT_7	Model 2c PICALM x age x education	education_c	1.2101101921933113	0.868857743980815	-0.533380423818897	2.9536008082055196	0.16961897700915887	59
Men	0	CVLT_7	Model 3a APOE x PICALM	APOE	1.0714285714285692	1.2212088625165238	-1.375928678804142	3.5187858216612806	0.3841124980696251	59
Men	0	CVLT_7	Model 3a APOE x PICALM	APOE_PICALM	-2.239495798319329	1.782049535674785	-5.810802873851227	1.3318112772125685	0.21417464812624554	59
Men	0	CVLT_7	Model 3a APOE x PICALM	PICALM	0.6680672268907522	0.8885599643110625	-1.1126467342264224	2.448781188007927	0.45534446966824627	59
Men	0	CVLT_7	Model 3b APOE x PICALM x age	APOE	1.0714285714285727	1.2330682158082014	-1.4017930643824765	3.5446502072396218	0.3888128182200439	59
Men	0	CVLT_7	Model 3b APOE x PICALM x age	APOE_PICALM	-2.3056086194167524	1.8027540759628016	-5.921475387822106	1.3102581489886016	0.206490868850391	59
Men	0	CVLT_7	Model 3b APOE x PICALM x age	APOE_PICALM_age	-0.33017890772128045	0.34101298803385277	-1.014164342821561	0.353806527379	0.33732743747987803	59
Men	0	CVLT_7	Model 3b APOE x PICALM x age	PICALM	0.7420634920634872	0.9089932198311403	-1.0811460183839177	2.5652730025108923	0.4179498475890323	59
Men	0	CVLT_7	Model 3b APOE x PICALM x age	age_c	0.0746233521657228	0.1472536270677958	-0.2207300206215395	0.3699767249529851	0.6144199059638933	59
Men	0	CVLT_7	Model 3c APOE x PICALM x age x education	APOE	1.3017934107769218	1.2452158134141327	-1.200561452741863	3.8041482742957067	0.3009515372486237	59

Sex	Cluster	Outcome	Model	Predictor	Beta	SE	CI_low	CI_high	p	N
Men	0	CVLT_7	Model 3c APOE x PICALM x age x education	APOE_PICALM	-2.5190171119593794	1.8670602975374837	-6.2710152521178575	1.2329810281990983	0.18347795878204975	59
Men	0	CVLT_7	Model 3c APOE x PICALM x age x education	APOE_PICALM_age	-0.5684056824456651	0.3654117951538063	-1.3027281773416965	0.16591681245036627	0.12625794239484908	59
Men	0	CVLT_7	Model 3c APOE x PICALM x age x education	APOE_PICALM_education	4.096590385883354	2.2388765068580194	-0.40260040128893415	8.595781173055641	0.07337406246010011	59
Men	0	CVLT_7	Model 3c APOE x PICALM x age x education	APOE_age_education	-0.05416963290821175	0.6581908364014071	-1.3768536390458619	1.2685143732294384	0.9347427967869391	59
Men	0	CVLT_7	Model 3c APOE x PICALM x age x education	PICALM	0.824482146987199	0.8597590951186735	-0.9032684404599298	2.5522327344343276	0.34228255486183057	59
Men	0	CVLT_7	Model 3c APOE x PICALM x age x education	age_c	0.07286868511523059	0.13925759929827375	-0.2069799380166465	0.3527173082471077	0.6031480221368268	59
Men	0	CVLT_7	Model 3c APOE x PICALM x age x education	age_education	0.21627771159343956	0.25252670009071426	-0.29119369162282194	0.723749114809701	0.39591540205167164	59
Men	0	CVLT_7	Model 3c APOE x PICALM x age x education	education_c	0.6795356932940444	0.6995467848713792	-0.7262562027968547	2.0853275893849434	0.336122916139898	59
Men	0	CVLT_8	Model 1a APOE	APOE	-0.07142857142856762	0.7284026865707983	-1.5300297917413646	1.3871726488842293	0.9222271469517749	59
Men	0	CVLT_8	Model 1b APOE x age	APOE	-0.03926653901847832	0.7314529562887857	-1.505131020290443	1.4265979422534865	0.9573823022268457	59
Men	0	CVLT_8	Model 1b APOE x age	APOE_age	-0.16644786196549233	0.23766874596966056	-0.6427466724768638	0.30985094854587913	0.48666988639694664	59
Men	0	CVLT_8	Model 1b APOE x age	age_c	-0.06540697674418605	0.12737889642161582	-0.3206799896190537	0.1898660361306816	0.6096697405605176	59
Men	0	CVLT_8	Model 1c APOE x age x education	APOE	-0.03219790380114218	0.7177796781567264	-1.4732007363311101	1.4088049287288258	0.9643959615673794	59
Men	0	CVLT_8	Model 1c APOE x age x education	APOE_age	-0.08801202046909573	0.2347586530924176	-0.5593096823586389	0.3832856414204474	0.7092862279903032	59
Men	0	CVLT_8	Model 1c APOE x age x education	APOE_age_education	-0.8134363111670745	0.4069634598163368	-1.6304495482059331	0.0035769258717841534	0.05097396564381492	59
Men	0	CVLT_8	Model 1c APOE x age x education	APOE_education	0.30210205738044094	1.086587495667789	-1.8793133639503408	2.4835174787112226	0.7821147217996876	59
Men	0	CVLT_8	Model 1c APOE x age x education	age_c	-0.07895592950158803	0.12512488563858684	-0.33015461917224	0.17224276016906392	0.5308473383414642	59
Men	0	CVLT_8	Model 1c APOE x age x education	age_education	0.14213702222072924	0.21419693116162078	-0.287881260430799	0.5721553048722574	0.5099471630282641	59
Men	0	CVLT_8	Model 1c APOE x age x education	education_c	0.7681858967994426	0.6014180493879204	-0.43921121832675425	1.9755830119256395	0.20728388711711765	59
Men	0	CVLT_8	Model 2a PICALM	PICALM	-0.11190476190476151	0.6306942931429703	-1.3748482992955164	1.1510387754859934	0.8597988265991268	59
Men	0	CVLT_8	Model 2b PICALM x age	PICALM	-0.2626724734249657	0.6314933461457238	-1.5282134194501105	1.002868472600179	0.6790619222568063	59
Men	0	CVLT_8	Model 2b PICALM x age	PICALM_age	-0.298745359108947	0.2154556401163737	-0.7305281107143893	0.13303739249649532	0.17116639288935806	59
Men	0	CVLT_8	Model 2b PICALM x age	age_c	0.013698630136985912	0.14309680372366834	-0.27307377287078244	0.30047103314475426	0.9240831154234264	59

Sex	Cluster	Outcome	Model	Predictor	Beta	SE	CI_low	CI_high	p	N
Men	0	CVLT_8	Model 2c PICALM x age x education	PICALM	-0.15325441882962876	0.6303021693424938	-1.418048253164195	1.1115394155049374	0.8088501704914983	59
Men	0	CVLT_8	Model 2c PICALM x age x education	PICALM_age	-0.3288225549604024	0.21482532779632077	-0.7599011126292192	0.10225600270841445	0.13191709197514148	59
Men	0	CVLT_8	Model 2c PICALM x age x education	PICALM_education	-1.043871057610044	1.0608580182050829	-3.1726384104653476	1.0848962952452594	0.3296779897983042	59
Men	0	CVLT_8	Model 2c PICALM x age x education	age_c	0.040946765994092305	0.1436703285700006	-0.24734883981306233	0.32924237180124694	0.7767723581700064	59
Men	0	CVLT_8	Model 2c PICALM x age x education	age_education	-0.1703803028043788	0.19357893656531586	-0.558824857390409	0.21806425178165142	0.3828227435479591	59
Men	0	CVLT_8	Model 2c PICALM x age x education	education_c	1.2948763006135775	0.7085546726337864	-0.12694266943854005	2.716695270665695	0.07336678620997773	59
Men	0	CVLT_8	Model 3a APOE x PICALM	APOE	1.0357142857142843	1.0008011360369407	-0.9699360100703975	3.041364581498966	0.3052514786669314	59
Men	0	CVLT_8	Model 3a APOE x PICALM	APOE_PICALM	-2.329831932773111	1.4604194700178041	-5.2565779530761905	0.5969140875299686	0.11637216405974099	59
Men	0	CVLT_8	Model 3a APOE x PICALM	PICALM	0.47268907563024865	0.728189787197374	-0.9866358686470822	1.9320140199075795	0.518956625470838	59
Men	0	CVLT_8	Model 3b APOE x PICALM x age	APOE	1.0357142857142874	0.9903710599107592	-0.9507185015804309	3.0221470730090054	0.3004069795735739	59
Men	0	CVLT_8	Model 3b APOE x PICALM x age	APOE_PICALM	-2.321313005469977	1.4479291916542532	-5.225491183134334	0.58286517219438	0.11483519161347612	59
Men	0	CVLT_8	Model 3b APOE x PICALM x age	APOE_PICALM_age	-0.3983008046567371	0.2738935203037901	-0.9476616361495774	0.1510600268361032	0.15178269315021595	59
Men	0	CVLT_8	Model 3b APOE x PICALM x age	PICALM	0.43795093795093365	0.7300817319225176	-1.0264075721073898	1.902309448009257	0.5511519638972262	59
Men	0	CVLT_8	Model 3b APOE x PICALM x age	age_c	-0.03503252867659867	0.11827061053491686	-0.2722533321208075	0.20218827476761014	0.768229934158915	59
Men	0	CVLT_8	Model 3c APOE x PICALM x age x education	APOE	0.9430646801220472	1.0475356331434535	-1.1620369882535369	3.0481663484976314	0.3723801217322309	59
Men	0	CVLT_8	Model 3c APOE x PICALM x age x education	APOE_PICALM	-2.034774135040805	1.5706612217969567	-5.191136032283126	1.1215877622015156	0.20122167489252435	59
Men	0	CVLT_8	Model 3c APOE x PICALM x age x education	APOE_PICALM_age	-0.43850860653601265	0.3074020359129692	-1.0562561257498289	0.17923891267780356	0.16006530367908495	59
Men	0	CVLT_8	Model 3c APOE x PICALM x age x education	APOE_PICALM_education	1.1553682067856084	1.8834509599674676	-2.6295682027123095	4.940304616283527	0.5424282738676418	59
Men	0	CVLT_8	Model 3c APOE x PICALM x age x education	APOE_age_education	-0.3891721656966731	0.5537018941709032	-1.501877780974075	0.7235334495807288	0.4854721088609688	59
Men	0	CVLT_8	Model 3c APOE x PICALM x age x education	PICALM	0.5080294996597985	0.7232708405674996	-0.9454376712822989	1.961496670601896	0.48574978311023276	59
Men	0	CVLT_8	Model 3c APOE x PICALM x age x education	age_c	-0.03661153984906962	0.11715021274182841	-0.27203370639947033	0.1988106267013311	0.75597312095506	59
Men	0	CVLT_8	Model 3c APOE x PICALM x age x education	age_education	0.14477028076855475	0.2124376464027252	-0.2821391528763793	0.5716797144134889	0.4987812985010103	59
Men	0	CVLT_8	Model 3c APOE x PICALM x age x education	education_c	0.7247876850180457	0.5884925137551185	-0.45783229786017876	1.9074076678962701	0.22397648899619926	59

Sex	Cluster	Outcome	Model	Predictor	Beta	SE	CI_low	CI_high	p	N
Men	0	CVLT_9	Model 1a APOE	APOE	0.27301587301587404	1.037651543620112	-1.8048455018633958	2.350877247895144	0.7934150173326353	59
Men	0	CVLT_9	Model 1b APOE x age	APOE	0.2897968057958613	1.04659413272247	-1.8076247061076327	2.3872183176993556	0.7828986440054588	59
Men	0	CVLT_9	Model 1b APOE x age	APOE_age	0.25749270197984275	0.3400665933806788	-0.42401598045561784	0.9390013844153033	0.4521718650427031	59
Men	0	CVLT_9	Model 1b APOE x age	age_c	-0.18592012133468172	0.1822591657895938	-0.5511756517419488	0.17933540907258533	0.3121536311739074	59
Men	0	CVLT_9	Model 1c APOE x age x education	APOE	0.0826105682055035	0.926957030826152	-1.7783333226612354	1.9435544590722422	0.9293354674046005	59
Men	0	CVLT_9	Model 1c APOE x age x education	APOE_age	0.3579710461817347	0.30317267352862964	-0.25067349279760365	0.966615585161073	0.24317967497670492	59
Men	0	CVLT_9	Model 1c APOE x age x education	APOE_age_education	-1.5492252297326048	0.525561884581135	-2.6043347395142957	-0.49411571995091363	0.004818357259233599	59
Men	0	CVLT_9	Model 1c APOE x age x education	APOE_education	-2.938523853229754	1.4032438495662074	-5.755653431414388	-0.12139427504511957	0.0412407466368614	59
Men	0	CVLT_9	Model 1c APOE x age x education	age_c	-0.1905878138190214	0.16158912825709873	-0.5149915251674568	0.13381589752941403	0.2436881926586105	59
Men	0	CVLT_9	Model 1c APOE x age x education	age_education	0.034810232964668786	0.2766187973328164	-0.5205251751249789	0.5901456410543164	0.900351865329499	59
Men	0	CVLT_9	Model 1c APOE x age x education	education_c	0.3364252678206372	0.7766849721595988	-1.22283487693518	1.8956854125764544	0.6667284085665202	59
Men	0	CVLT_9	Model 2a PICALM	PICALM	-0.5821428571428567	0.8958659828853225	-2.3760835440245036	1.21179782973879	0.5184251374238531	59
Men	0	CVLT_9	Model 2b PICALM x age	PICALM	-0.7046833836461501	0.91553559233102	-2.539457711372669	1.1300909440803686	0.4447739587153059	59
Men	0	CVLT_9	Model 2b PICALM x age	PICALM_age	-0.12174399023073917	0.31236640623206824	-0.7477402571148937	0.5042522766534154	0.6982285490351035	59
Men	0	CVLT_9	Model 2b PICALM x age	age_c	-0.07218124341412019	0.20746096179387516	-0.4879423016332845	0.3435798148050441	0.7292232227468058	59
Men	0	CVLT_9	Model 2c PICALM x age x education	PICALM	-0.7609422223474644	0.8955783337963875	-2.5580516245424554	1.0361671798475265	0.39940602380627366	59
Men	0	CVLT_9	Model 2c PICALM x age x education	PICALM_age	-0.14439713485671596	0.305239166994758	-0.7569043340864381	0.46811006437300623	0.638148554555641	59
Men	0	CVLT_9	Model 2c PICALM x age x education	PICALM_education	2.018600345710953	1.5073428310261616	-1.0061043303003339	5.04330502172224	0.18633728228421154	59
Men	0	CVLT_9	Model 2c PICALM x age x education	age_c	-0.051623273514074475	0.20413706272171336	-0.461254258219278	0.35800771119112906	0.80135385294485	59
Men	0	CVLT_9	Model 2c PICALM x age x education	age_education	-0.32402001814965353	0.27505077707108283	-0.8759497811890765	0.22790974488976945	0.24414500751816806	59
Men	0	CVLT_9	Model 2c PICALM x age x education	education_c	-1.775580628624986	1.0067650786970406	-3.7958025572400813	0.2446412999901093	0.08366563568168908	59
Men	0	CVLT_9	Model 3a APOE x PICALM	APOE	0.6785714285714284	1.4512969922553234	-2.2298927377610807	3.5870355949039374	0.6419451223226432	59
Men	0	CVLT_9	Model 3a APOE x PICALM	APOE_PICALM	-0.7037815126050422	2.117805733775386	-4.947959045397446	3.5403960201873614	0.7409136124931757	59

Sex	Cluster	Outcome	Model	Predictor	Beta	SE	CI_low	CI_high	p	N
Men	0	CVLT_9	Model 3a APOE x PICALM	PICALM	-0.43907563025210183	1.0559736693899842	-2.555294153683797	1.6771428931795933	0.67917305433628	59
Men	0	CVLT_9	Model 3b APOE x PICALM x age	APOE	0.6785714285714293	1.469614375844285	-2.2691017204398145	3.626244577582673	0.6461615065331467	59
Men	0	CVLT_9	Model 3b APOE x PICALM x age	APOE_PICALM	-0.6160770915131368	2.148586162696865	-4.92559518293776	3.693440999911487	0.7754324165712383	59
Men	0	CVLT_9	Model 3b APOE x PICALM x age	APOE_PICALM_age	-0.008590138674884562	0.4064313580864517	-0.8237882075283878	0.8066079301786186	0.9832169206605786	59
Men	0	CVLT_9	Model 3b APOE x PICALM x age	PICALM	-0.5627705627705631	1.0833703166480915	-2.735736236833724	1.6101951112925978	0.6056007819796585	59
Men	0	CVLT_9	Model 3b APOE x PICALM x age	age_c	-0.12474319465844952	0.17550208857845292	-0.4767558059946033	0.22726941667770428	0.4803375313721989	59
Men	0	CVLT_9	Model 3c APOE x PICALM x age x education	APOE	-1.3957528135435586	1.3958108350481184	-4.2007397033729434	1.4092340762858262	0.32224331945839735	59
Men	0	CVLT_9	Model 3c APOE x PICALM x age x education	APOE_PICALM	2.9210474786742435	2.092860502506533	-1.2847131619287429	7.12680811927723	0.16909151489448357	59
Men	0	CVLT_9	Model 3c APOE x PICALM x age x education	APOE_PICALM_age	0.6790303300484952	0.40960429303545615	-0.14410031425738612	1.5021609743543767	0.10375307920573555	59
Men	0	CVLT_9	Model 3c APOE x PICALM x age x education	APOE_PICALM_education	-4.948892911072974	2.5096437524663693	-9.992210850045494	0.0944250278995451	0.05427394538201664	59
Men	0	CVLT_9	Model 3c APOE x PICALM x age x education	APOE_age_education	-2.6940375996057595	0.7377917073342882	-4.176685544824048	-1.2113896543874707	0.000633168297063925	59
Men	0	CVLT_9	Model 3c APOE x PICALM x age x education	PICALM	-0.5671687236108006	0.9637374080622081	-2.5038715539477314	1.36953410672613	0.5588934271028725	59
Men	0	CVLT_9	Model 3c APOE x PICALM x age x education	age_c	-0.14181757541868928	0.15609925915603026	-0.4555107811528674	0.17187563031548886	0.3680562772115924	59
Men	0	CVLT_9	Model 3c APOE x PICALM x age x education	age_education	0.013607737465172243	0.2830669995742644	-0.5552366953277426	0.5824521702580869	0.9618538730640751	59
Men	0	CVLT_9	Model 3c APOE x PICALM x age x education	education_c	0.1703521749605041	0.7841491984183504	-1.4054546363957556	1.746158986316764	0.8289197962121561	59
Men	0	RPM	Model 1a APOE	APOE	-0.16349206349204426	1.1457081575731765	-2.4577330754602684	2.13074894847618	0.8870306927856926	59
Men	0	RPM	Model 1b APOE x age	APOE	-0.19527086373780644	1.1571269728364755	-2.5142051372539065	2.1236634097782936	0.8666085138991222	59
Men	0	RPM	Model 1b APOE x age	APOE_age	-0.12444653282886353	0.37598168713004215	-0.8779306715340763	0.6290376058763493	0.7419090800060024	59
Men	0	RPM	Model 1b APOE x age	age_c	0.19198685540950367	0.20150791045733726	-0.21184402133401942	0.5958177321530267	0.344884800598649	59
Men	0	RPM	Model 1c APOE x age x education	APOE	-0.24493700695043863	1.1675735467995725	-2.5889387101553574	2.09906469625448	0.8346728917933024	59
Men	0	RPM	Model 1c APOE x age x education	APOE_age	-0.08170022053198966	0.3818692581780721	-0.8483347456328351	0.6849343045688557	0.8314405726471805	59
Men	0	RPM	Model 1c APOE x age x education	APOE_age_education	-0.5736838648614189	0.6619855432739559	-1.9026752977219241	0.7553075679990864	0.39021423301126046	59
Men	0	RPM	Model 1c APOE x age x education	APOE_education	-2.245200669503813	1.7674933615881678	-5.793591656369198	1.303190317361572	0.2097518307457781	59

Sex	Cluster	Outcome	Model	Predictor	Beta	SE	CI_low	CI_high	p	N
Men	0	RPM	Model 1c APOE x age x education	age_c	0.18642466845070205	0.2035339129314784	-0.2221867118594104	0.5950360487608145	0.3640096554483424	59
Men	0	RPM	Model 1c APOE x age x education	age_education	-0.05637431225557288	0.34842261245427797	-0.7558618942298255	0.6431132697186798	0.8721037183132103	59
Men	0	RPM	Model 1c APOE x age x education	education_c	0.8970636150398086	0.9782943518774436	-1.0669442483809968	2.861071478460614	0.3634759354676671	59
Men	0	RPM	Model 2a PICALM	PICALM	0.27142857142857224	0.9917381588529438	-1.7144928363353056	2.2573499791924503	0.7853123805898057	59
Men	0	RPM	Model 2b PICALM x age	PICALM	0.45869353320134465	1.0055946208792972	-1.5565631208754374	2.4739501872781267	0.6500842624776533	59
Men	0	RPM	Model 2b PICALM x age	PICALM_age	0.29963660715165785	0.34309313639091654	-0.3879374030148699	0.9872106173181856	0.3862755543444485	59
Men	0	RPM	Model 2b PICALM x age	age_c	0.03213909378292801	0.22786839634623104	-0.4245193771911998	0.4887975647570558	0.8883518084445544	59
Men	0	RPM	Model 2c PICALM x age x education	PICALM	0.5082572863443939	1.033418335373998	-1.5654483146260074	2.5819628873147953	0.6249154228798893	59
Men	0	RPM	Model 2c PICALM x age x education	PICALM_age	0.2725361321785602	0.35221905213975935	-0.434243103479544	0.9793153678366644	0.44257115726564655	59
Men	0	RPM	Model 2c PICALM x age x education	PICALM_education	0.04340274686760459	1.7393405584900372	-3.4468394277406404	3.5336449214758496	0.9801875176807192	59
Men	0	RPM	Model 2c PICALM x age x education	age_c	0.058167241511564605	0.23555614912181722	-0.41451075253636444	0.5308452355594937	0.8059302427757169	59
Men	0	RPM	Model 2c PICALM x age x education	age_education	-0.2432529942306504	0.31738431520468924	-0.8801312163128318	0.393625227851531	0.44688576749756326	59
Men	0	RPM	Model 2c PICALM x age x education	education_c	0.10333158909061879	1.161718023402192	-2.227826170951969	2.4344893491332065	0.9294655501692829	59
Men	0	RPM	Model 3a APOE x PICALM	APOE	0.96428571428571	1.5930243106174033	-2.2282063450598852	4.156777773631305	0.5474576478462503	59
Men	0	RPM	Model 3a APOE x PICALM	APOE_PICALM	-2.476890756302531	2.3246213814764025	-7.135536108972723	2.1817545963676617	0.29130368291796443	59
Men	0	RPM	Model 3a APOE x PICALM	PICALM	0.905462184873933	1.1590954406209955	-1.4174169862368071	3.228341355984673	0.43804630505616626	59
Men	0	RPM	Model 3b APOE x PICALM x age	APOE	0.9642857142857282	1.6065136820337969	-2.257972669876924	4.18654409844838	0.5509076530719137	59
Men	0	RPM	Model 3b APOE x PICALM x age	APOE_PICALM	-2.617334264572616	2.348733874774467	-7.328297827968923	2.093629298823691	0.27015288504339585	59
Men	0	RPM	Model 3b APOE x PICALM x age	APOE_PICALM_age	-0.09356702619414659	0.44429174639663765	-0.9847034172819846	0.7975693648936915	0.8340084377213675	59
Men	0	RPM	Model 3b APOE x PICALM x age	PICALM	1.0974025974025814	1.1842897463523843	-1.277981818639728	3.472787013444891	0.35831737085483994	59
Men	0	RPM	Model 3b APOE x PICALM x age	age_c	0.1935670261941369	0.19185067263976327	-0.19123669215210765	0.5783707445403814	0.3175846131661591	59
Men	0	RPM	Model 3c APOE x PICALM x age x education	APOE	-0.11699371681126469	1.7283708438292513	-3.5902849651466964	3.3562975315241674	0.9463077347249553	59
Men	0	RPM	Model 3c APOE x PICALM x age x education	APOE_PICALM	-0.707994857387348	2.591496628273006	-5.915802308668699	4.499812593894003	0.785848280469204	59

Sex	Cluster	Outcome	Model	Predictor	Beta	SE	CI_low	CI_high	p	N
Men	0	RPM	Model 3c APOE x PICALM x age x education	APOE_PICALM_age	0.32552992553571514	0.5071948861647645	-0.6937163580995791	1.3447762091710094	0.5239786901384916	59
Men	0	RPM	Model 3c APOE x PICALM x age x education	APOE_PICALM_education	-4.566161159980082	3.1075808993928433	-10.811078782775752	1.6787564628155875	0.14812627127856032	59
Men	0	RPM	Model 3c APOE x PICALM x age x education	APOE_age_education	-1.4814933931322951	0.9135748510876318	-3.3173907911420324	0.3544040048774424	0.11129744231745327	59
Men	0	RPM	Model 3c APOE x PICALM x age x education	PICALM	1.1454420309543991	1.193353422525103	-1.2526914560954747	3.543575518004273	0.3418425508527879	59
Men	0	RPM	Model 3c APOE x PICALM x age x education	age_c	0.19188145118523803	0.1932908109710498	-0.19655097610681244	0.5803138784772885	0.3257290478683992	59
Men	0	RPM	Model 3c APOE x PICALM x age x education	age_education	-0.03332064677886526	0.35050935028564933	-0.7376955574951648	0.6710542639374343	0.9246520892455927	59
Men	0	RPM	Model 3c APOE x PICALM x age x education	education_c	1.0002742965566913	0.970977282685754	-0.9509776065436375	2.95152619965702	0.307987259787067	59
Men	1	CVLT_1	Model 1a APOE	APOE	0.1923076923077014	5.930589641100134	-12.448444903114009	12.83306028772941	0.9745595516341854	17
Men	1	CVLT_1	Model 1b APOE x age	APOE	2.5488361919624847	6.927867912496054	-12.417912502295977	17.515584886220946	0.7188607428598732	17
Men	1	CVLT_1	Model 1b APOE x age	APOE_age	0.29257164888831877	1.6108791338006618	-3.187521141121697	3.7726644388983344	0.8586803964760307	17
Men	1	CVLT_1	Model 1b APOE x age	age_c	-1.145161290322596	0.9337691377208729	-3.1624468678253965	0.8721242871802044	0.24180384579758551	17
Men	1	CVLT_1	Model 1c APOE x age x education	APOE	0.4139022558660752	9.844738154196682	-21.254220326423223	22.08202483815537	0.9672178623544457	17
Men	1	CVLT_1	Model 1c APOE x age x education	APOE_age	-0.6598377219010421	1.8525549226788338	-4.7372836149557696	3.417608171153686	0.7284452140773279	17
Men	1	CVLT_1	Model 1c APOE x age x education	APOE_age_education	7.636950031828594	2.167296662444055	2.8667622402919246	12.407137823365263	0.004767160318355496	17
Men	1	CVLT_1	Model 1c APOE x age x education	APOE_education	-3.7186292913557057	3.138066345515708	-10.62546674919151	3.1882081664800985	0.26098499008666043	17
Men	1	CVLT_1	Model 1c APOE x age x education	age_c	-0.6182108743886414	0.9615051173840606	-2.734469369094773	1.49804762031749	0.5334222788366164	17
Men	1	CVLT_1	Model 1c APOE x age x education	age_education	-3.4540051023367404	1.3154995072244804	-6.349399995834254	-0.5586102088392271	0.023590786239463722	17
Men	1	CVLT_1	Model 1c APOE x age x education	education_c	7.836387934251972	1.7156218740610516	4.0603296491298995	11.612446219374045	0.0008064535285105782	17
Men	1	CVLT_1	Model 2a PICALM	PICALM	1.6944444444444606	5.021184545372052	-9.007957072958297	12.396845961847218	0.7404467968409831	17
Men	1	CVLT_1	Model 2b PICALM x age	PICALM	2.2227867891342603	5.1436139244165755	-8.889315514111733	13.334889092380255	0.6727176444833154	17
Men	1	CVLT_1	Model 2b PICALM x age	PICALM_age	0.21232492997198804	1.5393476314887007	-3.1132334444936984	3.5378833044376745	0.8924079059670622	17
Men	1	CVLT_1	Model 2b PICALM x age	age_c	-1.0980392156862706	1.2629002759974113	-3.82636938818704	1.6302909568144988	0.4003651461069849	17
Men	1	CVLT_1	Model 2c PICALM x age x education	PICALM	3.522306466910468	5.4068885760873	-8.378175051279705	15.42278798510064	0.528132719734156	17

Sex	Cluster	Outcome	Model	Predictor	Beta	SE	CI_low	CI_high	p	N
Men	1	CVLT_1	Model 2c PICALM x age x education	PICALM_age	-0.20658123081030233	1.6902327159913104	-3.9267583557938743	3.5135958941732697	0.9049286241166896	17
Men	1	CVLT_1	Model 2c PICALM x age x education	PICALM_education	-4.754218579046734	1.8158909080128243	-8.75096751991251	-0.7574696381809565	0.02390872434264723	17
Men	1	CVLT_1	Model 2c PICALM x age x education	age_c	-1.0420476551065025	1.3545019824491886	-4.023286417780102	1.9391911075670967	0.4579039309233017	17
Men	1	CVLT_1	Model 2c PICALM x age x education	age_education	0.7398400608650295	1.4180826000667082	-2.3813386976536384	3.8610188193836974	0.6122095882846279	17
Men	1	CVLT_1	Model 2c PICALM x age x education	education_c	-2.126573744714676	4.19092335878868	-11.350733864453549	7.097586375024196	0.6218763541667606	17
Men	1	CVLT_1	Model 3a APOE x PICALM	APOE	0.5714285714285765	8.897903374301087	-18.651322986630174	19.794180129487327	0.9497716020716853	17
Men	1	CVLT_1	Model 3a APOE x PICALM	APOE_PICALM	-0.9047619047619122	12.699515367265194	-28.340396856446603	26.53087304692278	0.9442880805561189	17
Men	1	CVLT_1	Model 3a APOE x PICALM	PICALM	1.9047619047618944	6.174154306939183	-11.433687540103284	15.243211349627073	0.7625855685839016	17
Men	1	CVLT_1	Model 3b APOE x PICALM x age	APOE	2.5714285714285765	8.943199089522485	-17.112419908277754	22.255277051134907	0.7790519121545791	17
Men	1	CVLT_1	Model 3b APOE x PICALM x age	APOE_PICALM	-2.8504983388704375	14.847892248231792	-35.53048883573926	29.829492157998388	0.8512559512894354	17
Men	1	CVLT_1	Model 3b APOE x PICALM x age	APOE_PICALM_age	1.3333333333333237	2.148708402010854	-3.3959419728381133	6.062608639504761	0.547551341736328	17
Men	1	CVLT_1	Model 3b APOE x PICALM x age	PICALM	1.9047619047619264	6.133722528051249	-11.595470355545341	15.404994165069194	0.7619556890220255	17
Men	1	CVLT_1	Model 3b APOE x PICALM x age	age_c	-1.3333333333333202	0.904719327162465	-3.3246071464581357	0.6579404797914956	0.16858061264065136	17
Men	1	CVLT_1	Model 3c APOE x PICALM x age x education	APOE	-3.1959149858208775	7.0786120615556145	-19.208847963934332	12.817017992292577	0.6623201053709684	17
Men	1	CVLT_1	Model 3c APOE x PICALM x age x education	APOE_PICALM	4.563824176853849	9.540394043953755	-17.018046546126726	26.145694899834425	0.64379434442844	17
Men	1	CVLT_1	Model 3c APOE x PICALM x age x education	APOE_PICALM_age	0.8409807770335096	2.2550300777924748	-4.260251665896174	5.942213219963193	0.7178281993716795	17
Men	1	CVLT_1	Model 3c APOE x PICALM x age x education	APOE_PICALM_education	1.1940237672001395	2.4960333254530216	-4.452395898695951	6.84044343309623	0.6437943444284393	17
Men	1	CVLT_1	Model 3c APOE x PICALM x age x education	APOE_age_education	2.6064497255098935	6.903489044417773	-13.010327465004574	18.22322691602436	0.71451059799658	17
Men	1	CVLT_1	Model 3c APOE x PICALM x age x education	PICALM	-0.04689203925846286	6.860460148026858	-15.566331103592514	15.472547025075588	0.9946955188525454	17
Men	1	CVLT_1	Model 3c APOE x PICALM x age x education	age_c	-1.2561549590703143	0.9694108535999937	-3.4491146652900464	0.9368047471494179	0.22729120598876568	17
Men	1	CVLT_1	Model 3c APOE x PICALM x age x education	age_education	-1.0195617408359214	2.914868003440677	-7.613451273593476	5.5743277919216325	0.7345495815310243	17
Men	1	CVLT_1	Model 3c APOE x PICALM x age x education	education_c	-1.818208660495639	9.79666083801811	-23.979795147270547	20.34337782627927	0.8568780034739187	17
Men	1	CVLT_10	Model 1a APOE	APOE	0.21153846153846156	0.7229507527674489	-1.3293945919094878	1.7524715149864112	0.7738332929895737	17

Sex	Cluster	Outcome	Model	Predictor	Beta	SE	CI_low	CI_high	p	N
Men	1	CVLT_10	Model 1b APOE x age	APOE	0.5729928689650189	0.8434100895551897	-1.2490838530529889	2.3950695909830264	0.5088157719934852	17
Men	1	CVLT_10	Model 1b APOE x age	APOE_age	-0.002441845521141184	0.19611108809548575	-0.42611409342709305	0.4212304023848107	0.9902545841680173	17
Men	1	CVLT_10	Model 1b APOE x age	age_c	-0.12903225806451624	0.11367859809343357	-0.37461993829599877	0.1165554221669663	0.27684442731105435	17
Men	1	CVLT_10	Model 1c APOE x age x education	APOE	2.001482787554412	1.1061394662376056	-0.4331137626166317	4.436079337725456	0.09776414570875161	17
Men	1	CVLT_10	Model 1c APOE x age x education	APOE_age	-0.1868437325376967	0.2081501895989254	-0.6449792109133836	0.27129174583799026	0.388602674477607	17
Men	1	CVLT_10	Model 1c APOE x age x education	APOE_age_education	-0.31720794656339674	0.2435140819212607	-0.8531788271433149	0.21876293401652153	0.21931354228154515	17
Men	1	CVLT_10	Model 1c APOE x age x education	APOE_education	0.6551303589530192	0.35258825355017076	-0.12091115473047076	1.4311718726365092	0.0901078045089056	17
Men	1	CVLT_10	Model 1c APOE x age x education	age_c	-0.10877823965898545	0.1080332194386044	-0.3465577524393385	0.1290012731213676	0.33562043289208177	17
Men	1	CVLT_10	Model 1c APOE x age x education	age_education	-0.07394761730343449	0.1478074784687716	-0.39926968396248075	0.2513744493556118	0.6267238982198334	17
Men	1	CVLT_10	Model 1c APOE x age x education	education_c	0.30684347316069305	0.19276460524554234	-0.1174285623739934	0.7311155086953796	0.13973592148179972	17
Men	1	CVLT_10	Model 2a PICALM	PICALM	0.06944444444444464	0.6158799619828663	-1.243272620643029	1.3821615095319182	0.9117188259277889	17
Men	1	CVLT_10	Model 2b PICALM x age	PICALM	0.1169223503354831	0.6354083160971089	-1.2557938598153826	1.4896385604863487	0.8568438172686776	17
Men	1	CVLT_10	Model 2b PICALM x age	PICALM_age	0.049299719887955167	0.19016090647263204	-0.3615179421397332	0.46011738191564355	0.799500328893195	17
Men	1	CVLT_10	Model 2b PICALM x age	age_c	-0.1421568627450979	0.15601041399333046	-0.47919687121779564	0.19488314572759982	0.37877596792868706	17
Men	1	CVLT_10	Model 2c PICALM x age x education	PICALM	0.007586335005564493	0.6729703999136002	-1.473611528379357	1.488784198390486	0.9912075639909972	17
Men	1	CVLT_10	Model 2c PICALM x age x education	PICALM_age	0.09609753926934536	0.2103754443652432	-0.3669356918244109	0.5591307703631017	0.6567097781039637	17
Men	1	CVLT_10	Model 2c PICALM x age x education	PICALM_education	0.08051668369275725	0.22601553802486382	-0.4169401614481315	0.5779735288336459	0.7283965216123087	17
Men	1	CVLT_10	Model 2c PICALM x age x education	age_c	-0.1616869996593572	0.16858859360335318	-0.5327479923395927	0.2093739930208783	0.35813329178678044	17
Men	1	CVLT_10	Model 2c PICALM x age x education	age_education	-0.06922419075206786	0.17650217884978325	-0.4577028671227473	0.3192544856186116	0.7024026395143557	17
Men	1	CVLT_10	Model 2c PICALM x age x education	education_c	0.4072192034035937	0.5216248363697977	-0.7408693205770276	1.555307727384215	0.45146732676579604	17
Men	1	CVLT_10	Model 3a APOE x PICALM	APOE	-0.7142857142857133	1.025384256189573	-2.9294937221862556	1.5009222936148292	0.4983185876941001	17
Men	1	CVLT_10	Model 3a APOE x PICALM	APOE_PICALM	1.8809523809523805	1.4634776948062906	-1.2806989603369456	5.042603722241706	0.2211268723012425	17
Men	1	CVLT_10	Model 3a APOE x PICALM	PICALM	-0.3809523809523808	0.7115025141659015	-1.918060111549602	1.1561553496448402	0.6013996938811452	17

Sex	Cluster	Outcome	Model	Predictor	Beta	SE	CI_low	CI_high	p	N
Men	1	CVLT_10	Model 3b APOE x PICALM x age	APOE	-0.5526695526695503	0.9484501024939785	-2.640194153337949	1.5348550479988483	0.5718412896339263	17
Men	1	CVLT_10	Model 3b APOE x PICALM x age	APOE_PICALM	3.208459526046735	1.5746585515638918	-0.2573405781431015	6.6742596302365715	0.06638231525559021	17
Men	1	CVLT_10	Model 3b APOE x PICALM x age	APOE_PICALM_age	-0.2672558922558923	0.22787625364445305	-0.7688081448626315	0.2342963603508469	0.26564336176583314	17
Men	1	CVLT_10	Model 3b APOE x PICALM x age	PICALM	-0.38095238095238104	0.6504976241908145	-1.8126879984655786	1.0507832365608167	0.5699425234972837	17
Men	1	CVLT_10	Model 3b APOE x PICALM x age	age_c	-0.10774410774410764	0.09594789627134864	-0.31892400357852413	0.10343578809030882	0.2853729431669387	17
Men	1	CVLT_10	Model 3c APOE x PICALM x age x education	APOE	0.8900651071989898	0.7776459895928651	-0.8690923383232745	2.649222552721254	0.28191584974193395	17
Men	1	CVLT_10	Model 3c APOE x PICALM x age x education	APOE_PICALM	1.6942209706893059	1.0480937651195232	-0.6767318474185458	4.065173788797157	0.14044630594422944	17
Men	1	CVLT_10	Model 3c APOE x PICALM x age x education	APOE_PICALM_age	-0.05262425646623643	0.2477343130485421	-0.6130382072137357	0.5077896942812627	0.8365107957759681	17
Men	1	CVLT_10	Model 3c APOE x PICALM x age x education	APOE_PICALM_education	0.44325548651755164	0.27421057808359667	-0.17705193682461984	1.0635629098597232	0.1404463059442296	17
Men	1	CVLT_10	Model 3c APOE x PICALM x age x education	APOE_age_education	-0.7070008911455061	0.7584072305284622	-2.422637240045807	1.0086354577547947	0.3755549430692935	17
Men	1	CVLT_10	Model 3c APOE x PICALM x age x education	PICALM	-0.42420937840785183	0.7536801387731826	-2.1291523028344783	1.2807335460187748	0.587281526477688	17
Men	1	CVLT_10	Model 3c APOE x PICALM x age x education	age_c	-0.14545787868339555	0.1064980614863871	-0.3863732313049024	0.09545747393811133	0.20514935013370605	17
Men	1	CVLT_10	Model 3c APOE x PICALM x age x education	age_education	0.030781496606321027	0.32022314450300027	-0.6936135834428043	0.7551765766554464	0.9255275889485559	17
Men	1	CVLT_10	Model 3c APOE x PICALM x age x education	education_c	-0.2516442434646973	1.0762468610848035	-2.6862837892669265	2.1829953023375315	0.8203593062612293	17
Men	1	CVLT_11	Model 1a APOE	APOE	0.961538461538461	0.7561059796519813	-0.6500632851854413	2.5731402082623633	0.22284476874114476	17
Men	1	CVLT_11	Model 1b APOE x age	APOE	0.9854239772400948	0.9391899105973261	-1.0435724680787895	3.0144204225589792	0.3131885533002422	17
Men	1	CVLT_11	Model 1b APOE x age	APOE_age	0.04848348541318606	0.21838196812736987	-0.4233020736654517	0.5202690444918239	0.8277539606358909	17
Men	1	CVLT_11	Model 1b APOE x age	age_c	-0.05645161290322599	0.12658823234674488	-0.32992886234193775	0.2170256365354858	0.6629726102063911	17
Men	1	CVLT_11	Model 1c APOE x age x education	APOE	2.533687278764015	1.2261730219595517	-0.1651013462630222	5.232475903791052	0.06317099605725007	17
Men	1	CVLT_11	Model 1c APOE x age x education	APOE_age	-0.1691908832054081	0.23073776390069006	-0.6770412774215501	0.3386595110107339	0.4787355850866364	17
Men	1	CVLT_11	Model 1c APOE x age x education	APOE_age_education	-0.35616065140354497	0.26993919558328006	-0.9502928150070731	0.23797151219998308	0.21383497369215512	17
Men	1	CVLT_11	Model 1c APOE x age x education	APOE_education	0.8157210270189859	0.39084963294328867	-0.04453321491306017	1.6759752689510319	0.06095128174892613	17
Men	1	CVLT_11	Model 1c APOE x age x education	age_c	-0.03372199789050502	0.1197565254602335	-0.2973043332515748	0.22986033747056478	0.7834862428451688	17

Sex	Cluster	Outcome	Model	Predictor	Beta	SE	CI_low	CI_high	p	N
Men	1	CVLT_11	Model 1c APOE x age x education	age_education	-0.03832925250740372	0.16384691810946023	-0.39895388779165186	0.32229538277684444	0.819333520349641	17
Men	1	CVLT_11	Model 1c APOE x age x education	education_c	0.3486320985903623	0.21368260129504726	-0.12168013582795822	0.8189443330086829	0.13104766015252522	17
Men	1	CVLT_11	Model 2a PICALM	PICALM	0.4444444444444448	0.6665123278136514	-0.9761929537836489	1.8650818426725386	0.5150134419320143	17
Men	1	CVLT_11	Model 2b PICALM x age	PICALM	0.35233046707054927	0.7073060158844985	-1.1757112801726364	1.8803722143137351	0.6267128002059512	17
Men	1	CVLT_11	Model 2b PICALM x age	PICALM_age	0.18459383753501396	0.21167798677911276	-0.27270865036534986	0.6418963254353778	0.3990009476705293	17
Men	1	CVLT_11	Model 2b PICALM x age	age_c	-0.1274509803921567	0.17366329895696475	-0.5026277282364018	0.24772576745208846	0.4760400923715261	17
Men	1	CVLT_11	Model 2c PICALM x age x education	PICALM	0.31921083968433156	0.7310756206311735	-1.2898757522233129	1.928297431591976	0.6708252875135001	17
Men	1	CVLT_11	Model 2c PICALM x age x education	PICALM_age	0.2517420905086102	0.22853955920590988	-0.251270087795496	0.7547542688127163	0.2941869227335256	17
Men	1	CVLT_11	Model 2c PICALM x age x education	PICALM_education	0.215087008668307	0.24553004077895502	-0.32532096744073424	0.7554949847773482	0.39974962760688704	17
Men	1	CVLT_11	Model 2c PICALM x age x education	age_c	-0.16276894609616216	0.18314477236403234	-0.5658678722161672	0.24032998002384282	0.39316039432765315	17
Men	1	CVLT_11	Model 2c PICALM x age x education	age_education	-0.0033154625981018093	0.19174162780699736	-0.42533593997145114	0.41870501477524746	0.9865138709572602	17
Men	1	CVLT_11	Model 2c PICALM x age x education	education_c	0.5205068704591588	0.5666626660468926	-0.7267092482830918	1.7677229892014092	0.3780333761075483	17
Men	1	CVLT_11	Model 3a APOE x PICALM	APOE	-0.7142857142857126	0.91043143636079	-2.6811532532563427	1.2525818246849174	0.44677468415153165	17
Men	1	CVLT_11	Model 3a APOE x PICALM	APOE_PICALM	3.3809523809523814	1.299411505220279	0.5737444932296651	6.188160268675098	0.021926357362118856	17
Men	1	CVLT_11	Model 3a APOE x PICALM	PICALM	-0.3809523809523805	0.6317380553057902	-1.7457394747286434	0.9838347128238825	0.5568681066510464	17
Men	1	CVLT_11	Model 3b APOE x PICALM x age	APOE	-0.643578643578641	0.929141365115878	-2.688604999817901	1.4014477126606193	0.5028842763578447	17
Men	1	CVLT_11	Model 3b APOE x PICALM x age	APOE_PICALM	4.301989300535812	1.5426013370068188	0.9067466498596879	7.697231951211936	0.017624677809641328	17
Men	1	CVLT_11	Model 3b APOE x PICALM x age	APOE_PICALM_age	-0.2028619528619528	0.22323710317701545	-0.6942035041344699	0.2884795984105643	0.38297160747368497	17
Men	1	CVLT_11	Model 3b APOE x PICALM x age	PICALM	-0.38095238095238126	0.6372546631140523	-1.7835404376600694	1.0216356757553067	0.5620769593312627	17
Men	1	CVLT_11	Model 3b APOE x PICALM x age	age_c	-0.04713804713804708	0.09399456975874335	-0.25401870030542273	0.15974260602932855	0.6259059047126125	17
Men	1	CVLT_11	Model 3c APOE x PICALM x age x education	APOE	1.0070456794653388	0.7696527642873746	-0.7340298341778033	2.748121193108481	0.22314949155807143	17
Men	1	CVLT_11	Model 3c APOE x PICALM x age x education	APOE_PICALM	2.4578598473981264	1.0373206759272722	0.11127745017259194	4.804442244623661	0.04194805470698755	17
Men	1	CVLT_11	Model 3c APOE x PICALM x age x education	APOE_PICALM_age	0.09666859636844112	0.24518791506462828	-0.4579850019402703	0.6513221946771526	0.702565779019361	17

Sex	Cluster	Outcome	Model	Predictor	Beta	SE	CI_low	CI_high	p	N
Men	1	CVLT_11	Model 3c APOE x PICALM x age x education	APOE_PICALM_education	0.6430447275169527	0.27139203730655426	0.0291132863823641	1.2569761686515415	0.04194805470698763	17
Men	1	CVLT_11	Model 3c APOE x PICALM x age x education	APOE_age_education	-1.1280978479005446	0.7506117555333414	-2.826099607202783	0.5699039114016937	0.16711344202379105	17
Men	1	CVLT_11	Model 3c APOE x PICALM x age x education	PICALM	-0.35550708833151606	0.7459332523517128	-2.0429253381499977	1.3319111614869654	0.645009781612917	17
Men	1	CVLT_11	Model 3c APOE x PICALM x age x education	age_c	-0.10233060325348704	0.10540339500388603	-0.3407696482506678	0.13610844174369374	0.35699103242136954	17
Men	1	CVLT_11	Model 3c APOE x PICALM x age x education	age_education	0.19418374088338644	0.316931652260643	-0.5227654664132118	0.9111329481799846	0.5552387499636628	17
Men	1	CVLT_11	Model 3c APOE x PICALM x age x education	education_c	-0.6924962590070959	1.065184393380853	-3.102110764253991	1.717118246239799	0.5318576410004955	17
Men	1	CVLT_12	Model 1a APOE	APOE	0.3461538461538487	0.6031639131400336	-0.9394596024062589	1.6317672947139563	0.5745428845875471	17
Men	1	CVLT_12	Model 1b APOE x age	APOE	0.3219401234004491	0.7431252934144883	-1.2834844683156048	1.927364715116503	0.6719531194077197	17
Men	1	CVLT_12	Model 1b APOE x age	APOE_age	-0.056548001542218285	0.17279270391423984	-0.4298439431436902	0.3167479400592536	0.7486817776054748	17
Men	1	CVLT_12	Model 1b APOE x age	age_c	0.06451612903225401	0.100161763072675	-0.15187020448582716	0.2809024625503352	0.5306990300111887	17
Men	1	CVLT_12	Model 1c APOE x age x education	APOE	-0.0163821682833456	1.0711249798760745	-2.373912353584733	2.3411480170180416	0.9880712218661339	17
Men	1	CVLT_12	Model 1c APOE x age x education	APOE_age	-0.3713389999377471	0.20156126279781247	-0.8149723482033118	0.0722943483278175	0.09252485919708313	17
Men	1	CVLT_12	Model 1c APOE x age x education	APOE_age_education	1.6930873430727214	0.23580572256828086	1.1740824470372981	2.2120922391081446	1.798045607213613e-05	17
Men	1	CVLT_12	Model 1c APOE x age x education	APOE_education	-0.8815546307065687	0.34142718663953886	-1.6330308017490651	-0.1300784596640724	0.02550063269622111	17
Men	1	CVLT_12	Model 1c APOE x age x education	age_c	0.21583594581825039	0.10461346288521806	-0.014416733537002901	0.4460886251735037	0.06351654264074871	17
Men	1	CVLT_12	Model 1c APOE x age x education	age_education	-0.6696951706830871	0.1431286806345522	-0.9847192727419882	-0.354671068624186	0.0006726772400788461	17
Men	1	CVLT_12	Model 1c APOE x age x education	education_c	2.2812131467162002	0.1866627041314688	1.8703713049818833	2.692054988450517	9.640701226380301e-08	17
Men	1	CVLT_12	Model 2a PICALM	PICALM	0.027777777777783452	0.51813808795465	-1.0766074143301319	1.1321629698856988	0.9579527798804222	17
Men	1	CVLT_12	Model 2b PICALM x age	PICALM	0.0653947625561846	0.5389304421152716	-1.0988936726023244	1.2296831977146936	0.9052758863865702	17
Men	1	CVLT_12	Model 2b PICALM x age	PICALM_age	-0.14481792717086828	0.1612876300200546	-0.4932586677410746	0.20362281339933802	0.38557394854855354	17
Men	1	CVLT_12	Model 2b PICALM x age	age_c	0.15196078431372628	0.1323224126250851	-0.13390440846880858	0.43782597709626114	0.2714913171947391	17
Men	1	CVLT_12	Model 2c PICALM x age x education	PICALM	0.4673418311860851	0.5421901147061898	-0.7260105652259106	1.6606942275980807	0.4071121955544885	17
Men	1	CVLT_12	Model 2c PICALM x age x education	PICALM_age	-0.21211453744493225	0.16949257549277205	-0.5851651808487625	0.16093610595889796	0.23672357495684843	17

Sex	Cluster	Outcome	Model	Predictor	Beta	SE	CI_low	CI_high	p	N
Men	1	CVLT_12	Model 2c PICALM x age x education	PICALM_education	-1.2467884930817146	0.18209328449336695	-1.6475731100023778	-0.8460038761610513	2.7752665600738938e-05	17
Men	1	CVLT_12	Model 2c PICALM x age x education	age_c	0.1347042462485517	0.13582628435915256	-0.16424738997514982	0.43365588247225323	0.3426392403559897	17
Men	1	CVLT_12	Model 2c PICALM x age x education	age_education	0.24957488100554603	0.1422019996848935	-0.06340961003502471	0.5625593720461168	0.10701961465154969	17
Men	1	CVLT_12	Model 2c PICALM x age x education	education_c	-0.19069312073868971	0.42025597247850455	-1.1156702796001063	0.734284038122727	0.6588268565256771	17
Men	1	CVLT_12	Model 3a APOE x PICALM	APOE	0.3571428571428594	0.9084895256470273	-1.6055294387881118	2.3198151530738307	0.7006038061711839	17
Men	1	CVLT_12	Model 3a APOE x PICALM	APOE_PICALM	-0.0238095238095255	1.2966399169130252	-2.8250297590246367	2.7774107114055857	0.9856285210371034	17
Men	1	CVLT_12	Model 3a APOE x PICALM	PICALM	0.02380952380952106	0.6303905854701772	-1.338066538368357	1.385685585987399	0.9704453220763534	17
Men	1	CVLT_12	Model 3b APOE x PICALM x age	APOE	0.3116883116883127	0.9799696218506673	-1.8452102833371051	2.4685869067137305	0.7563967043918864	17
Men	1	CVLT_12	Model 3b APOE x PICALM x age	APOE_PICALM	-0.474501031913821	1.6269886431160814	-4.0554788910358095	3.106476827208167	0.7759854335562416	17
Men	1	CVLT_12	Model 3b APOE x PICALM x age	APOE_PICALM_age	0.09469696969696717	0.2354491876014312	-0.42352319816736916	0.6129171375613035	0.6952438891663	17
Men	1	CVLT_12	Model 3b APOE x PICALM x age	PICALM	0.02380952380953061	0.6721153902738674	-1.455506476046611	1.5031255236656722	0.9723757187569797	17
Men	1	CVLT_12	Model 3b APOE x PICALM x age	age_c	0.030303030303033718	0.09913650004270787	-0.18789493511352892	0.24850099571959636	0.7655629682672174	17
Men	1	CVLT_12	Model 3c APOE x PICALM x age x education	APOE	-1.01627775513722	0.7790918057178637	-2.77870586396282	0.7461503536883798	0.22445312455413316	17
Men	1	CVLT_12	Model 3c APOE x PICALM x age x education	APOE_PICALM	0.9259001760647703	1.0500424035570644	-1.4494607684423775	3.301261120571918	0.40083517546256875	17
Men	1	CVLT_12	Model 3c APOE x PICALM x age x education	APOE_PICALM_age	-0.05080441938998126	0.2481949060038386	-0.6122603037904646	0.5106514650105021	0.8423642592315658	17
Men	1	CVLT_12	Model 3c APOE x PICALM x age x education	APOE_PICALM_education	0.2422413251332256	0.274720396279465	-0.3792193870924827	0.8637020373589339	0.40083517546256797	17
Men	1	CVLT_12	Model 3c APOE x PICALM x age x education	APOE_age_education	0.5260772141976302	0.7598172775394262	-1.1927488826484838	2.2449033110437444	0.5061724384568151	17
Men	1	CVLT_12	Model 3c APOE x PICALM x age x education	PICALM	-0.11232279171210746	0.7550813970736336	-1.8204355826401082	1.5957899992158933	0.8850260466836581	17
Men	1	CVLT_12	Model 3c APOE x PICALM x age x education	age_c	0.05415541100735949	0.1066960649695128	-0.18720785661177022	0.2955186786264892	0.6239610593582303	17
Men	1	CVLT_12	Model 3c APOE x PICALM x age x education	age_education	-0.061107671046283674	0.32081851025054636	-0.7868495619857384	0.6646342198931711	0.8531646634909822	17
Men	1	CVLT_12	Model 3c APOE x PICALM x age x education	education_c	-0.16645320248048456	1.0782478423629944	-2.60561928241391	2.272712877452941	0.8807213768984159	17
Men	1	CVLT_13	Model 1a APOE	APOE	0.9423076923076916	0.4048217763552742	0.07945050106272455	1.8051648835526586	0.034332263056756775	17
Men	1	CVLT_13	Model 1b APOE x age	APOE	0.6253422457667731	0.4341687407586317	-0.3126222933833218	1.563306784916868	0.1734238131365999	17

Sex	Cluster	Outcome	Model	Predictor	Beta	SE	CI_low	CI_high	p	N
Men	1	CVLT_13	Model 1b APOE x age	APOE_age	0.01876365505719059	0.10095362294293556	-0.19933338770491077	0.23686069781929192	0.8554200982238848	17
Men	1	CVLT_13	Model 1b APOE x age	age_c	0.09677419354838691	0.05851921194286708	-0.029648877733782095	0.2231972648305559	0.12211623332865719	17
Men	1	CVLT_13	Model 1c APOE x age x education	APOE	1.2663930625892208	0.5286702353121542	0.10279772008960886	2.4299884050888325	0.03551853793292764	17
Men	1	CVLT_13	Model 1c APOE x age x education	APOE_age	-0.0957778928644187	0.09948366645829046	-0.3147399664097578	0.12318418068092041	0.3563600245516175	17
Men	1	CVLT_13	Model 1c APOE x age x education	APOE_age_education	-0.1896979224211684	0.116385547139932	-0.44586078452429323	0.06646493968195644	0.13139491443113435	17
Men	1	CVLT_13	Model 1c APOE x age x education	APOE_education	0.4120019358267736	0.16851664791122256	0.041099294547249476	0.7829045771062977	0.032540288241072614	17
Men	1	CVLT_13	Model 1c APOE x age x education	age_c	0.11023289849068792	0.051633586256896236	-0.0034118586226035974	0.22387765560397943	0.056099503267228915	17
Men	1	CVLT_13	Model 1c APOE x age x education	age_education	0.056443143546704044	0.07064336533327921	-0.09904185521016173	0.21192814230356982	0.44120859055212025	17
Men	1	CVLT_13	Model 1c APOE x age x education	education_c	0.21402453049241058	0.09213025330489809	0.011247210173646827	0.4168018508111743	0.0403593309791802	17
Men	1	CVLT_13	Model 2a PICALM	PICALM	0.652777777777778	0.36428448951391224	-0.12367623185096022	1.4292317874065161	0.09333277147041472	17
Men	1	CVLT_13	Model 2b PICALM x age	PICALM	0.5386864047944768	0.31102280025177026	-0.13323750431418202	1.2106103139031357	0.10691982747067223	17
Men	1	CVLT_13	Model 2b PICALM x age	PICALM_age	0.06442577030812328	0.09308089952743713	-0.1366632875461486	0.26551482816239513	0.5010207781874685	17
Men	1	CVLT_13	Model 2b PICALM x age	age_c	0.07843137254901965	0.07636474783126368	-0.08654463512419151	0.2434073802222308	0.3231228915458112	17
Men	1	CVLT_13	Model 2c PICALM x age x education	PICALM	0.6386356725014077	0.26055399456983835	0.06516019705286025	1.2121111479499551	0.03218473508557557	17
Men	1	CVLT_13	Model 2c PICALM x age x education	PICALM_age	0.10054509856271965	0.08145107481072907	-0.07872750836850112	0.2798177054939404	0.24276624026247037	17
Men	1	CVLT_13	Model 2c PICALM x age x education	PICALM_education	0.2374153888198174	0.08750645091491394	0.04481498894456487	0.43001578869506996	0.020178104219010413	17
Men	1	CVLT_13	Model 2c PICALM x age x education	age_c	0.0531205716231854	0.06527245701728382	-0.09054313763400842	0.19678428088037922	0.43300727948411366	17
Men	1	CVLT_13	Model 2c PICALM x age x education	age_education	0.09038449077450167	0.06833636034437074	-0.06002282423753924	0.24079180578654258	0.2127934632552764	17
Men	1	CVLT_13	Model 2c PICALM x age x education	education_c	0.20868984436945917	0.20195752264949274	-0.23581566594919037	0.6531953546881087	0.3236311733898266	17
Men	1	CVLT_13	Model 3a APOE x PICALM	APOE	0.357142857142858	0.4900901784830275	-0.701632603291258	1.415918317576974	0.47909058455522446	17
Men	1	CVLT_13	Model 3a APOE x PICALM	APOE_PICALM	1.1428571428571435	0.6994802585704438	-0.3682780835716881	2.653992369285975	0.12625896266576594	17
Men	1	CVLT_13	Model 3a APOE x PICALM	PICALM	0.3571428571428572	0.3400680204068023	-0.3775294352087425	1.0918151494944568	0.31275347246045837	17
Men	1	CVLT_13	Model 3b APOE x PICALM x age	APOE	0.20057720057720063	0.4401947872895467	-0.7682849937929619	1.1694393949473632	0.6575009592175876	17

Sex	Cluster	Outcome	Model	Predictor	Beta	SE	CI_low	CI_high	p	N
Men	1	CVLT_13	Model 3b APOE x PICALM x age	APOE_PICALM	1.2951748940121042	0.7308307356777725	-0.31337270974717724	2.9037224977713856	0.10402197844237379	17
Men	1	CVLT_13	Model 3b APOE x PICALM x age	APOE_PICALM_age	-0.10437710437710442	0.10576195704717702	-0.3371576023392713	0.12840339358506248	0.34489974307360083	17
Men	1	CVLT_13	Model 3b APOE x PICALM x age	PICALM	0.35714285714285654	0.30190904356494497	-0.3073544674384239	1.021640181724137	0.26176659881830266	17
Men	1	CVLT_13	Model 3b APOE x PICALM x age	age_c	0.10437710437710442	0.04453135033565348	0.0063642631298762575	0.20238994562433257	0.0389036381378649	17
Men	1	CVLT_13	Model 3c APOE x PICALM x age x education	APOE	0.5121405436668098	0.2929143933870813	-0.1504778494368395	1.1747589367704592	0.11432561955094839	17
Men	1	CVLT_13	Model 3c APOE x PICALM x age x education	APOE_PICALM	0.7228642906219118	0.39478342784677295	-0.17019786845776008	1.6159264497015837	0.10032975984754562	17
Men	1	CVLT_13	Model 3c APOE x PICALM x age x education	APOE_PICALM_age	-0.06843911589309658	0.09331359898836517	-0.27952914223632186	0.14265091045012873	0.48196207930256274	17
Men	1	CVLT_13	Model 3c APOE x PICALM x age x education	APOE_PICALM_education	0.18912147138363986	0.10328636193665588	-0.04452851209650727	0.422771454863787	0.1003297598475459	17
Men	1	CVLT_13	Model 3c APOE x PICALM x age x education	APOE_age_education	-0.26360133005882025	0.28566776765212426	-0.9098267168496137	0.3826240567319732	0.3802077274168675	17
Men	1	CVLT_13	Model 3c APOE x PICALM x age x education	PICALM	0.5670665212649943	0.2838872232495128	-0.07513099425165182	1.2092640367816405	0.07686119320935392	17
Men	1	CVLT_13	Model 3c APOE x PICALM x age x education	age_c	0.09784476100077055	0.040114416449980206	0.0070996464947355176	0.1885898755068056	0.03741725473424794	17
Men	1	CVLT_13	Model 3c APOE x PICALM x age x education	age_education	0.15120641986948907	0.12061782530339767	-0.12165005760847636	0.4240628973474545	0.2415723705026843	17
Men	1	CVLT_13	Model 3c APOE x PICALM x age x education	education_c	0.031099062534305832	0.40538779942072756	-0.8859518516589538	0.9481499767275655	0.9405291140628987	17
Men	1	CVLT_2	Model 1a APOE	APOE	0.6538461538461549	1.2056327367229152	-1.9158991949533457	3.2235915026456556	0.5955598372302708	17
Men	1	CVLT_2	Model 1b APOE x age	APOE	0.4324595696503013	1.2647945889403402	-2.2999630171579004	3.164882156458503	0.7378747447898022	17
Men	1	CVLT_2	Model 1b APOE x age	APOE_age	0.48281069271301913	0.2940920984063493	-0.1525366587969057	1.118158044222944	0.12460987852595969	17
Men	1	CVLT_2	Model 1b APOE x age	age_c	-0.39516129032258307	0.17047469259316944	-0.7634494729206927	-0.026873107724473433	0.037387565005211294	17
Men	1	CVLT_2	Model 1c APOE x age x education	APOE	0.5730244220987939	1.891052743918231	-3.5891546041993734	4.735203448396961	0.7675292516482595	17
Men	1	CVLT_2	Model 1c APOE x age x education	APOE_age	0.21950676836849303	0.35585294549430285	-0.563720283836274	1.00273382057326	0.5498897073273761	17
Men	1	CVLT_2	Model 1c APOE x age x education	APOE_age_education	0.9031788916963052	0.41631095070339985	-0.013115332781902023	1.8194731161745124	0.052824804542779054	17
Men	1	CVLT_2	Model 1c APOE x age x education	APOE_education	-0.26452231494270406	0.602783830340398	-1.591240580259878	1.0621959503744698	0.6692763379916107	17
Men	1	CVLT_2	Model 1c APOE x age x education	age_c	-0.29509043302525484	0.1846932708662698	-0.7015975813690951	0.11141671531858544	0.1384084164860208	17
Men	1	CVLT_2	Model 1c APOE x age x education	age_education	-0.34126545599235825	0.25269122589100756	-0.8974350942616341	0.21490418227691765	0.203977292708804	17

Sex	Cluster	Outcome	Model	Predictor	Beta	SE	CI_low	CI_high	p	N
Men	1	CVLT_2	Model 1c APOE x age x education	education_c	1.5183606135511596	0.3295497962113166	0.7930264025816918	2.2436948245206274	0.0007557707303528865	17
Men	1	CVLT_2	Model 2a PICALM	PICALM	0.23611111111111382	1.032789333109305	-1.9652272436032305	2.437449465825458	0.8222556864200743	17
Men	1	CVLT_2	Model 2b PICALM x age	PICALM	0.17011106768288675	1.0088882162579673	-2.0094594125936855	2.349681547959459	0.8686966611989256	17
Men	1	CVLT_2	Model 2b PICALM x age	PICALM_age	0.37198879551820707	0.30193356440719216	-0.28029901336064333	1.0242766043970575	0.23976064425159813	17
Men	1	CVLT_2	Model 2b PICALM x age	age_c	-0.43627450980392096	0.24771011694996978	-0.9714196823509276	0.09887066274308559	0.1016855744428164	17
Men	1	CVLT_2	Model 2c PICALM x age x education	PICALM	0.37295545667786945	1.0390830765529928	-1.9140509749086023	2.659961888264341	0.7264437087177935	17
Men	1	CVLT_2	Model 2c PICALM x age x education	PICALM_age	0.46153161571663803	0.32482493136444734	-0.253403237841473	1.176466469274749	0.1830784867785945	17
Men	1	CVLT_2	Model 2c PICALM x age x education	PICALM_education	-0.34310566477556803	0.34897362592739095	-1.1111914367020939	0.4249801071509577	0.3466473721696217	17
Men	1	CVLT_2	Model 2c PICALM x age x education	age_c	-0.5172240544037223	0.2603049919217914	-1.0901514787190971	0.05570336991165248	0.07239840053384264	17
Men	1	CVLT_2	Model 2c PICALM x age x education	age_education	0.162354926686109	0.27252376485070573	-0.4374658355202295	0.7621756888924476	0.5634018488894578	17
Men	1	CVLT_2	Model 2c PICALM x age x education	education_c	0.8919233819248029	0.8054017529614488	-0.880753924248139	2.664600688097745	0.29173786677340596	17
Men	1	CVLT_2	Model 3a APOE x PICALM	APOE	-0.4999999999999991	1.7556815150868283	-4.292919315921566	3.2929193159215675	0.780291512734981	17
Men	1	CVLT_2	Model 3a APOE x PICALM	APOE_PICALM	2.333333333333332	2.5057930439281653	-3.08010341834711	7.746770085013774	0.36873358289639757	17
Men	1	CVLT_2	Model 3a APOE x PICALM	PICALM	-0.3333333333333346	1.2182475052824788	-2.9651970595574237	2.298530392890755	0.788677871561978	17
Men	1	CVLT_2	Model 3b APOE x PICALM x age	APOE	0.030303030303303294	1.608890899773163	-3.510841964290202	3.571448024896268	0.9853102416917916	17
Men	1	CVLT_2	Model 3b APOE x PICALM x age	APOE_PICALM	1.3225055790462772	2.6711513944690832	-4.55665900051505	7.201670158607604	0.6302656328022813	17
Men	1	CVLT_2	Model 3b APOE x PICALM x age	APOE_PICALM_age	0.47853535353535237	0.38655489603396315	-0.37226623619280763	1.3293369432635123	0.2415061408159249	17
Men	1	CVLT_2	Model 3b APOE x PICALM x age	PICALM	-0.33333333333333015	1.1034631185474608	-2.7620392819551434	2.0953726152884826	0.7682264761586568	17
Men	1	CVLT_2	Model 3b APOE x PICALM x age	age_c	-0.3535353535353517	0.1627599562248266	-0.7117676018419454	0.004696894771242022	0.05258183169647548	17
Men	1	CVLT_2	Model 3c APOE x PICALM x age x education	APOE	0.38451852400954434	1.3160769327901833	-2.592654336368822	3.3616913843879104	0.7767796348324941	17
Men	1	CVLT_2	Model 3c APOE x PICALM x age x education	APOE_PICALM	0.8886408625350217	1.7737788738513063	-3.1239257222669883	4.901207447337032	0.6284032929543777	17
Men	1	CVLT_2	Model 3c APOE x PICALM x age x education	APOE_PICALM_age	0.4009655762149137	0.41926200253987617	-0.5474709659432208	1.3494021183730482	0.36387223771194643	17
Men	1	CVLT_2	Model 3c APOE x PICALM x age x education	APOE_PICALM_education	0.2324932489190472	0.4640700542052844	-0.817306148267527	1.2822926461056214	0.628403292954377	17

Sex	Cluster	Outcome	Model	Predictor	Beta	SE	CI_low	CI_high	p	N
Men	1	CVLT_2	Model 3c APOE x PICALM x age x education	APOE_age_education	0.4405332717091692	1.283517532550204	-2.4629851081980942	3.3440516516164327	0.7393115120714605	17
Men	1	CVLT_2	Model 3c APOE x PICALM x age x education	PICALM	-0.31297709923664296	1.2755174701804834	-3.1983980807509638	2.572443882277678	0.8116715830946357	17
Men	1	CVLT_2	Model 3c APOE x PICALM x age x education	age_c	-0.3343693665947967	0.1802357936449784	-0.7420910581914779	0.07335232500188454	0.09655204517303166	17
Men	1	CVLT_2	Model 3c APOE x PICALM x age x education	age_education	-0.21730100625717708	0.5419410624705714	-1.4432568625697408	1.008654850055387	0.6977963824647622	17
Men	1	CVLT_2	Model 3c APOE x PICALM x age x education	education_c	0.921429081715285	1.8214247701619553	-3.1989200087064633	5.041778172137033	0.6250960777970651	17
Men	1	CVLT_3	Model 1a APOE	APOE	1.7884615384615385	1.4908580720266496	-1.389227221653112	4.966150298576189	0.24889345025164114	17
Men	1	CVLT_3	Model 1b APOE x age	APOE	2.6503799563130097	1.7476257862861686	-1.1251360156026613	6.42589592822868	0.15331225487391045	17
Men	1	CVLT_3	Model 1b APOE x age	APOE_age	-0.08893458424366996	0.40636079503514455	-0.9668237090521742	0.7889545405648343	0.8301605663725717	17
Men	1	CVLT_3	Model 1b APOE x age	age_c	-0.22580645161290647	0.23555284888958647	-0.7346874432940663	0.2830745400682534	0.35523866336473686	17
Men	1	CVLT_3	Model 1c APOE x age x education	APOE	2.7382088050361717	2.3851744716521073	-2.5115248112788042	7.987942421351148	0.27531753805107606	17
Men	1	CVLT_3	Model 1c APOE x age x education	APOE_age	-0.35180268611603416	0.44883537171817817	-1.3396826785880585	0.63607730635599	0.44969866261351965	17
Men	1	CVLT_3	Model 1c APOE x age x education	APOE_age_education	1.728109087794082	0.5250907226572336	0.5723921995282795	2.8838259760598843	0.007191085405635729	17
Men	1	CVLT_3	Model 1c APOE x age x education	APOE_education	-0.17553375684575623	0.7602879447315725	-1.8489162405899124	1.4978487268984	0.8216475871079931	17
Men	1	CVLT_3	Model 1c APOE x age x education	age_c	-0.12393580634285473	0.23295261127587194	-0.6366610467636207	0.3887894340779112	0.6052896699667413	17
Men	1	CVLT_3	Model 1c APOE x age x education	age_education	-0.9237898068673089	0.31871805963322647	-1.6252835263704726	-0.2222960873641452	0.014485108045269781	17
Men	1	CVLT_3	Model 1c APOE x age x education	education_c	1.4903756306781508	0.4156593535475575	0.575515561870305	2.4052356994859965	0.004276698739172689	17
Men	1	CVLT_3	Model 2a PICALM	PICALM	0.9305555555555598	1.3044272113821604	-1.849765231360161	3.7108763424712805	0.48656083698588093	17
Men	1	CVLT_3	Model 2b PICALM x age	PICALM	1.0350791479382466	1.384035302804511	-1.9549473396761456	4.025105635552639	0.4678522047411795	17
Men	1	CVLT_3	Model 2b PICALM x age	PICALM_age	0.017086834733893497	0.4142051671404417	-0.8777490257005117	0.9119226951682987	0.9677217559636297	17
Men	1	CVLT_3	Model 2b PICALM x age	age_c	-0.1813725490196071	0.33981916053317246	-0.915507212300366	0.5527621142611518	0.6025336990901438	17
Men	1	CVLT_3	Model 2c PICALM x age x education	PICALM	1.521192252497491	1.3691697257788573	-1.4923299955766143	4.534714500571596	0.29024912537137737	17
Men	1	CVLT_3	Model 2c PICALM x age x education	PICALM_age	-0.17177946869576743	0.4280124200249305	-1.1138284535018295	0.7702695161102946	0.6958548148248594	17
Men	1	CVLT_3	Model 2c PICALM x age x education	PICALM_education	-1.1075417912306726	0.45983245661175165	-2.1196262043576244	-0.0954573781037209	0.03470177518951283	17

Sex	Cluster	Outcome	Model	Predictor	Beta	SE	CI_low	CI_high	p	N
Men	1	CVLT_3	Model 2c PICALM x age x education	age_c	-0.1242583089519943	0.3429963613599961	-0.8791882102677944	0.6306715923638058	0.7240119451276408	17
Men	1	CVLT_3	Model 2c PICALM x age x education	age_education	0.28962300445221123	0.3590966851530981	-0.5007434706047367	1.0799894795091591	0.4370265739007091	17
Men	1	CVLT_3	Model 2c PICALM x age x education	education_c	-1.3453306447198852	1.0612546023771172	-3.681136275621651	0.9904749861818805	0.23108942042172154	17
Men	1	CVLT_3	Model 3a APOE x PICALM	APOE	2.6428571428571432	2.1809795733864727	-2.068862767868704	7.35457705358299	0.2471642549315837	17
Men	1	CVLT_3	Model 3a APOE x PICALM	APOE_PICALM	-1.8095238095238066	3.1127988743852333	-8.534316931612786	4.915269312565172	0.570968853091665	17
Men	1	CVLT_3	Model 3a APOE x PICALM	PICALM	1.3095238095238075	1.513357007816257	-1.9598852361980579	4.578932855245673	0.4025548145395209	17
Men	1	CVLT_3	Model 3b APOE x PICALM x age	APOE	3.03174603174603	2.244391952496138	-1.9081273491075654	7.971619412599625	0.20388846651851475	17
Men	1	CVLT_3	Model 3b APOE x PICALM x age	APOE_PICALM	-1.1980358680940064	3.726238177175653	-9.399430798992157	7.003359062804144	0.7538479082406437	17
Men	1	CVLT_3	Model 3b APOE x PICALM x age	APOE_PICALM_age	0.009259259259257635	0.5392414724820236	-1.1776032193749542	1.1961217378934692	0.9866077707754908	17
Men	1	CVLT_3	Model 3b APOE x PICALM x age	PICALM	1.3095238095238124	1.539323607022321	-2.078504606097673	4.697552225145298	0.4130635035651635	17
Men	1	CVLT_3	Model 3b APOE x PICALM x age	age_c	-0.2592592592592564	0.22704904104506257	-0.7589908292105034	0.24047231069199065	0.27775755847856026	17
Men	1	CVLT_3	Model 3c APOE x PICALM x age x education	APOE	1.0083603652739321	1.7327490537526256	-2.9113903181012324	4.928111048649097	0.5748907407758317	17
Men	1	CVLT_3	Model 3c APOE x PICALM x age x education	APOE_PICALM	1.3820704681267504	2.335360181958486	-3.9008812953348917	6.6650222315883925	0.5685491579843616	17
Men	1	CVLT_3	Model 3c APOE x PICALM x age x education	APOE_PICALM_age	-0.28000171279189656	0.5520010419415355	-1.5287148237228674	0.9687113981390743	0.6241757695839825	17
Men	1	CVLT_3	Model 3c APOE x PICALM x age x education	APOE_PICALM_education	0.36158820387037194	0.6109953964426282	-1.0205794086631996	1.7437558164039435	0.568549157984361	17
Men	1	CVLT_3	Model 3c APOE x PICALM x age x education	APOE_age_education	1.456827581446479	1.689881293858857	-2.3659494918294874	5.279604654722446	0.4110198845434474	17
Men	1	CVLT_3	Model 3c APOE x PICALM x age x education	PICALM	0.6619411123227894	1.6793483985882844	-3.1370088964712592	4.460891121116838	0.7026354892844717	17
Men	1	CVLT_3	Model 3c APOE x PICALM x age x education	age_c	-0.2067836971366253	0.23729874227685374	-0.7435907467144789	0.3300233524412282	0.4061767062392153	17
Men	1	CVLT_3	Model 3c APOE x PICALM x age x education	age_education	-0.5517811257196913	0.713520493968937	-2.1658766219947174	1.0623143705553346	0.4591603969002803	17
Men	1	CVLT_3	Model 3c APOE x PICALM x age x education	education_c	0.2419202026708298	2.398090847385805	-5.182938184917825	5.666778590259485	0.9218570644951591	17
Men	1	CVLT_4	Model 1a APOE	APOE	-0.6153846153846134	0.8743552146697116	-2.479028640349794	1.2482594095805675	0.4923297330361911	17
Men	1	CVLT_4	Model 1b APOE x age	APOE	-0.8701747197832242	1.0637899378347557	-3.1683531585400395	1.4280037189735912	0.42809917894462446	17
Men	1	CVLT_4	Model 1b APOE x age	APOE_age	0.20045623955789743	0.24735416945726696	-0.3339199551825281	0.734832434298323	0.43229501726254793	17

Sex	Cluster	Outcome	Model	Predictor	Beta	SE	CI_low	CI_high	p	N
Men	1	CVLT_4	Model 1b APOE x age	age_c	-0.10483870967742143	0.1433823833702699	-0.4145975165992293	0.20492009724438642	0.4776407650496455	17
Men	1	CVLT_4	Model 1c APOE x age x education	APOE	-2.491516326756343	1.287044758207586	-5.324282739933787	0.34125008642110055	0.07899807274185956	17
Men	1	CVLT_4	Model 1c APOE x age x education	APOE_age	0.1398370854734905	0.2421924346976205	-0.39322486918033095	0.6728990401273119	0.5753097948163379	17
Men	1	CVLT_4	Model 1c APOE x age x education	APOE_age_education	0.9683177068614189	0.2833399695542262	0.34469063861421256	1.5919447751086253	0.005748443097038135	17
Men	1	CVLT_4	Model 1c APOE x age x education	APOE_education	-1.1084256340578384	0.4102528454941107	-2.011386058872179	-0.20546520924349798	0.020590007760396312	17
Men	1	CVLT_4	Model 1c APOE x age x education	age_c	-0.022627386834777663	0.12570167961159992	-0.29929491825741045	0.2540401445878551	0.8604190505322399	17
Men	1	CVLT_4	Model 1c APOE x age x education	age_education	0.02676467856848541	0.17198088142915013	-0.35176268927505927	0.4052920464120301	0.8791470516289794	17
Men	1	CVLT_4	Model 1c APOE x age x education	education_c	1.2768430459003681	0.22429059112509514	0.7831827832878013	1.770503308512935	0.0001396684807482927	17
Men	1	CVLT_4	Model 2a PICALM	PICALM	-1.1249999999999987	0.6971336818504545	-2.6109052693742476	0.36090526937425027	0.12741662216222102	17
Men	1	CVLT_4	Model 2b PICALM x age	PICALM	-1.1525755651097676	0.7493806027646358	-2.771513931082348	0.4663628008628127	0.1480171902012661	17
Men	1	CVLT_4	Model 2b PICALM x age	PICALM_age	0.11050420168067188	0.2242697980253566	-0.37400124056415046	0.5950096439254942	0.63042158554526	17
Men	1	CVLT_4	Model 2b PICALM x age	age_c	-0.1176470588235291	0.18399378024195537	-0.515141454642025	0.27984733699496683	0.533663447456524	17
Men	1	CVLT_4	Model 2c PICALM x age x education	PICALM	-0.7938823047868502	0.753913353953235	-2.453234408826284	0.8654697992525837	0.31491566604213106	17
Men	1	CVLT_4	Model 2c PICALM x age x education	PICALM_age	0.17114314955724685	0.2356788008375484	-0.3475823936323421	0.6898686927468358	0.4828995368032517	17
Men	1	CVLT_4	Model 2c PICALM x age x education	PICALM_education	-0.6154462588670171	0.2532000402094155	-1.1727357899003465	-0.05815672783368764	0.03336992856515341	17
Men	1	CVLT_4	Model 2c PICALM x age x education	age_c	-0.1985772793803171	0.1888659472364325	-0.6142684264927143	0.21711386773208002	0.31561591726254584	17
Men	1	CVLT_4	Model 2c PICALM x age x education	age_education	0.26482018255624284	0.1977313558720826	-0.1703835974012914	0.7000239625137771	0.20749131408730118	17
Men	1	CVLT_4	Model 2c PICALM x age x education	education_c	0.7101149003786005	0.5843643790920827	-0.5760624260841603	1.9962922268413612	0.24973185182070395	17
Men	1	CVLT_4	Model 3a APOE x PICALM	APOE	-0.6428571428571423	1.2150934611416777	-3.2679069709783506	1.982192685264066	0.6056802729893915	17
Men	1	CVLT_4	Model 3a APOE x PICALM	APOE_PICALM	0.14285714285714057	1.734239790353339	-3.603740143009731	3.889454428724012	0.9356037406016555	17
Men	1	CVLT_4	Model 3a APOE x PICALM	PICALM	-1.1428571428571443	0.843139581410751	-2.964349467558589	0.6786351818443004	0.19834800340345238	17
Men	1	CVLT_4	Model 3b APOE x PICALM x age	APOE	-0.4761904761904754	1.1862419780747313	-3.087091466200402	2.134710513819451	0.6957936827912413	17
Men	1	CVLT_4	Model 3b APOE x PICALM x age	APOE_PICALM	-1.5040259320782585	1.9694510761163646	-5.838758524119771	2.830706659963254	0.46112554758263624	17

Sex	Cluster	Outcome	Model	Predictor	Beta	SE	CI_low	CI_high	p	N
Men	1	CVLT_4	Model 3b APOE x PICALM x age	APOE_PICALM_age	0.4861111111111099	0.2850085388452698	-0.14118845338425362	1.1134106756064734	0.11612298238354765	17
Men	1	CVLT_4	Model 3b APOE x PICALM x age	PICALM	-1.1428571428571381	0.8135879646424773	-2.9335521794573216	0.6478378937430453	0.1877105080185719	17
Men	1	CVLT_4	Model 3b APOE x PICALM x age	age_c	-0.11111111111110938	0.1200035953032715	-0.3752372435302098	0.15301502130799105	0.37436632208554654	17
Men	1	CVLT_4	Model 3c APOE x PICALM x age x education	APOE	-1.5146733587913381	0.9074045601069124	-3.5673650840436633	0.538018366460987	0.1294120520426237	17
Men	1	CVLT_4	Model 3c APOE x PICALM x age x education	APOE_PICALM	-0.7887979144288426	1.2229794464535155	-3.555369629247004	1.9777738003893188	0.5350332146318612	17
Men	1	CVLT_4	Model 3c APOE x PICALM x age x education	APOE_PICALM_age	0.29976027153889495	0.2890714391427538	-0.35416475509442835	0.9536852981722183	0.32680395984417876	17
Men	1	CVLT_4	Model 3c APOE x PICALM x age x education	APOE_PICALM_education	-0.20637154737963892	0.31996555285121103	-0.9301839146285779	0.5174408198693	0.535033214631862	17
Men	1	CVLT_4	Model 3c APOE x PICALM x age x education	APOE_age_education	0.41158635482368044	0.8849556078336843	-1.590322312245192	2.413495021892553	0.6529128427042961	17
Men	1	CVLT_4	Model 3c APOE x PICALM x age x education	PICALM	-0.768811341330427	0.8794397501398381	-2.758242271407881	1.2206195887470273	0.40472961070489255	17
Men	1	CVLT_4	Model 3c APOE x PICALM x age x education	age_c	-0.07803372790626431	0.12426840481217938	-0.35914838996858867	0.20308093415606007	0.5456416523275474	17
Men	1	CVLT_4	Model 3c APOE x PICALM x age x education	age_education	0.17425885618048154	0.3736558092788796	-0.6710093092217811	1.019527021582744	0.6520383536049873	17
Men	1	CVLT_4	Model 3c APOE x PICALM x age x education	education_c	0.4321939100516121	1.2558301883102279	-2.408691345762782	3.2730791658660063	0.7386378327667245	17
Men	1	CVLT_5	Model 1a APOE	APOE	-0.6923076923076898	1.5677343292221717	-4.033854315886037	2.649238931270658	0.6650799594528727	17
Men	1	CVLT_5	Model 1b APOE x age	APOE	-0.20883684949738757	1.8970373781714949	-4.307136941434062	3.8894632424392865	0.9140226444615658	17
Men	1	CVLT_5	Model 1b APOE x age	APOE_age	0.03437861457396196	0.4411022217996303	-0.918564799697273	0.9873220288451969	0.9390642267688911	17
Men	1	CVLT_5	Model 1b APOE x age	age_c	-0.20967741935484174	0.2556912139800372	-0.76206470376978	0.3427098650600965	0.4269727787618113	17
Men	1	CVLT_5	Model 1c APOE x age x education	APOE	-0.21404965834108225	2.8382538537446265	-6.461004270981245	6.032904954299081	0.9412378538559351	17
Men	1	CVLT_5	Model 1c APOE x age x education	APOE_age	-0.2389166760353646	0.5340945656665692	-1.4144508891484313	0.9366175370777022	0.6633170846679141	17
Men	1	CVLT_5	Model 1c APOE x age x education	APOE_age_education	1.1913275490367645	0.6248351157788257	-0.18392526829114209	2.566580366364671	0.08301370635480064	17
Men	1	CVLT_5	Model 1c APOE x age x education	APOE_education	-0.6704161471661494	0.9047095777422477	-2.6616685019617474	1.3208362076294486	0.47419840743494024	17
Men	1	CVLT_5	Model 1c APOE x age x education	age_c	-0.08570680414227239	0.2772034727655154	-0.6958275340226296	0.5244139257380849	0.7629592849961893	17
Men	1	CVLT_5	Model 1c APOE x age x education	age_education	-0.49657621527842416	0.37926062506674213	-1.3313232228541068	0.3381707922972585	0.21711277382859762	17
Men	1	CVLT_5	Model 1c APOE x age x education	education_c	1.8739078639802909	0.4946165473732392	0.7852641832803267	2.962551544680255	0.003001950670147586	17

Sex	Cluster	Outcome	Model	Predictor	Beta	SE	CI_low	CI_high	p	N
Men	1	CVLT_5	Model 2a PICALM	PICALM	1.5972222222222263	1.2759598581737526	-1.1224218376341568	4.3168662820786095	0.22981874616040937	17
Men	1	CVLT_5	Model 2b PICALM x age	PICALM	1.7728161031854628	1.3179003656334094	-1.0743345390674632	4.619966745438389	0.20156335527386735	17
Men	1	CVLT_5	Model 2b PICALM x age	PICALM_age	-0.04677871148459345	0.39441272929635585	-0.8988556095656829	0.805298186596496	0.907402618072997	17
Men	1	CVLT_5	Model 2b PICALM x age	age_c	-0.19607843137254816	0.3235811940695582	-0.8951331008606498	0.5029762381155536	0.554972239068295	17
Men	1	CVLT_5	Model 2c PICALM x age x education	PICALM	2.071175227962908	1.4240428641949745	-1.0631219834522492	5.205472439378065	0.17375885611806025	17
Men	1	CVLT_5	Model 2c PICALM x age x education	PICALM_age	-0.06213456147376761	0.44516616241759477	-1.0419386787259695	0.9176695557784342	0.8915176786803485	17
Men	1	CVLT_5	Model 2c PICALM x age x education	PICALM_education	-0.38427154012747233	0.47826147206893044	-1.436917942790614	0.6683748625356694	0.4387174589601306	17
Men	1	CVLT_5	Model 2c PICALM x age x education	age_c	-0.21838973109064924	0.35674285783794374	-1.0035754671575445	0.5667960049762459	0.5528644069474339	17
Men	1	CVLT_5	Model 2c PICALM x age x education	age_education	0.20927953782527875	0.3734884451651489	-0.6127629874456682	1.0313220630962256	0.5864801485399813	17
Men	1	CVLT_5	Model 2c PICALM x age x education	education_c	-0.045651006583830256	1.1037872187463946	-2.4750702949338765	2.383768281766216	0.9677512304563807	17
Men	1	CVLT_5	Model 3a APOE x PICALM	APOE	-2.2857142857142825	2.1590050909461866	-6.949961213334182	2.378532641905617	0.3090172259224468	17
Men	1	CVLT_5	Model 3a APOE x PICALM	APOE_PICALM	3.1190476190476186	3.081435836858426	-3.5379897797970354	9.776085017892273	0.32990907900042943	17
Men	1	CVLT_5	Model 3a APOE x PICALM	PICALM	0.8809523809523787	1.4981091634989876	-2.3555156998278606	4.1174204617326176	0.5665793520510944	17
Men	1	CVLT_5	Model 3b APOE x PICALM x age	APOE	-1.810966810966809	2.179178196355246	-6.607305682321032	2.985372060387414	0.42362715377425064	17
Men	1	CVLT_5	Model 3b APOE x PICALM x age	APOE_PICALM	2.6571808897390277	3.617967432603184	-5.305911739083987	10.620273518562042	0.4780448187320404	17
Men	1	CVLT_5	Model 3b APOE x PICALM x age	APOE_PICALM_age	0.3164983164983156	0.5235731032168478	-0.8358783139005445	1.4688749468971758	0.5577759669616098	17
Men	1	CVLT_5	Model 3b APOE x PICALM x age	PICALM	0.8809523809523836	1.4945965377514538	-2.4086324189499213	4.170537180854689	0.5674849311954551	17
Men	1	CVLT_5	Model 3b APOE x PICALM x age	age_c	-0.31649831649831417	0.22045183293340964	-0.8017095292978342	0.16871289630120595	0.17891401047591812	17
Men	1	CVLT_5	Model 3c APOE x PICALM x age x education	APOE	-2.145739989348962	1.7078247733767755	-6.009108033342914	1.7176280546449894	0.24059605341697057	17
Men	1	CVLT_5	Model 3c APOE x PICALM x age x education	APOE_PICALM	2.723000167765978	2.3017677977481643	-2.483960343136938	7.929960678668894	0.26711475320852834	17
Men	1	CVLT_5	Model 3c APOE x PICALM x age x education	APOE_PICALM_age	0.631550977203972	0.5440609257964331	-0.5992003431154616	1.8623022975234056	0.2755824762111833	17
Men	1	CVLT_5	Model 3c APOE x PICALM x age x education	APOE_PICALM_education	0.7124128345899376	0.6022066912713231	-0.6498733455881531	2.0746990147680284	0.2671147532085282	17
Men	1	CVLT_5	Model 3c APOE x PICALM x age x education	APOE_age_education	-1.669907823301089	1.6655736336822	-5.437697148996207	2.0978815023940296	0.342245450119153	17

Sex	Cluster	Outcome	Model	Predictor	Beta	SE	CI_low	CI_high	p	N
Men	1	CVLT_5	Model 3c APOE x PICALM x age x education	PICALM	0.9585605234460153	1.655192246118023	-2.785744472010436	4.702865518902467	0.5767112400679099	17
Men	1	CVLT_5	Model 3c APOE x PICALM x age x education	age_c	-0.3878326376239332	0.23388537992496794	-0.9169181251080518	0.14125284986018533	0.1316497638818162	17
Men	1	CVLT_5	Model 3c APOE x PICALM x age x education	age_education	0.738362169795158	0.7032570430629435	-0.8525157874972732	2.3292401270875893	0.32112976271955573	17
Men	1	CVLT_5	Model 3c APOE x PICALM x age x education	education_c	-2.690574628510394	2.363596130151617	-8.037400544427104	2.656251287406316	0.28437270689617256	17
Men	1	CVLT_6	Model 1a APOE	APOE	0.5769230769230791	1.3684598809462287	-2.3398801144359256	3.4937262682820833	0.6793068715264287	17
Men	1	CVLT_6	Model 1b APOE x age	APOE	1.157831625773436	1.6807565131210058	-2.473222064315888	4.7888853158627605	0.5030115233562761	17
Men	1	CVLT_6	Model 1b APOE x age	APOE_age	-0.26352653900526923	0.3908122426962811	-1.1078250586875493	0.5807719806770109	0.5119307736642746	17
Men	1	CVLT_6	Model 1b APOE x age	age_c	0.048387096774190064	0.22653990806390628	-0.4410226200446325	0.5377968135930127	0.8341786711097839	17
Men	1	CVLT_6	Model 1c APOE x age x education	APOE	-0.014956687825053994	2.25838085096782	-4.985619426620728	4.955706050970619	0.9948344332328645	17
Men	1	CVLT_6	Model 1c APOE x age x education	APOE_age	-0.3747923836875549	0.42497570755201464	-1.3101576094052902	0.5605728420301805	0.39668521441565574	17
Men	1	CVLT_6	Model 1c APOE x age x education	APOE_age_education	1.9683334284438665	0.4971773961040931	0.8740533576900755	3.0626134991976572	0.0022378270327779275	17
Men	1	CVLT_6	Model 1c APOE x age x education	APOE_education	-1.0165028503448905	0.7198717561378888	-2.600929902767235	0.5679242020774542	0.18559077016861913	17
Men	1	CVLT_6	Model 1c APOE x age x education	age_c	0.14735657663053148	0.22056907062399325	-0.33811267458616556	0.6328258278472285	0.5178582900999686	17
Men	1	CVLT_6	Model 1c APOE x age x education	age_education	-0.8260073595655849	0.3017753088036085	-1.4902103359217764	-0.1618043832093934	0.01932933182719925	17
Men	1	CVLT_6	Model 1c APOE x age x education	education_c	1.4547878596753892	0.3935632951526846	0.5885608874909848	2.3210148318597934	0.0035233173523955526	17
Men	1	CVLT_6	Model 2a PICALM	PICALM	-0.583333333333329	1.1601005703529907	-3.0560291668153226	1.8893625001486645	0.622385930390675	17
Men	1	CVLT_6	Model 2b PICALM x age	PICALM	-0.6099480489870379	1.265456263880159	-3.34380009759599	2.1239039996219136	0.6378204402062995	17
Men	1	CVLT_6	Model 2b PICALM x age	PICALM_age	0.05840336134453761	0.3787175964567395	-0.7597662636908528	0.876572986379928	0.8798097721804872	17
Men	1	CVLT_6	Model 2b PICALM x age	age_c	-0.04411764705882304	0.31070470847949155	-0.7153543606727728	0.6271190665551267	0.8892631119337977	17
Men	1	CVLT_6	Model 2c PICALM x age x education	PICALM	-0.219454370269903	1.19702865613469	-2.8540966786163904	2.4151879380765844	0.8578726119882865	17
Men	1	CVLT_6	Model 2c PICALM x age x education	PICALM_age	-0.13513104614426075	0.3741998689460875	-0.9587394045995836	0.6884773123110621	0.7248496714983804	17
Men	1	CVLT_6	Model 2c PICALM x age x education	PICALM_education	-1.6039952467909968	0.40201928016773997	-2.488833716507422	-0.7191567770745718	0.002122735840039296	17
Men	1	CVLT_6	Model 2c PICALM x age x education	age_c	0.011846893569829153	0.2998725912262533	-0.6481682296347747	0.671862016774433	0.9691945407007099	17

Sex	Cluster	Outcome	Model	Predictor	Beta	SE	CI_low	CI_high	p	N
Men	1	CVLT_6	Model 2c PICALM x age x education	age_education	0.20181169389292664	0.3139486758712209	-0.4891846827273223	0.8928080705131756	0.5335123818139651	17
Men	1	CVLT_6	Model 2c PICALM x age x education	education_c	-1.1730118304507156	0.9278266577049756	-3.215144535188729	0.8691208742872978	0.23226955910239674	17
Men	1	CVLT_6	Model 3a APOE x PICALM	APOE	0.2142857142857153	2.037231938622243	-4.186886311855083	4.615457740426514	0.9178348519635189	17
Men	1	CVLT_6	Model 3a APOE x PICALM	APOE_PICALM	0.7857142857142829	2.9076353409208395	-5.49584996922943	7.067278540657996	0.7912299024331039	17
Men	1	CVLT_6	Model 3a APOE x PICALM	PICALM	-0.7857142857142876	1.4136121532187993	-3.839637673920544	2.2682091024919693	0.587772590997031	17
Men	1	CVLT_6	Model 3b APOE x PICALM x age	APOE	0.330447330447333	2.2220121508078208	-4.560168439004676	5.221063099899342	0.8844695377883999	17
Men	1	CVLT_6	Model 3b APOE x PICALM x age	APOE_PICALM	0.17779155061131924	3.6890822466547464	-7.941823728601243	8.297406829823881	0.9624255514582638	17
Men	1	CVLT_6	Model 3b APOE x PICALM x age	APOE_PICALM_age	0.20244107744107553	0.5338644628189644	-0.9725866827191202	1.3774688376012714	0.7117577059654769	17
Men	1	CVLT_6	Model 3b APOE x PICALM x age	PICALM	-0.7857142857142803	1.5239743463813749	-4.1399592064467985	2.5685306350182375	0.6163587321279115	17
Men	1	CVLT_6	Model 3b APOE x PICALM x age	age_c	-0.07744107744107431	0.22478503697640606	-0.572189608034841	0.41730745315269235	0.7369579071979742	17
Men	1	CVLT_6	Model 3c APOE x PICALM x age x education	APOE	-1.3447416248950765	1.5083467085989932	-4.756858935819694	2.067375686029541	0.3958523358422016	17
Men	1	CVLT_6	Model 3c APOE x PICALM x age x education	APOE_PICALM	2.4675629987128858	2.0329157509689337	-2.1312119288204077	7.066337926246179	0.25571271422319186	17
Men	1	CVLT_6	Model 3c APOE x PICALM x age x education	APOE_PICALM_age	0.3138932281536956	0.4805132935738983	-0.7731033607511132	1.4008898170585042	0.5299305330192974	17
Men	1	CVLT_6	Model 3c APOE x PICALM x age x education	APOE_PICALM_education	0.6455833426865127	0.5318674929860592	-0.5575845162611535	1.848751201634179	0.2557127142231913	17
Men	1	CVLT_6	Model 3c APOE x PICALM x age x education	APOE_age_education	-0.32809876450536013	1.4710306042267274	-3.655801182634445	2.999603653623725	0.8284842298068303	17
Men	1	CVLT_6	Model 3c APOE x PICALM x age x education	PICALM	-1.5681570338058917	1.4618617878427398	-4.8751181482770445	1.7388040806652616	0.3113197408738502	17
Men	1	CVLT_6	Model 3c APOE x PICALM x age x education	age_c	-0.11007796314762675	0.20656700177836165	-0.5773649858298431	0.35720905953458953	0.6070133957685333	17
Men	1	CVLT_6	Model 3c APOE x PICALM x age x education	age_education	0.026849307148822055	0.6211149192464784	-1.3782102563801446	1.4319088706777887	0.9664639690594907	17
Men	1	CVLT_6	Model 3c APOE x PICALM x age x education	education_c	-2.4536327685094035	2.08752238458423	-7.1759364834148895	2.2686709463960826	0.2699993693178757	17
Men	1	CVLT_7	Model 1a APOE	APOE	0.15384615384615552	1.5177451353432119	-3.0811510251560064	3.3888433328483174	0.9206033114184262	17
Men	1	CVLT_7	Model 1b APOE x age	APOE	0.5852792735802699	1.8853125436105596	-3.4876908532687687	4.658249400429309	0.7611463043958031	17
Men	1	CVLT_7	Model 1b APOE x age	APOE_age	-0.16742706592982926	0.43837594416558245	-1.1144807154516565	0.779626583591998	0.708686638357687	17
Men	1	CVLT_7	Model 1b APOE x age	age_c	0.008064516129029031	0.2541108881429731	-0.5409086818805204	0.5570377141385785	0.9751644969434683	17

Sex	Cluster	Outcome	Model	Predictor	Beta	SE	CI_low	CI_high	p	N
Men	1	CVLT_7	Model 1c APOE x age x education	APOE	-1.3443222974897697	2.457454221707873	-6.753142571051992	4.0644979760724524	0.5952757188404846	17
Men	1	CVLT_7	Model 1c APOE x age x education	APOE_age	-0.17579926319103256	0.4624367702194398	-1.1936157319207081	0.842017205538643	0.7110658431169146	17
Men	1	CVLT_7	Model 1c APOE x age x education	APOE_age_education	2.178893963797747	0.5410029448620844	0.9881545105951253	3.369633417000369	0.001990440617317272	17
Men	1	CVLT_7	Model 1c APOE x age x education	APOE_education	-1.4419707150821912	0.7833275266442304	-3.166062976710623	0.2821215465462408	0.09275598211101375	17
Men	1	CVLT_7	Model 1c APOE x age x education	age_c	0.09847139157923557	0.24001195084116383	-0.4297913504647243	0.6267341336231954	0.6894803452994767	17
Men	1	CVLT_7	Model 1c APOE x age x education	age_education	-0.8302684300361858	0.3283764145931433	-1.55302004547694	-0.1075168145954315	0.02805292649379724	17
Men	1	CVLT_7	Model 1c APOE x age x education	education_c	1.3216587258991068	0.4282553940216737	0.379074958931927	2.2642424928662868	0.010357058914964367	17
Men	1	CVLT_7	Model 2a PICALM	PICALM	0.6944444444444486	1.2777576487947544	-2.0290315154140797	3.417920404302977	0.5947803473957749	17
Men	1	CVLT_7	Model 2b PICALM x age	PICALM	0.6528442772457823	1.3858395013299192	-2.341079944312935	3.6467684988044997	0.6453892138138067	17
Men	1	CVLT_7	Model 2b PICALM x age	PICALM_age	0.1421568627450982	0.4147451160494449	-0.7538454863884079	1.0381592118786043	0.7372599712100978	17
Men	1	CVLT_7	Model 2b PICALM x age	age_c	-0.1421568627450972	0.3402621414507091	-0.8772485281155127	0.5929348026253183	0.6829215166611143	17
Men	1	CVLT_7	Model 2c PICALM x age x education	PICALM	1.095289036145695	1.3414970124290968	-1.8573259805063582	4.047904052797748	0.4315592916783675	17
Men	1	CVLT_7	Model 2c PICALM x age x education	PICALM_age	-0.06258621456859226	0.41936172845142994	-0.9855951555969249	0.8604227264597404	0.8840640146685672	17
Men	1	CVLT_7	Model 2c PICALM x age x education	PICALM_education	-1.2631330906717713	0.45053864042435776	-2.2547619522897304	-0.2715042290538121	0.017163684991521228	17
Men	1	CVLT_7	Model 2c PICALM x age x education	age_c	-0.07513038245377501	0.3360639556770362	-0.8148021617377055	0.6645413968301555	0.8271974998527685	17
Men	1	CVLT_7	Model 2c PICALM x age x education	age_education	0.24855671210391028	0.351838870839801	-0.5258354213548331	1.0229488455626536	0.4946000884616265	17
Men	1	CVLT_7	Model 2c PICALM x age x education	education_c	-1.4170133198908443	1.0398052569455345	-3.7056092598042034	0.8715826200225147	0.2002025181810835	17
Men	1	CVLT_7	Model 3a APOE x PICALM	APOE	-0.07142857142857117	2.262849234085413	-4.960017131043506	4.817159988186364	0.975297829989775	17
Men	1	CVLT_7	Model 3a APOE x PICALM	APOE_PICALM	0.40476190476190155	3.2296471891423764	-6.5724666540887	7.381990463612503	0.9021820761130893	17
Men	1	CVLT_7	Model 3a APOE x PICALM	PICALM	0.5952380952380931	1.5701654375045278	-2.7968981014050818	3.987374291881268	0.7107387279657902	17
Men	1	CVLT_7	Model 3b APOE x PICALM x age	APOE	0.16594516594516673	2.387984162361618	-5.089972537925865	5.421862869816199	0.9458454158380507	17
Men	1	CVLT_7	Model 3b APOE x PICALM x age	APOE_PICALM	-1.3109098319854162	3.9646362759349607	-10.037015440444792	7.4151957764739596	0.7471207958293526	17
Men	1	CVLT_7	Model 3b APOE x PICALM x age	APOE_PICALM_age	0.5332491582491568	0.5737411839066225	-0.7295466732577414	1.7960449897560549	0.37261591164947794	17

Sex	Cluster	Outcome	Model	Predictor	Beta	SE	CI_low	CI_high	p	N
Men	1	CVLT_7	Model 3b APOE x PICALM x age	PICALM	0.5952380952380998	1.6378067967274923	-3.0095503594421014	4.2000265499183005	0.723167241393384	17
Men	1	CVLT_7	Model 3b APOE x PICALM x age	age_c	-0.15824915824915553	0.24157523532910427	-0.6899526662520601	0.373454349753749	0.5258831963668165	17
Men	1	CVLT_7	Model 3c APOE x PICALM x age x education	APOE	-2.416908532192039	1.6177317425450668	-6.076471981166806	1.2426549167827274	0.16937984335003312	17
Men	1	CVLT_7	Model 3c APOE x PICALM x age x education	APOE_PICALM	1.8093948467533174	2.1803424381897987	-3.122882417272507	6.741672110779142	0.4280763939173138	17
Men	1	CVLT_7	Model 3c APOE x PICALM x age x education	APOE_PICALM_age	0.6068122434879815	0.5153600318135044	-0.559013143927654	1.772637630903617	0.26921261572919597	17
Men	1	CVLT_7	Model 3c APOE x PICALM x age x education	APOE_PICALM_education	0.47338818665057847	0.5704384285961692	-0.8170331905654824	1.7638095638666393	0.42807639391731334	17
Men	1	CVLT_7	Model 3c APOE x PICALM x age x education	APOE_age_education	-0.42649592371013156	1.5777094809476582	-3.9955227269386997	3.1425308795184366	0.7930062307021517	17
Men	1	CVLT_7	Model 3c APOE x PICALM x age x education	PICALM	-0.06761177753544478	1.5678757436368769	-3.614393121468803	3.4791695663979136	0.9665449273606638	17
Men	1	CVLT_7	Model 3c APOE x PICALM x age x education	age_c	-0.18870822721799385	0.2215472038584604	-0.6898828213367074	0.3124663669007197	0.4164277309686273	17
Men	1	CVLT_7	Model 3c APOE x PICALM x age x education	age_education	0.26174279485595076	0.6661580622711313	-1.245211437303696	1.7686970270155975	0.703527318694986	17
Men	1	CVLT_7	Model 3c APOE x PICALM x age x education	education_c	-3.024868474697966	2.2389091351231882	-8.089632811696362	2.0398958623004306	0.2096637470397872	17
Men	1	CVLT_8	Model 1a APOE	APOE	0.5961538461538476	1.166585655119152	-1.890364618305894	3.082672310613589	0.6167727020252401	17
Men	1	CVLT_8	Model 1b APOE x age	APOE	0.950059491389855	1.4428091745404417	-2.1669402265417017	4.067059209321412	0.5217221753473	17
Men	1	CVLT_8	Model 1b APOE x age	APOE_age	-0.18792571648888345	0.33548433986899845	-0.9126955690753814	0.5368441360976145	0.5848928857798141	17
Men	1	CVLT_8	Model 1b APOE x age	age_c	0.056451612903222204	0.19446829758060286	-0.3636716018652452	0.4765748276716896	0.7761755602611751	17
Men	1	CVLT_8	Model 1c APOE x age x education	APOE	-0.3923250361345145	1.866770141246899	-4.5010584143048895	3.7164083420358605	0.8373829523562978	17
Men	1	CVLT_8	Model 1c APOE x age x education	APOE_age	-0.2916940099930521	0.3512835141483758	-1.0648638116154159	0.4814757916293116	0.42398755178818426	17
Men	1	CVLT_8	Model 1c APOE x age x education	APOE_age_education	2.0080712545222017	0.41096519108026575	1.1035429676438833	2.91259954140052	0.00048207165464260677	17
Men	1	CVLT_8	Model 1c APOE x age x education	APOE_education	-1.124051970729406	0.595043612466553	-2.4337341313704384	0.1856301899116264	0.08552327057246863	17
Men	1	CVLT_8	Model 1c APOE x age x education	age_c	0.15887873503656125	0.18232166419007428	-0.24240854220741903	0.5601660122805415	0.4021438033232919	17
Men	1	CVLT_8	Model 1c APOE x age x education	age_education	-0.7983809428064087	0.24944647205927178	-1.347408926043912	-0.24935295956890557	0.0084454953589681	17
Men	1	CVLT_8	Model 1c APOE x age x education	education_c	1.511031523553302	0.32531811796355015	0.795011173609414	2.22705187349719	0.0007110917955694951	17
Men	1	CVLT_8	Model 2a PICALM	PICALM	-0.08333333333332904	0.9997684917204771	-2.21428943067547	2.047622764008812	0.9346733501331914	17

Sex	Cluster	Outcome	Model	Predictor	Beta	SE	CI_low	CI_high	p	N
Men	1	CVLT_8	Model 2b PICALM x age	PICALM	-0.14420884632923103	1.087437397296853	-2.4934745153128937	2.2050568226544316	0.8965297530280536	17
Men	1	CVLT_8	Model 2b PICALM x age	PICALM_age	0.09411764705882364	0.3254412571625928	-0.6089554444345099	0.7971907385521572	0.7769887679884644	17
Men	1	CVLT_8	Model 2b PICALM x age	age_c	-0.04411764705882282	0.26699612555619134	-0.6209277081069478	0.5326924139893021	0.8712992706135191	17
Men	1	CVLT_8	Model 2c PICALM x age x education	PICALM	0.28173562309718525	1.0167470485393355	-1.95610954229603	2.5195807884904005	0.7868450740016351	17
Men	1	CVLT_8	Model 2c PICALM x age x education	PICALM_age	-0.0833689316068158	0.3178425264632348	-0.7829356155956676	0.616197752382036	0.7979401692658432	17
Men	1	CVLT_8	Model 2c PICALM x age x education	PICALM_education	-1.4405309395002073	0.34147212305372326	-2.192106014923471	-0.6889558640769438	0.0014397912694914632	17
Men	1	CVLT_8	Model 2c PICALM x age x education	age_c	0.0026469648525505596	0.2547095013177603	-0.5579648676799682	0.5632587973850693	0.9918945394240777	17
Men	1	CVLT_8	Model 2c PICALM x age x education	age_education	0.2369758658789698	0.2666656207008791	-0.3499512079879735	0.8239029397459131	0.3932024403833675	17
Men	1	CVLT_8	Model 2c PICALM x age x education	education_c	-1.1012523864559411	0.7880889157857409	-2.8358243949262176	0.6333196220143353	0.18984857521053267	17
Men	1	CVLT_8	Model 3a APOE x PICALM	APOE	0.2142857142857153	1.7499719665841662	-3.566298872008156	3.9948703005795867	0.9044146341600399	17
Men	1	CVLT_8	Model 3a APOE x PICALM	APOE_PICALM	0.7857142857142829	2.4976441018797355	-4.610117746981414	6.18154631840998	0.7580708334154415	17
Men	1	CVLT_8	Model 3a APOE x PICALM	PICALM	-0.28571428571428825	1.214285714285714	-2.9090190828455174	2.337590511416941	0.8176463469656278	17
Men	1	CVLT_8	Model 3b APOE x PICALM x age	APOE	0.3203463203463217	1.8816094967935288	-3.8210482591673802	4.461740899860024	0.8679035075116083	17
Men	1	CVLT_8	Model 3b APOE x PICALM x age	APOE_PICALM	-0.3072942883989409	3.1239307972435713	-7.183019614258137	6.568431037460255	0.9234098433266931	17
Men	1	CVLT_8	Model 3b APOE x PICALM x age	APOE_PICALM_age	0.32070707070706883	0.45207873542705	-0.6743115171569339	1.3157256585710715	0.49283592947825394	17
Men	1	CVLT_8	Model 3b APOE x PICALM x age	PICALM	-0.28571428571428026	1.2905080658440176	-3.126103387604312	2.5546748161757513	0.8288397783691696	17
Men	1	CVLT_8	Model 3b APOE x PICALM x age	age_c	-0.07070707070706783	0.19034894123244214	-0.48966226559717435	0.3482481241830387	0.717350310466532	17
Men	1	CVLT_8	Model 3c APOE x PICALM x age x education	APOE	-1.5972854629798188	1.2208264674843998	-4.358986801001521	1.1644158750418834	0.22317468242718216	17
Men	1	CVLT_8	Model 3c APOE x PICALM x age x education	APOE_PICALM	2.121600356097275	1.6454024401684273	-1.6005585597073462	5.843759271901897	0.22940473669824624	17
Men	1	CVLT_8	Model 3c APOE x PICALM x age x education	APOE_PICALM_age	0.3882362048079344	0.3889181071094679	-0.49155767695340613	1.2680300865692749	0.3442405847932113	17
Men	1	CVLT_8	Model 3c APOE x PICALM x age x education	APOE_PICALM_education	0.5550698606068472	0.43048319655569395	-0.4187507859699451	1.5288905071836394	0.22940473669824551	17
Men	1	CVLT_8	Model 3c APOE x PICALM x age x education	APOE_age_education	-0.2584797568672439	1.1906235389260267	-2.951857323711452	2.434897809976964	0.8329750025739826	17
Men	1	CVLT_8	Model 3c APOE x PICALM x age x education	PICALM	-0.9422028353326093	1.1832024774066408	-3.618792794704757	1.7343871240395385	0.4463441099700296	17

Sex	Cluster	Outcome	Model	Predictor	Beta	SE	CI_low	CI_high	p	N
Men	1	CVLT_8	Model 3c APOE x PICALM x age x education	age_c	-0.0956478966031562	0.16719131061992845	-0.473860917488992	0.2825651242826796	0.5812692503102184	17
Men	1	CVLT_8	Model 3c APOE x PICALM x age x education	age_education	0.09569777884008523	0.5027183262592513	-1.041530083805304	1.2329256414854746	0.8532510972968625	17
Men	1	CVLT_8	Model 3c APOE x PICALM x age x education	education_c	-2.348362087169499	1.689599986553302	-6.170502799109241	1.473778624770243	0.1979765080618947	17
Men	1	CVLT_9	Model 1a APOE	APOE	2.46153846153846	1.741986442337021	-1.2514177493512855	6.174494672428205	0.17805104471091668	17
Men	1	CVLT_9	Model 1b APOE x age	APOE	3.1965173382297416	2.143500696112233	-1.4342343807534887	7.8272690572129715	0.15975553939233894	17
Men	1	CVLT_9	Model 1b APOE x age	APOE_age	-0.24672278627425742	0.49841027402185717	-1.323472720329222	0.8300271477807073	0.6288474724341369	17
Men	1	CVLT_9	Model 1b APOE x age	age_c	-0.024193548387097558	0.28891064639130426	-0.6483470533687622	0.5999599565945671	0.9345384649344369	17
Men	1	CVLT_9	Model 1c APOE x age x education	APOE	4.27301220898558	3.125655404737686	-2.6065089523751297	11.15253337034629	0.1988879595934942	17
Men	1	CVLT_9	Model 1c APOE x age x education	APOE_age	-0.5404376226428367	0.5881769749433216	-1.835006415995568	0.7541311707098945	0.37788970072928696	17
Men	1	CVLT_9	Model 1c APOE x age x education	APOE_age_education	-0.05483184415698983	0.6881059120653654	-1.5693427451782016	1.4596790568642222	0.9379188595161346	17
Men	1	CVLT_9	Model 1c APOE x age x education	APOE_education	1.0755215318228983	0.9963204586711504	-1.1173650123993406	3.2684080760451373	0.3034688062455676	17
Men	1	CVLT_9	Model 1c APOE x age x education	age_c	0.0148719448399115	0.3052730930739177	-0.6570296027884022	0.6867734924682252	0.962018240885056	17
Men	1	CVLT_9	Model 1c APOE x age x education	age_education	0.07454501083411517	0.4176645513860777	-0.8447284686593441	0.9938184903275744	0.8615902223592008	17
Men	1	CVLT_9	Model 1c APOE x age x education	education_c	0.6126662652440026	0.544701412993822	-0.5862134614316131	1.8115459919196182	0.2846291792567313	17
Men	1	CVLT_9	Model 2a PICALM	PICALM	1.4305555555555567	1.5319597645384198	-1.8347393883850254	4.695850499496139	0.36519261353903376	17
Men	1	CVLT_9	Model 2b PICALM x age	PICALM	1.0592306690117907	1.521850635973643	-2.228527744760926	4.346989082784507	0.4986748156243097	17
Men	1	CVLT_9	Model 2b PICALM x age	PICALM_age	0.748179271708683	0.45544965201316523	-0.23575988109663448	1.7321184245140007	0.12439491617269237	17
Men	1	CVLT_9	Model 2b PICALM x age	age_c	-0.5196078431372542	0.3736566578363383	-1.3268439750048566	0.2876282887303483	0.18769334124176817	17
Men	1	CVLT_9	Model 2c PICALM x age x education	PICALM	1.6294261906467802	1.5310038633074403	-1.7402905925225594	4.99914297381612	0.3100040918938054	17
Men	1	CVLT_9	Model 2c PICALM x age x education	PICALM_age	0.7853134872958659	0.4786029491186507	-0.26808450128621886	1.8387114758779508	0.1290837290384891	17
Men	1	CVLT_9	Model 2c PICALM x age x education	PICALM_education	0.3429084258407007	0.514184073962245	-0.7888030905011938	1.4746199421825952	0.5185813162500224	17
Men	1	CVLT_9	Model 2c PICALM x age x education	age_c	-0.5796362850137501	0.3835380993717396	-1.4237979500573683	0.26452538002986814	0.158897754934188	17
Men	1	CVLT_9	Model 2c PICALM x age x education	age_education	0.44723830779217993	0.401541461163659	-0.4365484893870573	1.3310251049714172	0.28910910529455963	17

Sex	Cluster	Outcome	Model	Predictor	Beta	SE	CI_low	CI_high	p	N
Men	1	CVLT_9	Model 2c PICALM x age x education	education_c	0.08237514431938231	1.1866935600463286	-2.529519770908591	2.694270059547356	0.9459045798711512	17
Men	1	CVLT_9	Model 3a APOE x PICALM	APOE	0.07142857142857428	2.374808923983508	-5.0590341930293015	5.201891335886449	0.9764620192683275	17
Men	1	CVLT_9	Model 3a APOE x PICALM	APOE_PICALM	4.761904761904763	3.3894414398286274	-2.56053828761109	12.084347811420615	0.18348684187397737	17
Men	1	CVLT_9	Model 3a APOE x PICALM	PICALM	0.23809523809523903	1.6478529974284069	-3.321874728004421	3.798065204194899	0.8873309766821991	17
Men	1	CVLT_9	Model 3b APOE x PICALM x age	APOE	0.27849927849928147	2.526606409801973	-5.282523934845319	5.839522491843882	0.9142149808154489	17
Men	1	CVLT_9	Model 3b APOE x PICALM x age	APOE_PICALM	6.045190624405741	4.194783024609435	-3.1874645625274916	15.277845811338974	0.1774050830772736	17
Men	1	CVLT_9	Model 3b APOE x PICALM x age	APOE_PICALM_age	-0.23695286195286228	0.6070468036070359	-1.5730538681677366	1.0991481442620121	0.703740206220867	17
Men	1	CVLT_9	Model 3b APOE x PICALM x age	PICALM	0.23809523809523747	1.7328813213470038	-3.5759508343744493	4.052141310564925	0.8931985346988305	17
Men	1	CVLT_9	Model 3b APOE x PICALM x age	age_c	-0.13804713804713753	0.2555986541503309	-0.7006159827691899	0.42452170667491484	0.5998963416148384	17
Men	1	CVLT_9	Model 3c APOE x PICALM x age x education	APOE	0.5642583434767114	1.9643251602469616	-3.8793538879504785	5.007870574903902	0.780422669436219	17
Men	1	CVLT_9	Model 3c APOE x PICALM x age x education	APOE_PICALM	5.035682182705874	2.6474732470492484	-0.9533183865711834	11.024682751982931	0.08959088950703224	17
Men	1	CVLT_9	Model 3c APOE x PICALM x age x education	APOE_PICALM_age	0.22886950792254274	0.6257741320475694	-1.1867299272176735	1.6444689430627588	0.7230090867918046	17
Men	1	CVLT_9	Model 3c APOE x PICALM x age x education	APOE_PICALM_education	1.3174749896614224	0.6926528844024208	-0.2494146941610662	2.884364673483911	0.08959088950703228	17
Men	1	CVLT_9	Model 3c APOE x PICALM x age x education	APOE_age_education	-2.490481382953475	1.9157282678461898	-6.824159806143809	1.8431970402368592	0.2259004379803892	17
Men	1	CVLT_9	Model 3c APOE x PICALM x age x education	PICALM	0.6095965103598678	1.9037876864068732	-3.697070440998874	4.91626346171861	0.7561242203943372	17
Men	1	CVLT_9	Model 3c APOE x PICALM x age x education	age_c	-0.23935170884057103	0.269012924254599	-0.8479012223434393	0.36919780466229735	0.39676445174856056	17
Men	1	CVLT_9	Model 3c APOE x PICALM x age x education	age_education	1.0972133509068276	0.8088801177640808	-0.732600601383455	2.92702730319711	0.20800045256598024	17
Men	1	CVLT_9	Model 3c APOE x PICALM x age x education	education_c	-3.2296955551104762	2.7185876557693405	-9.379568093455823	2.920176983234872	0.2652359568457788	17
Men	1	RPM	Model 1a APOE	APOE	0.21153846153846856	1.7714520615335978	-3.564222229997444	3.9872991530743818	0.9065310133674596	17
Men	1	RPM	Model 1b APOE x age	APOE	1.1241081751820108	2.1468230483042463	-3.5138210493425692	5.762037399706591	0.6093573153107181	17
Men	1	RPM	Model 1b APOE x age	APOE_age	-0.45961316026217736	0.4991827927662745	-1.53803201959911	0.6188056990747552	0.3739613254218973	17
Men	1	RPM	Model 1b APOE x age	age_c	0.12096774193546889	0.28935844793439447	-0.5041531794642028	0.7460886633351406	0.6827299755658376	17
Men	1	RPM	Model 1c APOE x age x education	APOE	1.3704706902715529	3.064552986660763	-5.374564955656665	8.115506336199772	0.6634076984663306	17

Sex	Cluster	Outcome	Model	Predictor	Beta	SE	CI_low	CI_high	p	N
Men	1	RPM	Model 1c APOE x age x education	APOE_age	-1.7178848242128388	0.5766788950936578	-2.9871465144470113	-0.4486231339786664	0.012543530278269079	17
Men	1	RPM	Model 1c APOE x age x education	APOE_age_education	5.747089931277043	0.6746543540156548	4.262185709903239	7.231994152650847	3.5775537340149624e-06	17
Men	1	RPM	Model 1c APOE x age x education	APOE_education	-2.6779260669427583	0.9768437149737991	-4.827944587320388	-0.5279075465651286	0.01918313355669597	17
Men	1	RPM	Model 1c APOE x age x education	age_c	0.6741091810877506	0.2993054089420185	0.015342417673809505	1.3328759445016918	0.04570960085566991	17
Men	1	RPM	Model 1c APOE x age x education	age_education	-2.5765950921576137	0.40949976329202853	-3.4778979942208488	-1.6752921900943787	5.9025190823307546e-05	17
Men	1	RPM	Model 1c APOE x age x education	education_c	8.326518940461332	0.5340532227251854	7.151075722548725	9.501962158373939	7.581814044454286e-09	17
Men	1	RPM	Model 2a PICALM	PICALM	-0.8749999999999822	1.4891194887757844	-4.0489830576346595	2.2989830576346946	0.5655451666774584	17
Men	1	RPM	Model 2b PICALM x age	PICALM	-1.274053807569545	1.449665914298381	-4.405866611159664	1.857758996020574	0.39543148260057126	17
Men	1	RPM	Model 2b PICALM x age	PICALM_age	0.7941176470588225	0.4338466736455598	-0.14315110839491496	1.7313864025125598	0.09020243998144963	17
Men	1	RPM	Model 2b PICALM x age	age_c	-0.5441176470588207	0.3559333010163909	-1.3130647943653344	0.224829500247693	0.1502981690100467	17
Men	1	RPM	Model 2c PICALM x age x education	PICALM	-0.3516408453764601	1.4848606185571231	-3.619797031612276	2.916515340859356	0.8171507818181865	17
Men	1	RPM	Model 2c PICALM x age x education	PICALM_age	0.6805321941885865	0.4641782350152541	-0.3411172127134834	1.7021816010906563	0.1706156572867123	17
Men	1	RPM	Model 2c PICALM x age x education	PICALM_education	-4.646290361746796	0.4986869729162036	-5.743892988662047	-3.548687734831544	1.4919717605106704e-06	17
Men	1	RPM	Model 2c PICALM x age x education	age_c	-0.6513795429829718	0.3719785646020825	-1.470098843541112	0.16733975757516828	0.10772243617998775	17
Men	1	RPM	Model 2c PICALM x age x education	age_education	0.5284154563219357	0.3894393193180809	-0.32873470624996526	1.3855656188938366	0.20201589857073854	17
Men	1	RPM	Model 2c PICALM x age x education	education_c	0.6268210413278457	1.1509275553371379	-1.9063534282997416	3.159995510955433	0.5968810306242542	17
Men	1	RPM	Model 3a APOE x PICALM	APOE	-1.2857142857142811	2.5670750692768958	-6.831542804162569	4.260114232734007	0.6248506279919924	17
Men	1	RPM	Model 3a APOE x PICALM	APOE_PICALM	3.119047619047606	3.6638529235282924	-4.796225398825965	11.034320636921176	0.4100089777300867	17
Men	1	RPM	Model 3a APOE x PICALM	PICALM	-1.6190476190476275	1.7812642965969603	-5.467235174288773	2.2291399361935182	0.37992874540952404	17
Men	1	RPM	Model 3b APOE x PICALM x age	APOE	-1.2251082251082224	2.815524584362206	-7.42203605313815	4.971819602921705	0.6718850058749417	17
Men	1	RPM	Model 3b APOE x PICALM x age	APOE_PICALM	3.554998699620782	4.674457678106969	-6.733413281328454	13.843410680570019	0.46293675795322764	17
Men	1	RPM	Model 3b APOE x PICALM x age	APOE_PICALM_age	-0.08459595959596777	0.6764627813748257	-1.5734805027503955	1.40428858355846	0.9027351468813885	17
Men	1	RPM	Model 3b APOE x PICALM x age	PICALM	-1.6190476190475955	1.9310368022128745	-5.869230964292165	2.631135726196974	0.4196331171226081	17

Sex	Cluster	Outcome	Model	Predictor	Beta	SE	CI_low	CI_high	p	N
Men	1	RPM	Model 3b APOE x PICALM x age	age_c	-0.04040404040402912	0.28482643426308457	-0.6673027954164198	0.5864947146083616	0.8897593056557982	17
Men	1	RPM	Model 3c APOE x PICALM x age x education	APOE	-3.72720451905346	1.9477786258109757	-8.133385889085872	0.6789768509789527	0.08795798957782192	17
Men	1	RPM	Model 3c APOE x PICALM x age x education	APOE_PICALM	6.522205005968615	2.6251722002891773	0.5836529093589968	12.460757102578235	0.03473480864300203	17
Men	1	RPM	Model 3c APOE x PICALM x age x education	APOE_PICALM_age	-0.012835550661769446	0.6205029104419914	-1.416510654089936	1.390839552766397	0.9839477075371637	17
Men	1	RPM	Model 3c APOE x PICALM x age x education	APOE_PICALM_education	1.706390844584817	0.6868183082151929	0.15269988907648413	3.26008180009315	0.03473480864300188	17
Men	1	RPM	Model 3c APOE x PICALM x age x education	APOE_age_education	-0.21718225148246262	1.8995910903587703	-4.5143558430313835	4.079991340066458	0.9114853425013701	17
Men	1	RPM	Model 3c APOE x PICALM x age x education	PICALM	-2.529989094874603	1.8877510906591626	-6.800378746294865	1.7404005565456595	0.2130255028742749	17
Men	1	RPM	Model 3c APOE x PICALM x age x education	age_c	-0.06920121415505398	0.266746888210778	-0.6726245979901072	0.5342221696799992	0.8011428416350517	17
Men	1	RPM	Model 3c APOE x PICALM x age x education	age_education	0.05296721922673697	0.8020664990241543	-1.761433256626064	1.867367695079538	0.9487909151313041	17
Men	1	RPM	Model 3c APOE x PICALM x age x education	education_c	-2.942537661744968	2.695687575286851	-9.040606618996918	3.1555312955069814	0.3033795337637473	17
Women	0	CVLT_1	Model 1a APOE	APOE	-1.2657142857142905	2.673739752848891	-6.610443895619089	4.079015324190508	0.637599517717848	64
Women	0	CVLT_1	Model 1b APOE x age	APOE	0.02483167175758738	2.6884797335993125	-5.3529284838905955	5.40259182740577	0.9926612262495939	64
Women	0	CVLT_1	Model 1b APOE x age	APOE_age	-1.1155922643029923	0.8021064949868594	-2.7200441392486994	0.48885961064271455	0.16941438812669735	64
Women	0	CVLT_1	Model 1b APOE x age	age_c	-0.30205479452054895	0.3584319166829945	-1.0190253768019395	0.4149157877608417	0.40273650858537413	64
Women	0	CVLT_1	Model 1c APOE x age x education	APOE	0.36719744082045835	2.8255784528976875	-5.293116370323363	6.027511251964279	0.8970678789782853	64
Women	0	CVLT_1	Model 1c APOE x age x education	APOE_age	-1.4166926446207926	0.8418969026574147	-3.103214801096029	0.2698295118544436	0.09799379836596178	64
Women	0	CVLT_1	Model 1c APOE x age x education	APOE_age_education	1.0094318761402281	1.4022428442622996	-1.7995980871990653	3.8184618394795216	0.4745992103168094	64
Women	0	CVLT_1	Model 1c APOE x age x education	APOE_education	5.52140382259406	4.238207402947874	-2.968745821913611	14.01155346710173	0.19798567837861825	64
Women	0	CVLT_1	Model 1c APOE x age x education	age_c	-0.3172085810250478	0.36391490455655406	-1.0462177360283738	0.4118005739782782	0.38711788885190246	64
Women	0	CVLT_1	Model 1c APOE x age x education	age_education	-0.34767799328565974	0.5646062130588614	-1.4787201493996678	0.7833641628283483	0.5405290011244046	64
Women	0	CVLT_1	Model 1c APOE x age x education	education_c	-0.02125225463849567	1.8910712467931778	-3.8095231784570025	3.767018669180011	0.9910733364880191	64
Women	0	CVLT_1	Model 2a PICALM	PICALM	-1.0508308895405416	2.2116916734826293	-5.471939549292205	3.3702777702111217	0.6363671518876592	64
Women	0	CVLT_1	Model 2b PICALM x age	PICALM	-1.579090702660674	2.2098889208264163	-5.999526697883198	2.8413452925618503	0.47765404419934654	64

Sex	Cluster	Outcome	Model	Predictor	Beta	SE	CI_low	CI_high	p	N
Women	0	CVLT_1	Model 2b PICALM x age	PICALM_age	-0.3703943246832566	0.6688264539718698	-1.7082464238687085	0.9674577745021953	0.5817756100054656	64
Women	0	CVLT_1	Model 2b PICALM x age	age_c	-0.4245112003107572	0.4044060122524907	-1.2334436658288865	0.3844212652073721	0.29805883770079666	64
Women	0	CVLT_1	Model 2c PICALM x age x education	PICALM	-1.71174305193414	2.2744544817740278	-6.266259590416258	2.842773486547978	0.4547902899124504	64
Women	0	CVLT_1	Model 2c PICALM x age x education	PICALM_age	-0.34420815284721784	0.6913977090669874	-1.7287081838867928	1.0402918781923571	0.620509389737051	64
Women	0	CVLT_1	Model 2c PICALM x age x education	PICALM_education	-3.1760070554838453	3.3622065928333824	-9.908709624633197	3.556695513665505	0.3488400457237225	64
Women	0	CVLT_1	Model 2c PICALM x age x education	age_c	-0.43100612203852884	0.40895772176407086	-1.249929834181423	0.3879175901043651	0.2963695354994237	64
Women	0	CVLT_1	Model 2c PICALM x age x education	age_education	-0.32781671938105017	0.5483629594635998	-1.4258946048615033	0.770261166099403	0.5523347246095802	64
Women	0	CVLT_1	Model 2c PICALM x age x education	education_c	2.2681291944453594	2.11265740686766	-1.962394290122437	6.498652679013156	0.28753025886674616	64
Women	0	CVLT_1	Model 3a APOE x PICALM	APOE	0.3194444444444846	3.4856103075542615	-6.65281426214676	7.291703151035729	0.9272840987853279	64
Women	0	CVLT_1	Model 3a APOE x PICALM	APOE_PICALM	-4.565598290598286	5.577890475839716	-15.723040460854556	6.591843879657983	0.4163010939903473	64
Women	0	CVLT_1	Model 3a APOE x PICALM	PICALM	-0.27884615384613287	2.524302771852753	-5.328203490487754	4.770511182795488	0.9124095748996677	64
Women	0	CVLT_1	Model 3b APOE x PICALM x age	APOE	0.3844953310849055	3.473661974745682	-6.568794577773992	7.337785239943803	0.9122452975694181	64
Women	0	CVLT_1	Model 3b APOE x PICALM x age	APOE_PICALM	-5.114393997719976	10.875381591659318	-26.883835476495673	16.65504748105572	0.6399238819045949	64
Women	0	CVLT_1	Model 3b APOE x PICALM x age	APOE_PICALM_age	1.0918356645520735	5.3212065288216825	-9.559716480938068	11.743387810042215	0.8381454406466318	64
Women	0	CVLT_1	Model 3b APOE x PICALM x age	PICALM	-0.8876557339425037	2.5458447330163683	-5.983717647940428	4.20840618005542	0.7286007648977719	64
Women	0	CVLT_1	Model 3b APOE x PICALM x age	age_c	-0.5204070931234652	0.33517603386907724	-1.1913348203856529	0.1505206341387224	0.12594966742685673	64
Women	0	CVLT_1	Model 3c APOE x PICALM x age x education	APOE	0.6383193824240561	3.632199226480263	-6.640770529270586	7.917409294118698	0.8611443669884371	64
Women	0	CVLT_1	Model 3c APOE x PICALM x age x education	APOE_PICALM	-5.436323332044523	10.420885623036927	-26.32024480214454	15.447598138055493	0.6039906739651499	64
Women	0	CVLT_1	Model 3c APOE x PICALM x age x education	APOE_PICALM_age	1.1004547972928265	5.433534043730344	-9.788590757868947	11.9895003524546	0.8402495842071863	64
Women	0	CVLT_1	Model 3c APOE x PICALM x age x education	APOE_PICALM_education	-1.4222938950116468	2.7263944943991936	-6.886110558700599	4.041522768677305	0.6039906739651499	64
Women	0	CVLT_1	Model 3c APOE x PICALM x age x education	APOE_age_education	0.28903890697854306	1.3123686226885176	-2.341006585072742	2.919084399029828	0.8264980347340806	64
Women	0	CVLT_1	Model 3c APOE x PICALM x age x education	PICALM	-0.7975498784339052	2.6314396382404204	-6.071072757989897	4.475973001122086	0.7629685442298316	64
Women	0	CVLT_1	Model 3c APOE x PICALM x age x education	age_c	-0.5485274356980656	0.3433692438807168	-1.2366547776391545	0.13959990624302343	0.1158875820411043	64

Sex	Cluster	Outcome	Model	Predictor	Beta	SE	CI_low	CI_high	p	N
Women	0	CVLT_1	Model 3c APOE x PICALM x age x education	age_education	-0.21450094939148823	0.583427169636829	-1.3837151251313269	0.9547132263483504	0.7145402445605218	64
Women	0	CVLT_1	Model 3c APOE x PICALM x age x education	education_c	1.1601148576695373	1.7726347925345807	-2.3923246509862266	4.712554366325302	0.5155443850996055	64
Women	0	CVLT_10	Model 1a APOE	APOE	-0.011428571428571371	0.5345205132314141	-1.0799198526482314	1.0570627097910887	0.9830104100055428	64
Women	0	CVLT_10	Model 1b APOE x age	APOE	-0.1518364706507008	0.5474666258109384	-1.2469327698858166	0.943259828584415	0.7824694802628075	64
Women	0	CVLT_10	Model 1b APOE x age	APOE_age	0.2463268869191511	0.1633363758943407	-0.08039451003800102	0.5730482838763032	0.1367778802477184	64
Women	0	CVLT_10	Model 1b APOE x age	age_c	-0.04828767123287677	0.0729890240781827	-0.194287457127412	0.09771211466165844	0.5107767852913148	64
Women	0	CVLT_10	Model 1c APOE x age x education	APOE	-0.10335966202900237	0.5755404169328666	-1.256305660571602	1.0495863365135971	0.8581249345637247	64
Women	0	CVLT_10	Model 1c APOE x age x education	APOE_age	0.26482815791729386	0.17148548605083896	-0.07869855043116547	0.6083548662657532	0.12814405796817074	64
Women	0	CVLT_10	Model 1c APOE x age x education	APOE_age_education	-0.2848948256778393	0.2856220220678033	-0.8570644904837282	0.28727483912804963	0.32283482131608576	64
Women	0	CVLT_10	Model 1c APOE x age x education	APOE_education	-0.3851170788163786	0.8632779787937127	-2.1144706776206332	1.3442365199878763	0.6572378701275718	64
Women	0	CVLT_10	Model 1c APOE x age x education	age_c	-0.04071990423982544	0.0741256133524697	-0.18921135121706625	0.10777154273741538	0.5849585913879466	64
Women	0	CVLT_10	Model 1c APOE x age x education	age_education	0.1255455210387171	0.11500430820936415	-0.10483579200921173	0.35592683408664594	0.2796574253369104	64
Women	0	CVLT_10	Model 1c APOE x age x education	education_c	-0.15336989452831754	0.3851911925195133	-0.9250005759249763	0.6182607868683413	0.6920220625108078	64
Women	0	CVLT_10	Model 2a PICALM	PICALM	-1.1925708699902247	0.41540859872099045	-2.022960826764233	-0.36218091321621637	0.00559417631678052	64
Women	0	CVLT_10	Model 2b PICALM x age	PICALM	-1.168924348209421	0.4221332503567017	-2.0133165694977317	-0.3245321269211102	0.007468364588016752	64
Women	0	CVLT_10	Model 2b PICALM x age	PICALM_age	0.13595457187291274	0.12775931056032894	-0.11960209878295666	0.3915112425287821	0.29152649533816466	64
Women	0	CVLT_10	Model 2b PICALM x age	age_c	-0.06862618153567257	0.07724968563220616	-0.22314855945706083	0.08589619638571569	0.37789025063101733	64
Women	0	CVLT_10	Model 2c PICALM x age x education	PICALM	-1.2110337298606035	0.42536169820145636	-2.0628058382143855	-0.3592616215068215	0.006124160342195239	64
Women	0	CVLT_10	Model 2c PICALM x age x education	PICALM_age	0.12412521596296777	0.12930313884846042	-0.13479985335898337	0.3830502852849189	0.3411322487048041	64
Women	0	CVLT_10	Model 2c PICALM x age x education	PICALM_education	0.8738593943333551	0.6287898559817515	-0.385270573422669	2.132989362089379	0.1700098103528577	64
Women	0	CVLT_10	Model 2c PICALM x age x education	age_c	-0.0690277747678733	0.07648205423151955	-0.2221804466221128	0.0841248970863662	0.3705706843766199	64
Women	0	CVLT_10	Model 2c PICALM x age x education	age_education	0.1728967020192108	0.10255320629071502	-0.032462551317491206	0.37825595535591283	0.09727802641705666	64
Women	0	CVLT_10	Model 2c PICALM x age x education	education_c	-0.3636630099180381	0.395102891486397	-1.154842902985551	0.42751688314947467	0.36122928315076863	64

Sex	Cluster	Outcome	Model	Predictor	Beta	SE	CI_low	CI_high	p	N
Women	0	CVLT_10	Model 3a APOE x PICALM	APOE	-0.5555555555555545	0.65509288202303	-1.8659364206832658	0.7548253095721569	0.3997772347698524	64
Women	0	CVLT_10	Model 3a APOE x PICALM	APOE_PICALM	0.8863247863247865	1.0483203872525173	-1.2106282010695695	2.9832777737191423	0.401206843487664	64
Women	0	CVLT_10	Model 3a APOE x PICALM	PICALM	-1.3974358974358974	0.4744227357624659	-2.3464226624956046	-0.44844913237619	0.004582988100387214	64
Women	0	CVLT_10	Model 3b APOE x PICALM x age	APOE	-0.5523660210588798	0.6651804482020016	-1.8838693543363907	0.7791373122186313	0.4097177036267726	64
Women	0	CVLT_10	Model 3b APOE x PICALM x age	APOE_PICALM	0.5317152301819664	2.0825547373639814	-3.6369709992890047	4.700401459652937	0.7993801740341735	64
Women	0	CVLT_10	Model 3b APOE x PICALM x age	APOE_PICALM_age	0.23980199025910587	1.0189714973853112	-1.799891071902723	2.2794950524209345	0.8147759109612316	64
Women	0	CVLT_10	Model 3b APOE x PICALM x age	PICALM	-1.42728666900733	0.48751034293845347	-2.4031446461688724	-0.45142869184578793	0.00487163616503161	64
Women	0	CVLT_10	Model 3b APOE x PICALM x age	age_c	-0.025516275973389825	0.06418371910005008	-0.15399394869342656	0.10296139674664692	0.6924208571292323	64
Women	0	CVLT_10	Model 3c APOE x PICALM x age x education	APOE	-0.5208629376607204	0.6771584948830404	-1.8779188867910075	0.8361930114695665	0.4450718305870036	64
Women	0	CVLT_10	Model 3c APOE x PICALM x age x education	APOE_PICALM	0.5735177184999705	1.9427874915005974	-3.3199154188812074	4.466950855881148	0.7689499498947695	64
Women	0	CVLT_10	Model 3c APOE x PICALM x age x education	APOE_PICALM_age	0.26852189706099927	1.0129851105424195	-1.7615456292711154	2.298589423393114	0.7919389634088849	64
Women	0	CVLT_10	Model 3c APOE x PICALM x age x education	APOE_PICALM_education	0.15004824030522473	0.5082874251019	-0.8685825223817103	1.1686790029921599	0.7689499498947694	64
Women	0	CVLT_10	Model 3c APOE x PICALM x age x education	APOE_age_education	-0.31216150790757524	0.24466762582642004	-0.8024863870847531	0.17816337126960258	0.20736726555332957	64
Women	0	CVLT_10	Model 3c APOE x PICALM x age x education	PICALM	-1.553629685397958	0.4905847927656723	-2.53678358010099	-0.5704757906949258	0.0025138456602105466	64
Women	0	CVLT_10	Model 3c APOE x PICALM x age x education	age_c	-0.021371446123893433	0.06401504594799166	-0.1496604650079811	0.10691757276019423	0.7397620590680797	64
Women	0	CVLT_10	Model 3c APOE x PICALM x age x education	age_education	0.18654518115114826	0.10876954688633299	-0.03143386186713151	0.404524224169428	0.09196583433016321	64
Women	0	CVLT_10	Model 3c APOE x PICALM x age x education	education_c	-0.06434596522725677	0.33047601005444177	-0.7266346891790721	0.5979427587245586	0.8463398425948154	64
Women	0	CVLT_11	Model 1a APOE	APOE	0.34285714285714297	0.2420017464667474	-0.14089745540221815	0.8266117411165042	0.16155986820247695	64
Women	0	CVLT_11	Model 1b APOE x age	APOE	0.25366370425200924	0.24072481747476326	-0.22785762384754	0.7351850323515585	0.2962226008500283	64
Women	0	CVLT_11	Model 1b APOE x age	APOE_age	0.17516116035455287	0.07182012093597998	0.03149952886951138	0.3188227918395944	0.017708263842219952	64
Women	0	CVLT_11	Model 1b APOE x age	age_c	-0.0428082191780823	0.03209377278999529	-0.10700532299013041	0.0213888846339658	0.1872948652858301	64
Women	0	CVLT_11	Model 1c APOE x age x education	APOE	0.28800217299408293	0.2441418612165927	-0.2010727445703016	0.7770770905584674	0.2431263113826782	64
Women	0	CVLT_11	Model 1c APOE x age x education	APOE_age	0.2087431355204279	0.07274343296201104	0.06302052858214693	0.3544657424587089	0.005788276539816965	64

Sex	Cluster	Outcome	Model	Predictor	Beta	SE	CI_low	CI_high	p	N
Women	0	CVLT_11	Model 1c APOE x age x education	APOE_age_education	-0.2343428546592035	0.121159678834882	-0.4770548567837698	0.008369147465362825	0.058151803462681874	64
Women	0	CVLT_11	Model 1c APOE x age x education	APOE_education	-0.6418890101407545	0.3661989432700074	-1.3754736444982951	0.09169562421678612	0.08510210469346667	64
Women	0	CVLT_11	Model 1c APOE x age x education	age_c	-0.04073387456710722	0.031443778187005056	-0.10372333138573625	0.02225558225152182	0.20047948870107066	64
Women	0	CVLT_11	Model 1c APOE x age x education	age_education	0.01980245266955797	0.04878435124293955	-0.0779243461828753	0.11752925152199126	0.6863496518001714	64
Women	0	CVLT_11	Model 1c APOE x age x education	education_c	-0.09186385670312593	0.16339650856687307	-0.4191863959818612	0.23545868257560937	0.5762136282618435	64
Women	0	CVLT_11	Model 2a PICALM	PICALM	-0.41446725317693056	0.19646972120116366	-0.8072046298175456	-0.02172987653631553	0.03893927327763844	64
Women	0	CVLT_11	Model 2b PICALM x age	PICALM	-0.4161157362692763	0.20143200157067417	-0.8190397302950688	-0.01319174224348374	0.04317501773665629	64
Women	0	CVLT_11	Model 2b PICALM x age	PICALM_age	0.016266281464247856	0.06096372087181137	-0.10567931661752171	0.13821187954601744	0.7905234084543764	64
Women	0	CVLT_11	Model 2b PICALM x age	age_c	-0.014113686391298674	0.036861722653811324	-0.08784810993141118	0.05962073714881383	0.7031604476203257	64
Women	0	CVLT_11	Model 2c PICALM x age x education	PICALM	-0.3941011555387772	0.20486284980480693	-0.8043319361648245	0.01612962508727006	0.05938711515177273	64
Women	0	CVLT_11	Model 2c PICALM x age x education	PICALM_age	0.008720803408488183	0.06227502293978651	-0.1159827790049894	0.13342438582196578	0.88912447706887	64
Women	0	CVLT_11	Model 2c PICALM x age x education	PICALM_education	0.4504702386987732	0.30283799027849256	-0.155952376595014	1.0568928539925604	0.14239534603550463	64
Women	0	CVLT_11	Model 2c PICALM x age x education	age_c	-0.013092088591595389	0.03683531369901146	-0.0868535319560147	0.060669354772823925	0.723585237295683	64
Women	0	CVLT_11	Model 2c PICALM x age x education	age_education	0.02693067141380195	0.04939171106888391	-0.07197452397691938	0.12583586680452327	0.5877111190559862	64
Women	0	CVLT_11	Model 2c PICALM x age x education	education_c	-0.2925009443886838	0.19028959273546917	-0.6735492811040137	0.0885473923266461	0.1297930577168111	64
Women	0	CVLT_11	Model 3a APOE x PICALM	APOE	0.27777777777777823	0.3086347696214894	-0.33958367969395986	0.8951392352495163	0.3717072812769794	64
Women	0	CVLT_11	Model 3a APOE x PICALM	APOE_PICALM	0.006837606837606425	0.4938965604541813	-0.9811026073392266	0.9947778210144396	0.989000204669763	64
Women	0	CVLT_11	Model 3a APOE x PICALM	PICALM	-0.38461538461538447	0.22351540640018414	-0.831712765224305	0.062481995993536044	0.09044879556518436	64
Women	0	CVLT_11	Model 3b APOE x PICALM x age	APOE	0.27936912522501056	0.31291464895961735	-0.34699759864262425	0.9057358490926454	0.37565552562746063	64
Women	0	CVLT_11	Model 3b APOE x PICALM x age	APOE_PICALM	0.3949988622039359	0.9796768476026253	-1.5660374124545267	2.3560351368623986	0.688288733027792	64
Women	0	CVLT_11	Model 3b APOE x PICALM x age	APOE_PICALM_age	-0.2015549347078533	0.4793452803161041	-1.161068763259098	0.7579588938433914	0.6756889346045039	64
Women	0	CVLT_11	Model 3b APOE x PICALM x age	PICALM	-0.3995087645702523	0.2293349545031167	-0.8585725527247932	0.05955502358428855	0.08680388036718101	64
Women	0	CVLT_11	Model 3b APOE x PICALM x age	age_c	-0.012730779577859837	0.03019334976757427	-0.07316933571252573	0.04770777655680607	0.6748451261595446	64

Sex	Cluster	Outcome	Model	Predictor	Beta	SE	CI_low	CI_high	p	N
Women	0	CVLT_11	Model 3c APOE x PICALM x age x education	APOE	0.337885819116954	0.32060566267577034	-0.3046222866613832	0.9803939248952911	0.296537363623734	64
Women	0	CVLT_11	Model 3c APOE x PICALM x age x education	APOE_PICALM	0.36830463514716805	0.9198270063175227	-1.4750698783919356	2.2116791486862715	0.6904082907019775	64
Women	0	CVLT_11	Model 3c APOE x PICALM x age x education	APOE_PICALM_age	-0.1653039716028516	0.47960524028017293	-1.1264543514244694	0.7958464082187663	0.7316594437979281	64
Women	0	CVLT_11	Model 3c APOE x PICALM x age x education	APOE_PICALM_education	0.09635877082338688	0.2406524144435377	-0.3859194449281223	0.578636986574896	0.6904082907019777	64
Women	0	CVLT_11	Model 3c APOE x PICALM x age x education	APOE_age_education	-0.17694479983484235	0.11583968436656085	-0.4090927149875099	0.05520311531782515	0.13236836401054436	64
Women	0	CVLT_11	Model 3c APOE x PICALM x age x education	PICALM	-0.41518416640650924	0.23227097315003065	-0.8806655984573215	0.05029726564430298	0.0793670011289966	64
Women	0	CVLT_11	Model 3c APOE x PICALM x age x education	age_c	-0.01066140036492456	0.030308393651504898	-0.07140077855209682	0.050077977822247696	0.7263594622016525	64
Women	0	CVLT_11	Model 3c APOE x PICALM x age x education	age_education	0.030475935864057488	0.05149774081245073	-0.07272784296231094	0.13367971469042592	0.5564149412503563	64
Women	0	CVLT_11	Model 3c APOE x PICALM x age x education	education_c	-0.1406377165087036	0.15646629408414803	-0.45420317692862583	0.17292774391121862	0.3726577576156256	64
Women	0	CVLT_12	Model 1a APOE	APOE	-0.5799999999999998	0.24341654196710993	-1.0665827341670127	-0.09341726583298704	0.020263214481520173	64
Women	0	CVLT_12	Model 1b APOE x age	APOE	-0.3963065700953825	0.22914256943124073	-0.8546599526594447	0.06204681246867966	0.0888580902147961	64
Women	0	CVLT_12	Model 1b APOE x age	APOE_age	-0.22921031426269411	0.0683645634080009	-0.3659598015506741	-0.09246082697471414	0.0013898684949831574	64
Women	0	CVLT_12	Model 1b APOE x age	age_c	0.002739726027397249	0.030549611116074203	-0.0583685945514686	0.0638480466062631	0.9288392483799766	64
Women	0	CVLT_12	Model 1c APOE x age x education	APOE	-0.3650287574285098	0.2341838021237587	-0.8341552855374359	0.10409777068041631	0.124694889854751	64
Women	0	CVLT_12	Model 1c APOE x age x education	APOE_age	-0.24694681316679157	0.06977637356285019	-0.3867256859014332	-0.10716794043214992	0.0008157965225075682	64
Women	0	CVLT_12	Model 1c APOE x age x education	APOE_age_education	-0.028662421923085102	0.11621781742899973	-0.26147468605248525	0.20414984220631505	0.806098950240526	64
Women	0	CVLT_12	Model 1c APOE x age x education	APOE_education	-0.009666654397958219	0.35126241948566156	-0.7133298361126575	0.6939965273167411	0.9781430247872058	64
Women	0	CVLT_12	Model 1c APOE x age x education	age_c	-1.743819005217695e-05	0.03016124925186937	-0.06043768082271681	0.06040280444261246	0.9995407455151386	64
Women	0	CVLT_12	Model 1c APOE x age x education	age_education	0.036992794474104085	0.04679453495309082	-0.056747923363477434	0.13073351231168562	0.4325495989711907	64
Women	0	CVLT_12	Model 1c APOE x age x education	education_c	0.33802441025086427	0.15673189120152836	0.02405270385392816	0.6519961166478003	0.03533684156141708	64
Women	0	CVLT_12	Model 2a PICALM	PICALM	0.059628543499516296	0.21023546533643878	-0.36062616357828303	0.4798832505773156	0.777640897871892	64
Women	0	CVLT_12	Model 2b PICALM x age	PICALM	0.029482171602187135	0.21025574178765105	-0.3910919307616439	0.45005627396601816	0.8889554187266221	64
Women	0	CVLT_12	Model 2b PICALM x age	PICALM_age	0.03432122025417178	0.06363424011125018	-0.09296621164589441	0.16160865215423798	0.591640306069275	64

Sex	Cluster	Outcome	Model	Predictor	Beta	SE	CI_low	CI_high	p	N
Women	0	CVLT_12	Model 2b PICALM x age	age_c	-0.06493590573611224	0.03847645249867842	-0.14190026986805382	0.012028458395829333	0.09666517146047364	64
Women	0	CVLT_12	Model 2c PICALM x age x education	PICALM	-0.02859842795047629	0.2135265810650375	-0.456178031173715	0.39898117527276244	0.8939266926811658	64
Women	0	CVLT_12	Model 2c PICALM x age x education	PICALM_age	0.024837136897815304	0.06490865838656992	-0.1051402095302105	0.1548144833258411	0.7034043170974494	64
Women	0	CVLT_12	Model 2c PICALM x age x education	PICALM_education	-0.1067198150774864	0.3156451291309543	-0.738788283555672	0.5253486534006993	0.7365295071557256	64
Women	0	CVLT_12	Model 2c PICALM x age x education	age_c	-0.06662688240658138	0.038393093741018076	-0.14350772649829183	0.01025396168512907	0.08807619807368187	64
Women	0	CVLT_12	Model 2c PICALM x age x education	age_education	0.016345363257260603	0.05148050614125299	-0.08674257211741707	0.11943329863193827	0.7520199680610682	64
Women	0	CVLT_12	Model 2c PICALM x age x education	education_c	0.32523122951785277	0.19833701516784102	-0.0719317926546254	0.722394251690331	0.10655670213004723	64
Women	0	CVLT_12	Model 3a APOE x PICALM	APOE	-0.5833333333333226	0.3198575923629337	-1.223143778691624	0.05647711202497874	0.07317471940892889	64
Women	0	CVLT_12	Model 3a APOE x PICALM	APOE_PICALM	0.006410256410258608	0.5118560196472727	-1.017454224875069	1.0302747376955863	0.9900494604571414	64
Women	0	CVLT_12	Model 3a APOE x PICALM	PICALM	-0.006410256410252169	0.23164305121832163	-0.4697653472469986	0.45694483442649425	0.9780148031300548	64
Women	0	CVLT_12	Model 3b APOE x PICALM x age	APOE	-0.5782663280303542	0.30715031597757103	-1.1930944857833918	0.036561829722683514	0.064762319159121	64
Women	0	CVLT_12	Model 3b APOE x PICALM x age	APOE_PICALM	1.9269367150417125	0.9616298063945554	0.0020255183065329696	3.851847911776892	0.049767257254755906	64
Women	0	CVLT_12	Model 3b APOE x PICALM x age	APOE_PICALM_age	-1.0308925290047908	0.47051505834247287	-1.9727307478425344	-0.08905431016704723	0.032484011014745226	64
Women	0	CVLT_12	Model 3b APOE x PICALM x age	PICALM	-0.05383222911755259	0.22511027839231884	-0.5044394092362587	0.3967749510011535	0.8118422569926662	64
Women	0	CVLT_12	Model 3b APOE x PICALM x age	age_c	-0.04053604242377268	0.02963714531219849	-0.09986123437535341	0.018789149527808045	0.17666852165575225	64
Women	0	CVLT_12	Model 3c APOE x PICALM x age x education	APOE	-0.5447193093554858	0.3128942143090614	-1.1717733272629163	0.08233470855194469	0.08729196848512753	64
Women	0	CVLT_12	Model 3c APOE x PICALM x age x education	APOE_PICALM	1.7061469158665243	0.8977026358172567	-0.09288936838796502	3.5051832001210137	0.06260431817429579	64
Women	0	CVLT_12	Model 3c APOE x PICALM x age x education	APOE_PICALM_age	-1.0367428456926384	0.4680694145684361	-1.9747749141757174	-0.09871077720955945	0.030925231436355338	64
Women	0	CVLT_12	Model 3c APOE x PICALM x age x education	APOE_PICALM_education	0.44637564659298645	0.23486406169637508	-0.024302451031734174	0.9170537442177071	0.06260431817429526	64
Women	0	CVLT_12	Model 3c APOE x PICALM x age x education	APOE_age_education	0.01052803486046583	0.11305342121277501	-0.2160360841539864	0.23709215387491805	0.9261431143216179	64
Women	0	CVLT_12	Model 3c APOE x PICALM x age x education	PICALM	-0.0897046477714305	0.22668421712837206	-0.5439899705615285	0.3645806750186676	0.6938386902989855	64
Women	0	CVLT_12	Model 3c APOE x PICALM x age x education	age_c	-0.04562256422738942	0.029579393387533168	-0.10490099323853272	0.013655864783753885	0.12871665596873283	64
Women	0	CVLT_12	Model 3c APOE x PICALM x age x education	age_education	0.03127499226245167	0.05025907844459672	-0.06944645170736234	0.1319964362322657	0.5363321185965406	64

Sex	Cluster	Outcome	Model	Predictor	Beta	SE	CI_low	CI_high	p	N
Women	0	CVLT_12	Model 3c APOE x PICALM x age x education	education_c	0.29836613844637494	0.15270284917837162	-0.007657209842929946	0.6043894867356798	0.055805887433496404	64
Women	0	CVLT_13	Model 1a APOE	APOE	0.3028571428571428	0.28332270952987904	-0.2634968836218068	0.8692111693360924	0.2892374852810279	64
Women	0	CVLT_13	Model 1b APOE x age	APOE	0.2900879042282727	0.2946121610286389	-0.2992241598162282	0.8793999682727736	0.3287533194692668	64
Women	0	CVLT_13	Model 1b APOE x age	APOE_age	-0.023381681439699167	0.08789738115184363	-0.19920262151848928	0.1524392586390909	0.791142614033204	64
Women	0	CVLT_13	Model 1b APOE x age	age_c	0.025342465753424595	0.03927810957095811	-0.05322545127420038	0.10391038278104957	0.5212534550608223	64
Women	0	CVLT_13	Model 1c APOE x age x education	APOE	0.263254354848689	0.26887865132111155	-0.27537430790666084	0.8018830176040388	0.3317502001232383	64
Women	0	CVLT_13	Model 1c APOE x age x education	APOE_age	0.06259870386489513	0.0801138979191329	-0.09788871859233367	0.22308612632212393	0.43787512751692426	64
Women	0	CVLT_13	Model 1c APOE x age x education	APOE_age_education	-0.372284561904612	0.13343574460063973	-0.6395884788383985	-0.10498064497082543	0.007191895886172599	64
Women	0	CVLT_13	Model 1c APOE x age x education	APOE_education	-1.749970483527808	0.40330272527210487	-2.557882924815205	-0.9420580422404109	6.039795010862692e-05	64
Women	0	CVLT_13	Model 1c APOE x age x education	age_c	0.025038338059782686	0.03462970516089236	-0.044333297400213176	0.09440997351977855	0.472670203841307	64
Women	0	CVLT_13	Model 1c APOE x age x education	age_education	0.030720253712550417	0.05372724899537014	-0.07690835918665345	0.1383488666117543	0.5697580294242233	64
Women	0	CVLT_13	Model 1c APOE x age x education	education_c	0.12813640576920507	0.1799520681750803	-0.23235090464000405	0.48862371617841416	0.4793846682364501	64
Women	0	CVLT_13	Model 2a PICALM	PICALM	-0.037145650048875656	0.2364676508844966	-0.5098377488667771	0.4355464487690258	0.8756878677110549	64
Women	0	CVLT_13	Model 2b PICALM x age	PICALM	-0.022193956113272156	0.24159638450103926	-0.5054586778372208	0.4610707656106764	0.9271123000237476	64
Women	0	CVLT_13	Model 2b PICALM x age	PICALM_age	-0.015658970123224106	0.07311953628774578	-0.16191981930629462	0.13060187905984644	0.8311517303651632	64
Women	0	CVLT_13	Model 2b PICALM x age	age_c	0.031205490094522875	0.04421173820544221	-0.05723115354528782	0.11964213373433358	0.4830322090332758	64
Women	0	CVLT_13	Model 2c PICALM x age x education	PICALM	0.029260082076068034	0.24126708309280404	-0.4538689182811654	0.5123890824333014	0.9038983801372398	64
Women	0	CVLT_13	Model 2c PICALM x age x education	PICALM_age	-0.03088376876010366	0.07334132639732127	-0.17774724160932726	0.11597970408911995	0.6752683216460418	64
Women	0	CVLT_13	Model 2c PICALM x age x education	PICALM_education	0.711077461079541	0.3566524561861506	-0.0031067634045248704	1.4252616855636067	0.05097155633966479	64
Women	0	CVLT_13	Model 2c PICALM x age x education	age_c	0.03329524892308333	0.04338096780083146	-0.05357364068869702	0.12016413853486368	0.44594716882309915	64
Women	0	CVLT_13	Model 2c PICALM x age x education	age_education	0.00762174105151586	0.0581686434114643	-0.1088589581937568	0.12410244029678852	0.896214255557952	64
Women	0	CVLT_13	Model 2c PICALM x age x education	education_c	-0.4745081200147689	0.22410415078159782	-0.9232689412386622	-0.02574729879087556	0.038604938678428485	64
Women	0	CVLT_13	Model 3a APOE x PICALM	APOE	0.3333333333333335	0.37224215407789923	-0.4112618367305849	1.0779285033972519	0.37411204524765573	64

Sex	Cluster	Outcome	Model	Predictor	Beta	SE	CI_low	CI_high	p	N
Women	0	CVLT_13	Model 3a APOE x PICALM	APOE_PICALM	-0.07948717948717948	0.5956850544758873	-1.2710346965617425	1.1120603375873837	0.8942933378893533	64
Women	0	CVLT_13	Model 3a APOE x PICALM	PICALM	0.012820512820512941	0.26958030830434504	-0.5264203907386011	0.5520614163796271	0.9622270061115851	64
Women	0	CVLT_13	Model 3b APOE x PICALM x age	APOE	0.3304903386819019	0.37716665149367107	-0.4244907420494922	1.0854714194132962	0.38451168222136023	64
Women	0	CVLT_13	Model 3b APOE x PICALM x age	APOE_PICALM	0.15188990302676264	1.180837769611237	-2.211813706343114	2.5155935123966398	0.8980962678591015	64
Women	0	CVLT_13	Model 3b APOE x PICALM x age	APOE_PICALM_age	-0.1656011000685917	0.5777711426653345	-1.322135698176363	0.9909334980391795	0.7754242457175762	64
Women	0	CVLT_13	Model 3b APOE x PICALM x age	PICALM	0.03942802686595755	0.27642520779382473	-0.5138971446334213	0.5927531983653365	0.8870724732898301	64
Women	0	CVLT_13	Model 3b APOE x PICALM x age	age_c	0.02274395721144863	0.03639306969832163	-0.05010468670539796	0.09559260112829522	0.5344529811303542	64
Women	0	CVLT_13	Model 3c APOE x PICALM x age x education	APOE	0.36695578466421913	0.3901038453240689	-0.414829791498517	1.1487413608269552	0.3509914358390527	64
Women	0	CVLT_13	Model 3c APOE x PICALM x age x education	APOE_PICALM	0.16560614541326685	1.1192193213389257	-2.0773594968724254	2.4085717876989587	0.8829109188067447	64
Women	0	CVLT_13	Model 3c APOE x PICALM x age x education	APOE_PICALM_age	-0.1346357190413247	0.5835700059361715	-1.304136145121725	1.0348647070390755	0.818395778553602	64
Women	0	CVLT_13	Model 3c APOE x PICALM x age x education	APOE_PICALM_education	0.04332718920695926	0.29281900848983494	-0.5434952172049946	0.630149595618913	0.8829109188067448	64
Women	0	CVLT_13	Model 3c APOE x PICALM x age x education	APOE_age_education	-0.12055466053977561	0.14095043092929438	-0.4030256363459851	0.1619163152664339	0.396096678108493	64
Women	0	CVLT_13	Model 3c APOE x PICALM x age x education	PICALM	0.05590214989849042	0.2826207092749467	-0.5104824081734449	0.6222867079704257	0.8439314217223797	64
Women	0	CVLT_13	Model 3c APOE x PICALM x age x education	age_c	0.02588545941899175	0.03687839076318726	-0.048020487206072306	0.09979140604405581	0.48569262461489937	64
Women	0	CVLT_13	Model 3c APOE x PICALM x age x education	age_education	-0.009809426491054235	0.06266098530129775	-0.13538484719987784	0.11576599421776937	0.8761746383274995	64
Women	0	CVLT_13	Model 3c APOE x PICALM x age x education	education_c	-0.2267271776691094	0.19038373332650965	-0.608264705265213	0.1548103499269942	0.23880886931103118	64
Women	0	CVLT_2	Model 1a APOE	APOE	-0.3942857142857147	0.6513929976542745	-1.6964017629916	0.9078303344201705	0.5471914945621946	64
Women	0	CVLT_2	Model 1b APOE x age	APOE	-0.25592071441510594	0.6750542811419085	-1.6062303227246673	1.0943888938944555	0.7059435678337898	64
Women	0	CVLT_2	Model 1b APOE x age	APOE_age	-0.14468842331453297	0.201402084830932	-0.5475525749509775	0.25817572832191155	0.47529545258186257	64
Women	0	CVLT_2	Model 1b APOE x age	age_c	-0.01609589041095902	0.08999919055771348	-0.19612107526659964	0.1639292944446816	0.858662082813781	64
Women	0	CVLT_2	Model 1c APOE x age x education	APOE	-0.25677525183621625	0.7105450685011989	-1.6801680656343683	1.1666175619619357	0.71917689528777	64
Women	0	CVLT_2	Model 1c APOE x age x education	APOE_age	-0.20163634603485248	0.21171087702632638	-0.6257441955169839	0.22247150344727895	0.34498011196697576	64
Women	0	CVLT_2	Model 1c APOE x age x education	APOE_age_education	0.1856622609874941	0.35262044726094505	-0.5207213772639796	0.8920458992389677	0.6006052444245722	64

Sex	Cluster	Outcome	Model	Predictor	Beta	SE	CI_low	CI_high	p	N
Women	0	CVLT_2	Model 1c APOE x age x education	APOE_education	0.7014263989268392	1.0657772982242257	-1.4335820820996013	2.8364348799532797	0.513147364486314	64
Women	0	CVLT_2	Model 1c APOE x age x education	age_c	-0.019024280734238785	0.09151327598834394	-0.2023474015092526	0.16429884004077502	0.8360725874654431	64
Women	0	CVLT_2	Model 1c APOE x age x education	age_education	0.06308786590318305	0.1419808959551963	-0.2213340461727693	0.3475097779791354	0.6585088829381947	64
Women	0	CVLT_2	Model 1c APOE x age x education	education_c	0.4401749215033852	0.47554558154820425	-0.5124573511221687	1.392807194128939	0.3586148889672479	64
Women	0	CVLT_2	Model 2a PICALM	PICALM	-0.11925708699901855	0.5402106015387461	-1.1991226926744398	0.9606085186764026	0.8260044131626794	64
Women	0	CVLT_2	Model 2b PICALM x age	PICALM	-0.22372117713486334	0.5438814136198014	-1.3116459842325887	0.8642036299628622	0.6822875213991915	64
Women	0	CVLT_2	Model 2b PICALM x age	PICALM_age	-0.22773538865742435	0.16460658896670063	-0.5569975900567121	0.10152681274186345	0.17163361899466734	64
Women	0	CVLT_2	Model 2b PICALM x age	age_c	0.029457464715784393	0.09952939785678395	-0.1696309730435313	0.2285459024751001	0.7682768772086404	64
Women	0	CVLT_2	Model 2c PICALM x age x education	PICALM	-0.3719595477653186	0.5542651199225591	-1.481856305700031	0.7379372101693937	0.5048762540678104	64
Women	0	CVLT_2	Model 2c PICALM x age x education	PICALM_age	-0.23122936703123492	0.16848771307627775	-0.5686201927814148	0.106161458718945	0.17532130378265948	64
Women	0	CVLT_2	Model 2c PICALM x age x education	PICALM_education	-0.6249478990240662	0.8193410135548987	-2.265649978048236	1.0157541800001033	0.4487609009408142	64
Women	0	CVLT_2	Model 2c PICALM x age x education	age_c	0.024782993136653664	0.09965950187757576	-0.17478171706333906	0.2243477033366464	0.8045047217806842	64
Women	0	CVLT_2	Model 2c PICALM x age x education	age_education	0.05809886680271931	0.1336313669601846	-0.20949332981289182	0.32569106341833043	0.6653712437130306	64
Women	0	CVLT_2	Model 2c PICALM x age x education	education_c	0.8653676005734796	0.5148365554712936	-0.1655748189381443	1.8963100200851035	0.09826287374060659	64
Women	0	CVLT_2	Model 3a APOE x PICALM	APOE	-0.05555555555554981	0.851967700842889	-1.7597446919780773	1.6486335808669776	0.9482247582366174	64
Women	0	CVLT_2	Model 3a APOE x PICALM	APOE_PICALM	-0.9367521367521363	1.3633717211460343	-3.6639016211563815	1.7903973476521091	0.494676460124983	64
Women	0	CVLT_2	Model 3a APOE x PICALM	PICALM	0.02564102564102877	0.6170008230999708	-1.2085443769868487	1.259826428268906	0.9669894195771923	64
Women	0	CVLT_2	Model 3b APOE x PICALM x age	APOE	-0.05051248886030968	0.864832737075373	-1.7816632995252635	1.6806383218046441	0.9536250214797973	64
Women	0	CVLT_2	Model 3b APOE x PICALM x age	APOE_PICALM	-1.027024905850527	2.707628461558196	-6.446932137920833	4.392882326219779	0.7058444139528881	64
Women	0	CVLT_2	Model 3b APOE x PICALM x age	APOE_PICALM_age	0.11177310499050597	1.3248133066261074	-2.5401288541112366	2.7636750640922485	0.9330536364648159	64
Women	0	CVLT_2	Model 3b APOE x PICALM x age	PICALM	-0.021556906250330687	0.6338353831289713	-1.2903162747294865	1.2472024622288251	0.9729857454514417	64
Women	0	CVLT_2	Model 3b APOE x PICALM x age	age_c	-0.04034453356192824	0.08344830581688491	-0.20738446633788532	0.12669539921402884	0.6305841537647916	64
Women	0	CVLT_2	Model 3c APOE x PICALM x age x education	APOE	-0.04314635238625786	0.8941268472953935	-1.835016596307362	1.7487238915348462	0.96168758994874	64

Sex	Cluster	Outcome	Model	Predictor	Beta	SE	CI_low	CI_high	p	N
Women	0	CVLT_2	Model 3c APOE x PICALM x age x education	APOE_PICALM	-1.0973100909979765	2.5652760289751497	-6.238238134562321	4.043617952566368	0.6704996438594581	64
Women	0	CVLT_2	Model 3c APOE x PICALM x age x education	APOE_PICALM_age	0.0748936605335131	1.3375556684154264	-2.6056277991132486	2.755415120180275	0.9555503557821725	64
Women	0	CVLT_2	Model 3c APOE x PICALM x age x education	APOE_PICALM_education	-0.2870869424122605	0.6711477982783816	-1.6320971863680471	1.0579233015435259	0.6704996438594587	64
Women	0	CVLT_2	Model 3c APOE x PICALM x age x education	APOE_age_education	0.09291809089487102	0.32306157948031217	-0.5545117821438004	0.7403479639335424	0.7747206894018897	64
Women	0	CVLT_2	Model 3c APOE x PICALM x age x education	PICALM	-0.10750773314969031	0.6477730655396974	-1.4056739658997388	1.1906584996003582	0.8687929905135774	64
Women	0	CVLT_2	Model 3c APOE x PICALM x age x education	age_c	-0.04955929410527754	0.08452610673197518	-0.21895339735323396	0.11983480914267888	0.5600596424620035	64
Women	0	CVLT_2	Model 3c APOE x PICALM x age x education	age_education	0.08326420377309326	0.1436203972542989	-0.20455750411829923	0.3710859116644858	0.5644493512024923	64
Women	0	CVLT_2	Model 3c APOE x PICALM x age x education	education_c	0.6192012795259241	0.43636382798059076	-0.255291373554661	1.4936939326065093	0.16154083318040505	64
Women	0	CVLT_3	Model 1a APOE	APOE	0.17142857142857237	0.5422990097091585	-0.912611702695443	1.2554688455525878	0.7529773931923125	64
Women	0	CVLT_3	Model 1b APOE x age	APOE	0.432594486504383	0.5411044514176393	-0.6497755691485418	1.5149645421573077	0.4271741084892957	64
Women	0	CVLT_3	Model 1b APOE x age	APOE_age	-0.14850255170561605	0.1614382245564944	-0.4714270806758208	0.1744219772645887	0.36132308306774574	64
Women	0	CVLT_3	Model 1b APOE x age	age_c	-0.11130136986301392	0.07214080999884179	-0.2556044749820417	0.03300173525601385	0.1281289997890329	64
Women	0	CVLT_3	Model 1c APOE x age x education	APOE	0.46581959736198075	0.5760230086656948	-0.6880931485903996	1.6197323433143611	0.42212031910702585	64
Women	0	CVLT_3	Model 1c APOE x age x education	APOE_age	-0.16918108570618579	0.17162927695662655	-0.5129958418521188	0.17463367043974726	0.32850283425348215	64
Women	0	CVLT_3	Model 1c APOE x age x education	APOE_age_education	0.12807250447347474	0.28586151667584164	-0.4445769256831812	0.7007219346301307	0.6558652239942165	64
Women	0	CVLT_3	Model 1c APOE x age x education	APOE_education	0.5717010514659346	0.8640018390187141	-1.1591026136157974	2.3025047165476664	0.5108837993906525	64
Women	0	CVLT_3	Model 1c APOE x age x education	age_c	-0.11327990869173798	0.07418776782006618	-0.26189586602932635	0.03533604864585041	0.13240593707453102	64
Women	0	CVLT_3	Model 1c APOE x age x education	age_education	-0.10167924490591018	0.11510073954024658	-0.3322537331224354	0.12889524331061503	0.38080048279207823	64
Women	0	CVLT_3	Model 1c APOE x age x education	education_c	-0.19472598341712244	0.38551417606610544	-0.9670036786058325	0.5775517117715876	0.6154657135553654	64
Women	0	CVLT_3	Model 2a PICALM	PICALM	-0.09775171065493149	0.4487801260117058	-0.9948503999629819	0.7993469786531189	0.8282873899253166	64
Women	0	CVLT_3	Model 2b PICALM x age	PICALM	-0.219659178457011	0.44342587359467656	-1.1066429876332131	0.6673246307191911	0.6221495193764826	64
Women	0	CVLT_3	Model 2b PICALM x age	PICALM_age	-0.06211961939881805	0.13420355740088424	-0.3305667029743725	0.20632746417673642	0.6451276501562744	64
Women	0	CVLT_3	Model 2b PICALM x age	age_c	-0.1151107082739862	0.08114620041759366	-0.2774272762340314	0.04720585968605903	0.16120111980686097	64

Sex	Cluster	Outcome	Model	Predictor	Beta	SE	CI_low	CI_high	p	N
Women	0	CVLT_3	Model 2c PICALM x age x education	PICALM	-0.1736850533759463	0.4610974586993528	-1.097016787788262	0.7496466810363693	0.7078118803459372	64
Women	0	CVLT_3	Model 2c PICALM x age x education	PICALM_age	-0.06994861133109925	0.14016623729162678	-0.35062666006636894	0.21072943740417044	0.6196715692053951	64
Women	0	CVLT_3	Model 2c PICALM x age x education	PICALM_education	0.10438989183079994	0.6816161536758812	-1.260522912399943	1.469302696061543	0.8788204403761639	64
Women	0	CVLT_3	Model 2c PICALM x age x education	age_c	-0.1137121279074218	0.08290751374976292	-0.2797315605070163	0.05230730469217268	0.1755774437832932	64
Women	0	CVLT_3	Model 2c PICALM x age x education	age_education	-0.07095372765469715	0.11116897219956905	-0.29356575462921636	0.15165829931982205	0.525866277862333	64
Women	0	CVLT_3	Model 2c PICALM x age x education	education_c	-0.11916576653429489	0.42829652965806236	-0.9768147735087711	0.7384832404401813	0.7818411560682993	64
Women	0	CVLT_3	Model 3a APOE x PICALM	APOE	0.555555555555565	0.7076829313828945	-0.860021070766306	1.971132181877436	0.4355221690959924	64
Women	0	CVLT_3	Model 3a APOE x PICALM	APOE_PICALM	-1.0170940170940161	1.1324782561951754	-3.2823878064397327	1.2481997722517004	0.37271499604143765	64
Women	0	CVLT_3	Model 3a APOE x PICALM	PICALM	0.1282051282051324	0.5125088084032506	-0.8969651250070134	1.1533753814172782	0.8033243172191169	64
Women	0	CVLT_3	Model 3b APOE x PICALM x age	APOE	0.5731242137624002	0.6912417931355005	-0.8105465693288352	1.9567949968536356	0.4104343192929406	64
Women	0	CVLT_3	Model 3b APOE x PICALM x age	APOE_PICALM	-2.150443640651903	2.164147901294219	-6.482456332948759	2.181569051644953	0.32451228733813475	64
Women	0	CVLT_3	Model 3b APOE x PICALM x age	APOE_PICALM_age	0.8548349799404309	1.058894149565698	-1.264771953104327	2.974441912985189	0.42279761630746215	64
Women	0	CVLT_3	Model 3b APOE x PICALM x age	PICALM	-0.03621949347425768	0.5066106866727135	-1.0503109626418519	0.9778719756933365	0.9432507651576649	64
Women	0	CVLT_3	Model 3b APOE x PICALM x age	age_c	-0.1405492656547096	0.06669839620323702	-0.2740606114991754	-0.007037919810243848	0.039431664029238166	64
Women	0	CVLT_3	Model 3c APOE x PICALM x age x education	APOE	0.6028878911593453	0.7239430765378437	-0.8479264547746154	2.053702237093306	0.4085685641595813	64
Women	0	CVLT_3	Model 3c APOE x PICALM x age x education	APOE_PICALM	-2.0627883302522187	2.077013822146777	-6.225217045344917	2.0996403848404794	0.32498578240085607	64
Women	0	CVLT_3	Model 3c APOE x PICALM x age x education	APOE_PICALM_age	0.8672063054949408	1.0829718049092345	-1.3031176905826412	3.037530301572523	0.4267131653541626	64
Women	0	CVLT_3	Model 3c APOE x PICALM x age x education	APOE_PICALM_education	-0.5396829933799407	0.5434047790500284	-1.6286905060495411	0.5493245192896596	0.32498578240085585	64
Women	0	CVLT_3	Model 3c APOE x PICALM x age x education	APOE_age_education	0.0484490167476499	0.2615716041494566	-0.47575219200464214	0.5726502254999419	0.8537356721702529	64
Women	0	CVLT_3	Model 3c APOE x PICALM x age x education	PICALM	0.030294862007914425	0.5244790796559441	-1.0207847015208729	1.0813744255367017	0.9541477043679267	64
Women	0	CVLT_3	Model 3c APOE x PICALM x age x education	age_c	-0.1414726465434526	0.06843781722963539	-0.2786250971421994	-0.004320195944705757	0.043437031238672555	64
Women	0	CVLT_3	Model 3c APOE x PICALM x age x education	age_education	-0.09220560524792387	0.11628438689249511	-0.3252447241778054	0.14083351368195768	0.43122478471534653	64
Women	0	CVLT_3	Model 3c APOE x PICALM x age x education	education_c	-0.07540626470947132	0.3533084517858508	-0.7834522244028703	0.6326396949839277	0.831781564241524	64

Sex	Cluster	Outcome	Model	Predictor	Beta	SE	CI_low	CI_high	p	N
Women	0	CVLT_4	Model 1a APOE	APOE	0.4514285714285714	0.6271387107134375	-0.802203848516626	1.7050609913737689	0.4743391322267876	64
Women	0	CVLT_4	Model 1b APOE x age	APOE	0.6109849286959128	0.6477785467898299	-0.6847650875953464	1.906734944987172	0.34936099019128775	64
Women	0	CVLT_4	Model 1b APOE x age	APOE_age	-0.1832997582594694	0.193264384030767	-0.5698860847091402	0.20328656819020138	0.34670981697779557	64
Women	0	CVLT_4	Model 1b APOE x age	age_c	-0.007876712328767438	0.08636275111553735	-0.1806279352883364	0.16487451063080152	0.9276335390179277	64
Women	0	CVLT_4	Model 1c APOE x age x education	APOE	0.5573134443990848	0.6641080148339268	-0.7730547726294639	1.8876816614276335	0.4049303690889564	64
Women	0	CVLT_4	Model 1c APOE x age x education	APOE_age	-0.2636177347794688	0.19787469717758793	-0.660008385395305	0.1327729158363674	0.18817578963986503	64
Women	0	CVLT_4	Model 1c APOE x age x education	APOE_age_education	0.3231892510393667	0.3295752452610576	-0.33702930019185795	0.9834078022705913	0.330996080672586	64
Women	0	CVLT_4	Model 1c APOE x age x education	APOE_education	1.4803912373993977	0.9961243517905892	-0.5150856251434526	3.475868099942248	0.14284774665510896	64
Women	0	CVLT_4	Model 1c APOE x age x education	age_c	-0.0016370321784359987	0.08553250559568946	-0.1729792301728046	0.1697051658159326	0.9847980120395792	64
Women	0	CVLT_4	Model 1c APOE x age x education	age_education	0.16318269089546722	0.13270185824530653	-0.102651075008309	0.42901645679924344	0.22395310638783905	64
Women	0	CVLT_4	Model 1c APOE x age x education	education_c	0.07703874255777654	0.44446671453394276	-0.8133350781691507	0.9674125632847038	0.8630182358255301	64
Women	0	CVLT_4	Model 2a PICALM	PICALM	0.670576735092868	0.5139246725316582	-0.3567440471986232	1.6978975173843591	0.19677817053865357	64
Women	0	CVLT_4	Model 2b PICALM x age	PICALM	0.6252541778881588	0.5255316121396555	-0.42596556127443685	1.6764739170507545	0.23882915782054523	64
Women	0	CVLT_4	Model 2b PICALM x age	PICALM_age	-0.09550576290769591	0.1590529918879551	-0.41365911616602424	0.22264759035063242	0.5504578056149614	64
Women	0	CVLT_4	Model 2b PICALM x age	age_c	0.010358668911045255	0.09617141457885724	-0.18201280221107333	0.20273014003316384	0.9145847316477222	64
Women	0	CVLT_4	Model 2c PICALM x age x education	PICALM	0.4933388619964767	0.5350065181829204	-0.5779932111603676	1.564670935153321	0.36035404559668194	64
Women	0	CVLT_4	Model 2c PICALM x age x education	PICALM_age	-0.06874087837285364	0.16263340681105234	-0.39440865803880876	0.2569269012931015	0.674123580733816	64
Women	0	CVLT_4	Model 2c PICALM x age x education	PICALM_education	-0.5756578226152531	0.7908720341769244	-2.1593517537737608	1.0080361085432548	0.46966823833041105	64
Women	0	CVLT_4	Model 2c PICALM x age x education	age_c	0.006098313239150999	0.09619671378710531	-0.1865322834168304	0.1987289098951324	0.9496744022386239	64
Women	0	CVLT_4	Model 2c PICALM x age x education	age_education	0.16837908943156124	0.12898818595581382	-0.08991529760156128	0.4266734764646838	0.19700691695834704	64
Women	0	CVLT_4	Model 2c PICALM x age x education	education_c	0.4875929729585413	0.49694794616422694	-0.50752812430093	1.4827140702180126	0.3306527458294951	64
Women	0	CVLT_4	Model 3a APOE x PICALM	APOE	0.22222222222222787	0.807703566074175	-1.3934254618290953	1.837869906273551	0.7841627972718556	64
Women	0	CVLT_4	Model 3a APOE x PICALM	APOE_PICALM	0.8931623931623949	1.292537498739532	-1.6922975504380502	3.47862233676284	0.49222122486561504	64

Sex	Cluster	Outcome	Model	Predictor	Beta	SE	CI_low	CI_high	p	N
Women	0	CVLT_4	Model 3a APOE x PICALM	PICALM	0.5512820512820539	0.584944434625285	-0.6187810272982665	1.721345129862374	0.3497406643606955	64
Women	0	CVLT_4	Model 3b APOE x PICALM x age	APOE	0.22741804003346167	0.8173225693326499	-1.4086308371862102	1.8634669172531337	0.7818118664028488	64
Women	0	CVLT_4	Model 3b APOE x PICALM x age	APOE_PICALM	2.186270584553744	2.5588830719830673	-2.935890400518033	7.308431569625521	0.39640714000973165	64
Women	0	CVLT_4	Model 3b APOE x PICALM x age	APOE_PICALM_age	-0.672719171795821	1.2520337971010045	-3.17893711419365	1.833498770602008	0.5931151071362111	64
Women	0	CVLT_4	Model 3b APOE x PICALM x age	PICALM	0.5026545256127919	0.5990152102992902	-0.6964046941122324	1.7017137453378162	0.4048390365612403	64
Women	0	CVLT_4	Model 3b APOE x PICALM x age	age_c	-0.041566542489888586	0.07886401704375914	-0.19943002427630913	0.11629693929653195	0.6001580063494414	64
Women	0	CVLT_4	Model 3c APOE x PICALM x age x education	APOE	0.0277742351827035	0.8408622911501512	-1.657351452861316	1.712899923226723	0.9737697194395836	64
Women	0	CVLT_4	Model 3c APOE x PICALM x age x education	APOE_PICALM	2.2452442983707903	2.412458462332674	-2.5894304979687592	7.079919094710339	0.35608261212611814	64
Women	0	CVLT_4	Model 3c APOE x PICALM x age x education	APOE_PICALM_age	-0.762277017059263	1.257875353241797	-3.283115556751478	1.7585615226329518	0.5470053296698681	64
Women	0	CVLT_4	Model 3c APOE x PICALM x age x education	APOE_PICALM_education	0.5874185664342181	0.6311664581684315	-0.6774672814453141	1.8523044143137501	0.3560826121261178	64
Women	0	CVLT_4	Model 3c APOE x PICALM x age x education	APOE_age_education	0.16931935303145307	0.3038162881766782	-0.43954209436729075	0.7781808004301969	0.5795779139764832	64
Women	0	CVLT_4	Model 3c APOE x PICALM x age x education	PICALM	0.38293661497074083	0.6091841953775022	-0.8378957938377384	1.6037690237792201	0.532209259670112	64
Women	0	CVLT_4	Model 3c APOE x PICALM x age x education	age_c	-0.0391813270848399	0.07949075232853378	-0.19848435460856756	0.12012170043888776	0.6240424734080943	64
Women	0	CVLT_4	Model 3c APOE x PICALM x age x education	age_education	0.16387380998284112	0.13506470212413546	-0.106801901715535	0.43454952168121724	0.23020077262803285	64
Women	0	CVLT_4	Model 3c APOE x PICALM x age x education	education_c	0.2266338163737216	0.4103689418125589	-0.5957639206896241	1.0490315534370673	0.5830018585312873	64
Women	0	CVLT_5	Model 1a APOE	APOE	-0.2000000000000006	0.7059980026342159	-1.6112698983482543	1.211269898348253	0.7779006705897114	64
Women	0	CVLT_5	Model 1b APOE x age	APOE	0.1630288854949439	0.7065771927386807	-1.250335934225189	1.576393705215077	0.8183091487800335	64
Women	0	CVLT_5	Model 1b APOE x age	APOE_age	-0.33127182379801434	0.21080692869740095	-0.7529484641369408	0.09040481654091215	0.1213379629593864	64
Women	0	CVLT_5	Model 1b APOE x age	age_c	-0.07363013698630161	0.0942018696710624	-0.2620619317189989	0.11480165774639567	0.43751097888601376	64
Women	0	CVLT_5	Model 1c APOE x age x education	APOE	0.28062296738011705	0.7328059598888168	-1.1873637704835611	1.7486097052437952	0.7032117433532967	64
Women	0	CVLT_5	Model 1c APOE x age x education	APOE_age	-0.40975198357578524	0.2183436341137854	-0.8471468421337414	0.02764287498217094	0.06578171932590705	64
Women	0	CVLT_5	Model 1c APOE x age x education	APOE_age_education	0.3245075729854624	0.36366780488188655	-0.4040065818879556	1.0530217278588805	0.3760405438612596	64
Women	0	CVLT_5	Model 1c APOE x age x education	APOE_education	1.7426277674835964	1.0991673725923492	-0.4592690701224278	3.9445246050896206	0.11850521307528175	64

Sex	Cluster	Outcome	Model	Predictor	Beta	SE	CI_low	CI_high	p	N
Women	0	CVLT_5	Model 1c APOE x age x education	age_c	-0.07831560872389809	0.09438032438204928	-0.2673821175840898	0.11075090013629363	0.4101823671893233	64
Women	0	CVLT_5	Model 1c APOE x age x education	age_education	-0.20555391345850332	0.1464290603913273	-0.49888656965704425	0.08777874274003761	0.1659063411473234	64
Women	0	CVLT_5	Model 1c APOE x age x education	education_c	-0.3409677792991834	0.4904440996155185	-1.323445369967672	0.6415098113693051	0.4897907589308198	64
Women	0	CVLT_5	Model 2a PICALM	PICALM	-0.271749755620718	0.5833602964330922	-1.4378703723586177	0.8943708611171817	0.6429658605709474	64
Women	0	CVLT_5	Model 2b PICALM x age	PICALM	-0.41099258079560985	0.5830368853591994	-1.5772399927335943	0.7552548311423746	0.4835894358808208	64
Women	0	CVLT_5	Model 2b PICALM x age	PICALM_age	-0.10074870865819324	0.176457055779876	-0.453715373013728	0.2522179556973415	0.5701638050307272	64
Women	0	CVLT_5	Model 2b PICALM x age	age_c	-0.10960766541499345	0.10669478433153648	-0.32302901013364704	0.10381367930366017	0.3084025116574962	64
Women	0	CVLT_5	Model 2c PICALM x age x education	PICALM	-0.3505745471533763	0.5979878378946328	-1.5480245376134885	0.8468754433067358	0.5600173637746858	64
Women	0	CVLT_5	Model 2c PICALM x age x education	PICALM_age	-0.09353420401855533	0.1817787185821321	-0.45753980921345516	0.2704714011763445	0.6088571140108288	64
Women	0	CVLT_5	Model 2c PICALM x age x education	PICALM_education	-0.7307122227947964	0.8839740109616615	-2.5008396466564538	1.0394152010668607	0.4118984087253822	64
Women	0	CVLT_5	Model 2c PICALM x age x education	age_c	-0.1085583520770394	0.10752105429573874	-0.32386554945080914	0.10674884529673037	0.31693114044194903	64
Women	0	CVLT_5	Model 2c PICALM x age x education	age_education	-0.1878578367743201	0.14417276016681316	-0.47655880917900983	0.10084313563036962	0.1978123896226967	64
Women	0	CVLT_5	Model 2c PICALM x age x education	education_c	0.261780524846019	0.5554489857099676	-0.8504868834364152	1.3740479331284532	0.6392287537674743	64
Women	0	CVLT_5	Model 3a APOE x PICALM	APOE	-0.1666666666666572	0.9256222080226926	-2.018186753382479	1.684853420049165	0.8577129588069801	64
Women	0	CVLT_5	Model 3a APOE x PICALM	APOE_PICALM	-0.21025641025640862	1.481238246044271	-3.1731740477029864	2.752661227190169	0.8875979164931906	64
Women	0	CVLT_5	Model 3a APOE x PICALM	PICALM	-0.2564102564102533	0.6703419198457757	-1.597293738682616	1.0844732258621095	0.7034372266529052	64
Women	0	CVLT_5	Model 3b APOE x PICALM x age	APOE	-0.14821797964229655	0.9198228197583168	-1.989443400268291	1.6930074409836982	0.8725450554719585	64
Women	0	CVLT_5	Model 3b APOE x PICALM x age	APOE_PICALM	-0.20645002273378932	2.8797920564277555	-5.9709801327877905	5.558080087320212	0.9430958774586621	64
Women	0	CVLT_5	Model 3b APOE x PICALM x age	APOE_PICALM_age	0.21901806762347897	1.409051090590204	-2.601504136464845	3.039540271711803	0.8770168585173159	64
Women	0	CVLT_5	Model 3b APOE x PICALM x age	PICALM	-0.42907104522800776	0.6741375810354777	-1.778504027906099	0.9203619374500835	0.526971500277249	64
Women	0	CVLT_5	Model 3b APOE x PICALM x age	age_c	-0.14758949619490158	0.08875433672887402	-0.32525060381880233	0.030071611428999195	0.10172797377605386	64
Women	0	CVLT_5	Model 3c APOE x PICALM x age x education	APOE	-0.07856666236180199	0.957396225474528	-1.9972315735647486	1.8400982488411446	0.934894656574306	64
Women	0	CVLT_5	Model 3c APOE x PICALM x age x education	APOE_PICALM	-0.3281271533958776	2.746797722124217	-5.832832799169424	5.1765784923776685	0.9053477368188316	64

Sex	Cluster	Outcome	Model	Predictor	Beta	SE	CI_low	CI_high	p	N
Women	0	CVLT_5	Model 3c APOE x PICALM x age x education	APOE_PICALM_age	0.24014405360711732	1.4322025472968771	-2.6300539899166155	3.11034209713085	0.8674542673848261	64
Women	0	CVLT_5	Model 3c APOE x PICALM x age x education	APOE_PICALM_education	-0.08584722036520054	0.7186389389278468	-1.526031836991999	1.3543373962615979	0.9053477368188315	64
Women	0	CVLT_5	Model 3c APOE x PICALM x age x education	APOE_age_education	0.10206690000666829	0.3459217645973526	-0.5911758077608321	0.7953096077741687	0.7690607115330741	64
Women	0	CVLT_5	Model 3c APOE x PICALM x age x education	PICALM	-0.3128700217297258	0.6936101849393215	-1.7028958944935928	1.0771558510341412	0.65370917753929	64
Women	0	CVLT_5	Model 3c APOE x PICALM x age x education	age_c	-0.15137925218749665	0.0905072650307243	-0.33275986452208783	0.030001360147094536	0.10009289063298979	64
Women	0	CVLT_5	Model 3c APOE x PICALM x age x education	age_education	-0.16832982352799875	0.15378313116124506	-0.4765181052895624	0.1398584582335649	0.2784658340930102	64
Women	0	CVLT_5	Model 3c APOE x PICALM x age x education	education_c	-0.029802616354261957	0.46724140216339327	-0.9661753108965161	0.9065700781879922	0.9493733220031338	64
Women	0	CVLT_6	Model 1a APOE	APOE	-0.671428571428571	0.48563295605350615	-1.6421950183122522	0.29933787545511026	0.17175233084758396	64
Women	0	CVLT_6	Model 1b APOE x age	APOE	-0.4097559404456266	0.4840629458988924	-1.3780259968449875	0.5585141159537345	0.40064134041242594	64
Women	0	CVLT_6	Model 1b APOE x age	APOE_age	-0.2704069298952482	0.14441992180025173	-0.5592897849277615	0.018475925137265037	0.06603139687002021	64
Women	0	CVLT_6	Model 1b APOE x age	age_c	-0.03253424657534265	0.06453595588815231	-0.16162537858002207	0.09655688542933677	0.6160191494033389	64
Women	0	CVLT_6	Model 1c APOE x age x education	APOE	-0.3426945024921908	0.5025506559117168	-1.3494244396982478	0.6640354347138662	0.4981064002879917	64
Women	0	CVLT_6	Model 1c APOE x age x education	APOE_age	-0.3417372567255325	0.14973777854464923	-0.641698071855999	-0.04177644159506594	0.02629541405201919	64
Women	0	CVLT_6	Model 1c APOE x age x education	APOE_age_education	0.24590639795021607	0.24939957353116418	-0.2537009830107059	0.7455137789111381	0.3283772984934109	64
Women	0	CVLT_6	Model 1c APOE x age x education	APOE_education	1.3713504665138783	0.7537974774889291	-0.1386873341567565	2.881388267184513	0.07422001384371024	64
Women	0	CVLT_6	Model 1c APOE x age x education	age_c	-0.03403662882026193	0.06472503844067508	-0.163696461353616	0.09562320371309216	0.6010584050707922	64
Women	0	CVLT_6	Model 1c APOE x age x education	age_education	-0.05549425705080718	0.10041951672358518	-0.25665872191851785	0.14567020781690349	0.5827199735715558	64
Women	0	CVLT_6	Model 1c APOE x age x education	education_c	-0.07380822077327442	0.3363414293017018	-0.7475810673859362	0.5999646258393874	0.8271018585573261	64
Women	0	CVLT_6	Model 2a PICALM	PICALM	0.15347018572825555	0.4073959375004285	-0.6609026894902116	0.9678430609467227	0.7076752320651818	64
Women	0	CVLT_6	Model 2b PICALM x age	PICALM	0.08308995565834532	0.40953267991064357	-0.7360973720105781	0.9022772833272688	0.839907722819978	64
Women	0	CVLT_6	Model 2b PICALM x age	PICALM_age	0.00757485406488187	0.12394572754715734	-0.24035351479566983	0.2555032229254336	0.9514713155929191	64
Women	0	CVLT_6	Model 2b PICALM x age	age_c	-0.09834261297423214	0.07494380211101483	-0.2482525371103628	0.05156731116189853	0.19444461931085882	64
Women	0	CVLT_6	Model 2c PICALM x age x education	PICALM	0.07430429849064524	0.4259486637802761	-0.7786431881605161	0.9272517851418066	0.8621344625607599	64

Sex	Cluster	Outcome	Model	Predictor	Beta	SE	CI_low	CI_high	p	N
Women	0	CVLT_6	Model 2c PICALM x age x education	PICALM_age	0.0014984127071693831	0.129481566976941	-0.25778395277903005	0.2607807781933688	0.9908071749268329	64
Women	0	CVLT_6	Model 2c PICALM x age x education	PICALM_education	-0.14322301632212575	0.6296575363660759	-1.404090484077464	1.1176444514332124	0.8208786048484018	64
Women	0	CVLT_6	Model 2c PICALM x age x education	age_c	-0.09869362861317421	0.07658759343126682	-0.25205763906950884	0.05467038184316042	0.20273014912705462	64
Women	0	CVLT_6	Model 2c PICALM x age x education	age_education	-0.04041576372105987	0.10269472162306575	-0.24605839662275433	0.1652268691806346	0.6953797558409507	64
Women	0	CVLT_6	Model 2c PICALM x age x education	education_c	0.18179433712985693	0.3956481022996302	-0.6104773217591873	0.9740659960189012	0.6476336661345201	64
Women	0	CVLT_6	Model 3a APOE x PICALM	APOE	-0.7499999999999903	0.6376604689756193	-2.025510847276513	0.5255108472765325	0.24417076551942196	64
Women	0	CVLT_6	Model 3a APOE x PICALM	APOE_PICALM	0.23461538461538645	1.0204239553142371	-1.8065364307308585	2.2757671999616313	0.8189363649706779	64
Women	0	CVLT_6	Model 3a APOE x PICALM	PICALM	0.032051282051286045	0.4617980632681568	-0.8916823781144114	0.9557849422169835	0.9448977259219913	64
Women	0	CVLT_6	Model 3b APOE x PICALM x age	APOE	-0.7387021170848629	0.6367219276534639	-2.0132395322074594	0.5358352980377337	0.2507352766802212	64
Women	0	CVLT_6	Model 3b APOE x PICALM x age	APOE_PICALM	0.5652315774935737	1.9934564679441182	-3.4251050882725744	4.555568243259722	0.7776990349910501	64
Women	0	CVLT_6	Model 3b APOE x PICALM x age	APOE_PICALM_age	-0.052474079536098284	0.9753766783026133	-2.0049026301219426	1.8999544710497458	0.9572803591207075	64
Women	0	CVLT_6	Model 3b APOE x PICALM x age	PICALM	-0.07368531446210858	0.46665311066465487	-1.00779300511031	0.8604223761860926	0.8750827294600954	64
Women	0	CVLT_6	Model 3b APOE x PICALM x age	age_c	-0.0903830633210373	0.06143773687247917	-0.21336405540499329	0.0325979287629187	0.1466602441263374	64
Women	0	CVLT_6	Model 3c APOE x PICALM x age x education	APOE	-0.7351467406831677	0.6681898084757196	-2.074229040605907	0.6039355592395718	0.27603438145624587	64
Women	0	CVLT_6	Model 3c APOE x PICALM x age x education	APOE_PICALM	0.4741410935186523	1.9170560683566795	-3.367725119544352	4.316007306581657	0.8055755648780465	64
Women	0	CVLT_6	Model 3c APOE x PICALM x age x education	APOE_PICALM_age	-0.062277265145462835	0.9995685384099097	-2.0654573800857987	1.940902849794873	0.9505464973235305	64
Women	0	CVLT_6	Model 3c APOE x PICALM x age x education	APOE_PICALM_education	0.12404854190894876	0.5015553667212238	-0.8810908742993944	1.129187958117292	0.8055755648780477	64
Women	0	CVLT_6	Model 3c APOE x PICALM x age x education	APOE_age_education	0.08617339844121086	0.24142710351644023	-0.3976573289055198	0.5700041257879416	0.7225075131910115	64
Women	0	CVLT_6	Model 3c APOE x PICALM x age x education	PICALM	-0.05360397691441743	0.48408719848639553	-1.0237364016981356	0.9165284478693009	0.912231905512545	64
Women	0	CVLT_6	Model 3c APOE x PICALM x age x education	age_c	-0.0933100134640783	0.063167192931606	-0.2198998969336823	0.03327987000552571	0.14532797019631835	64
Women	0	CVLT_6	Model 3c APOE x PICALM x age x education	age_education	-0.03751599067646705	0.10732893886906462	-0.2526079907129757	0.17757600936004156	0.7280171846346626	64
Women	0	CVLT_6	Model 3c APOE x PICALM x age x education	education_c	0.12519967700786072	0.32609899090498434	-0.5283173045511278	0.7787166585668492	0.7025094984032865	64
Women	0	CVLT_7	Model 1a APOE	APOE	-0.6485714285714276	0.6144299477011148	-1.8767993932382647	0.5796565360954095	0.29526246040262044	64

Sex	Cluster	Outcome	Model	Predictor	Beta	SE	CI_low	CI_high	p	N
Women	0	CVLT_7	Model 1b APOE x age	APOE	-0.23783974851488626	0.5962151006103671	-1.4304475157178165	0.9547680186880441	0.6913713433065664	64
Women	0	CVLT_7	Model 1b APOE x age	APOE_age	-0.43557614826752894	0.17788045735742702	-0.7913900396984906	-0.07976225683656724	0.017276620873777185	64
Women	0	CVLT_7	Model 1b APOE x age	age_c	-0.043835616438356206	0.07948823961600607	-0.20283576901800077	0.11516453614128835	0.5833575327676426	64
Women	0	CVLT_7	Model 1c APOE x age x education	APOE	-0.11358276003795864	0.6217622974849693	-1.3591223118042528	1.1319567917283355	0.8557098329179403	64
Women	0	CVLT_7	Model 1c APOE x age x education	APOE_age	-0.5206442468364305	0.18525755386651366	-0.8917597222159837	-0.14952877145687737	0.006805121681948889	64
Women	0	CVLT_7	Model 1c APOE x age x education	APOE_age_education	0.2046216631747404	0.3085604406369512	-0.41349917573484185	0.8227425020843226	0.5099555332811173	64
Women	0	CVLT_7	Model 1c APOE x age x education	APOE_education	1.6830875967746854	0.9326081777599443	-0.18515107964454924	3.5513262731939204	0.07649788473295024	64
Women	0	CVLT_7	Model 1c APOE x age x education	age_c	-0.04328806849928046	0.08007867094048067	-0.20370492283847114	0.11712878583991021	0.5909481837022879	64
Women	0	CVLT_7	Model 1c APOE x age x education	age_education	-0.05716201440810967	0.12424035009389174	-0.30604534264010863	0.19172131382388927	0.6472315350998439	64
Women	0	CVLT_7	Model 1c APOE x age x education	education_c	-0.2370167959115923	0.41612605090051147	-1.070617445248859	0.5965838534256743	0.5712409001756074	64
Women	0	CVLT_7	Model 2a PICALM	PICALM	-0.15151515151514594	0.5124427738152376	-1.175873660481384	0.8728433574510922	0.7684683334052145	64
Women	0	CVLT_7	Model 2b PICALM x age	PICALM	-0.2658898949796229	0.5102875887668118	-1.286617047390785	0.7548372574315392	0.6042436015406756	64
Women	0	CVLT_7	Model 2b PICALM x age	PICALM_age	-0.018516755552662745	0.1544393635735914	-0.32744147814218605	0.29040796703686056	0.9049656239509338	64
Women	0	CVLT_7	Model 2b PICALM x age	age_c	-0.1371876213906506	0.0933817835504388	-0.3239789996424004	0.04960375686109919	0.14702831608730976	64
Women	0	CVLT_7	Model 2c PICALM x age x education	PICALM	-0.26017540744669176	0.5322618254915453	-1.3260113282926889	0.8056605133993053	0.6268510822523301	64
Women	0	CVLT_7	Model 2c PICALM x age x education	PICALM_age	-0.019366326574292825	0.16179906422292128	-0.3433633640263	0.3046307108777144	0.905146682812219	64
Women	0	CVLT_7	Model 2c PICALM x age x education	PICALM_education	-0.16349487396844142	0.7868146991384827	-1.7390641318556985	1.4120743839188157	0.8361309677742081	64
Women	0	CVLT_7	Model 2c PICALM x age x education	age_c	-0.1371644843491468	0.0957032050011516	-0.32880684670739924	0.05447787800910564	0.15725529533603888	64
Women	0	CVLT_7	Model 2c PICALM x age x education	age_education	-0.04190154686147923	0.12832645022132394	-0.29887083094310724	0.21506773722014877	0.7452245748424254	64
Women	0	CVLT_7	Model 2c PICALM x age x education	education_c	0.10427359339674958	0.4943985017192072	-0.8857423294211884	1.0942895162146875	0.8337097031921408	64
Women	0	CVLT_7	Model 3a APOE x PICALM	APOE	-0.5138888888888794	0.805318482405458	-2.1247656952723464	1.0969879174945874	0.5258244141508572	64
Women	0	CVLT_7	Model 3a APOE x PICALM	APOE_PICALM	-0.4399572649572643	1.2887207394618283	-3.017782553287366	2.1378680233728375	0.734002203789946	64
Women	0	CVLT_7	Model 3a APOE x PICALM	PICALM	-0.13782051282050967	0.5832171407556879	-1.3044284892354963	1.028787463594477	0.8139963684795016	64

Sex	Cluster	Outcome	Model	Predictor	Beta	SE	CI_low	CI_high	p	N
Women	0	CVLT_7	Model 3b APOE x PICALM x age	APOE	-0.49700476086976675	0.7982324732160696	-2.0948406589188666	1.1008311371793333	0.535967059826741	64
Women	0	CVLT_7	Model 3b APOE x PICALM x age	APOE_PICALM	-0.447130779004568	2.4991155754912806	-5.449654121365147	4.555392563356011	0.8586274953873014	64
Women	0	CVLT_7	Model 3b APOE x PICALM x age	APOE_PICALM_age	0.20650159558149284	1.222790207819748	-2.241178942852883	2.6541821340158687	0.8664806794530024	64
Women	0	CVLT_7	Model 3b APOE x PICALM x age	PICALM	-0.2958386340250425	0.5850240905517418	-1.4668915847286295	0.8752143166785444	0.6149934544159338	64
Women	0	CVLT_7	Model 3b APOE x PICALM x age	age_c	-0.1350730241529155	0.07702200053522902	-0.2892493092881271	0.019103260982296116	0.0847658871034798	64
Women	0	CVLT_7	Model 3c APOE x PICALM x age x education	APOE	-0.46663987430292786	0.8402811071696751	-2.150600843622743	1.2173210950168873	0.5809154733210931	64
Women	0	CVLT_7	Model 3c APOE x PICALM x age x education	APOE_PICALM	-0.4713641684219736	2.410791028524988	-5.30269735273775	4.359969015893803	0.8457043922913347	64
Women	0	CVLT_7	Model 3c APOE x PICALM x age x education	APOE_PICALM_age	0.21415871589863755	1.2570059397689441	-2.3049374802587854	2.7332549120560605	0.8653427139329356	64
Women	0	CVLT_7	Model 3c APOE x PICALM x age x education	APOE_PICALM_education	-0.1233220208080732	0.6307302109513044	-1.3873336097278974	1.140689568111751	0.8457043922913361	64
Women	0	CVLT_7	Model 3c APOE x PICALM x age x education	APOE_age_education	0.003381438380116397	0.30360629759729985	-0.6050591784934843	0.6118220552537171	0.9911539851426836	64
Women	0	CVLT_7	Model 3c APOE x PICALM x age x education	PICALM	-0.2807535932400951	0.6087631417766464	-1.500742191776294	0.9392350052961038	0.6464832012130919	64
Women	0	CVLT_7	Model 3c APOE x PICALM x age x education	age_c	-0.13686023274858417	0.07943581021454488	-0.2960531538153895	0.02233268831822116	0.09052478168288534	64
Women	0	CVLT_7	Model 3c APOE x PICALM x age x education	age_education	-0.025817545404429543	0.13497134862020443	-0.29630617250025093	0.24467108169139185	0.8490093131596653	64
Women	0	CVLT_7	Model 3c APOE x PICALM x age x education	education_c	0.04716054746501808	0.41008530457781	-0.7746687678776826	0.8689898628077187	0.9088625258792931	64
Women	0	CVLT_8	Model 1a APOE	APOE	-0.6085714285714288	0.5163624225438997	-1.6407652037030385	0.42362234656018094	0.24307129898563307	64
Women	0	CVLT_8	Model 1b APOE x age	APOE	-0.2931482487866116	0.5030413537878868	-1.2993807731516265	0.7130842755784031	0.5622442184304519	64
Women	0	CVLT_8	Model 1b APOE x age	APOE_age	-0.4169218372280444	0.15008211967439833	-0.7171307743360269	-0.11671290012006186	0.007290463326440394	64
Women	0	CVLT_8	Model 1b APOE x age	age_c	0.019863013698630083	0.06706618404283393	-0.11428932817305815	0.1540153555703183	0.7681227176308868	64
Women	0	CVLT_8	Model 1c APOE x age x education	APOE	-0.2102669980995867	0.5316433163153848	-1.2752765372458845	0.8547425410467112	0.6939737486856971	64
Women	0	CVLT_8	Model 1c APOE x age x education	APOE_age	-0.45823137171299416	0.15840609941848446	-0.7755569201819664	-0.1409058232440219	0.0054298902618151	64
Women	0	CVLT_8	Model 1c APOE x age x education	APOE_age_education	0.11048515827654792	0.26383731629840557	-0.4180444968839634	0.6390148134370592	0.6769915687929394	64
Women	0	CVLT_8	Model 1c APOE x age x education	APOE_education	0.792904829130062	0.7974348178600049	-0.8045490686341359	2.39035872689426	0.3243445690803385	64
Women	0	CVLT_8	Model 1c APOE x age x education	age_c	0.016864894929856267	0.06847197129374792	-0.12030094606556216	0.1540307359252747	0.8063478023990228	64

Sex	Cluster	Outcome	Model	Predictor	Beta	SE	CI_low	CI_high	p	N
Women	0	CVLT_8	Model 1c APOE x age x education	age_education	-0.09170533896885552	0.10623280313277243	-0.304515215881775	0.12110453794406398	0.3916828895758062	64
Women	0	CVLT_8	Model 1c APOE x age x education	education_c	-0.08236487686456195	0.3558122366069014	-0.7951424374998403	0.6304126837707164	0.817782488959149	64
Women	0	CVLT_8	Model 2a PICALM	PICALM	0.1593352883675525	0.431419583832229	-0.7030601716034671	1.0217307483385722	0.7131417348210931	64
Women	0	CVLT_8	Model 2b PICALM x age	PICALM	0.1154977707637771	0.43803828315729315	-0.7607092529946221	0.9917047945221763	0.7929372984643424	64
Women	0	CVLT_8	Model 2b PICALM x age	PICALM_age	0.03698605038987074	0.13257299444646192	-0.22819942165929563	0.3021715224390371	0.7812146958339495	64
Women	0	CVLT_8	Model 2b PICALM x age	age_c	-0.08494108507056777	0.08016028029106663	-0.24528551914884097	0.07540334900770543	0.2935553304528617	64
Women	0	CVLT_8	Model 2c PICALM x age x education	PICALM	0.16125021086565083	0.453993293120223	-0.7478556773573733	1.070356099088675	0.7237642577183931	64
Women	0	CVLT_8	Model 2c PICALM x age x education	PICALM_age	0.03073129111354911	0.13800668481625178	-0.24562232838225573	0.30708491060935394	0.8245808066714612	64
Women	0	CVLT_8	Model 2c PICALM x age x education	PICALM_education	-0.07905877501340175	0.6711144388523338	-1.4229422580466666	1.2648247080198631	0.9066385058220459	64
Women	0	CVLT_8	Model 2c PICALM x age x education	age_c	-0.08370944046587198	0.08163015102672017	-0.24717099833358744	0.07975211740184349	0.30947285456018264	64
Women	0	CVLT_8	Model 2c PICALM x age x education	age_education	-0.09923174164573634	0.10945618291637683	-0.31841396724161936	0.11995048395014668	0.36844097864060443	64
Women	0	CVLT_8	Model 2c PICALM x age x education	education_c	-0.009081183051046882	0.4216977306270717	-0.8535163228931614	0.8353539567910676	0.9828942401784015	64
Women	0	CVLT_8	Model 3a APOE x PICALM	APOE	-0.5694444444444356	0.6782481306054827	-1.926142702879826	0.7872538139909547	0.4044761350263093	64
Women	0	CVLT_8	Model 3a APOE x PICALM	APOE_PICALM	-0.06132478632478522	1.0853748566674848	-2.232397748185795	2.1097481755362244	0.9551304200202753	64
Women	0	CVLT_8	Model 3a APOE x PICALM	PICALM	0.10576923076923461	0.49119192480604495	-0.8767609066112892	1.0882993681497584	0.8302389491068134	64
Women	0	CVLT_8	Model 3b APOE x PICALM x age	APOE	-0.5613492052635481	0.6839934590578047	-1.9305108713005343	0.8078124607734379	0.4151810913229139	64
Women	0	CVLT_8	Model 3b APOE x PICALM x age	APOE_PICALM	-0.38150665109698423	2.1414547320762956	-4.668094029799656	3.9050807276056876	0.8592235859415842	64
Women	0	CVLT_8	Model 3b APOE x PICALM x age	APOE_PICALM_age	0.2790476277328344	1.0477906274332258	-1.8183331909237603	2.376428446389429	0.7909372611319847	64
Women	0	CVLT_8	Model 3b APOE x PICALM x age	PICALM	0.030006094845525633	0.5012983870681453	-0.9734516513225868	1.033463841013638	0.9524754920144155	64
Women	0	CVLT_8	Model 3b APOE x PICALM x age	age_c	-0.06476191344711407	0.06599899946112943	-0.19687326460454885	0.0673494377103207	0.3305420466252412	64
Women	0	CVLT_8	Model 3c APOE x PICALM x age x education	APOE	-0.5157227909712693	0.7154496751929732	-1.9495159802476612	0.9180703983051225	0.47406251175105196	64
Women	0	CVLT_8	Model 3c APOE x PICALM x age x education	APOE_PICALM	-0.43727240659014255	2.052646005723003	-4.55086692629863	3.6763221131183443	0.8320922656783639	64
Women	0	CVLT_8	Model 3c APOE x PICALM x age x education	APOE_PICALM_age	0.294144790203402	1.0702662283489037	-1.8507166614497685	2.4390062418565726	0.7844740924409035	64

Sex	Cluster	Outcome	Model	Predictor	Beta	SE	CI_low	CI_high	p	N
Women	0	CVLT_8	Model 3c APOE x PICALM x age x education	APOE_PICALM_education	-0.11440266451486303	0.537029478241483	-1.1906337888571987	0.9618284598274727	0.8320922656783636	64
Women	0	CVLT_8	Model 3c APOE x PICALM x age x education	APOE_age_education	0.04672438688093428	0.2585028095349856	-0.47132682003324133	0.5647755937951099	0.857228199079644	64
Women	0	CVLT_8	Model 3c APOE x PICALM x age x education	PICALM	0.09679899048351182	0.5183258177975535	-0.9419491607177557	1.1355471416847793	0.8525413369110137	64
Women	0	CVLT_8	Model 3c APOE x PICALM x age x education	age_c	-0.06687622566841134	0.06763489519372336	-0.20241958454970077	0.0686671332128781	0.3270995495494199	64
Women	0	CVLT_8	Model 3c APOE x PICALM x age x education	age_education	-0.09634772561148941	0.114920122214748	-0.3266527970309062	0.1339573458079274	0.4054414030583564	64
Women	0	CVLT_8	Model 3c APOE x PICALM x age x education	education_c	-0.02556841462633841	0.3491633876546999	-0.7253074801713055	0.6741706509186287	0.9418906297018189	64
Women	0	CVLT_9	Model 1a APOE	APOE	3.337142857142856	1.6193276345910048	0.10015303885040217	6.57413267543531	0.043521500481020006	64
Women	0	CVLT_9	Model 1b APOE x age	APOE	2.7621761801248006	1.65716907458175	-0.5526555104704607	6.077007870720061	0.1007657468468704	64
Women	0	CVLT_9	Model 1b APOE x age	APOE_age	0.6082594681708303	0.494415509777278	-0.3807187992067289	1.5972377355483895	0.223401615943907	64
Women	0	CVLT_9	Model 1b APOE x age	age_c	0.06232876712328675	0.22093612246609826	-0.37960927744992545	0.5042668116964989	0.778828345071254	64
Women	0	CVLT_9	Model 1c APOE x age x education	APOE	3.0927949572062	1.6367851465460823	-0.18607969636029287	6.371669610772694	0.06399850513990624	64
Women	0	CVLT_9	Model 1c APOE x age x education	APOE_age	0.8819081384777703	0.48768928846397386	-0.09505090231920788	1.8588671792747484	0.07592402329883079	64
Women	0	CVLT_9	Model 1c APOE x age x education	APOE_age_education	-2.119969236329482	0.8122833244942537	-3.7471682671974897	-0.4927702054614742	0.011596916007307478	64
Women	0	CVLT_9	Model 1c APOE x age x education	APOE_education	-6.729213373890153	2.4550848758110604	-11.64733936534244	-1.8110873824378668	0.008207031636295948	64
Women	0	CVLT_9	Model 1c APOE x age x education	age_c	0.05923915680331432	0.2108065729953636	-0.3630571540217742	0.4815354676284028	0.7797369510645856	64
Women	0	CVLT_9	Model 1c APOE x age x education	age_education	0.201877164458609	0.32706190204510294	-0.45330655530197517	0.8570608842191932	0.5395752151649735	64
Women	0	CVLT_9	Model 1c APOE x age x education	education_c	0.9258824790217316	1.0954490839343658	-1.26856573134019	3.1203306893836533	0.4015914544294803	64
Women	0	CVLT_9	Model 2a PICALM	PICALM	1.1417399804496609	1.377014711504832	-1.6108732063844333	3.894353167283755	0.4102052179646599	64
Women	0	CVLT_9	Model 2b PICALM x age	PICALM	1.372966307622436	1.3934028240520533	-1.414254326517405	4.160186941762277	0.32841717740404175	64
Women	0	CVLT_9	Model 2b PICALM x age	PICALM_age	0.11424353209550642	0.42171561700785004	-0.7293132981146958	0.9578003623057086	0.7873963312965546	64
Women	0	CVLT_9	Model 2b PICALM x age	age_c	0.22096335620872717	0.2549904088959917	-0.28909340334045064	0.731020115757905	0.38963777028987356	64
Women	0	CVLT_9	Model 2c PICALM x age x education	PICALM	1.5054652127434478	1.4459029735419549	-1.3899055492604755	4.4008359747473715	0.302181872718616	64
Women	0	CVLT_9	Model 2c PICALM x age x education	PICALM_age	0.06811731773158242	0.4395313300182256	-0.8120289889061509	0.9482636243693157	0.8773868110701006	64

Sex	Cluster	Outcome	Model	Predictor	Beta	SE	CI_low	CI_high	p	N
Women	0	CVLT_9	Model 2c PICALM x age x education	PICALM_education	1.6086475979972021	2.137402418556357	-2.6714269177669623	5.888722113761367	0.45477655098480574	64
Women	0	CVLT_9	Model 2c PICALM x age x education	age_c	0.22617460638088924	0.2599802241328661	-0.29442681254390934	0.7467760253056879	0.3879665657103124	64
Women	0	CVLT_9	Model 2c PICALM x age x education	age_education	-0.050592850320097	0.3486021109775103	-0.7486565365884917	0.6474708359482978	0.8851197031771759	64
Women	0	CVLT_9	Model 2c PICALM x age x education	education_c	-1.0017240775250338	1.3430462781927313	-3.6911278598353188	1.6876797047852512	0.45881715164400005	64
Women	0	CVLT_9	Model 3a APOE x PICALM	APOE	2.8611111111111143	2.1000653357387797	-1.3396450060548126	7.061867228277041	0.1781668235826499	64
Women	0	CVLT_9	Model 3a APOE x PICALM	APOE_PICALM	1.8619658119658131	3.3606552084928163	-4.860345482123246	8.584277106054872	0.5816048462548113	64
Women	0	CVLT_9	Model 3a APOE x PICALM	PICALM	1.160256410256412	1.5208816477814937	-1.881959837342369	4.202472657855193	0.4485215876416251	64
Women	0	CVLT_9	Model 3b APOE x PICALM x age	APOE	2.834435522563337	2.0771955334941974	-1.3235230948203882	6.992394139947063	0.1776657868427034	64
Women	0	CVLT_9	Model 3b APOE x PICALM x age	APOE_PICALM	9.96861331078761	6.503308102940459	-3.0491722236516914	22.986398845226912	0.13074909545368504	64
Women	0	CVLT_9	Model 3b APOE x PICALM x age	APOE_PICALM_age	-4.927690422667871	3.181998281590943	-11.297152017448557	1.4417711721128148	0.1269139894428329	64
Women	0	CVLT_9	Model 3b APOE x PICALM x age	PICALM	1.4099125594855417	1.5223753338228871	-1.6374527636591711	4.457277882630255	0.35821778569208873	64
Women	0	CVLT_9	Model 3b APOE x PICALM x age	age_c	0.21340470838216158	0.20043002616514524	-0.18779957934029673	0.6146089961046199	0.2914088224758016	64
Women	0	CVLT_9	Model 3c APOE x PICALM x age x education	APOE	3.435517785925944	2.1414897302619242	-0.8561235364727455	7.727159108324633	0.11438319236028986	64
Women	0	CVLT_9	Model 3c APOE x PICALM x age x education	APOE_PICALM	8.92650152989381	6.143996557037145	-3.3863427187829522	21.239345778570573	0.15193839641081722	64
Women	0	CVLT_9	Model 3c APOE x PICALM x age x education	APOE_PICALM_age	-4.6391599814644815	3.203529494981105	-11.059176553994277	1.780856591065314	0.153254817860507	64
Women	0	CVLT_9	Model 3c APOE x PICALM x age x education	APOE_PICALM_education	2.335421911890821	1.607440959689949	-0.8859617578211201	5.556805581602761	0.15193839641081713	64
Women	0	CVLT_9	Model 3c APOE x PICALM x age x education	APOE_age_education	-1.113665664079539	0.773752691569414	-2.6643007091751585	0.4369693810160804	0.15573164653008495	64
Women	0	CVLT_9	Model 3c APOE x PICALM x age x education	PICALM	1.4150954469248762	1.5514570126035554	-1.6940938856806418	4.524284779530394	0.36569290904613305	64
Women	0	CVLT_9	Model 3c APOE x PICALM x age x education	age_c	0.21321271745895612	0.2024453130482359	-0.1924967560566991	0.6189221909746113	0.29686045723483967	64
Women	0	CVLT_9	Model 3c APOE x PICALM x age x education	age_education	0.011572031897481572	0.3439798354188213	-0.6777789628302663	0.7009230266252294	0.9732847439956478	64
Women	0	CVLT_9	Model 3c APOE x PICALM x age x education	education_c	-0.5322767882748363	1.045118664208385	-2.6267413951997707	1.5621878186500984	0.6125807748071621	64
Women	0	RPM	Model 1a APOE	APOE	-1.225714285714285	1.6049127999544204	-4.43388926014546	1.9824606887168899	0.44792693869710154	64
Women	0	RPM	Model 1b APOE x age	APOE	-0.46574189981822633	1.577712126803828	-3.6216360308294098	2.6901522311929567	0.7688595999602219	64

Sex	Cluster	Outcome	Model	Predictor	Beta	SE	CI_low	CI_high	p	N
Women	0	RPM	Model 1b APOE x age	APOE_age	-0.20523771152297376	0.47070957177521705	-1.1467970427462055	0.7363216197002579	0.6643886851193257	64
Women	0	RPM	Model 1b APOE x age	age_c	-0.4712328767123304	0.21034280992224938	-0.8919811412761653	-0.05048461214849548	0.028786203727195415	64
Women	0	RPM	Model 1c APOE x age x education	APOE	-0.27783412014506714	1.5878637085066087	-3.4587073570062934	2.903039116716159	0.8617315347264313	64
Women	0	RPM	Model 1c APOE x age x education	APOE_age	-0.5116374717418866	0.473112872397118	-1.4593964423388708	0.43612149885509754	0.2841418916663407	64
Women	0	RPM	Model 1c APOE x age x education	APOE_age_education	1.2409668120306283	0.7880052031943396	-0.3375972976722639	2.8195309217335205	0.12093104163338318	64
Women	0	RPM	Model 1c APOE x age x education	APOE_education	5.959776198170402	2.381705493741834	1.188646772803085	10.73090562353772	0.01528507834647945	64
Women	0	RPM	Model 1c APOE x age x education	age_c	-0.4786253291660576	0.20450583112898688	-0.8882997373254707	-0.06895092100664446	0.022851202840858086	64
Women	0	RPM	Model 1c APOE x age x education	age_education	-0.229362660167844	0.31728643541789436	-0.8649637671350656	0.4062384467993776	0.4727569553217261	64
Women	0	RPM	Model 1c APOE x age x education	education_c	-0.21415366811207193	1.0627074961956329	-2.3430125967160333	1.9147052604918895	0.8410245263500353	64
Women	0	RPM	Model 2a PICALM	PICALM	-0.020527859237517987	1.3338135442607904	-2.6867831432481775	2.6457274247731415	0.9877701799494093	64
Women	0	RPM	Model 2b PICALM x age	PICALM	-0.424869824452875	1.286271313348924	-2.9977955310641495	2.1480558821583995	0.7423154412297266	64
Women	0	RPM	Model 2b PICALM x age	PICALM_age	-0.008854141003939553	0.38929209212523835	-0.7875542650094285	0.7698459830015494	0.9819297828689693	64
Women	0	RPM	Model 2b PICALM x age	age_c	-0.5265440890845503	0.23538551988019646	-0.9973852318346016	-0.055702946334499015	0.029017733388410894	64
Women	0	RPM	Model 2c PICALM x age x education	PICALM	-0.5912746591219289	1.3140944806487438	-3.222703466865993	2.0401541486221353	0.6544542537737149	64
Women	0	RPM	Model 2c PICALM x age x education	PICALM_age	0.01203349198231507	0.3994636607145703	-0.7878786908405538	0.811945674805184	0.9760732935226647	64
Women	0	RPM	Model 2c PICALM x age x education	PICALM_education	-2.280238454123147	1.9425568468607102	-6.170141442670969	1.6096645344246756	0.24534308217860618	64
Women	0	RPM	Model 2c PICALM x age x education	age_c	-0.5332062360236902	0.2362804308880616	-1.0063496375834295	-0.06006283446395089	0.027880144858198907	64
Women	0	RPM	Model 2c PICALM x age x education	age_education	-0.13520487409248527	0.31682354788708433	-0.769633085426402	0.4992233372414314	0.671168011203399	64
Women	0	RPM	Model 2c PICALM x age x education	education_c	1.8020494098387196	1.2206142000701092	-0.6421883649217968	4.246287184599236	0.14535446047115166	64
Women	0	RPM	Model 3a APOE x PICALM	APOE	-2.6944444444444096	2.0875112429159297	-6.870088637079425	1.4811997481906052	0.20174371035833052	64
Women	0	RPM	Model 3a APOE x PICALM	APOE_PICALM	3.6944444444444535	3.340565368088793	-2.9876811856398238	10.37657007452873	0.27316986661205805	64
Women	0	RPM	Model 3a APOE x PICALM	PICALM	-0.9166666666666528	1.511789888085312	-3.9406966871468843	2.1073633538135788	0.5465728419570066	64
Women	0	RPM	Model 3b APOE x PICALM x age	APOE	-2.6248685909760487	1.964356172595349	-6.5569546867487745	1.3072175047966774	0.18668697374615542	64

Sex	Cluster	Outcome	Model	Predictor	Beta	SE	CI_low	CI_high	p	N
Women	0	RPM	Model 3b APOE x PICALM x age	APOE_PICALM	9.83762039949034	6.150029310341826	-2.4730007990265577	22.14824159800724	0.11511988950780447	64
Women	0	RPM	Model 3b APOE x PICALM x age	APOE_PICALM_age	-2.657678886538788	3.0091427912500963	-8.681132624073703	3.365774850996127	0.38077370614963235	64
Women	0	RPM	Model 3b APOE x PICALM x age	PICALM	-1.567825295280814	1.439675435355565	-4.449648785726453	1.3139981951648245	0.2806524285367667	64
Women	0	RPM	Model 3b APOE x PICALM x age	age_c	-0.5566068277468996	0.18954207859702688	-0.9360165204557988	-0.1771971350380005	0.0047520463534523865	64
Women	0	RPM	Model 3c APOE x PICALM x age x education	APOE	-2.6656910173457535	2.048849964941835	-6.771678101329588	1.4402960666380804	0.19865992276314817	64
Women	0	RPM	Model 3c APOE x PICALM x age x education	APOE_PICALM	8.985108486408226	5.878210365710538	-2.7950883320701596	20.76530530488661	0.13210857916289181	64
Women	0	RPM	Model 3c APOE x PICALM x age x education	APOE_PICALM_age	-2.7389370717018	3.0649464252529413	-8.881226966290672	3.403352822887073	0.3754120672500405	64
Women	0	RPM	Model 3c APOE x PICALM x age x education	APOE_PICALM_education	2.3507551272579637	1.5379038747498486	-0.7312731101346346	5.432783364650562	0.1321085791628919	64
Women	0	RPM	Model 3c APOE x PICALM x age x education	APOE_age_education	0.4425965107743073	0.7402805405010028	-1.0409588445871967	1.9261518661358112	0.5523761716402673	64
Women	0	RPM	Model 3c APOE x PICALM x age x education	PICALM	-1.553540825923843	1.4843417649697404	-4.528228196629655	1.4211465447819687	0.29985310286109945	64
Women	0	RPM	Model 3c APOE x PICALM x age x education	age_c	-0.5716390431719471	0.19368763094221583	-0.9597977295493281	-0.18348035679456615	0.004642819641410451	64
Women	0	RPM	Model 3c APOE x PICALM x age x education	age_education	-0.07455320186000725	0.3290994412811641	-0.7340832203428967	0.5849768166228823	0.8216236274401136	64
Women	0	RPM	Model 3c APOE x PICALM x age x education	education_c	0.827591832214372	0.9990073580714798	-1.1762672925012092	2.8314509569299533	0.41143516506107747	64
Women	1	CVLT_1	Model 1a APOE	APOE	1.8727272727272721	2.226848444436747	-2.6751039697031525	6.420558515157697	0.4070145004618907	32
Women	1	CVLT_1	Model 1b APOE x age	APOE	2.022074419601135	2.2864800914856755	-2.6615677293707654	6.7057165685730356	0.38403244554472915	32
Women	1	CVLT_1	Model 1b APOE x age	APOE_age	-0.15605308262338013	0.8591527555906621	-1.9159477230683561	1.6038415578215959	0.857176913201812	32
Women	1	CVLT_1	Model 1b APOE x age	age_c	-0.3017782426778206	0.4517014547809608	-1.2270467286104423	0.623490243254801	0.5095448701349043	32
Women	1	CVLT_1	Model 1c APOE x age x education	APOE	1.3779776735425582	2.2338521092609325	-3.2137710986177597	5.969726445702876	0.5426904802457391	32
Women	1	CVLT_1	Model 1c APOE x age x education	APOE_age	-0.3507802746609978	0.8465232664265635	-2.090833769296921	1.3892732199749256	0.6819964906796849	32
Women	1	CVLT_1	Model 1c APOE x age x education	APOE_age_education	-0.09177390906828564	0.22147411040229859	-0.5470204628974517	0.36347264476088037	0.6819964906796805	32
Women	1	CVLT_1	Model 1c APOE x age x education	APOE_education	0.3605174145896206	0.584438051841524	-0.8408122060337202	1.5618470352129614	0.542690480245742	32
Women	1	CVLT_1	Model 1c APOE x age x education	age_c	-0.1861735354260805	0.4684518749827134	-1.1490901550404977	0.7767430841883367	0.6942995771389489	32
Women	1	CVLT_1	Model 1c APOE x age x education	age_education	0.3941976286942581	0.6241070726884416	-0.8886728320820596	1.6770680894705758	0.533150665166453	32

Sex	Cluster	Outcome	Model	Predictor	Beta	SE	CI_low	CI_high	p	N
Women	1	CVLT_1	Model 1c APOE x age x education	education_c	1.3842290440022658	1.979184222034287	-2.684042388886699	5.452500476891231	0.4905162888338731	32
Women	1	CVLT_1	Model 2a PICALM	PICALM	1.8727272727272712	2.2268484444367473	-2.6751039697031542	6.420558515157697	0.40701450046189114	32
Women	1	CVLT_1	Model 2b PICALM x age	PICALM	1.7403918348590448	2.300975710953277	-2.9729432445550366	6.453726914273126	0.4557438926189705	32
Women	1	CVLT_1	Model 2b PICALM x age	PICALM_age	0.6484893776022216	0.8376714851010972	-1.0674028749570943	2.3643816301615375	0.44532583838992745	32
Women	1	CVLT_1	Model 2b PICALM x age	age_c	-0.4829721362229269	0.4662671365700386	-1.4380770687574125	0.4721327963115586	0.30914634871476254	32
Women	1	CVLT_1	Model 2c PICALM x age x education	PICALM	1.1676497857418582	2.2570036272277023	-3.471687613148424	5.80698718463214	0.6092890162848066	32
Women	1	CVLT_1	Model 2c PICALM x age x education	PICALM_age	0.5057362408088657	0.8916130070697175	-1.3270005430998326	2.338473024717564	0.5754332767272379	32
Women	1	CVLT_1	Model 2c PICALM x age x education	PICALM_education	0.30548976952548657	0.5904951350305035	-0.9082903639051105	1.5192699029560837	0.6092890162848061	32
Women	1	CVLT_1	Model 2c PICALM x age x education	age_c	-0.4052889565919653	0.48529354102914524	-1.4028241165606152	0.5922462033766847	0.41124660617659525	32
Women	1	CVLT_1	Model 2c PICALM x age x education	age_education	0.24871183626523985	0.6318785380322345	-1.050133100306637	1.5475567728371167	0.6970805490339598	32
Women	1	CVLT_1	Model 2c PICALM x age x education	education_c	1.5163320788120305	1.9896632819872548	-2.5734793702995638	5.606143527923624	0.45285594179570077	32
Women	1	CVLT_1	Model 3a APOE x PICALM	APOE	3.717647058823532	2.977931683769494	-2.3823694699882356	9.8176635876353	0.22222321552440732	32
Women	1	CVLT_1	Model 3a APOE x PICALM	APOE_PICALM	-5.717647058823552	4.751135358757087	-15.449906659337477	4.014612541690374	0.23888734552329838	32
Women	1	CVLT_1	Model 3a APOE x PICALM	PICALM	3.7176470588235446	2.977931683769495	-2.382369469988226	9.817663587635316	0.2222232155244061	32
Women	1	CVLT_1	Model 3b APOE x PICALM x age	APOE	3.7405124999999977	3.051077915204555	-2.531067974296082	10.012092974296078	0.23120075248627622	32
Women	1	CVLT_1	Model 3b APOE x PICALM x age	APOE_PICALM	-5.413533391757839	4.893458773541802	-15.472181957558249	4.645115174042572	0.2787368721948575	32
Women	1	CVLT_1	Model 3b APOE x PICALM x age	APOE_PICALM_age	-0.49978308823528894	1.03549351925197	-2.628270500581622	1.628704324111044	0.6333817558778712	32
Women	1	CVLT_1	Model 3b APOE x PICALM x age	PICALM	3.4224750000000057	3.1367110617270786	-3.025126927896739	9.87007692789675	0.2852361200236743	32
Women	1	CVLT_1	Model 3b APOE x PICALM x age	age_c	-0.1766875000000021	0.4370451053708363	-1.0750465801045315	0.7216715801045274	0.6893150968886325	32
Women	1	CVLT_1	Model 3c APOE x PICALM x age x education	APOE	3.3029489229509896	3.349629996666878	-3.6262886617418535	10.232186507643833	0.33436212022957024	32
Women	1	CVLT_1	Model 3c APOE x PICALM x age x education	APOE_PICALM	-4.931873771073417	4.910175347969807	-15.089345373143122	5.2255978309962865	0.32562751653788535	32
Women	1	CVLT_1	Model 3c APOE x PICALM x age x education	APOE_PICALM_age	-0.48599439775909303	1.5777812358004117	-3.749883558773974	2.7778947632557878	0.7608363780945796	32
Women	1	CVLT_1	Model 3c APOE x PICALM x age x education	APOE_PICALM_education	-1.2903158121994438	1.2846388991781468	-3.9477938476246557	1.367162223225768	0.3256275165378839	32

Sex	Cluster	Outcome	Model	Predictor	Beta	SE	CI_low	CI_high	p	N
Women	1	CVLT_1	Model 3c APOE x PICALM x age x education	APOE_age_education	-0.956848709539063	5.200049586785299	-11.71397086179886	9.800273442720734	0.8556201640545242	32
Women	1	CVLT_1	Model 3c APOE x PICALM x age x education	PICALM	3.2706859579502634	3.392015535815312	-3.746232794873701	10.287604710774229	0.3449596239674936	32
Women	1	CVLT_1	Model 3c APOE x PICALM x age x education	age_c	-0.06824231383816759	0.4994323960844571	-1.1013969408880964	0.9649123132117612	0.8925045721612159	32
Women	1	CVLT_1	Model 3c APOE x PICALM x age x education	age_education	0.48964366994488273	0.6526714002456103	-0.8605099892760508	1.8397973291658163	0.460731642029784	32
Women	1	CVLT_1	Model 3c APOE x PICALM x age x education	education_c	0.858629880166049	2.117199032265171	-3.5211300107011247	5.238389771033223	0.688821252782585	32
Women	1	CVLT_10	Model 1a APOE	APOE	-0.572727272727273	0.6008625572226785	-1.7998523233658752	0.6543977779113292	0.3481187579646663	32
Women	1	CVLT_10	Model 1b APOE x age	APOE	-0.6134237494885648	0.5961163977707044	-1.83451283602333	0.6076653370462004	0.3122716130510121	32
Women	1	CVLT_10	Model 1b APOE x age	APOE_age	-0.042408126228764415	0.22399278598778408	-0.5012365487567549	0.41642029629922617	0.8512014307102707	32
Women	1	CVLT_10	Model 1b APOE x age	age_c	0.1809623430962346	0.11776470090183594	-0.06026771128246697	0.4221923974749362	0.1356052540547724	32
Women	1	CVLT_10	Model 1c APOE x age x education	APOE	-0.6333113574363372	0.5954551244824505	-1.8572868952007695	0.5906641803280952	0.2972999503877981	32
Women	1	CVLT_10	Model 1c APOE x age x education	APOE_age	-0.038676399822989815	0.22564905478639313	-0.5025046747383589	0.4251518750923793	0.865235754994969	32
Women	1	CVLT_10	Model 1c APOE x age x education	APOE_age_education	-0.010118825535084669	0.05903608991504471	-0.13146924629782658	0.11123159522765723	0.8652357549949674	32
Women	1	CVLT_10	Model 1c APOE x age x education	APOE_education	-0.16569192491066995	0.15578767791692044	-0.4859180830467138	0.15453423322537388	0.297299950387798	32
Women	1	CVLT_10	Model 1c APOE x age x education	age_c	0.185465037016913	0.12487042825057772	-0.0712098042680919	0.4421398783019179	0.14949993408035694	32
Women	1	CVLT_10	Model 1c APOE x age x education	age_education	-0.02135495882380503	0.16636184334558585	-0.3633166252875504	0.32060670763994026	0.8988482753104374	32
Women	1	CVLT_10	Model 1c APOE x age x education	education_c	0.2190603515930546	0.5275709087541336	-0.8653771823226389	1.3034978855087482	0.6813842616548031	32
Women	1	CVLT_10	Model 2a PICALM	PICALM	-0.7181818181818185	0.5956314796114561	-1.9346235830982474	0.4982599467346105	0.23733853048569334	32
Women	1	CVLT_10	Model 2b PICALM x age	PICALM	-0.6154506814102221	0.5971367510809562	-1.8386298669528618	0.6077285041324176	0.31151697273765255	32
Women	1	CVLT_10	Model 2b PICALM x age	PICALM_age	-0.1936372371089997	0.2173879657683122	-0.6389362987291505	0.25166182451115104	0.38065634380131164	32
Women	1	CVLT_10	Model 2b PICALM x age	age_c	0.20743034055727547	0.12100312130279021	-0.040433317298876253	0.4552939984134272	0.09753246373948556	32
Women	1	CVLT_10	Model 2c PICALM x age x education	PICALM	-0.6296771713661391	0.5966516963695514	-1.8561122978699598	0.5967579551376816	0.30097313449211427	32
Women	1	CVLT_10	Model 2c PICALM x age x education	PICALM_age	-0.19596308460739686	0.2357029500332449	-0.6804574371757014	0.28853126796090767	0.4133191646612171	32
Women	1	CVLT_10	Model 2c PICALM x age x education	PICALM_education	-0.1647411204155596	0.15610073451528958	-0.48561077560551263	0.15612853477439342	0.30097313449211427	32

Sex	Cluster	Outcome	Model	Predictor	Beta	SE	CI_low	CI_high	p	N
Women	1	CVLT_10	Model 2c PICALM x age x education	age_c	0.21078241419890614	0.1282900971000584	-0.05292165707661628	0.47448648547442857	0.11242085305351963	32
Women	1	CVLT_10	Model 2c PICALM x age x education	age_education	-0.003922464369692435	0.16704067156486196	-0.3472794822219413	0.3394345534825564	0.9814450189490813	32
Women	1	CVLT_10	Model 2c PICALM x age x education	education_c	0.1872919572290711	0.5259787614342801	-0.8938728710000071	1.2684567854581492	0.7246517852118384	32
Women	1	CVLT_10	Model 3a APOE x PICALM	APOE	-0.6705882352941175	0.8126046075098989	-2.3351333167731156	0.9939568461848807	0.41621497624044124	32
Women	1	CVLT_10	Model 3a APOE x PICALM	APOE_PICALM	0.6705882352941175	1.2964684530782034	-1.9851070031035065	3.3262834736917415	0.609049345944331	32
Women	1	CVLT_10	Model 3a APOE x PICALM	PICALM	-0.8705882352941173	0.8126046075098993	-2.5351333167731163	0.7939568461848815	0.29315783712105575	32
Women	1	CVLT_10	Model 3b APOE x PICALM x age	APOE	-0.6921999999999999	0.8101025722883526	-2.3573896856590233	0.9729896856590232	0.40065516516236943	32
Women	1	CVLT_10	Model 3b APOE x PICALM x age	APOE_PICALM	0.4717282945736442	1.2992796808230467	-2.198979338388641	3.1424359275359297	0.7194884367171303	32
Women	1	CVLT_10	Model 3b APOE x PICALM x age	APOE_PICALM_age	-0.08366666666666626	0.27493757512832967	-0.6488089461320339	0.4814756127987014	0.7633129413059241	32
Women	1	CVLT_10	Model 3b APOE x PICALM x age	PICALM	-0.5915999999999999	0.8328393342456069	-2.3035257692015745	1.1203257692015747	0.4838159945478028	32
Women	1	CVLT_10	Model 3b APOE x PICALM x age	age_c	0.16699999999999973	0.11604140369624476	-0.071526521399073	0.40552652139907247	0.16204170467357307	32
Women	1	CVLT_10	Model 3c APOE x PICALM x age x education	APOE	-0.8541815092633562	0.9029641311668896	-2.722105131137159	1.0137421126104467	0.3539943921505714	32
Women	1	CVLT_10	Model 3c APOE x PICALM x age x education	APOE_PICALM	0.6160685981523226	1.3236423788204958	-2.122094282267859	3.3542314785725043	0.645997489852996	32
Women	1	CVLT_10	Model 3c APOE x PICALM x age x education	APOE_PICALM_age	-0.1388888888888888	0.4253245475391606	-1.0187397510538083	0.7409619732760307	0.7469627247899531	32
Women	1	CVLT_10	Model 3c APOE x PICALM x age x education	APOE_PICALM_education	0.16118073788868886	0.346301785156525	-0.5551990854770561	0.8775605612544339	0.6459974898529963	32
Women	1	CVLT_10	Model 3c APOE x PICALM x age x education	APOE_age_education	0.28663564558004817	1.4017841558108353	-2.6131758165028667	3.186447107662963	0.8397777355084259	32
Women	1	CVLT_10	Model 3c APOE x PICALM x age x education	PICALM	-0.7472237653648034	0.9143900562897507	-2.638783714200091	1.1443361834704842	0.4222085918597728	32
Women	1	CVLT_10	Model 3c APOE x PICALM x age x education	age_c	0.15852236731951316	0.13463264302497488	-0.11998647428493106	0.4370312089239574	0.25105931002609655	32
Women	1	CVLT_10	Model 3c APOE x PICALM x age x education	age_education	-0.043160644618582406	0.17594148143128918	-0.4071233291698191	0.3208020399326543	0.8083901141418683	32
Women	1	CVLT_10	Model 3c APOE x PICALM x age x education	education_c	0.3391391947848452	0.5707361071458734	-0.8415183968034028	1.5197967863730932	0.5581694079901502	32
Women	1	CVLT_11	Model 1a APOE	APOE	-0.3363636363636365	0.3205367399423857	-0.9909869915805634	0.31825971885329046	0.3023855693202774	32
Women	1	CVLT_11	Model 1b APOE x age	APOE	-0.3430823493448351	0.33081929929596543	-1.020734964666389	0.33457026597671885	0.3085775254037065	32
Women	1	CVLT_11	Model 1b APOE x age	APOE_age	0.05964233503049857	0.12430648911008955	-0.19498796503410162	0.31427263509509873	0.635095969505987	32

Sex	Cluster	Outcome	Model	Predictor	Beta	SE	CI_low	CI_high	p	N
Women	1	CVLT_11	Model 1b APOE x age	age_c	-0.04759414225941418	0.06535441061483734	-0.18146658371066587	0.08627829919183753	0.4725094499572615	32
Women	1	CVLT_11	Model 1c APOE x age x education	APOE	-0.294083972867299	0.3289508957414988	-0.9702522229318915	0.3820842771972936	0.37952016728407034	32
Women	1	CVLT_11	Model 1c APOE x age x education	APOE_age	0.039640751633200924	0.12465667964436877	-0.21659472309927263	0.29587622636567446	0.7530254942271238	32
Women	1	CVLT_11	Model 1c APOE x age x education	APOE_age_education	0.010371126880779336	0.032613666186026714	-0.056667224066670144	0.07740947782822881	0.7530254942271233	32
Women	1	CVLT_11	Model 1c APOE x age x education	APOE_education	-0.076940574296677	0.08606273435097365	-0.25384505832520443	0.0999639097318504	0.37952016728407145	32
Women	1	CVLT_11	Model 1c APOE x age x education	age_c	-0.0440222203034176	0.06898293009125445	-0.18581866386983426	0.09777422326299906	0.5289500865779508	32
Women	1	CVLT_11	Model 1c APOE x age x education	age_education	0.052426690183748556	0.09190428486664277	-0.13648527289705614	0.24133865326455328	0.5732707065489453	32
Women	1	CVLT_11	Model 1c APOE x age x education	education_c	-0.1382946477039888	0.2914492056016281	-0.7373770696872142	0.46078777427923656	0.6390997618940168	32
Women	1	CVLT_11	Model 2a PICALM	PICALM	-0.3363636363636365	0.32053673994238574	-0.9909869915805636	0.3182597188532906	0.3023855693202775	32
Women	1	CVLT_11	Model 2b PICALM x age	PICALM	-0.3731462800335213	0.333548904408825	-1.0563902379625376	0.3100976778954951	0.27276769133581397	32
Women	1	CVLT_11	Model 2b PICALM x age	PICALM_age	0.03503789900715275	0.1214286638402755	-0.21369744322192147	0.283773241236227	0.775053316510417	32
Women	1	CVLT_11	Model 2b PICALM x age	age_c	-0.05572755417956667	0.06758997577612168	-0.19417934327314187	0.08272423491400852	0.41662742427879784	32
Women	1	CVLT_11	Model 2c PICALM x age x education	PICALM	-0.3172343963601969	0.3320477138334905	-0.9997682471790003	0.36529945445860645	0.34818084545274874	32
Women	1	CVLT_11	Model 2c PICALM x age x education	PICALM_age	0.01644331671755793	0.1311730548636088	-0.2531867591113064	0.2860733925464223	0.9012060622928939	32
Women	1	CVLT_11	Model 2c PICALM x age x education	PICALM_education	-0.08299737114074915	0.08687294838666902	-0.26156727397125	0.09557253168975172	0.34818084545274897	32
Women	1	CVLT_11	Model 2c PICALM x age x education	age_c	-0.049772874620167946	0.071395813853794	-0.196529071792508	0.09698332255217212	0.4919022346290529	32
Women	1	CVLT_11	Model 2c PICALM x age x education	age_education	0.04831251710208384	0.09296122586730973	-0.14277201932050046	0.23939705352466814	0.6076651765730523	32
Women	1	CVLT_11	Model 2c PICALM x age x education	education_c	-0.12698234520275004	0.29271691729348526	-0.7286705858882994	0.47470589548279934	0.6680077469475988	32
Women	1	CVLT_11	Model 3a APOE x PICALM	APOE	-0.5647058823529408	0.4284494800960347	-1.4423448572801174	0.31293309257423585	0.19817806507041805	32
Women	1	CVLT_11	Model 3a APOE x PICALM	APOE_PICALM	0.7647058823529408	0.6835688962980682	-0.6355215267331146	2.164933291438996	0.27277575238776586	32
Women	1	CVLT_11	Model 3a APOE x PICALM	PICALM	-0.5647058823529408	0.42844948009603484	-1.4423448572801179	0.3129330925742362	0.19817806507041816	32
Women	1	CVLT_11	Model 3b APOE x PICALM x age	APOE	-0.5574749999999999	0.43802556481582344	-1.4578494433570959	0.3428994433570961	0.21438832668558544	32
Women	1	CVLT_11	Model 3b APOE x PICALM x age	APOE_PICALM	0.8283607700638393	0.7025255017258021	-0.6157010801308997	2.2724226202585784	0.2490289353994943	32

Sex	Cluster	Outcome	Model	Predictor	Beta	SE	CI_low	CI_high	p	N
Women	1	CVLT_11	Model 3b APOE x PICALM x age	APOE_PICALM_age	0.04607107843137277	0.14865980031947493	-0.2595035174680788	0.35164567433082433	0.7591002043135606	32
Women	1	CVLT_11	Model 3b APOE x PICALM x age	PICALM	-0.6580499999999996	0.4503194191240212	-1.5836948228019825	0.2675948228019833	0.155915528651701	32
Women	1	CVLT_11	Model 3b APOE x PICALM x age	age_c	-0.055874999999999994	0.06274403160144044	-0.18484720405589944	0.07309720405589944	0.3813518595339409	32
Women	1	CVLT_11	Model 3c APOE x PICALM x age x education	APOE	-0.6213126755644667	0.48637797210816963	-1.6274621691062117	0.3848368179772783	0.21419598619073568	32
Women	1	CVLT_11	Model 3c APOE x PICALM x age x education	APOE_PICALM	0.8390291093843085	0.7129746063945904	-0.635871236169381	2.3139294549379983	0.2513108010380535	32
Women	1	CVLT_11	Model 3c APOE x PICALM x age x education	APOE_PICALM_age	-0.065359477124183	0.22909934490153896	-0.5392875804967683	0.40856862624840223	0.7779762286650178	32
Women	1	CVLT_11	Model 3c APOE x PICALM x age x education	APOE_PICALM_education	0.21951342978077792	0.18653405399858464	-0.16636166062571045	0.6053885201872663	0.25131080103805453	32
Women	1	CVLT_11	Model 3c APOE x PICALM x age x education	APOE_age_education	0.4390407746138029	0.7550653580841119	-1.1229309247506731	2.001012473978279	0.5665856270938834	32
Women	1	CVLT_11	Model 3c APOE x PICALM x age x education	PICALM	-0.6638258096442518	0.4925325003988287	-1.6827069149730078	0.35505529568450434	0.19085553061231997	32
Women	1	CVLT_11	Model 3c APOE x PICALM x age x education	age_c	-0.06795780741248433	0.07251932788230286	-0.2179754669386839	0.08205985211371522	0.3584441107525246	32
Women	1	CVLT_11	Model 3c APOE x PICALM x age x education	age_education	0.03305474606403839	0.09477016638265719	-0.16299227986412412	0.2291017719922009	0.7304208152462943	32
Women	1	CVLT_11	Model 3c APOE x PICALM x age x education	education_c	-0.03161785422162408	0.3074246925443099	-0.6675742840841444	0.6043385756408962	0.9189754970751859	32
Women	1	CVLT_12	Model 1a APOE	APOE	0.11818181818181772	0.22425880366060047	-0.3398157596172937	0.5761793959809292	0.6020770498759613	32
Women	1	CVLT_12	Model 1b APOE x age	APOE	0.13250381598398941	0.2248217837608795	-0.3280227315029422	0.5930303634709211	0.5603371469240496	32
Women	1	CVLT_12	Model 1b APOE x age	APOE_age	0.011430659878005733	0.08447755821458361	-0.16161377369017083	0.1844750934461823	0.8933348268551913	32
Women	1	CVLT_12	Model 1b APOE x age	age_c	-0.05962343096234268	0.044414262415578123	-0.150601923291981	0.03135506136729563	0.19023732094260298	32
Women	1	CVLT_12	Model 1c APOE x age x education	APOE	-0.017588230744770422	0.1520183450403009	-0.3300664141888784	0.2948899526993375	0.9087808036221366	32
Women	1	CVLT_12	Model 1c APOE x age x education	APOE_age	-0.06565643987688685	0.05760769276836858	-0.1840707482545625	0.052757868500788785	0.2647957291959189	32
Women	1	CVLT_12	Model 1c APOE x age x education	APOE_age_education	-0.017177556944534544	0.015071780084747593	-0.04815804460148457	0.013802930712415475	0.26479572919591354	32
Women	1	CVLT_12	Model 1c APOE x age x education	APOE_education	-0.00460157199717888	0.03977224143496251	-0.0863545851075561	0.07715144111319834	0.9087808036221254	32
Women	1	CVLT_12	Model 1c APOE x age x education	age_c	-0.020643135119487566	0.031879137598531025	-0.08617164093181488	0.04488537069283975	0.5229572438705413	32
Women	1	CVLT_12	Model 1c APOE x age x education	age_education	0.16283022967498073	0.04247180192668762	0.07552819050246533	0.2501322688474961	0.0007200152161079662	32
Women	1	CVLT_12	Model 1c APOE x age x education	education_c	0.33217224181813343	0.13468765846950856	0.0553177948121818	0.6090266888240851	0.020565928232369957	32

Sex	Cluster	Outcome	Model	Predictor	Beta	SE	CI_low	CI_high	p	N
Women	1	CVLT_12	Model 2a PICALM	PICALM	0.40909090909090917	0.2125536717181841	-0.02500160014483349	0.8431834183266518	0.06380320784381839	32
Women	1	CVLT_12	Model 2b PICALM x age	PICALM	0.3792291479849963	0.21565333450420213	-0.06251668256537007	0.8209749785353626	0.0895872956853122	32
Women	1	CVLT_12	Model 2b PICALM x age	PICALM_age	0.0650154798761613	0.0785087161595018	-0.0958023349981379	0.2258332947504605	0.4145996924920158	32
Women	1	CVLT_12	Model 2b PICALM x age	age_c	-0.06501547987616507	0.04369974975937363	-0.15453035937793103	0.024499399625600868	0.14798610879510357	32
Women	1	CVLT_12	Model 2c PICALM x age x education	PICALM	0.26212581359335063	0.1487360111204671	-0.0436054358510829	0.5678570630377842	0.08976330393165843	32
Women	1	CVLT_12	Model 2c PICALM x age x education	PICALM_age	-0.01100330678935113	0.05875708861733942	-0.13178023217124024	0.109773618592538	0.8529044409921431	32
Women	1	CVLT_12	Model 2c PICALM x age x education	PICALM_education	0.06857942797500483	0.038913491281517554	-0.011408398914527204	0.14856725486453687	0.08976330393165709	32
Women	1	CVLT_12	Model 2c PICALM x age x education	age_c	-0.030557790293751275	0.03198073084351305	-0.0962951240119064	0.03517954342440385	0.34812215892572773	32
Women	1	CVLT_12	Model 2c PICALM x age x education	age_education	0.1588557488509742	0.04164064785974102	0.07326217133111529	0.2444493263708331	0.0007561642334586295	32
Women	1	CVLT_12	Model 2c PICALM x age x education	education_c	0.2689930605851436	0.13111834490011012	-0.0005245573031620876	0.5385106784734492	0.050415571750670705	32
Women	1	CVLT_12	Model 3a APOE x PICALM	APOE	0.011764705882354454	0.2934782966895175	-0.5893983330183568	0.6129277447830657	0.9683081286729954	32
Women	1	CVLT_12	Model 3a APOE x PICALM	APOE_PICALM	-0.011764705882359117	0.4682293821678211	-0.9708891163132986	0.9473597045485803	0.9801327232718482	32
Women	1	CVLT_12	Model 3a APOE x PICALM	PICALM	0.4117647058823577	0.29347829668951764	-0.18939833301835374	1.0129277447830691	0.17159427537691246	32
Women	1	CVLT_12	Model 3b APOE x PICALM x age	APOE	0.018987499999998825	0.29486890665069293	-0.5871242181609371	0.6250992181609347	0.9491498289500839	32
Women	1	CVLT_12	Model 3b APOE x PICALM x age	APOE_PICALM	0.05025893895349176	0.47292428394040953	-0.9218508489351199	1.0223687268421036	0.9161817218263524	32
Women	1	CVLT_12	Model 3b APOE x PICALM x age	APOE_PICALM_age	0.0558125000000016	0.1000744164362764	-0.14989340903977053	0.26151840903977375	0.581813070518061	32
Women	1	CVLT_12	Model 3b APOE x PICALM x age	PICALM	0.3185250000000046	0.30314485141182895	-0.30459816625002867	0.9416481662500379	0.30304883118681497	32
Women	1	CVLT_12	Model 3b APOE x PICALM x age	age_c	-0.0558125000000006	0.042237863456559874	-0.14263367176033734	0.031008671760336143	0.19788368212083718	32
Women	1	CVLT_12	Model 3c APOE x PICALM x age x education	APOE	-0.09692768947288927	0.21109258055560634	-0.5336059627422387	0.33975058379646017	0.6504212277770642	32
Women	1	CVLT_12	Model 3c APOE x PICALM x age x education	APOE_PICALM	0.050834783840896014	0.30943763526564494	-0.5892857353014517	0.6909553029832437	0.8709449733365158	32
Women	1	CVLT_12	Model 3c APOE x PICALM x age x education	APOE_PICALM_age	0.11111111111111349	0.09943125448146184	-0.09457811018547488	0.3168003324077019	0.2753321796223559	32
Women	1	CVLT_12	Model 3c APOE x PICALM x age x education	APOE_PICALM_education	0.013299798097909432	0.08095752085438382	-0.15417359353816984	0.1807731897339887	0.87094497333651	32
Women	1	CVLT_12	Model 3c APOE x PICALM x age x education	APOE_age_education	-0.5559703419816966	0.3277054144439551	-1.23388064154671	0.121939957583317	0.10327538385538243	32

Sex	Cluster	Outcome	Model	Predictor	Beta	SE	CI_low	CI_high	p	N
Women	1	CVLT_12	Model 3c APOE x PICALM x age x education	PICALM	0.28354091497716194	0.21376370329035219	-0.15866299666578282	0.7257448266201068	0.19772098940772595	32
Women	1	CVLT_12	Model 3c APOE x PICALM x age x education	age_c	-0.010419491852197593	0.03147406531689908	-0.07552855660082684	0.05468957289643166	0.7436038554610077	32
Women	1	CVLT_12	Model 3c APOE x PICALM x age x education	age_education	0.1711045972587496	0.041131136952374435	0.0860183577770324	0.25619083674046683	0.00037788888041008603	32
Women	1	CVLT_12	Model 3c APOE x PICALM x age x education	education_c	0.28660722281559436	0.1334251865774458	0.010596195180580692	0.5626182504506081	0.042473565150139794	32
Women	1	CVLT_13	Model 1a APOE	APOE	-0.40000000000000013	0.42010099795745376	-1.2579606969931703	0.45796069699317	0.3486288531915377	32
Women	1	CVLT_13	Model 1b APOE x age	APOE	-0.4031692963348969	0.4248529387634098	-1.273441090310563	0.4671024976407692	0.35075921203356575	32
Women	1	CVLT_13	Model 1b APOE x age	APOE_age	-0.08521323788879365	0.15963995243982254	-0.4122208565823792	0.24179438080479193	0.5977035856129458	32
Women	1	CVLT_13	Model 1b APOE x age	age_c	0.10930962343096248	0.08393105683360853	-0.06261535280542377	0.28123459966734876	0.20339997489196066	32
Women	1	CVLT_13	Model 1c APOE x age x education	APOE	-0.384529182132122	0.42531722035817054	-1.2587812493400985	0.48972288507585454	0.3742437924835583	32
Women	1	CVLT_13	Model 1c APOE x age x education	APOE_age	-0.07032996829997981	0.16117491446834595	-0.40162974976041166	0.2609698131604521	0.6661791196123381	32
Women	1	CVLT_13	Model 1c APOE x age x education	APOE_age_education	-0.01840028240406453	0.042167855529509114	-0.10507755080941009	0.06827698600128101	0.6661791196123372	32
Women	1	CVLT_13	Model 1c APOE x age x education	APOE_education	-0.10060356509270646	0.11127485416347502	-0.3293323036064216	0.12812517342100865	0.37424379248355844	32
Women	1	CVLT_13	Model 1c APOE x age x education	age_c	0.10652640014370221	0.0891915129534423	-0.07680938040919538	0.2898621806965998	0.24312149827507057	32
Women	1	CVLT_13	Model 1c APOE x age x education	age_education	-0.027848783459652436	0.11882768973884504	-0.2721025978437698	0.21640503092446492	0.8165391368381231	32
Women	1	CVLT_13	Model 1c APOE x age x education	education_c	0.04926538718721263	0.3768293918843506	-0.7253185211769609	0.8238492955513862	0.896990012122097	32
Women	1	CVLT_13	Model 2a PICALM	PICALM	-0.5454545454545456	0.4146092454905958	-1.3921995876478275	0.3012904967387362	0.19827871348528375	32
Women	1	CVLT_13	Model 2b PICALM x age	PICALM	-0.49995090948363274	0.427086397520786	-1.3747977363288135	0.3748959173615479	0.2516231171263137	32
Women	1	CVLT_13	Model 2b PICALM x age	PICALM_age	-0.09907120743034063	0.15548104014079223	-0.41755968046849246	0.21941726560781116	0.5291761212179382	32
Women	1	CVLT_13	Model 2b PICALM x age	age_c	0.09907120743034059	0.08654430843927918	-0.07820677205840931	0.2763491869190905	0.26200601151528774	32
Women	1	CVLT_13	Model 2c PICALM x age x education	PICALM	-0.4814330152976163	0.42712252193683947	-1.3593959330461751	0.39652990245094266	0.2699725983474687	32
Women	1	CVLT_13	Model 2c PICALM x age x education	PICALM_age	-0.08427443105135493	0.1687316721945552	-0.43110735047870186	0.26255848837599205	0.6216569393441955	32
Women	1	CVLT_13	Model 2c PICALM x age x education	PICALM_education	-0.1259563121418182	0.1117471714369638	-0.35565591271556907	0.10374328843193265	0.2699725983474687	32
Women	1	CVLT_13	Model 2c PICALM x age x education	age_c	0.09379197324584251	0.09183848826092991	-0.09498474297495663	0.28256868946664165	0.31653532966525594	32

Sex	Cluster	Outcome	Model	Predictor	Beta	SE	CI_low	CI_high	p	N
Women	1	CVLT_13	Model 2c PICALM x age x education	age_education	-0.03637816127670822	0.1195786978214115	-0.2821756948831997	0.20941937232978325	0.7633823057635224	32
Women	1	CVLT_13	Model 2c PICALM x age x education	education_c	0.058907820887402984	0.37653018743766	-0.7150610639284252	0.8328767057032311	0.8768874935259852	32
Women	1	CVLT_13	Model 3a APOE x PICALM	APOE	-0.44705882352941156	0.5662094828310891	-1.606886371912806	0.7127687248539829	0.4364178363873962	32
Women	1	CVLT_13	Model 3a APOE x PICALM	APOE_PICALM	0.44705882352941184	0.9033578268448217	-1.4033858005761526	2.297503447634976	0.6245420110064115	32
Women	1	CVLT_13	Model 3a APOE x PICALM	PICALM	-0.6470588235294116	0.5662094828310894	-1.8068863719128068	0.5127687248539835	0.2628033726132357	32
Women	1	CVLT_13	Model 3b APOE x PICALM x age	APOE	-0.4582124999999999	0.5757158188138068	-1.641613313864165	0.7251883138641653	0.4332968822596911	32
Women	1	CVLT_13	Model 3b APOE x PICALM x age	APOE_PICALM	0.3512798691860466	0.9233594496561218	-1.5467126620311917	2.249272400403285	0.7067116927361328	32
Women	1	CVLT_13	Model 3b APOE x PICALM x age	APOE_PICALM_age	-0.0861874999999998	0.19538996245940526	-0.4878173198506327	0.3154423198506331	0.6627833294941146	32
Women	1	CVLT_13	Model 3b APOE x PICALM x age	PICALM	-0.5030749999999998	0.5918741597142921	-1.7196897592647398	0.71353975926474	0.403101413306004	32
Women	1	CVLT_13	Model 3b APOE x PICALM x age	age_c	0.08618749999999988	0.08246717641763943	-0.08332620884821308	0.25570120884821285	0.3055907013394136	32
Women	1	CVLT_13	Model 3c APOE x PICALM x age x education	APOE	-0.48604342791485605	0.6443305793827966	-1.818942784580788	0.846855928751076	0.4583002683628574	32
Women	1	CVLT_13	Model 3c APOE x PICALM x age x education	APOE_PICALM	0.3681962595703827	0.9445151046463922	-1.585682099812113	2.3220746189528785	0.7002519443236329	32
Women	1	CVLT_13	Model 3c APOE x PICALM x age x education	APOE_PICALM_age	-0.05555555555555552	0.30349999815328454	-0.6833931364975123	0.5722820253864014	0.8563631184643807	32
Women	1	CVLT_13	Model 3c APOE x PICALM x age x education	APOE_PICALM_education	0.09633041674806522	0.24711150993655598	-0.41485868890433175	0.6075195224004621	0.7002519443236329	32
Women	1	CVLT_13	Model 3c APOE x PICALM x age x education	APOE_age_education	-0.07306442227609194	1.0002749457124167	-2.1422908012355752	1.9961619566833915	0.9424023573854335	32
Women	1	CVLT_13	Model 3c APOE x PICALM x age x education	PICALM	-0.5623266334611884	0.6524838079555458	-1.912092228463624	0.7874389615412473	0.39768063237992834	32
Women	1	CVLT_13	Model 3c APOE x PICALM x age x education	age_c	0.08625057187156604	0.09607018251324778	-0.11248574231881023	0.28498688606194233	0.3786014034321252	32
Women	1	CVLT_13	Model 3c APOE x PICALM x age x education	age_education	-0.04425875119843715	0.1255470430720106	-0.30397259731495896	0.21545509491808468	0.7276528853852301	32
Women	1	CVLT_13	Model 3c APOE x PICALM x age x education	education_c	0.13963126521705274	0.4072617215888222	-0.7028537945800247	0.9821163250141302	0.7348221666785406	32
Women	1	CVLT_2	Model 1a APOE	APOE	-0.5000000000000009	0.6286107819243282	-1.78379448565804	0.7837944856580383	0.4326266902755017	32
Women	1	CVLT_2	Model 1b APOE x age	APOE	-0.42085441845224725	0.6401202341126743	-1.732081277616293	0.8903724407117984	0.5162529607527988	32
Women	1	CVLT_2	Model 1b APOE x age	APOE_age	-0.1856316479306344	0.2405273787841722	-0.6783296484294227	0.30706635256815384	0.446716148596231	32
Women	1	CVLT_2	Model 1b APOE x age	age_c	-0.040271966527196223	0.1264577995059423	-0.2993090261708795	0.21876509311648706	0.7524980781006354	32

Sex	Cluster	Outcome	Model	Predictor	Beta	SE	CI_low	CI_high	p	N
Women	1	CVLT_2	Model 1c APOE x age x education	APOE	-0.6483827360573268	0.6042485126772262	-1.8904333421215354	0.5936678700068817	0.2931120467358442	32
Women	1	CVLT_2	Model 1c APOE x age x education	APOE_age	-0.2345602681926391	0.22898132896279824	-0.7052381307752382	0.2361175943899601	0.31510016596151014	32
Women	1	CVLT_2	Model 1c APOE x age x education	APOE_age_education	-0.06136751202714441	0.05990790583329023	-0.18450997607491745	0.06177495202062863	0.3151001659615065	32
Women	1	CVLT_2	Model 1c APOE x age x education	APOE_education	-0.169635018154534	0.1580882736655536	-0.4945901185783099	0.15532008226924196	0.2931120467358425	32
Women	1	CVLT_2	Model 1c APOE x age x education	age_c	0.0024277069685985087	0.12671445327363817	-0.25803758203690125	0.26289299597409826	0.9848606335363924	32
Women	1	CVLT_2	Model 1c APOE x age x education	age_education	0.08517593077798202	0.16881859316465522	-0.2618356572622409	0.43218751881820494	0.6181306124884789	32
Women	1	CVLT_2	Model 1c APOE x age x education	education_c	0.7831184350499599	0.5353618162637094	-0.31733353860541047	1.8835704087053302	0.15551097571242978	32
Women	1	CVLT_2	Model 2a PICALM	PICALM	-0.06363636363636394	0.6350983091293453	-1.3606801474147145	1.2334074201419867	0.9208527794740814	32
Women	1	CVLT_2	Model 2b PICALM x age	PICALM	-0.20659953717235524	0.6528132016342629	-1.5438267616581982	1.1306276873134877	0.753988845274548	32
Women	1	CVLT_2	Model 2b PICALM x age	PICALM_age	-0.18424255364577755	0.23765700850445837	-0.6710608671640031	0.302575759872448	0.4446929574607903	32
Women	1	CVLT_2	Model 2b PICALM x age	age_c	-0.043343653250776604	0.13228533477870674	-0.3143178777662543	0.2276305712647011	0.7456115865526585	32
Women	1	CVLT_2	Model 2c PICALM x age x education	PICALM	-0.40032803811273476	0.6238613649246767	-1.6826934393473307	0.8820373631218612	0.5266892694593321	32
Women	1	CVLT_2	Model 2c PICALM x age x education	PICALM_age	-0.23364556214219412	0.24645193337963195	-0.7402347664144792	0.2729436421300909	0.35183964006171053	32
Women	1	CVLT_2	Model 2c PICALM x age x education	PICALM_education	-0.10473698671554088	0.16321954314889797	-0.44023956261994096	0.2307655891888592	0.5266892694593328	32
Women	1	CVLT_2	Model 2c PICALM x age x education	age_c	-0.014318953903083464	0.13414063107530308	-0.29004896999650165	0.2614110621903347	0.9158100177006692	32
Women	1	CVLT_2	Model 2c PICALM x age x education	age_education	0.07789042607957794	0.17465838443848852	-0.28112502483953905	0.436905876998695	0.6593188372140997	32
Women	1	CVLT_2	Model 2c PICALM x age x education	education_c	0.6915757899099979	0.5499654656584441	-0.43889441498786863	1.8220459948078644	0.2197545899568644	32
Women	1	CVLT_2	Model 3a APOE x PICALM	APOE	1.0823529411764703	0.7205342116603556	-0.39359448389640805	2.5583003662493486	0.14425245282435398	32
Women	1	CVLT_2	Model 3a APOE x PICALM	APOE_PICALM	-4.082352941176474	1.149574917675869	-6.437150412572404	-1.727555469780544	0.0013796326844387136	32
Women	1	CVLT_2	Model 3a APOE x PICALM	PICALM	1.682352941176473	0.7205342116603558	0.20640551610359426	3.158300366249352	0.026946258136069927	32
Women	1	CVLT_2	Model 3b APOE x PICALM x age	APOE	1.0821750000000006	0.7161096848801372	-0.38980953856839085	2.554159538568392	0.14280083372359287	32
Women	1	CVLT_2	Model 3b APOE x PICALM x age	APOE_PICALM	-4.029999290355672	1.1485295746896798	-6.390835642282285	-1.6691629384290594	0.0016585596183407063	32
Women	1	CVLT_2	Model 3b APOE x PICALM x age	APOE_PICALM_age	-0.3396102941176464	0.24303769303028042	-0.8391814268412371	0.15996083860594423	0.17411797132789641	32

Sex	Cluster	Outcome	Model	Predictor	Beta	SE	CI_low	CI_high	p	N
Women	1	CVLT_2	Model 3b APOE x PICALM x age	PICALM	1.6846500000000009	0.7362083933614731	0.17135197446952222	3.1979480255304793	0.030496490493366796	32
Women	1	CVLT_2	Model 3b APOE x PICALM x age	age_c	0.001374999999999571	0.10257759433997711	-0.2094762649109897	0.21222626491098884	0.9894074172977028	32
Women	1	CVLT_2	Model 3c APOE x PICALM x age x education	APOE	0.9318749023924181	0.7487741933941	-0.6170825312576654	2.4808323360425018	0.2258362598830151	32
Women	1	CVLT_2	Model 3c APOE x PICALM x age x education	APOE_PICALM	-3.7377832465389815	1.0976175246991997	-6.00837809233729	-1.4671884007406728	0.0024267894658689996	32
Women	1	CVLT_2	Model 3c APOE x PICALM x age x education	APOE_PICALM_age	-0.19537815126050306	0.3526962301401585	-0.9249858919060473	0.5342295893850412	0.5849551827787296	32
Women	1	CVLT_2	Model 3c APOE x PICALM x age x education	APOE_PICALM_education	-0.9779084075247327	0.2871673756480463	-1.5719593846231272	-0.38385743042633835	0.0024267894658690043	32
Women	1	CVLT_2	Model 3c APOE x PICALM x age x education	APOE_age_education	-0.90227862946307	1.162415830652627	-3.3069189840141977	1.5023617250880577	0.44553433309415535	32
Women	1	CVLT_2	Model 3c APOE x PICALM x age x education	PICALM	1.5676127131353774	0.7582490302922121	-0.0009449139714647892	3.1361703402422196	0.05012706349099617	32
Women	1	CVLT_2	Model 3c APOE x PICALM x age x education	age_c	0.05895116553771085	0.111642805296448	-0.1719995732873175	0.2899019043627392	0.602533824742751	32
Women	1	CVLT_2	Model 3c APOE x PICALM x age x education	age_education	0.13092242848719327	0.14589775639596006	-0.17089007562437275	0.4327349325987593	0.37882578983458215	32
Women	1	CVLT_2	Model 3c APOE x PICALM x age x education	education_c	0.531203105410864	0.47327734681639866	-0.4478456799198107	1.5102518907415388	0.2732762253563548	32
Women	1	CVLT_3	Model 1a APOE	APOE	0.6090909090909087	0.41146114698971725	-0.2312248582443056	1.4494066764261229	0.14921638283485883	32
Women	1	CVLT_3	Model 1b APOE x age	APOE	0.5885450066296108	0.4233637245458506	-0.2786762703071439	1.4557662835663656	0.17543234137173802	32
Women	1	CVLT_3	Model 1b APOE x age	APOE_age	0.11252331501739199	0.15908037507746317	-0.21333806141074974	0.4383846914455337	0.48520360415530106	32
Women	1	CVLT_3	Model 1b APOE x age	age_c	-0.06433054393305371	0.08363685780206791	-0.2356528807721229	0.10699179290601549	0.4482354488887549	32
Women	1	CVLT_3	Model 1c APOE x age x education	APOE	0.5890865877524314	0.4223640249454641	-0.279095099346662	1.4572682748515247	0.17489823877384028	32
Women	1	CVLT_3	Model 1c APOE x age x education	APOE_age	0.09469073880843326	0.16005579444388415	-0.23430865849634264	0.42369013611320916	0.5592206136873188	32
Women	1	CVLT_3	Model 1c APOE x age x education	APOE_age_education	0.024773739804531736	0.041875062499853406	-0.06130168390892707	0.11084916351799054	0.5592206136873221	32
Women	1	CVLT_3	Model 1c APOE x age x education	APOE_education	0.15412149098174077	0.11050221582875532	-0.073019066689534	0.38126204865301555	0.174898238773841	32
Women	1	CVLT_3	Model 1c APOE x age x education	age_c	-0.063671222209575	0.08857221057324562	-0.2457340084885567	0.11839156406940671	0.4786371924479791	32
Women	1	CVLT_3	Model 1c APOE x age x education	age_education	0.04086625511773667	0.11800260819631134	-0.2016915798664218	0.2834240901018952	0.7318918346894467	32
Women	1	CVLT_3	Model 1c APOE x age x education	education_c	-0.16584172719571266	0.37421287231209194	-0.9350473025523233	0.6033638481608979	0.6613049986768156	32
Women	1	CVLT_3	Model 2a PICALM	PICALM	0.46363636363636207	0.41773362015819177	-0.3894895028837163	1.3167622301564403	0.2758660541645678	32

Sex	Cluster	Outcome	Model	Predictor	Beta	SE	CI_low	CI_high	p	N
Women	1	CVLT_3	Model 2b PICALM x age	PICALM	0.4790590126689631	0.43460323648953303	-0.41118536080352375	1.3693033861414499	0.2797202914372464	32
Women	1	CVLT_3	Model 2b PICALM x age	PICALM_age	0.131589623145084	0.15821754954080125	-0.19250433529163613	0.4556835815818041	0.4126150550209722	32
Women	1	CVLT_3	Model 2b PICALM x age	age_c	-0.05572755417957054	0.08806751225465702	-0.23612567524215014	0.12467056688300907	0.5320101439101271	32
Women	1	CVLT_3	Model 2c PICALM x age x education	PICALM	0.4803751888626886	0.43379272589153534	-0.4112985294764998	1.372048907201877	0.27826803129541655	32
Women	1	CVLT_3	Model 2c PICALM x age x education	PICALM_age	0.11299504085549122	0.1713666881662042	-0.2392542314728744	0.46524431318385684	0.5154528482938476	32
Women	1	CVLT_3	Model 2c PICALM x age x education	PICALM_education	0.1256795552257035	0.11349228293673888	-0.10760717340954923	0.35896628386095625	0.2782680312954162	32
Women	1	CVLT_3	Model 2c PICALM x age x education	age_c	-0.049772874620169216	0.09327269370814802	-0.24149764235878715	0.14195189311844872	0.5981322073962061	32
Women	1	CVLT_3	Model 2c PICALM x age x education	age_education	0.04831251710208617	0.12144611117973522	-0.20132353963655464	0.297948573840727	0.6940175229677248	32
Women	1	CVLT_3	Model 2c PICALM x age x education	education_c	-0.12698234520274587	0.38241031085967064	-0.9130379968153705	0.6590733064098787	0.7425077045209065	32
Women	1	CVLT_3	Model 3a APOE x PICALM	APOE	0.470588235294118	0.5625483996313825	-0.6817399241162909	1.6229163947045269	0.40993997101424773	32
Women	1	CVLT_3	Model 3a APOE x PICALM	APOE_PICALM	0.12941176470587745	0.8975167587181482	-1.709067973733296	1.967891503145051	0.8863842794926347	32
Women	1	CVLT_3	Model 3a APOE x PICALM	PICALM	0.2705882352941229	0.5625483996313828	-0.8817399241162867	1.4229163947045325	0.6342508995038695	32
Women	1	CVLT_3	Model 3b APOE x PICALM x age	APOE	0.4743249999999999	0.5825109920137443	-0.7230434924173139	1.6716934924173137	0.4228822203008814	32
Women	1	CVLT_3	Model 3b APOE x PICALM x age	APOE_PICALM	0.16306193285454063	0.9342578602628339	-1.757332602199212	2.0834564679082934	0.8627959778840163	32
Women	1	CVLT_3	Model 3b APOE x PICALM x age	APOE_PICALM_age	0.01907107843137379	0.19769614997945034	-0.3872991777577426	0.42544133462049016	0.923889729922128	32
Women	1	CVLT_3	Model 3b APOE x PICALM x age	PICALM	0.22235000000000404	0.5988600497947709	-1.008624461980283	1.4533244619802912	0.7134309206068841	32
Women	1	CVLT_3	Model 3b APOE x PICALM x age	age_c	-0.02887500000000054	0.08344053641358716	-0.2003894789742814	0.1426394789742803	0.7320862083797601	32
Women	1	CVLT_3	Model 3c APOE x PICALM x age x education	APOE	0.43089466119434006	0.6404533753675294	-0.8939840878782623	1.7557734102669424	0.5077808547269909	32
Women	1	CVLT_3	Model 3c APOE x PICALM x age x education	APOE_PICALM	0.20763165674641804	0.9388315659887618	-1.7344894071378598	2.149752720630696	0.826919773941008	32
Women	1	CVLT_3	Model 3c APOE x PICALM x age x education	APOE_PICALM_age	-0.16853408029878403	0.30167371293708284	-0.7925937024294495	0.45552554183188143	0.5817902745259899	32
Women	1	CVLT_3	Model 3c APOE x PICALM x age x education	APOE_PICALM_education	0.05432223577667747	0.24562453761333877	-0.45379083326281366	0.5624353048161687	0.8269197739410131	32
Women	1	CVLT_3	Model 3c APOE x PICALM x age x education	APOE_age_education	0.7643623350474709	0.9942558769921298	-1.2924126515961558	2.8211373216910975	0.4498476309104279	32
Women	1	CVLT_3	Model 3c APOE x PICALM x age x education	PICALM	0.2684960648284207	0.6485575425864161	-1.07314743143764	1.6101395610944813	0.682716940621962	32

Sex	Cluster	Outcome	Model	Predictor	Beta	SE	CI_low	CI_high	p	N
Women	1	CVLT_3	Model 3c APOE x PICALM x age x education	age_c	-0.05399005155489811	0.09549208842722039	-0.25153048701467556	0.14355038390487934	0.5772844211581243	32
Women	1	CVLT_3	Model 3c APOE x PICALM x age x education	age_education	0.04870157981986889	0.12479157450497472	-0.20944946049602176	0.30685262013575954	0.6999327854552586	32
Women	1	CVLT_3	Model 3c APOE x PICALM x age x education	education_c	-0.2089890390995317	0.40481105909858106	-1.0464045172856036	0.6284264390865401	0.6105987882214223	32
Women	1	CVLT_4	Model 1a APOE	APOE	-0.49090909090909113	0.7108447618728676	-1.9426477687880606	0.9608295869698782	0.49512615881504873	32
Women	1	CVLT_4	Model 1b APOE x age	APOE	-0.4206079694861967	0.7151565306316936	-1.885539714333667	1.0443237753612737	0.5611565407087983	32
Women	1	CVLT_4	Model 1b APOE x age	APOE_age	-0.34323486414276333	0.2687225251857071	-0.8936880038944182	0.20721827560889144	0.21199233213980512	32
Women	1	CVLT_4	Model 1b APOE x age	age_c	0.1715481171548121	0.14128145986722426	-0.11785383424046825	0.46095006855009246	0.2348021408655513	32
Women	1	CVLT_4	Model 1c APOE x age x education	APOE	-0.42092688979423754	0.7122179156712778	-1.8849117821854147	1.0430580025969396	0.5596180064727837	32
Women	1	CVLT_4	Model 1c APOE x age x education	APOE_age	-0.2831430426664033	0.26989657635887127	-0.8379234007609866	0.2716373154281801	0.30379572180304815	32
Women	1	CVLT_4	Model 1c APOE x age x education	APOE_age_education	-0.07407812162783836	0.07061247637296049	-0.21922414554793282	0.0710679022922561	0.30379572180304626	32
Women	1	CVLT_4	Model 1c APOE x age x education	APOE_education	-0.11012622116709722	0.186336082588416	-0.4931455244089755	0.27289308207478113	0.5596180064727836	32
Women	1	CVLT_4	Model 1c APOE x age x education	age_c	0.16014735429361493	0.14935626965156368	-0.14685885482105698	0.46715356340828684	0.2934596551354966	32
Women	1	CVLT_4	Model 1c APOE x age x education	age_education	-0.11202992463456812	0.19898373604192038	-0.5210468518798779	0.2969870026107417	0.5782510428728631	32
Women	1	CVLT_4	Model 1c APOE x age x education	education_c	0.19260069934981966	0.6310222845562983	-1.10448418299533	1.4896855816949695	0.7626286515245853	32
Women	1	CVLT_4	Model 2a PICALM	PICALM	0.09090909090909038	0.7162805674033533	-1.3719309828826	1.5537491647007808	0.899851957098694	32
Women	1	CVLT_4	Model 2b PICALM x age	PICALM	0.09596146428320651	0.7323312481829014	-1.4041510946544742	1.5960740232208872	0.8966841149392666	32
Women	1	CVLT_4	Model 2b PICALM x age	PICALM_age	-0.3113056474858543	0.26660559749983725	-0.8574224574471092	0.23481116247540057	0.2527898455494041	32
Women	1	CVLT_4	Model 2b PICALM x age	age_c	0.17337461300309398	0.14839878251889002	-0.13060651294231956	0.4773557389485075	0.2525365173008368	32
Women	1	CVLT_4	Model 2c PICALM x age x education	PICALM	0.09022304092494382	0.7293127762605552	-1.408900840656989	1.5893469225068766	0.9024965711855903	32
Women	1	CVLT_4	Model 2c PICALM x age x education	PICALM_age	-0.26354728923075965	0.288109752989083	-0.8557653680599455	0.3286707895984262	0.36873096005905304	32
Women	1	CVLT_4	Model 2c PICALM x age x education	PICALM_education	0.02360486535827022	0.19080857518444758	-0.3686077780788633	0.41581750879540375	0.9024965711855901	32
Women	1	CVLT_4	Model 2c PICALM x age x education	age_c	0.15434482314733072	0.15681444878492193	-0.16799189273463105	0.4766815390292925	0.33406313151663025	32
Women	1	CVLT_4	Model 2c PICALM x age x education	age_education	-0.10980697254854502	0.20418092610590974	-0.5295068769686072	0.3098929318715171	0.595294155865764	32

Sex	Cluster	Outcome	Model	Predictor	Beta	SE	CI_low	CI_high	p	N
Women	1	CVLT_4	Model 2c PICALM x age x education	education_c	0.03502359130331634	0.642926238356203	-1.2865302185137821	1.3565774011204148	0.9569729372281974	32
Women	1	CVLT_4	Model 3a APOE x PICALM	APOE	-0.3999999999999996	0.9785944302025884	-2.404559819748029	1.60455981974803	0.6858330113487965	32
Women	1	CVLT_4	Model 3a APOE x PICALM	APOE_PICALM	-0.4000000000000039	1.561296595405091	-3.598171096488392	2.7981710964883844	0.7996714480625446	32
Women	1	CVLT_4	Model 3a APOE x PICALM	PICALM	0.4000000000000017	0.9785944302025887	-1.6045598197480286	2.404559819748032	0.6858330113487949	32
Women	1	CVLT_4	Model 3b APOE x PICALM x age	APOE	-0.41760000000000075	0.9959075125057095	-2.4647172101210795	1.6295172101210782	0.6784294920379816	32
Women	1	CVLT_4	Model 3b APOE x PICALM x age	APOE_PICALM	-0.5331743274053768	1.5972821704818783	-3.8164348506502583	2.7500861958395046	0.7412070874988728	32
Women	1	CVLT_4	Model 3b APOE x PICALM x age	APOE_PICALM_age	-0.24874509803921463	0.33799719431448005	-0.9435082811313231	0.44601808505289386	0.4683562164551256	32
Women	1	CVLT_4	Model 3b APOE x PICALM x age	PICALM	0.6272000000000024	1.0238591729717665	-1.4773726710680068	2.7317726710680117	0.5454724015746002	32
Women	1	CVLT_4	Model 3b APOE x PICALM x age	age_c	0.13599999999999957	0.14265663343883636	-0.15723490965121356	0.4292349096512127	0.34919856841307795	32
Women	1	CVLT_4	Model 3c APOE x PICALM x age x education	APOE	-0.1787706311958388	1.0875306240912759	-2.4284991332860253	2.0709578708943477	0.8708663994567818	32
Women	1	CVLT_4	Model 3c APOE x PICALM x age x education	APOE_PICALM	-0.750487778178606	1.5941957965174813	-4.048333045142538	2.547357488785326	0.6422443567542535	32
Women	1	CVLT_4	Model 3c APOE x PICALM x age x education	APOE_PICALM_age	0.15709617180205526	0.5122611792218709	-0.9025968151175	1.2167891587216104	0.7618521866135468	32
Women	1	CVLT_4	Model 3c APOE x PICALM x age x education	APOE_PICALM_education	-0.19634854661649315	0.417086109553992	-1.0591569013454283	0.6664598081124421	0.6422443567542577	32
Women	1	CVLT_4	Model 3c APOE x PICALM x age x education	APOE_age_education	-1.6431879072221518	1.6883098067695654	-5.135722837741113	1.8493470232968097	0.34054277220445095	32
Women	1	CVLT_4	Model 3c APOE x PICALM x age x education	PICALM	0.6688957440232981	1.101292016211722	-1.60930036660681	2.947091854653406	0.5495530941725811	32
Women	1	CVLT_4	Model 3c APOE x PICALM x age x education	age_c	0.18426574817850383	0.16215164837477633	-0.15117049327396948	0.5197019896309771	0.26750008569299083	32
Women	1	CVLT_4	Model 3c APOE x PICALM x age x education	age_education	-0.09251002832008792	0.21190404192162643	-0.5308669373198158	0.34584688067964003	0.666497301970739	32
Women	1	CVLT_4	Model 3c APOE x PICALM x age x education	education_c	0.08510916752064246	0.6873949621826762	-1.3368756523622594	1.507093987403544	0.9025381715825096	32
Women	1	CVLT_5	Model 1a APOE	APOE	1.0727272727272725	0.6148215266578371	-0.18290579670710505	2.32836034216165	0.09126147440277324	32
Women	1	CVLT_5	Model 1b APOE x age	APOE	1.151013133491443	0.5981205393292763	-0.07418125092506966	2.3762075179079556	0.06452383437436883	32
Women	1	CVLT_5	Model 1b APOE x age	APOE_age	-0.05726042244290963	0.22474584906891595	-0.5176314247645131	0.4031105798786938	0.8007561491940227	32
Women	1	CVLT_5	Model 1b APOE x age	age_c	-0.1867154811715478	0.11816062547645392	-0.42875655027650905	0.055325587933413495	0.12529678024303556	32
Women	1	CVLT_5	Model 1c APOE x age x education	APOE	0.919588907494561	0.577094658759111	-0.26664615246835355	2.1058239674574755	0.12313844146794443	32

Sex	Cluster	Outcome	Model	Predictor	Beta	SE	CI_low	CI_high	p	N
Women	1	CVLT_5	Model 1c APOE x age x education	APOE_age	-0.12107661929370883	0.21869131512547324	-0.570603055509644	0.32844981692222636	0.5845544081176748	32
Women	1	CVLT_5	Model 1c APOE x age x education	APOE_age_education	-0.03167702248963333	0.05721575105026916	-0.14928568312752333	0.08593163814825668	0.5845544081176723	32
Women	1	CVLT_5	Model 1c APOE x age x education	APOE_education	0.24059012114683237	0.15098406769860484	-0.06976207477369806	0.5509423170673629	0.12313844146794581	32
Women	1	CVLT_5	Model 1c APOE x age x education	age_c	-0.1495888733865336	0.12102013101827801	-0.39834931536302126	0.09917156858995407	0.22748513964233377	32
Women	1	CVLT_5	Model 1c APOE x age x education	age_education	0.13369056291884707	0.16123218571593145	-0.1977269412769846	0.4651080671146787	0.4145503624993262	32
Women	1	CVLT_5	Model 1c APOE x age x education	education_c	0.4126346936502744	0.511303608014669	-0.6383649247081927	1.4636343120087414	0.42697348315526873	32
Women	1	CVLT_5	Model 2a PICALM	PICALM	0.7818181818181822	0.6292765534455053	-0.5033359906797468	2.0669723543161114	0.22371045253692126	32
Women	1	CVLT_5	Model 2b PICALM x age	PICALM	0.6744429580613489	0.614728677154339	-0.584771654487942	1.9336575706106398	0.28192674177046	32
Women	1	CVLT_5	Model 2b PICALM x age	PICALM_age	0.2938400768655922	0.22379231622256976	-0.16457770196361932	0.7522578556948037	0.19984146431669822	32
Women	1	CVLT_5	Model 2b PICALM x age	age_c	-0.26625386996904365	0.12456792946566722	-0.5214197063251627	-0.011088033612924708	0.04143666139310031	32
Women	1	CVLT_5	Model 2c PICALM x age x education	PICALM	0.4652354296599932	0.5946730354655781	-0.7571325011066188	1.6876033604266052	0.4410831967863186	32
Women	1	CVLT_5	Model 2c PICALM x age x education	PICALM_age	0.2512513167904861	0.23492129431179185	-0.231636319431488	0.7341389530124602	0.2946678103213911	32
Women	1	CVLT_5	Model 2c PICALM x age x education	PICALM_education	0.12171857171337053	0.15558306160436636	-0.19808699156859183	0.4415241349953329	0.4410831967863177	32
Women	1	CVLT_5	Model 2c PICALM x age x education	age_c	-0.24179409393119788	0.12786465190138827	-0.5046236500763246	0.021035462213928824	0.06981467783440874	32
Women	1	CVLT_5	Model 2c PICALM x age x education	age_education	0.06929300565354521	0.1664867188178742	-0.27292534601965324	0.4115113573267436	0.6806739500786765	32
Women	1	CVLT_5	Model 2c PICALM x age x education	education_c	0.5524300207268943	0.5242344713938715	-0.5251493679745926	1.6300094094283812	0.3016766930482383	32
Women	1	CVLT_5	Model 3a APOE x PICALM	APOE	0.7529411764705878	0.8375162772043914	-0.962633147124653	2.4685155000658288	0.37631038242783665	32
Women	1	CVLT_5	Model 3a APOE x PICALM	APOE_PICALM	0.4470588235294075	1.3362137284235938	-2.2900509207383326	3.1841685677971476	0.7404418134107678	32
Women	1	CVLT_5	Model 3a APOE x PICALM	PICALM	0.3529411764705923	0.8375162772043918	-1.3626331471246493	2.0685155000658337	0.676670348299129	32
Women	1	CVLT_5	Model 3b APOE x PICALM x age	APOE	0.7758874999999994	0.8126430201677731	-0.8945241510625104	2.446299151062509	0.34848724953406685	32
Women	1	CVLT_5	Model 3b APOE x PICALM x age	APOE_PICALM	0.6917382253192013	1.3033541677125242	-1.9873446353917699	3.3708210860301726	0.6001056515181609	32
Women	1	CVLT_5	Model 3b APOE x PICALM x age	APOE_PICALM_age	-0.12170710784313593	0.2757997678969994	-0.6886216499262892	0.44520743424001735	0.6626526572969191	32
Women	1	CVLT_5	Model 3b APOE x PICALM x age	PICALM	0.05672500000000413	0.835451083662234	-1.6605692970138057	1.774019297013814	0.9463867337971006	32

Sex	Cluster	Outcome	Model	Predictor	Beta	SE	CI_low	CI_high	p	N
Women	1	CVLT_5	Model 3b APOE x PICALM x age	age_c	-0.1773125000000001	0.1164053046984504	-0.4165870306218582	0.06196203062185798	0.13977183710527943	32
Women	1	CVLT_5	Model 3c APOE x PICALM x age x education	APOE	0.5468212009291562	0.8778535866531361	-1.2691573019345044	2.362799703792817	0.5394735098652916	32
Women	1	CVLT_5	Model 3c APOE x PICALM x age x education	APOE_PICALM	0.7925669656632011	1.2868331859340516	-1.869450297759055	3.454584229085457	0.5440048975176077	32
Women	1	CVLT_5	Model 3c APOE x PICALM x age x education	APOE_PICALM_age	-0.17203548085900788	0.4134966901358191	-1.0274185557915538	0.6833475940735381	0.6812296792997429	32
Women	1	CVLT_5	Model 3c APOE x PICALM x age x education	APOE_PICALM_education	0.2073576363653711	0.3366714730641413	-0.4891003685997539	0.9038156413304961	0.54400489751761	32
Women	1	CVLT_5	Model 3c APOE x PICALM x age x education	APOE_age_education	-0.03556586129688366	1.3628019169508327	-2.854736418290881	2.7836046956971137	0.9794046058032617	32
Women	1	CVLT_5	Model 3c APOE x PICALM x age x education	PICALM	-0.07020550867140729	0.8889617680345735	-1.9091630354876936	1.768752018144879	0.9377358268946011	32
Women	1	CVLT_5	Model 3c APOE x PICALM x age x education	age_c	-0.1521202755163169	0.13088864162005298	-0.4228840601210499	0.11864350908841609	0.2570679855355782	32
Women	1	CVLT_5	Model 3c APOE x PICALM x age x education	age_education	0.13164180679770904	0.17104872185335687	-0.2221994334166873	0.4854830470121054	0.44936025408025193	32
Women	1	CVLT_5	Model 3c APOE x PICALM x age x education	education_c	0.42391671739966774	0.5548644972674474	-0.7239079476239721	1.5717413824233075	0.4526339931281933	32
Women	1	CVLT_6	Model 1a APOE	APOE	0.8818181818181808	0.4141305738911401	0.03605071744808108	1.7275856461882806	0.04154920007565386	32
Women	1	CVLT_6	Model 1b APOE x age	APOE	0.9120751640404167	0.408708283561694	0.07487419708176701	1.7492761309990663	0.033824183731163054	32
Women	1	CVLT_6	Model 1b APOE x age	APOE_age	0.0278204869687958	0.15357354274036064	-0.2867606547713561	0.3424016287089477	0.8575515225781545	32
Women	1	CVLT_6	Model 1b APOE x age	age_c	-0.13023012552301227	0.08074162856405663	-0.29562185411380476	0.03516160306778021	0.11797667575919828	32
Women	1	CVLT_6	Model 1c APOE x age x education	APOE	0.6946670950096508	0.3798731634667464	-0.08617337544664183	1.4755075654659435	0.07894185737732674	32
Women	1	CVLT_6	Model 1c APOE x age x education	APOE_age	-0.03306515834619317	0.14395378719679594	-0.3289664057333384	0.26283608904095207	0.8201278808485877	32
Women	1	CVLT_6	Model 1c APOE x age x education	APOE_age_education	-0.00865076817196958	0.037662328045673356	-0.08606679219767618	0.06876525585373702	0.8201278808485788	32
Women	1	CVLT_6	Model 1c APOE x age x education	APOE_education	0.1817442981129897	0.0993854206744396	-0.02254535985522671	0.3860339560812061	0.07894185737732766	32
Women	1	CVLT_6	Model 1c APOE x age x education	age_c	-0.09599816889556934	0.07966162797611853	-0.2597449903306975	0.0677486525395588	0.2390289338926852	32
Women	1	CVLT_6	Model 1c APOE x age x education	age_education	0.11052708937769407	0.10613125509126344	-0.10762882982251398	0.3286830085779021	0.3072688031366072	32
Women	1	CVLT_6	Model 1c APOE x age x education	education_c	0.4377780615528064	0.3365661354172551	-0.25404353784762446	1.1295996609532373	0.20476553179204127	32
Women	1	CVLT_6	Model 2a PICALM	PICALM	0.5909090909090904	0.4310279851319892	-0.28936749102099035	1.4711856728391712	0.18056665410854625	32
Women	1	CVLT_6	Model 2b PICALM x age	PICALM	0.5448382732687334	0.4227453290671888	-0.32111627795307685	1.4107928244905437	0.20801138208276954	32

Sex	Cluster	Outcome	Model	Predictor	Beta	SE	CI_low	CI_high	p	N
Women	1	CVLT_6	Model 2b PICALM x age	PICALM_age	0.23544357852033782	0.15390067176004832	-0.07980765664003081	0.5506948136807064	0.1372764666298073	32
Women	1	CVLT_6	Model 2b PICALM x age	age_c	-0.1733746130030993	0.08566463919814898	-0.3488506717359005	0.0021014457297018896	0.05261990211412131	32
Women	1	CVLT_6	Model 2c PICALM x age x education	PICALM	0.3458653429374676	0.39206486792175843	-0.4600355349333358	1.151766220808271	0.38577173127789	32
Women	1	CVLT_6	Model 2c PICALM x age x education	PICALM_age	0.19144420030856324	0.15488239878616888	-0.12692112992403176	0.5098095305411583	0.22748672603723502	32
Women	1	CVLT_6	Model 2c PICALM x age x education	PICALM_education	0.09048802576852377	0.1025751107934833	-0.12035813413953525	0.30133418567658277	0.38577173127788855	32
Women	1	CVLT_6	Model 2c PICALM x age x education	age_c	-0.1484406539761356	0.0843005061097666	-0.321722825977254	0.024841518024982828	0.09002282651977758	32
Women	1	CVLT_6	Model 2c PICALM x age x education	age_education	0.072872268884181	0.10976383580761005	-0.15275052691672394	0.2984950646850859	0.5125987594908752	32
Women	1	CVLT_6	Model 2c PICALM x age x education	education_c	0.5445464050112153	0.3456250856004521	-0.1658961331739761	1.2549889431964067	0.12722278310973398	32
Women	1	CVLT_6	Model 3a APOE x PICALM	APOE	0.894117647058823	0.5633211313138146	-0.25979338144857134	2.0480286755662176	0.12369161133888429	32
Women	1	CVLT_6	Model 3a APOE x PICALM	APOE_PICALM	-0.2941176470588278	0.8987496119898483	-2.1351227709445375	1.5468874768268819	0.7459113762456636	32
Women	1	CVLT_6	Model 3a APOE x PICALM	PICALM	0.4941176470588271	0.5633211313138148	-0.6597933814485677	1.648028675566222	0.38786888400231645	32
Women	1	CVLT_6	Model 3b APOE x PICALM x age	APOE	0.9073500000000001	0.5576508699216377	-0.2389177796087325	2.0536177796087327	0.11577747952903049	32
Women	1	CVLT_6	Model 3b APOE x PICALM x age	APOE_PICALM	-0.15393827519379366	0.8943860556272634	-1.9923751420473137	1.6844985916597264	0.8646789463870383	32
Women	1	CVLT_6	Model 3b APOE x PICALM x age	APOE_PICALM_age	-0.06441666666666562	0.18925896940601905	-0.4534440498079482	0.324610716474617	0.7363182290610422	32
Women	1	CVLT_6	Model 3b APOE x PICALM x age	PICALM	0.32330000000000236	0.5733021905301481	-0.8551395298731612	1.501739529873166	0.5776368962332676	32
Women	1	CVLT_6	Model 3b APOE x PICALM x age	age_c	-0.10225000000000062	0.07987950159860172	-0.2664446670800467	0.06194466708004548	0.21183013269871764	32
Women	1	CVLT_6	Model 3c APOE x PICALM x age x education	APOE	0.6502304747911906	0.5788852021269809	-0.547284804147753	1.847745753730134	0.2729213064711943	32
Women	1	CVLT_6	Model 3c APOE x PICALM x age x education	APOE_PICALM	0.04653579277876613	0.8485796495782638	-1.7088849573680318	1.8019565429255642	0.9567398154412035	32
Women	1	CVLT_6	Model 3c APOE x PICALM x age x education	APOE_PICALM_age	-0.17460317460317232	0.27267316405314374	-0.7386705905787477	0.3894642413724031	0.5282808405624316	32
Women	1	CVLT_6	Model 3c APOE x PICALM x age x education	APOE_PICALM_education	0.012175062064210568	0.2220121176222201	-0.4470919946602422	0.4714421187886634	0.9567398154412087	32
Women	1	CVLT_6	Model 3c APOE x PICALM x age x education	APOE_age_education	0.25646251275723664	0.8986759012523555	-1.602590229668639	2.1155152551831122	0.777908596435974	32
Women	1	CVLT_6	Model 3c APOE x PICALM x age x education	PICALM	0.18126816615885133	0.5862102981589657	-1.031400228433708	1.3939365607514107	0.7599377703654536	32
Women	1	CVLT_6	Model 3c APOE x PICALM x age x education	age_c	-0.08946217719689864	0.08631222667691729	-0.26801262178431695	0.08908826739051964	0.3107465231493852	32

Sex	Cluster	Outcome	Model	Predictor	Beta	SE	CI_low	CI_high	p	N
Women	1	CVLT_6	Model 3c APOE x PICALM x age x education	age_education	0.11581690597490302	0.11279508955605233	-0.11751751445312189	0.3491513264029279	0.31519747910774554	32
Women	1	CVLT_6	Model 3c APOE x PICALM x age x education	education_c	0.4086482711088114	0.36589569324237203	-0.34826463933657226	1.165561181554195	0.2755930027146829	32
Women	1	CVLT_7	Model 1a APOE	APOE	0.7454545454545449	0.4776434346756012	-0.2300234851165548	1.7209325760256446	0.12908471571654032	32
Women	1	CVLT_7	Model 1b APOE x age	APOE	0.7812553854612754	0.43692187166554736	-0.11373849686497695	1.6762492677875276	0.08458941726461112	32
Women	1	CVLT_7	Model 1b APOE x age	APOE_age	0.1028255280536371	0.1641748954723564	-0.2334715003354254	0.4391225564426996	0.5361801196407403	32
Women	1	CVLT_7	Model 1b APOE x age	age_c	-0.2353556485355648	0.08631531312774801	-0.41216455239273664	-0.058546744678392965	0.0109131508572903	32
Women	1	CVLT_7	Model 1c APOE x age x education	APOE	0.6581615010679833	0.432883403764392	-0.23164307886965252	1.5479660810056193	0.14047634265810852	32
Women	1	CVLT_7	Model 1c APOE x age x education	APOE_age	0.07351235089214447	0.164042136638007	-0.26368109014515523	0.4107057919294442	0.6577708330041683	32
Women	1	CVLT_7	Model 1c APOE x age x education	APOE_age_education	0.019232882500851423	0.04291800086459486	-0.06898633172402349	0.10745209672572634	0.6577708330041739	32
Women	1	CVLT_7	Model 1c APOE x age x education	APOE_education	0.1721934159770882	0.11325437889184692	-0.06060429389031691	0.4049911258444933	0.14047634265811007	32
Women	1	CVLT_7	Model 1c APOE x age x education	age_c	-0.22121350807468487	0.09077818594240762	-0.40781074166590014	-0.03461627448346957	0.02196775592411097	32
Women	1	CVLT_7	Model 1c APOE x age x education	age_education	0.03875419111037459	0.12094157568389224	-0.2098447780637207	0.2873531602844699	0.7511980552107631	32
Women	1	CVLT_7	Model 1c APOE x age x education	education_c	0.21193366817630954	0.38353300075645524	-0.5764297055696227	1.000297041922242	0.5852690501967657	32
Women	1	CVLT_7	Model 2a PICALM	PICALM	0.8909090909090902	0.4692647082310631	-0.06745729742544648	1.849275479243627	0.06728422801904689	32
Women	1	CVLT_7	Model 2b PICALM x age	PICALM	0.7876107724680494	0.4179146911376774	-0.06844866551952244	1.6436702104556213	0.06990066970379313	32
Women	1	CVLT_7	Model 2b PICALM x age	PICALM_age	0.33000960819899694	0.15214207533978316	0.018360694505435038	0.6416585218925588	0.03872118629941432	32
Women	1	CVLT_7	Model 2b PICALM x age	age_c	-0.2817337461300349	0.08468576414767451	-0.45520467021851896	-0.10826282204155077	0.0024653475950795053	32
Women	1	CVLT_7	Model 2c PICALM x age x education	PICALM	0.659994776837534	0.41367970003843696	-0.19033602476042544	1.5103255784354936	0.12270232747320992	32
Women	1	CVLT_7	Model 2c PICALM x age x education	PICALM_age	0.3234350563811047	0.1634211823434336	-0.012481994823647646	0.659352107585857	0.05848053423532369	32
Women	1	CVLT_7	Model 2c PICALM x age x education	PICALM_education	0.17267305207958772	0.1082301540798236	-0.04979721578034352	0.39514331993951896	0.12270232747320911	32
Women	1	CVLT_7	Model 2c PICALM x age x education	age_c	-0.2756275635598924	0.0889480566454021	-0.45846291250459004	-0.09279221461519471	0.004624824294960611	32
Women	1	CVLT_7	Model 2c PICALM x age x education	age_education	0.0017902113469742409	0.11581519893035132	-0.23627133949664322	0.2398517621905917	0.9877852109989342	32
Women	1	CVLT_7	Model 2c PICALM x age x education	education_c	0.2668700023760957	0.36467965746292574	-0.48273976921314643	1.016479773965338	0.47084008059935445	32

Sex	Cluster	Outcome	Model	Predictor	Beta	SE	CI_low	CI_high	p	N
Women	1	CVLT_7	Model 3a APOE x PICALM	APOE	0.5058823529411762	0.6343285535047265	-0.7934807863025521	1.8052454921849046	0.4318674014874847	32
Women	1	CVLT_7	Model 3a APOE x PICALM	APOE_PICALM	0.09411764705881676	1.0120382667108971	-1.9789487662418648	2.1671840603594985	0.9265673059082327	32
Women	1	CVLT_7	Model 3a APOE x PICALM	PICALM	0.7058823529411811	0.6343285535047267	-0.5934807863025477	2.00524549218491	0.2752561274701356	32
Women	1	CVLT_7	Model 3b APOE x PICALM x age	APOE	0.5332124999999988	0.5871173156239337	-0.6736244262019748	1.7400494262019723	0.37212084201499807	32
Women	1	CVLT_7	Model 3b APOE x PICALM x age	APOE_PICALM	0.34676789643183303	0.9416456934517946	-1.5888125472296113	2.2823483400932774	0.7156627658689607	32
Women	1	CVLT_7	Model 3b APOE x PICALM x age	APOE_PICALM_age	0.09844240196078546	0.19925947231289806	-0.31114130930682055	0.5080261132283914	0.625425218296858	32
Women	1	CVLT_7	Model 3b APOE x PICALM x age	PICALM	0.35307500000000314	0.6035956568895529	-0.8876336417734543	1.5937836417734605	0.5636219158714146	32
Women	1	CVLT_7	Model 3b APOE x PICALM x age	age_c	-0.21118750000000042	0.0841003593494601	-0.38405826444325986	-0.03831673555674098	0.01858273374381127	32
Women	1	CVLT_7	Model 3c APOE x PICALM x age x education	APOE	0.40913019201862866	0.6515480568253222	-0.9386996542868117	1.756960038324069	0.5362318068844764	32
Women	1	CVLT_7	Model 3c APOE x PICALM x age x education	APOE_PICALM	0.42597447014143597	0.9550951029889202	-1.5497902833305512	2.401739223613423	0.6597661108177922	32
Women	1	CVLT_7	Model 3c APOE x PICALM x age x education	APOE_PICALM_age	0.022175536881421376	0.3068996573663801	-0.6126947749645367	0.6570458487273795	0.9430223514889716	32
Women	1	CVLT_7	Model 3c APOE x PICALM x age x education	APOE_PICALM_education	0.11144680904863065	0.24987953275872904	-0.40546838808066815	0.6283620061779295	0.6597661108177947	32
Women	1	CVLT_7	Model 3c APOE x PICALM x age x education	APOE_age_education	0.24308597403993648	1.0114795386465567	-1.849318871364403	2.335490819444276	0.8122058213848082	32
Women	1	CVLT_7	Model 3c APOE x PICALM x age x education	PICALM	0.2873670659034593	0.6597926138949496	-1.0775179461285997	1.6522520779355183	0.6672295897401566	32
Women	1	CVLT_7	Model 3c APOE x PICALM x age x education	age_c	-0.2108519051687996	0.09714631392369999	-0.41181436679121874	-0.009889443546380411	0.04054510448076391	32
Women	1	CVLT_7	Model 3c APOE x PICALM x age x education	age_education	0.04714021446416772	0.126953359922929	-0.215482819708667	0.30976324863700244	0.7137984012091554	32
Women	1	CVLT_7	Model 3c APOE x PICALM x age x education	education_c	0.165753786801341	0.41182366910897117	-0.686168380451625	1.017675954054307	0.6910418023872509	32
Women	1	CVLT_8	Model 1a APOE	APOE	0.7272727272727271	0.41989110486518244	-0.1302593108393295	1.5848047653847837	0.0935364703433236	32
Women	1	CVLT_8	Model 1b APOE x age	APOE	0.7207599494015753	0.419583213779414	-0.13871730228157697	1.5802372010847274	0.09687511561563411	32
Women	1	CVLT_8	Model 1b APOE x age	APOE_age	0.12970711297071136	0.1576598351590886	-0.19324441934342668	0.4526586452848494	0.4176289796258501	32
Women	1	CVLT_8	Model 1b APOE x age	age_c	-0.1297071129707108	0.08289000580918443	-0.29949959285369343	0.04008536691227185	0.1288606102922525	32
Women	1	CVLT_8	Model 1c APOE x age x education	APOE	0.6743020022335191	0.4198255257258126	-0.18866172498960865	1.5372657294566467	0.12032003524828158	32
Women	1	CVLT_8	Model 1c APOE x age x education	APOE_age	0.10884422961401619	0.15909382447177547	-0.2181778100939999	0.43586626932203226	0.49993278340934644	32

Sex	Cluster	Outcome	Model	Predictor	Beta	SE	CI_low	CI_high	p	N
Women	1	CVLT_8	Model 1c APOE x age x education	APOE_age_education	0.028476687980411086	0.04162338430947614	-0.057081403803662875	0.11403477976448505	0.49993278340934844	32
Women	1	CVLT_8	Model 1c APOE x age x education	APOE_education	0.1764162215145833	0.1098380735910558	-0.04935917223565395	0.40219161526482056	0.12032003524828241	32
Women	1	CVLT_8	Model 1c APOE x age x education	age_c	-0.1251892260139602	0.08803987241435053	-0.30615777553602014	0.05577932350809972	0.16692374153752462	32
Women	1	CVLT_8	Model 1c APOE x age x education	age_education	0.03399752048159099	0.11729338697686242	-0.20710248940748005	0.27509753037066204	0.7742298237327656	32
Women	1	CVLT_8	Model 1c APOE x age x education	education_c	-0.029546455005007632	0.37196377194311414	-0.7941289383427219	0.7350360283327066	0.9372959080713803	32
Women	1	CVLT_8	Model 2a PICALM	PICALM	0.5818181818181811	0.427382319923181	-0.2910129584710549	1.454649322107417	0.18354019543070127	32
Women	1	CVLT_8	Model 2b PICALM x age	PICALM	0.5634610597096392	0.42583024009680337	-0.30881264529703634	1.4357347647163148	0.19647118515385004	32
Women	1	CVLT_8	Model 2b PICALM x age	PICALM_age	0.235166008327106	0.1550237353331581	-0.08238571827711078	0.5527177349313228	0.1404850971904362	32
Women	1	CVLT_8	Model 2b PICALM x age	age_c	-0.14551083591331626	0.08628976210818431	-0.32226740087953365	0.031245729052901128	0.10285022528248516	32
Women	1	CVLT_8	Model 2c PICALM x age x education	PICALM	0.5133130232035796	0.4260723402460837	-0.3624912151637071	1.3891172615708665	0.2391498333574892	32
Women	1	CVLT_8	Model 2c PICALM x age x education	PICALM_age	0.2248416373029274	0.16831680549076775	-0.1211385114016717	0.5708217860075264	0.19318286378530083	32
Women	1	CVLT_8	Model 2c PICALM x age x education	PICALM_education	0.13429701188465737	0.11147241459926607	-0.0948378179207374	0.36343184169005216	0.2391498333574897	32
Women	1	CVLT_8	Model 2c PICALM x age x education	age_c	-0.1407656405398432	0.0916126816271777	-0.3290782045775238	0.04754692349783743	0.13648991167969135	32
Women	1	CVLT_8	Model 2c PICALM x age x education	age_education	0.02132484915382607	0.1192846853247466	-0.22386833311044183	0.26651803141809394	0.8595019309701881	32
Women	1	CVLT_8	Model 2c PICALM x age x education	education_c	0.041627644884112414	0.3756043989611574	-0.7304382544643083	0.8136935442325332	0.9126035075107043	32
Women	1	CVLT_8	Model 3a APOE x PICALM	APOE	0.6235294117647057	0.5709909347381433	-0.5460924968532492	1.7931513203826606	0.2841348996096389	32
Women	1	CVLT_8	Model 3a APOE x PICALM	APOE_PICALM	-0.023529411764711572	0.9109863850638089	-1.88960042900765	1.8425416054782269	0.9795773342339406	32
Women	1	CVLT_8	Model 3a APOE x PICALM	PICALM	0.4235294117647106	0.5709909347381437	-0.746092496853245	1.5931513203826662	0.4644188100632839	32
Women	1	CVLT_8	Model 3b APOE x PICALM x age	APOE	0.6360499999999989	0.5773499575069336	-0.5507098340547136	1.8228098340547114	0.2806988726489474	32
Women	1	CVLT_8	Model 3b APOE x PICALM x age	APOE_PICALM	0.08867286251710427	0.9259803562823393	-1.8147070194262582	1.9920527444604668	0.9244446265080997	32
Women	1	CVLT_8	Model 3b APOE x PICALM x age	APOE_PICALM_age	0.06733823529411898	0.19594456646275082	-0.33543158941217993	0.4701080600004179	0.7338659238621033	32
Women	1	CVLT_8	Model 3b APOE x PICALM x age	PICALM	0.26190000000000246	0.5935541629975853	-0.9581680554705632	1.481968055470568	0.662686708658722	32
Women	1	CVLT_8	Model 3b APOE x PICALM x age	age_c	-0.09675000000000078	0.08270125510627896	-0.2667448644836712	0.07324486448366965	0.2526645853380569	32

Sex	Cluster	Outcome	Model	Predictor	Beta	SE	CI_low	CI_high	p	N
Women	1	CVLT_8	Model 3c APOE x PICALM x age x education	APOE	0.5559505302766152	0.640023336833646	-0.7680386163100956	1.879939676863326	0.39401755631418967	32
Women	1	CVLT_8	Model 3c APOE x PICALM x age x education	APOE_PICALM	0.16739608558852603	0.9382011785698968	-1.7734209225642044	2.108213093741256	0.8599535380991786	32
Women	1	CVLT_8	Model 3c APOE x PICALM x age x education	APOE_PICALM_age	-0.09290382819794352	0.30147115124217694	-0.7165444195368624	0.5307367631409754	0.7607279967341926	32
Women	1	CVLT_8	Model 3c APOE x PICALM x age x education	APOE_PICALM_education	0.043795487508624234	0.24545961067235664	-0.4639764041592411	0.5515673791764896	0.8599535380991842	32
Women	1	CVLT_8	Model 3c APOE x PICALM x age x education	APOE_age_education	0.643540048362869	0.993588274390455	-1.4118538970780676	2.6989339938038057	0.5235968730687997	32
Women	1	CVLT_8	Model 3c APOE x PICALM x age x education	PICALM	0.2613424139637459	0.6481220624320756	-1.0794002229668518	1.6020850508943436	0.690502859352759	32
Women	1	CVLT_8	Model 3c APOE x PICALM x age x education	age_c	-0.11576599476286153	0.09542796935269579	-0.3131737898111506	0.08164180028542758	0.23739481203314308	32
Women	1	CVLT_8	Model 3c APOE x PICALM x age x education	age_education	0.041624085411522405	0.12470778201077429	-0.21635361692354454	0.29960178774658935	0.7415749041209319	32
Women	1	CVLT_8	Model 3c APOE x PICALM x age x education	education_c	-0.07154417536711699	0.4045392448478486	-0.9083973629347907	0.7653090122005567	0.861171599259979	32
Women	1	CVLT_9	Model 1a APOE	APOE	-1.3909090909090909	1.4454412044362421	-4.342893849932114	1.5610756681139322	0.3436050853629473	32
Women	1	CVLT_9	Model 1b APOE x age	APOE	-1.4694354190138001	1.41628500334403	-4.3705637346812125	1.431692896653612	0.30836728098771204	32
Women	1	CVLT_9	Model 1b APOE x age	APOE_age	-0.21607349901698839	0.5321739593779343	-1.306182438084201	0.8740354400502244	0.6878139245677715	32
Women	1	CVLT_9	Model 1b APOE x age	age_c	0.5052301255230134	0.2797916320274088	-0.06789705173647775	1.0783573027825044	0.0817220619150199	32
Women	1	CVLT_9	Model 1c APOE x age x education	APOE	-0.4285180123546668	1.1482506591205905	-2.788781045118121	1.9317450204087872	0.712030984331789	32
Women	1	CVLT_9	Model 1c APOE x age x education	APOE_age	0.06811570873964146	0.4351321623331676	-0.8263112606365131	0.962542678115796	0.8768163764147007	32
Women	1	CVLT_9	Model 1c APOE x age x education	APOE_age_education	0.01782097030979074	0.1138427168894915	-0.21618608563164501	0.25182802625122647	0.876816376414695	32
Women	1	CVLT_9	Model 1c APOE x age x education	APOE_education	-0.11211227067418408	0.30041441663038754	-0.7296229478506701	0.505398406502302	0.7120309843317945	32
Women	1	CVLT_9	Model 1c APOE x age x education	age_c	0.3305939836654558	0.24079489057721945	-0.1643670025908076	0.8255549699217192	0.18150124245018645	32
Women	1	CVLT_9	Model 1c APOE x age x education	age_education	-0.43655713330180584	0.32080519323789164	-1.0959816520718069	0.22286738546819523	0.1852516763099865	32
Women	1	CVLT_9	Model 1c APOE x age x education	education_c	-2.8060619994532887	1.0173455879421804	-4.89724580474188	-0.7148781941646973	0.01049520952365892	32
Women	1	CVLT_9	Model 2a PICALM	PICALM	-2.263636363636364	1.4081854373253966	-5.139534695650534	0.6122619683778057	0.11842352420732251	32
Women	1	CVLT_9	Model 2b PICALM x age	PICALM	-1.9658051600790936	1.4105642445763928	-4.85521503263039	0.9236047124722031	0.17439330655175692	32
Women	1	CVLT_9	Model 2b PICALM x age	PICALM_age	-0.3481370769723501	0.5135166963997593	-1.400028345308717	0.703754191364017	0.5033696858392931	32

Sex	Cluster	Outcome	Model	Predictor	Beta	SE	CI_low	CI_high	p	N
Women	1	CVLT_9	Model 2b PICALM x age	age_c	0.4860681114551081	0.2858351559887758	-0.09943866344845786	1.071574886358674	0.10011631435015422	32
Women	1	CVLT_9	Model 2c PICALM x age x education	PICALM	-1.0227471565479596	1.146465659118026	-3.3793410692581647	1.3338467561622454	0.3805285411492325	32
Women	1	CVLT_9	Model 2c PICALM x age x education	PICALM_age	-0.1006865142262936	0.45290299115911065	-1.0316419454032575	0.8302689169506702	0.8258068347933685	32
Women	1	CVLT_9	Model 2c PICALM x age x education	PICALM_education	-0.26757919793405915	0.2999474108157626	-0.8841299309105662	0.34897153504244793	0.3805285411492325	32
Women	1	CVLT_9	Model 2c PICALM x age x education	age_c	0.35061543503440823	0.24650929784508008	-0.15609168358533748	0.857322553654154	0.16682003341418522	32
Women	1	CVLT_9	Model 2c PICALM x age x education	age_education	-0.4280808099546874	0.32096848930521243	-1.087840988598281	0.2316793686889062	0.19386372962469775	32
Women	1	CVLT_9	Model 2c PICALM x age x education	education_c	-2.690418456779142	1.0106676828022312	-4.767875631464103	-0.6129612820941803	0.013143292674832512	32
Women	1	CVLT_9	Model 3a APOE x PICALM	APOE	-1.6352941176470575	1.9186933269923518	-5.565559231573069	2.2949709962789537	0.4012815311057495	32
Women	1	CVLT_9	Model 3a APOE x PICALM	APOE_PICALM	2.0352941176470587	3.061175566306327	-4.235239774663922	8.30582800995804	0.5115709233922896	32
Women	1	CVLT_9	Model 3a APOE x PICALM	PICALM	-2.8352941176470576	1.9186933269923525	-6.76555923157307	1.0949709962789549	0.15064356766702094	32
Women	1	CVLT_9	Model 3b APOE x PICALM x age	APOE	-1.6880374999999992	1.9036673147757763	-5.601081706903832	2.2250067069038337	0.38335361536504886	32
Women	1	CVLT_9	Model 3b APOE x PICALM x age	APOE_PICALM	1.5386456039101688	3.053188998213354	-4.737274263657916	7.814565471478254	0.6185423112233668	32
Women	1	CVLT_9	Model 3b APOE x PICALM x age	APOE_PICALM_age	-0.13305269607843045	0.6460782785778049	-1.461085617362818	1.1949802252059571	0.838442641839289	32
Women	1	CVLT_9	Model 3b APOE x PICALM x age	PICALM	-2.154424999999998	1.9570966360277715	-6.177294749624014	1.8684447496240182	0.2810594804519576	32
Women	1	CVLT_9	Model 3b APOE x PICALM x age	age_c	0.4075624999999994	0.2726867373760321	-0.15295311620391183	0.9680781162039106	0.14705316824846615	32
Women	1	CVLT_9	Model 3c APOE x PICALM x age x education	APOE	-0.47064754629285543	1.7459822034062489	-4.082486919025397	3.1411918264396865	0.7899021771418936	32
Women	1	CVLT_9	Model 3c APOE x PICALM x age x education	APOE_PICALM	0.5893263132368811	2.5594106757134956	-4.705218059265581	5.883870685739343	0.8199265062314954	32
Women	1	CVLT_9	Model 3c APOE x PICALM x age x education	APOE_PICALM_age	-0.01120448179271688	0.8224126131295214	-1.7124945928477115	1.690085629262278	0.9892475124799482	32
Women	1	CVLT_9	Model 3c APOE x PICALM x age x education	APOE_PICALM_education	0.1541842098584848	0.6696132581808562	-1.2310163527148332	1.5393847724318026	0.8199265062314971	32
Women	1	CVLT_9	Model 3c APOE x PICALM x age x education	APOE_age_education	0.48438758135832427	2.7105065468101253	-5.122722414791075	6.091497577507723	0.8597322892008725	32
Women	1	CVLT_9	Model 3c APOE x PICALM x age x education	PICALM	-1.313594591058788	1.7680755083708521	-4.971137447245665	2.3439482651280894	0.46503243386626736	32
Women	1	CVLT_9	Model 3c APOE x PICALM x age x education	age_c	0.2832296641354035	0.26032728278517486	-0.2552983505978582	0.8217576788686652	0.2878758989855297	32
Women	1	CVLT_9	Model 3c APOE x PICALM x age x education	age_education	-0.4748908055457062	0.3402025449482507	-1.1786533892368307	0.22887177814541837	0.17607281343854536	32

Sex	Cluster	Outcome	Model	Predictor	Beta	SE	CI_low	CI_high	p	N
Women	1	CVLT_9	Model 3c APOE x PICALM x age x education	education_c	-2.5949673766259105	1.1035821374546724	-4.87790096399403	-0.3120337892577907	0.027641160641980474	32
Women	1	RPM	Model 1a APOE	APOE	0.11818181818182039	1.8714735432649046	-3.703877051924576	3.940240688288217	0.9500666836881251	32
Women	1	RPM	Model 1b APOE x age	APOE	0.7324688214764234	1.5277014898301853	-2.3968858208229626	3.861823463775809	0.6353365694358624	32
Women	1	RPM	Model 1b APOE x age	APOE_age	-1.1832560366990952	0.5740390872394291	-2.359121802669965	-0.007390270728225401	0.04867170203338427	32
Women	1	RPM	Model 1b APOE x age	age_c	-0.6119246861924674	0.3018023152692117	-1.2301387042002605	0.0062893318153256494	0.05221803906626596	32
Women	1	RPM	Model 1c APOE x age x education	APOE	-0.1985852603017746	1.0770317717669071	-2.4124557735223418	2.015285252918793	0.8551444065082819	32
Women	1	RPM	Model 1c APOE x age x education	APOE_age	-1.61896951399034	0.40814360525547816	-2.4579207097868103	-0.7800183181938695	0.0005099334002452065	32
Women	1	RPM	Model 1c APOE x age x education	APOE_age_education	-0.42356760540445126	0.10678175718893324	-0.6430606508163186	-0.204074559992584	0.0005099334002451835	32
Women	1	RPM	Model 1c APOE x age x education	APOE_education	-0.05195544600918739	0.2817815681948308	-0.6311657547006158	0.5272548626822411	0.8551444065082762	32
Women	1	RPM	Model 1c APOE x age x education	age_c	-0.35609029108928514	0.22585987264263757	-0.8203519083143562	0.10817132613578595	0.12697894829643733	32
Women	1	RPM	Model 1c APOE x age x education	age_education	1.1111054306565684	0.3009076310303645	0.49258093676136716	1.7296299245517697	0.0010369302153420469	32
Women	1	RPM	Model 1c APOE x age x education	education_c	1.9892513084943384	0.9542459326082907	0.027770702312775786	3.950731914675901	0.047067373125139235	32
Women	1	RPM	Model 2a PICALM	PICALM	0.11818181818181905	1.871473543264905	-3.7038770519245783	3.9402406882882164	0.9500666836881256	32
Women	1	RPM	Model 2b PICALM x age	PICALM	-0.8801769673616935	1.6500582957334993	-4.260168164720682	2.499814229997295	0.5979509067377606	32
Women	1	RPM	Model 2b PICALM x age	PICALM_age	-0.16874132593146052	0.600704638693395	-1.3992289979405426	1.0617463460776215	0.7808480918693395	32
Women	1	RPM	Model 2b PICALM x age	age_c	-0.9071207430340689	0.33436596182345324	-1.5920383672064664	-0.22220311886167132	0.011275991113810075	32
Women	1	RPM	Model 2c PICALM x age x education	PICALM	-1.5368838639849347	1.3212018640989476	-4.252653190030159	1.1788854620602898	0.255294516807297	32
Women	1	RPM	Model 2c PICALM x age x education	PICALM_age	-0.6060753638355796	0.521931268866558	-1.6789204519390168	0.4667697242678577	0.25610419234922	32
Women	1	RPM	Model 2c PICALM x age x education	PICALM_education	-0.40209170860070875	0.34566327839798044	-1.1126127532055639	0.30842933600414635	0.25529451680729826	32
Women	1	RPM	Model 2c PICALM x age x education	age_c	-0.7007846587041611	0.2840805053691657	-1.2847205004350253	-0.11684881697329685	0.02053766430779178	32
Women	1	RPM	Model 2c PICALM x age x education	age_education	0.8829255114392726	0.36988824132185655	0.12260934239435806	1.643241680484187	0.02454865313904244	32
Women	1	RPM	Model 2c PICALM x age x education	education_c	2.1788681731744344	1.1647065185799919	-0.2152203631459888	4.572956709494857	0.07267702349875332	32
Women	1	RPM	Model 3a APOE x PICALM	APOE	0.41176470588235414	2.582177785449676	-4.8775867112177895	5.701116122982498	0.8744479638616653	32

Sex	Cluster	Outcome	Model	Predictor	Beta	SE	CI_low	CI_high	p	N
Women	1	RPM	Model 3a APOE x PICALM	APOE_PICALM	-0.8117647058823749	4.11973056531563	-9.25065021814707	7.627120806382321	0.8452171239775446	32
Women	1	RPM	Model 3a APOE x PICALM	PICALM	0.4117647058823728	2.582177785449677	-4.8775867112177735	5.701116122982519	0.8744479638616597	32
Women	1	RPM	Model 3b APOE x PICALM x age	APOE	0.5139999999999985	2.157507511032614	-3.9208202030206474	4.948820203020644	0.8135642314782778	32
Women	1	RPM	Model 3b APOE x PICALM x age	APOE_PICALM	0.35742453260375395	3.4603095536277237	-6.755343621695204	7.4701926869027115	0.9185233245521658	32
Women	1	RPM	Model 3b APOE x PICALM x age	APOE_PICALM_age	-1.038431372549014	0.7322281198649558	-2.5435478287335513	0.4666850836355234	0.16801351914976687	32
Women	1	RPM	Model 3b APOE x PICALM x age	PICALM	-0.9079999999999835	2.218061243828164	-5.467290183401597	3.6512901834016303	0.6856236534308264	32
Women	1	RPM	Model 3b APOE x PICALM x age	age_c	-0.79	0.309047531299902	-1.4252562985268526	-0.15474370147314742	0.016769850205512023	32
Women	1	RPM	Model 3c APOE x PICALM x age x education	APOE	0.043987053347821936	1.6336095545557716	-3.335391784137234	3.423365890832878	0.9787507893988401	32
Women	1	RPM	Model 3c APOE x PICALM x age x education	APOE_PICALM	-0.01142423295658257	2.394685195370672	-4.965207986917931	4.942359521004765	0.9962347149897582	32
Women	1	RPM	Model 3c APOE x PICALM x age x education	APOE_PICALM_age	-0.04271708683472575	0.7694815559829417	-1.634510963695923	1.5490767900264715	0.9562083148619211	32
Women	1	RPM	Model 3c APOE x PICALM x age x education	APOE_PICALM_education	-0.002988898157241182	0.6265164755330245	-1.299036973321547	1.2930591770070645	0.9962347149897637	32
Women	1	RPM	Model 3c APOE x PICALM x age x education	APOE_age_education	-6.392534903688194	2.5360564293934678	-11.63876733680513	-1.1463024705712588	0.019102088009340646	32
Women	1	RPM	Model 3c APOE x PICALM x age x education	PICALM	-1.095274570885227	1.6542809187950396	-4.517415383321588	2.3268662415511345	0.5144964959306293	32
Women	1	RPM	Model 3c APOE x PICALM x age x education	age_c	-0.39558263911023683	0.24357243483910407	-0.8994506101284453	0.10828533190797163	0.11798460235056035	32
Women	1	RPM	Model 3c APOE x PICALM x age x education	age_education	1.0791428322238599	0.3183068686653388	0.4206749059106527	1.737610758537067	0.0025172262805205157	32
Women	1	RPM	Model 3c APOE x PICALM x age x education	education_c	2.1652619087260936	1.0325548109630756	0.029259540852533572	4.301264276599653	0.04718734162284076	32
