# Supplementary material for: Sex-specific personality metatrait profiles and their associations with APOE, PICALM variants, and cognitive performance in healthy middle-aged adults: a cluster-based analysis
Source: Front Aging. 2026 Oct 29;7:1933176. doi: 10.3389/fragi.2026.1933176 (PMC13754840; doi:10.3389/fragi.2026.1933176)
Supplement: Supplementary file 3 [file DataSheet3.pdf]

Supplementary materials

Agglomerative hierarchical clustering was evaluated separately for women and men across solutions ranging from two to nine clusters. The two-cluster solution was retained because it provided the highest silhouette coefficient in women and showed comparable performance in men while maintaining sufficient cluster interpretability and size. In females, the highest silhouette coefficient was observed for the two-cluster solution (0.4025), supporting the selection of this solution. In males, the silhouette coefficient was similar for the two-cluster (0.3805) and nine-cluster (0.3821) solutions. However, the nine-cluster solution resulted in smaller and less interpretable subgroups; therefore, the two-cluster solution was retained to ensure comparability between sexes and adequate cluster stability (see Table S1 and Figure S1, S2).

Table S1. Silhouette coefficients for hierarchical clustering solutions based on Stability and Plasticity metatraits.

Number of clusters (k)	Women (Silhouette coefficient)	Men (Silhouette coefficient)
**2**	0.4025	0.3805
**3**	0.3296	0.3582
**4**	0.3561	0.3242
**5**	0.3803	0.3278
**6**	0.3627	0.3471
**7**	0.3851	0.3493
**8**	0.3943	0.3694
**9**	0.3743	0.3821

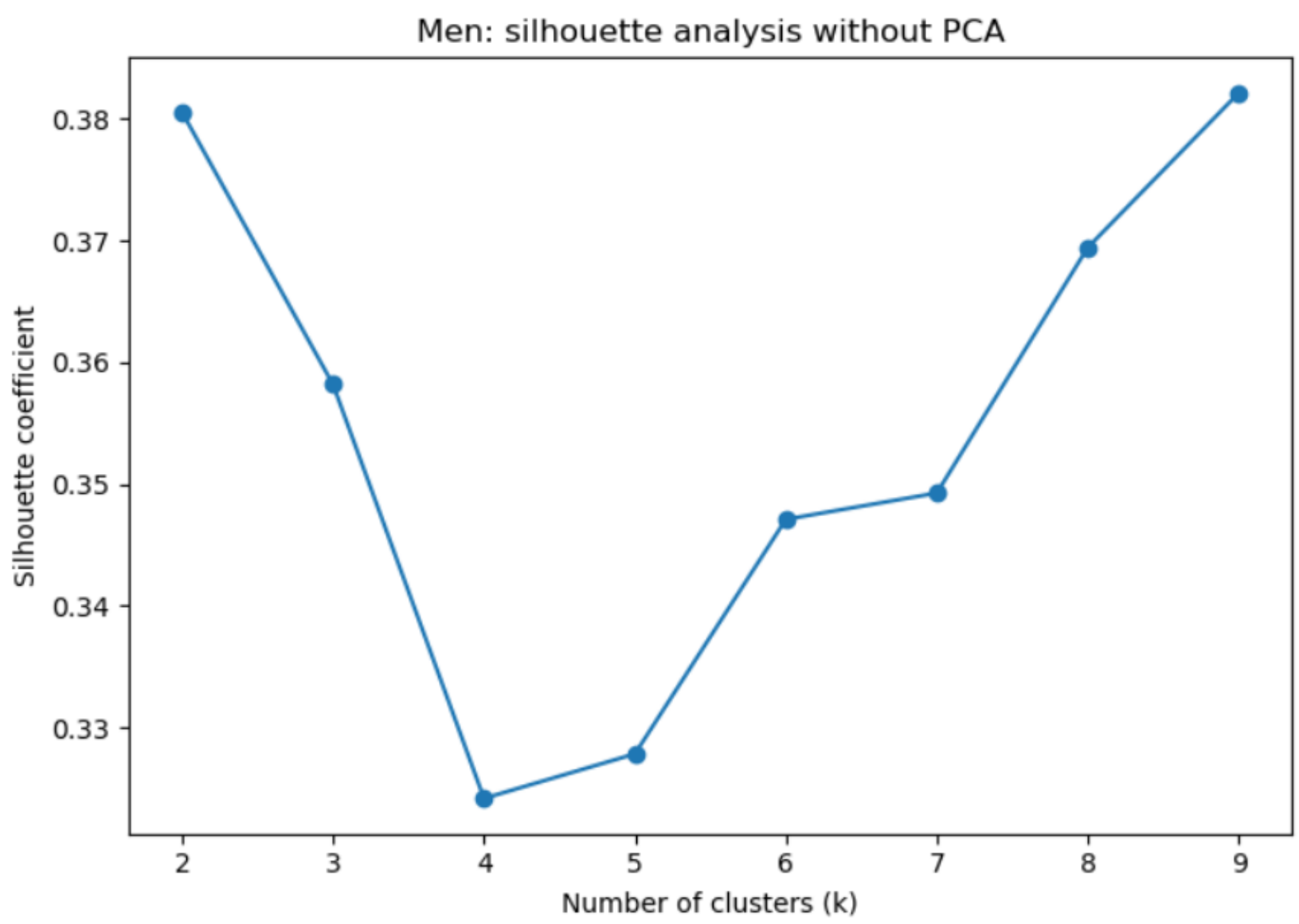

Figure S1. Silhoutte analysis in males sample.

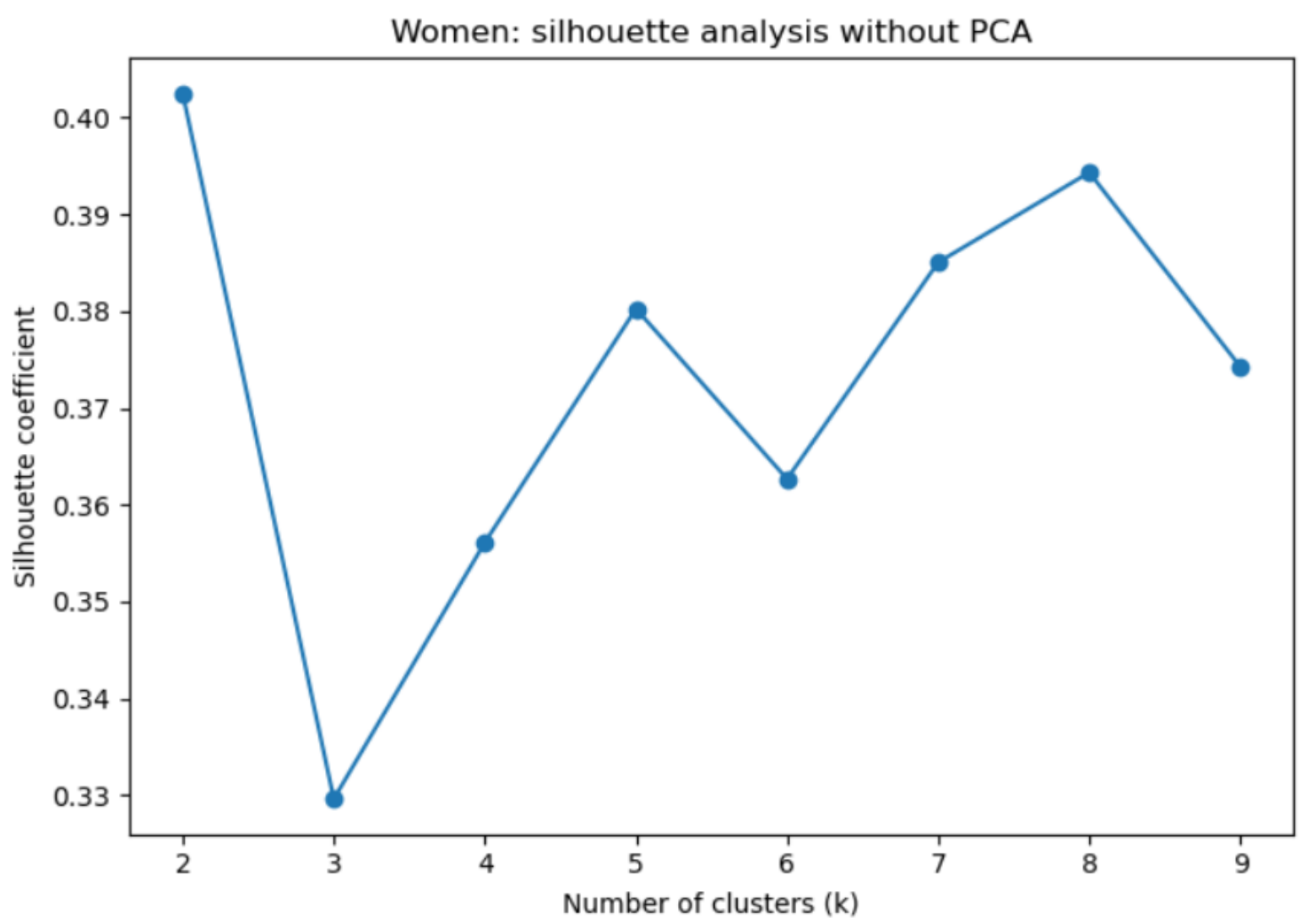

Figure S2. Silhoutte analysis in females sample.

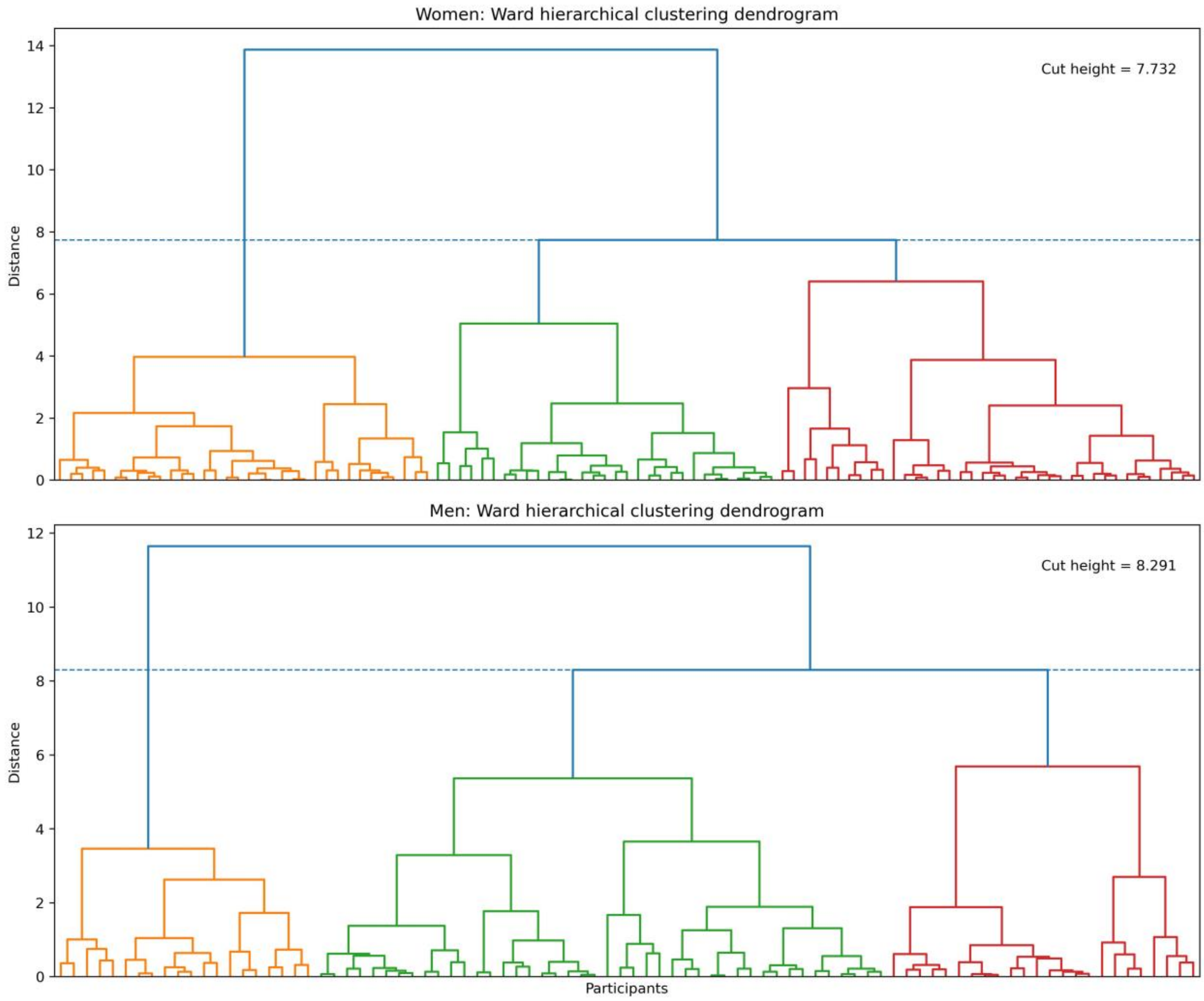

Figure S3. Hierarchical clustering dendrograms obtained using Ward's method for the female and male groups. Each observation initially forms a separate cluster, and clusters are then progressively merged based on their similarity.

Table S2. Cluster stability analyses for sex-stratified metatrait clusters

Sex	Analysis	Result
**Females**	Sample size	N = 103
**Females**	Original cluster sizes	Cluster 0: n = 69; Cluster 1: n = 34
**Females**	Bootstrap iterations	1000
**Females**	Mean bootstrap ARI ± SD	0.451 ± 0.339
**Females**	Mean individual assignment stability	63.5%
**Females**	Individual stability range	43.2–92.0%
**Females**	Average linkage ARI	0.145
**Females**	Complete linkage ARI	0.198
**Females**	K-means ARI	0.368
**Females**	Gaussian mixture model ARI	0.278
**Females**	Participants switching cluster (k-means comparison)	20/103 (19.4%)
**Males**	Sample size	N = 88
**Males**	Original cluster sizes	Cluster 0: n = 68; Cluster 1: n = 20
**Males**	Bootstrap iterations	1000
**Males**	Mean bootstrap ARI ± SD	0.552 ± 0.383
**Males**	Mean individual assignment stability	76.6%
**Males**	Individual stability range	51.8–95.3%
**Males**	Average linkage ARI	0.950
**Males**	Complete linkage ARI	0.123
**Males**	K-means ARI	0.340
**Males**	Gaussian mixture model ARI	0.337
**Males**	Participants switching cluster (k-means comparison)	18/88 (20.5%)

Regression Model Diagnostics and Assumption Checks

Additional diagnostic analyses were performed for all regression models that remained significant after false discovery rate (FDR) correction (Table S3). Model assumptions were evaluated using the Shapiro–Wilk test for residual normality, the Breusch–Pagan test for homoscedasticity, the Durbin–Watson statistic for residual autocorrelation, variance inflation factors (VIF) and the condition number to assess multicollinearity and model stability, and Cook's distance to identify influential observations. Influential cases were defined using the conventional threshold of Cook's distance greater than 4/N.

Residuals deviated from normality in six of the seven FDR-significant models (Shapiro–Wilk $p < 0.05$), whereas the PICALM × age × education model predicting RPM performance did not show evidence of non-normality ($p = 0.333$). Homoscedasticity was generally supported, with significant heteroscedasticity observed only for the APOE × age model predicting CVLT-12 performance in Cluster 0 (Breusch–Pagan $p = 0.003$). Durbin–Watson statistics ranged from 1.76 to 2.35, indicating no substantial residual autocorrelation. Cook's distance identified between zero and four potentially influential observations across models.

Variance inflation factors were generally low for the continuous predictors (approximately 1.2–3.2). However, several higher-order interaction models produced infinite VIF values because some genotype-by-cluster combinations contained very few observations, resulting in near-perfect linear dependence among predictors. This reflects sparse data within relatively small subgroup samples rather than a computational error. Together with the robustness analyses presented in Table S4, these findings indicate that the principal conclusions were generally robust to violations of model assumptions. Nevertheless, the higher-order interaction models should be regarded as exploratory and interpreted with appropriate caution.

Table S3. Diagnostic statistics for regression models that remained significant after false discovery rate correction (females)

Outcome	Model	Shapiro–Wilk W	Shapiro–Wilk p	Breusch–Pagan p	Durbin–Watson	Condition Number	Maximum Cook's D	Cook's D threshold	Influential cases
Cluster 0 (n=64)									
CVLT_12	Model 1b APOE × age	0.830	<0.001	0.0026	1.941	8.95	0.875	0.0625	4
Cluster 0 (n=32)									
RPM	Model 1c APOE × age × education	0.913	0.0139	0.846	1.757	3.04×10^{17}	0.214	0.125	2
CVLT_12	Model 2c PICALM × age × education	0.754	<0.001	0.225	2.269	1.52×10^{18}	1.480	0.125	3
RPM	Model 3c APOE × PICALM × age × education	0.896	0.0050	0.926	1.824	2.72×10^{17}	0.172	0.125	2
CVLT_12	Model 1c APOE × age × education	0.716	<0.001	0.869	2.351	3.04×10^{17}	1.178	0.125	3
CVLT_12	Model 3c APOE × PICALM × age × education	0.893	0.0041	0.266	2.166	2.72×10^{17}	1.070	0.125	4
RPM	Model 2c PICALM × age × education	0.963	0.333	0.226	2.172	1.52×10^{18}	0.111	0.125	0

Robustness analyses were performed for all regression models that remained significant after false discovery rate (FDR) correction (Table S4). For each regression coefficient, the table reports the ordinary least squares (OLS) estimate (β), conventional standard error, heteroscedasticity-consistent HC3 standard error, HC3-adjusted *p*-value, classical 95% confidence interval, HC3-based 95% confidence interval, and bootstrap-derived 95% confidence interval based on 1000 resampling iterations.

Overall, HC3-adjusted standard errors were similar to the conventional standard errors, indicating that the principal findings were not materially influenced by potential heteroscedasticity. Likewise, bootstrap confidence intervals were generally consistent with both the classical and HC3 confidence intervals, providing additional support for the stability of the main parameter estimates.

The strongest evidence of robustness was observed for the interaction effects underlying the FDR-significant models, particularly the APOE × age and APOE × age × education interactions predicting RPM performance in women, which remained consistently negative across estimation methods. In contrast, several coefficients from models containing multiple higher-order interaction terms exhibited wider bootstrap confidence intervals, reflecting the increased uncertainty associated with relatively small cluster-specific samples and greater model complexity. Accordingly, while the primary FDR-significant associations appeared robust across estimation approaches, individual higher-order interaction coefficients should be interpreted with appropriate caution.

Table S4. Robustness and stability of parameter estimates (females)

Outcome	Model	Parameter	Beta	SE_classical	SE_HC3	p_HC3	CI95_classical_low	CI95_classical_high	CI95_HC3_low	CI95_HC3_high	Bootstrap_CI_low	Bootstrap_CI_high
**Cluster 0**												
CVLT_12	Model 1b APOE x age	Intercept	15.581207	0.105271	0.093217	8.742837e-82	15.370634	15.791780	15.394746	15.767669	1.538624e+01	15.754818
CVLT_12	Model 1b APOE x age	APOE	-0.396307	0.229143	0.265495	1.407538e-01	-0.854660	0.062047	-0.927376	0.134763	-8.591270e-01	0.192267
CVLT_12	Model 1b APOE x age	age_c	0.002740	0.030550	0.023858	9.089607e-01	-0.058369	0.063848	-0.044984	0.050463	-4.379618e-02	0.042832
CVLT_12	Model 1b APOE x age	APOE_age	-0.229210	0.068365	0.114907	5.061876e-02	-0.365960	-0.092461	-0.459058	0.000638	-3.925062e-01	-0.016884
**Cluster 1**												
RPM	Model 1c APOE x age x education	APOE	-0.198585	1.077032	1.076935	8.551316e-01	-2.412456	2.015285	-2.412257	2.015087	-2.360584e+00	2.078849
RPM	Model 1c APOE x age x education	age_c	-0.356090	0.225860	0.275947	2.082597e-01	-0.820352	0.108171	-0.923307	0.211127	-1.300240e+00	0.494346
RPM	Model 1c APOE x age x education	education_c	1.989251	0.954246	1.107854	8.419223e-02	0.027771	3.950732	-0.287975	4.266478	5.476111e-01	12.988862
RPM	Model 1c APOE x age x education	APOE_age	-1.618970	0.408144	0.371431	1.825402e-04	-2.457921	-0.780018	-2.382457	-0.855482	-2.317680e+00	-0.738134

RPM	Model 1c APOE x age x education	APOE_education	-0.051955	0.281782	0.281756	8.551316e-01	-0.631166	0.527255	-0.631114	0.527203	-6.086565e-01	0.479483
RPM	Model 1c APOE x age x education	age_education	1.111105	0.300908	0.317845	1.714615e-03	0.492581	1.729630	0.457767	1.764444	-3.577029e+00	4.963845
RPM	Model 1c APOE x age x education	APOE_age_education	-0.423568	0.106782	0.097177	1.825402e-04	-0.643061	-0.204075	-0.623317	-0.223818	-5.951780e-01	-0.219333
CVLT_12	Model 2c PICALM x age x education	Intercept	15.649556	0.085997	0.114819	1.204119e-38	15.472786	15.826326	15.413541	15.885571	1.471615e+01	15.827393
CVLT_12	Model 2c PICALM x age x education	PICALM	0.262126	0.148736	0.112760	2.816818e-02	-0.043605	0.567857	0.030345	0.493907	7.184603e-02	0.490866
CVLT_12	Model 2c PICALM x age x education	age_c	-0.030558	0.031981	0.042655	4.801332e-01	-0.096295	0.035180	-0.118236	0.057121	-3.847683e-01	0.291267
CVLT_12	Model 2c PICALM x age x education	education_c	0.268993	0.131118	0.225562	2.438186e-01	-0.000525	0.538511	-0.194657	0.732643	3.387032e-02	3.749685
CVLT_12	Model 2c PICALM x age x education	PICALM_age	-0.011003	0.058757	0.042472	7.976200e-01	-0.131780	0.109774	-0.098306	0.076299	-9.938937e-02	0.067032
CVLT_12	Model 2c PICALM x age x education	PICALM_education	0.068579	0.038913	0.029501	2.816818e-02	-0.011408	0.148567	0.007939	0.129220	1.720795e-02	0.128582
CVLT_12	Model 2c PICALM x age x education	age_education	0.158856	0.041641	0.102109	1.318585e-01	0.073262	0.244449	-0.051033	0.368744	-1.181659e+00	1.534083
RPM	Model 3c APOE x PICALM x age x education	Intercept	53.388274	0.755837	0.861848	4.277843e-27	51.824705	54.951843	51.605406	55.171142	5.007733e+01	54.763840
RPM	Model 3c APOE x PICALM x	APOE	0.043987	1.633610	1.662178	9.791159e-01	-3.335392	3.423366	-3.394490	3.482464	-3.582498e+00	6.282371

	age x education											
RPM	Model 3c APOE x PICALM x age x education	PICALM	-1.095275	1.654281	1.198516	3.702720e-01	-4.517415	2.326866	-3.574593	1.384044	-3.309739e+00	1.229504
RPM	Model 3c APOE x PICALM x age x education	age_c	-0.395583	0.243572	0.259947	1.416945e-01	-0.899451	0.108285	-0.933324	0.142159	-1.431678e+00	0.665628
RPM	Model 3c APOE x PICALM x age x education	education_c	2.165262	1.032555	1.104852	6.224871e-02	0.029260	4.301264	-0.120298	4.450821	5.007297e-01	12.985732
RPM	Model 3c APOE x PICALM x age x education	APOE_PICALM	-0.011424	2.394685	2.196624	9.958952e-01	-4.965208	4.942360	-4.555487	4.532638	-9.081198e+00	3.916549
RPM	Model 3c APOE x PICALM x age x education	APOE_PICALM_age	-0.042717	0.769482	0.760895	9.557147e-01	-1.634511	1.549077	-1.616748	1.531314	-2.069716e+00	4.791364
RPM	Model 3c APOE x PICALM x age x education	APOE_PICALM_education	-0.002989	0.626516	0.574698	9.958952e-01	-1.299037	1.293059	-1.191842	1.185865	-2.493791e+00	0.970952
RPM	Model 3c APOE x PICALM x age x education	age_education	1.079143	0.318307	0.302985	1.658437e-03	0.420675	1.737611	0.452370	1.705915	-3.535801e+00	4.997912
RPM	Model 3c APOE x PICALM x age x education	APOE_age_education	-6.392535	2.536056	2.328115	1.151407e-02	-11.638767	-1.146302	-11.208608	-1.576462	-2.261732e+01	-0.858073
CVLT_12	Model 1c APOE x age x education	Intercept	15.749202	0.088207	0.096686	1.175754e-40	15.567890	15.930515	15.550462	15.947943	1.484634e+01	15.880864
CVLT_12	Model 1c APOE	APOE	-0.017588	0.152018	0.150091	9.076148e-01	-0.330066	0.294890	-0.326104	0.290928	-3.288670e-01	0.263991

	x age x education											
CVLT_12	Model 1c APOE x age x education	age_c	-0.020643	0.031879	0.035930	5.705416e-01	-0.086172	0.044885	-0.094498	0.053212	-3.520685e-01	0.305631
CVLT_12	Model 1c APOE x age x education	education_c	0.332172	0.134688	0.226836	1.550799e-01	0.055318	0.609027	-0.134096	0.798440	1.032165e-01	3.765474
CVLT_12	Model 1c APOE x age x education	APOE_age	-0.065656	0.057608	0.051662	2.150236e-01	-0.184071	0.052758	-0.171849	0.040536	-1.926908e-01	0.022450
CVLT_12	Model 1c APOE x age x education	APOE_education	-0.004602	0.039772	0.039268	9.076148e-01	-0.086355	0.077151	-0.085318	0.076115	-8.503369e-02	0.067273
CVLT_12	Model 1c APOE x age x education	age_education	0.162830	0.042472	0.100328	1.166580e-01	0.075528	0.250132	-0.043397	0.369057	-1.184553e+00	1.548201
CVLT_12	Model 1c APOE x age x education	APOE_age_education	-0.017178	0.015072	0.013516	2.150236e-01	-0.048158	0.013803	-0.044961	0.010605	-4.833890e-02	0.004629
CVLT_12	Model 1b APOE x age	Intercept	15.581207	0.105271	0.093217	8.742837e-82	15.370634	15.791780	15.394746	15.767669	1.538624e+01	15.754818
CVLT_12	Model 1b APOE x age	APOE	-0.396307	0.229143	0.265495	1.407538e-01	-0.854660	0.062047	-0.927376	0.134763	-8.591270e-01	0.192267
CVLT_12	Model 1b APOE x age	age_c	0.002740	0.030550	0.023858	9.089607e-01	-0.058369	0.063848	-0.044984	0.050463	-4.379618e-02	0.042832
CVLT_12	Model 1b APOE x age	APOE_age	-0.229210	0.068365	0.114907	5.061876e-02	-0.365960	-0.092461	-0.459058	0.000638	-3.925062e-01	-0.016884
CVLT_12	Model 3c APOE x PICALM x age x education	Intercept	15.684088	0.097668	0.125401	4.311013e-34	15.482046	15.886130	15.424675	15.943501	1.474774e+01	15.890841
CVLT_12	Model 3c APOE x PICALM x age x education	APOE	-0.096928	0.211093	0.273583	7.263488e-01	-0.533606	0.339751	-0.662876	0.469021	-2.758224e+00	0.343994
CVLT_12	Model 3c APOE x PICA	PICALM	0.283541	0.213764	0.173428	1.156803e-01	-0.158663	0.725745	-0.075223	0.642304	8.659740e-16	0.663254

	LM x age x education											
CVLT_12	Model 3c APOE x PICALM x age x education	age_c	-0.010419	0.031474	0.038944	7.914316e-01	-0.075529	0.054690	-0.090982	0.070143	-3.099518e-01	0.296856
CVLT_12	Model 3c APOE x PICALM x age x education	education_c	0.286607	0.133425	0.227239	2.198579e-01	0.010596	0.562618	-0.183471	0.756686	3.167637e-02	3.767632
CVLT_12	Model 3c APOE x PICALM x age x education	APOE_PICALM	0.050835	0.309438	0.275445	8.551955e-01	-0.589286	0.690955	-0.518966	0.620636	-3.862641e-01	2.611407
CVLT_12	Model 3c APOE x PICALM x age x education	APOE_PICALM_age	0.111111	0.099431	0.122847	3.751254e-01	-0.094578	0.316800	-0.143018	0.365240	-1.000000e+00	0.333333
CVLT_12	Model 3c APOE x PICALM x age x education	APOE_PICALM_education	0.013300	0.080958	0.072064	8.551955e-01	-0.154174	0.180773	-0.135776	0.162376	-1.043091e-01	0.661036
CVLT_12	Model 3c APOE x PICALM x age x education	age_education	0.171105	0.041131	0.101733	1.061198e-01	0.086018	0.256191	-0.039347	0.381556	-1.178487e+00	1.576230
CVLT_12	Model 3c APOE x PICALM x age x education	APOE_age_education	-0.555970	0.327705	0.490731	2.689108e-01	-1.233881	0.121940	-1.571125	0.459184	-1.495000e+00	3.730639
RPM	Model 2c PICALM x age x education	Intercept	53.290313	0.763903	0.769143	4.998601e-31	51.720089	54.860538	51.709318	54.871309	5.011776e+01	54.517297
RPM	Model 2c PICALM x age x education	PICALM	-1.536884	1.321202	1.269581	2.369603e-01	-4.252653	1.178885	-4.146545	1.072778	-3.790211e+00	0.716215
RPM	Model 2c PICALM x	age_c	-0.700785	0.284081	0.316849	3.597910e-02	-1.284721	-0.116849	-1.352077	-0.049492	-1.715813e+00	0.298475

	age x education											
RPM	Model 2c PICALM x age x education	education_c	2.178868	1.164707	0.950032	3.015383e-02	-0.215220	4.572957	0.226049	4.131687	7.499466e-01	12.821244
RPM	Model 2c PICALM x age x education	PICALM_age	-0.606075	0.521931	0.558006	2.873831e-01	-1.678920	0.466770	-1.753074	0.540923	-1.619302e+00	0.368173
RPM	Model 2c PICALM x age x education	PICALM_education	-0.402092	0.345663	0.332158	2.369603e-01	-1.112613	0.308429	-1.084852	0.280669	-1.010831e+00	0.194957
RPM	Model 2c PICALM x age x education	age_education	0.882926	0.369888	0.304624	7.520649e-03	0.122609	1.643242	0.25676			

Table S5A. Variance inflation factors for individual predictors

	Sex	Cluster	Outcome	Model	Max_VIF	Mean_VIF
**0**	Women	0	CVLT_12	Model 1b APOE x age	1.325192	1.216795
**1**	Women	1	CVLT_12	Model 1c APOE x age x education	inf	inf
**2**	Women	1	CVLT_12	Model 2c PICALM x age x education	inf	inf
**3**	Women	1	CVLT_12	Model 3c APOE x PICALM x age x education	inf	inf
**4**	Women	1	RPM	Model 1c APOE x age x education	inf	inf
**5**	Women	1	RPM	Model 2c PICALM x age x education	inf	inf
**6**	Women	1	RPM	Model 3c APOE x PICALM x age x education	inf	inf

Table S5B. Summary of multicollinearity diagnostics

Model	Max VIF	Interpretation
**Model 1b APOE × age**	1.33	No evidence of problematic multicollinearity
**Higher-order interaction models**	∞	Expected dependency due to interaction terms

The ratio of observations per estimated parameter was examined to evaluate the potential risk of model overfitting. Simpler models (main effects only) showed adequate

observation-to-parameter ratios across all subgroups. However, models including multiple interaction terms (particularly APOE × PICALM × age × education models) resulted in substantially lower observations-per-parameter ratios in smaller clusters. This limitation was most pronounced in Cluster 1 among men (N = 17), where the most complex models included up to 10 parameters, corresponding to fewer than 2 observations per parameter. Therefore, estimates from highly parameterized models in small subgroups should be interpreted cautiously (see Table S5C).

Table S5C. Regression model complexity and number of observations per estimated parameter across analytical subgroups.

Sex	Cluster	Outcome	Model	N	Parameters	N_per_parameter
Women	0	CVLT_1	Model 1a APOE	64	2	32.000000
Women	0	CVLT_1	Model 1b APOE x age	64	4	16.000000
Women	0	CVLT_1	Model 1c APOE x age x education	64	8	8.000000
Women	0	CVLT_1	Model 2a PICALM	64	2	32.000000
Women	0	CVLT_1	Model 2b PICALM x age	64	4	16.000000
Women	0	CVLT_1	Model 2c PICALM x age x education	64	7	9.142857
Women	0	CVLT_1	Model 3a APOE x PICALM	64	4	16.000000
Women	0	CVLT_1	Model 3b APOE x PICALM x age	64	6	10.666667
Women	0	CVLT_1	Model 3c APOE x PICALM x age x education	64	10	6.400000
Women	0	CVLT_2	Model 1a APOE	64	2	32.000000
Women	0	CVLT_2	Model 1b APOE x age	64	4	16.000000
Women	0	CVLT_2	Model 1c APOE x age x education	64	8	8.000000
Women	0	CVLT_2	Model 2a PICALM	64	2	32.000000
Women	0	CVLT_2	Model 2b PICALM x age	64	4	16.000000
Women	0	CVLT_2	Model 2c PICALM x age x education	64	7	9.142857
Women	0	CVLT_2	Model 3a APOE x PICALM	64	4	16.000000
Women	0	CVLT_2	Model 3b APOE x PICALM x age	64	6	10.666667
Women	0	CVLT_2	Model 3c APOE x PICALM x age x education	64	10	6.400000
Women	0	CVLT_3	Model 1a APOE	64	2	32.000000
Women	0	CVLT_3	Model 1b APOE x age	64	4	16.000000
Women	0	CVLT_3	Model 1c APOE x age x education	64	8	8.000000
Women	0	CVLT_3	Model 2a PICALM	64	2	32.000000

Women	0	CVLT_3	Model 2b PICALM x age	64	4	16.000000
Women	0	CVLT_3	Model 2c PICALM x age x education	64	7	9.142857
Women	0	CVLT_3	Model 3a APOE x PICALM	64	4	16.000000
Women	0	CVLT_3	Model 3b APOE x PICALM x age	64	6	10.666667
Women	0	CVLT_3	Model 3c APOE x PICALM x age x education	64	10	6.400000
Women	0	CVLT_4	Model 1a APOE	64	2	32.000000
Women	0	CVLT_4	Model 1b APOE x age	64	4	16.000000
Women	0	CVLT_4	Model 1c APOE x age x education	64	8	8.000000
Women	0	CVLT_4	Model 2a PICALM	64	2	32.000000
Women	0	CVLT_4	Model 2b PICALM x age	64	4	16.000000
Women	0	CVLT_4	Model 2c PICALM x age x education	64	7	9.142857
Women	0	CVLT_4	Model 3a APOE x PICALM	64	4	16.000000
Women	0	CVLT_4	Model 3b APOE x PICALM x age	64	6	10.666667
Women	0	CVLT_4	Model 3c APOE x PICALM x age x education	64	10	6.400000
Women	0	CVLT_5	Model 1a APOE	64	2	32.000000
Women	0	CVLT_5	Model 1b APOE x age	64	4	16.000000
Women	0	CVLT_5	Model 1c APOE x age x education	64	8	8.000000
Women	0	CVLT_5	Model 2a PICALM	64	2	32.000000
Women	0	CVLT_5	Model 2b PICALM x age	64	4	16.000000
Women	0	CVLT_5	Model 2c PICALM x age x education	64	7	9.142857
Women	0	CVLT_5	Model 3a APOE x PICALM	64	4	16.000000
Women	0	CVLT_5	Model 3b APOE x PICALM x age	64	6	10.666667
Women	0	CVLT_5	Model 3c APOE x PICALM x age x education	64	10	6.400000
Women	0	CVLT_6	Model 1a APOE	64	2	32.000000
Women	0	CVLT_6	Model 1b APOE x age	64	4	16.000000
Women	0	CVLT_6	Model 1c APOE x age x education	64	8	8.000000
Women	0	CVLT_6	Model 2a PICALM	64	2	32.000000
Women	0	CVLT_6	Model 2b PICALM x age	64	4	16.000000
Women	0	CVLT_6	Model 2c PICALM x age x education	64	7	9.142857
Women	0	CVLT_6	Model 3a APOE x PICALM	64	4	16.000000

Women	0	CVLT_6	Model 3b APOE x PICALM x age	64	6	10.666667
Women	0	CVLT_6	Model 3c APOE x PICALM x age x education	64	10	6.400000
Women	0	CVLT_7	Model 1a APOE	64	2	32.000000
Women	0	CVLT_7	Model 1b APOE x age	64	4	16.000000
Women	0	CVLT_7	Model 1c APOE x age x education	64	8	8.000000
Women	0	CVLT_7	Model 2a PICALM	64	2	32.000000
Women	0	CVLT_7	Model 2b PICALM x age	64	4	16.000000
Women	0	CVLT_7	Model 2c PICALM x age x education	64	7	9.142857
Women	0	CVLT_7	Model 3a APOE x PICALM	64	4	16.000000
Women	0	CVLT_7	Model 3b APOE x PICALM x age	64	6	10.666667
Women	0	CVLT_7	Model 3c APOE x PICALM x age x education	64	10	6.400000
Women	0	CVLT_8	Model 1a APOE	64	2	32.000000
Women	0	CVLT_8	Model 1b APOE x age	64	4	16.000000
Women	0	CVLT_8	Model 1c APOE x age x education	64	8	8.000000
Women	0	CVLT_8	Model 2a PICALM	64	2	32.000000
Women	0	CVLT_8	Model 2b PICALM x age	64	4	16.000000
Women	0	CVLT_8	Model 2c PICALM x age x education	64	7	9.142857
Women	0	CVLT_8	Model 3a APOE x PICALM	64	4	16.000000
Women	0	CVLT_8	Model 3b APOE x PICALM x age	64	6	10.666667
Women	0	CVLT_8	Model 3c APOE x PICALM x age x education	64	10	6.400000
Women	0	CVLT_9	Model 1a APOE	64	2	32.000000
Women	0	CVLT_9	Model 1b APOE x age	64	4	16.000000
Women	0	CVLT_9	Model 1c APOE x age x education	64	8	8.000000
Women	0	CVLT_9	Model 2a PICALM	64	2	32.000000
Women	0	CVLT_9	Model 2b PICALM x age	64	4	16.000000
Women	0	CVLT_9	Model 2c PICALM x age x education	64	7	9.142857
Women	0	CVLT_9	Model 3a APOE x PICALM	64	4	16.000000
Women	0	CVLT_9	Model 3b APOE x PICALM x age	64	6	10.666667
Women	0	CVLT_9	Model 3c APOE x PICALM x age x education	64	10	6.400000
Women	0	CVLT_10	Model 1a APOE	64	2	32.000000

Women	0	CVLT_10	Model 1b APOE x age	64	4	16.000000
Women	0	CVLT_10	Model 1c APOE x age x education	64	8	8.000000
Women	0	CVLT_10	Model 2a PICALM	64	2	32.000000
Women	0	CVLT_10	Model 2b PICALM x age	64	4	16.000000
Women	0	CVLT_10	Model 2c PICALM x age x education	64	7	9.142857
Women	0	CVLT_10	Model 3a APOE x PICALM	64	4	16.000000
Women	0	CVLT_10	Model 3b APOE x PICALM x age	64	6	10.666667
Women	0	CVLT_10	Model 3c APOE x PICALM x age x education	64	10	6.400000
Women	0	CVLT_11	Model 1a APOE	64	2	32.000000
Women	0	CVLT_11	Model 1b APOE x age	64	4	16.000000
Women	0	CVLT_11	Model 1c APOE x age x education	64	8	8.000000
Women	0	CVLT_11	Model 2a PICALM	64	2	32.000000
Women	0	CVLT_11	Model 2b PICALM x age	64	4	16.000000
Women	0	CVLT_11	Model 2c PICALM x age x education	64	7	9.142857
Women	0	CVLT_11	Model 3a APOE x PICALM	64	4	16.000000
Women	0	CVLT_11	Model 3b APOE x PICALM x age	64	6	10.666667
Women	0	CVLT_11	Model 3c APOE x PICALM x age x education	64	10	6.400000
Women	0	CVLT_12	Model 1a APOE	64	2	32.000000
Women	0	CVLT_12	Model 1b APOE x age	64	4	16.000000
Women	0	CVLT_12	Model 1c APOE x age x education	64	8	8.000000
Women	0	CVLT_12	Model 2a PICALM	64	2	32.000000
Women	0	CVLT_12	Model 2b PICALM x age	64	4	16.000000
Women	0	CVLT_12	Model 2c PICALM x age x education	64	7	9.142857
Women	0	CVLT_12	Model 3a APOE x PICALM	64	4	16.000000
Women	0	CVLT_12	Model 3b APOE x PICALM x age	64	6	10.666667
Women	0	CVLT_12	Model 3c APOE x PICALM x age x education	64	10	6.400000
Women	0	CVLT_13	Model 1a APOE	64	2	32.000000
Women	0	CVLT_13	Model 1b APOE x age	64	4	16.000000
Women	0	CVLT_13	Model 1c APOE x age x education	64	8	8.000000
Women	0	CVLT_13	Model 2a PICALM	64	2	32.000000
Women	0	CVLT_13	Model 2b PICALM x age	64	4	16.000000

Women	0	CVLT_13	Model 2c PICALM x age x education	64	7	9.142857
Women	0	CVLT_13	Model 3a APOE x PICALM	64	4	16.000000
Women	0	CVLT_13	Model 3b APOE x PICALM x age	64	6	10.666667
Women	0	CVLT_13	Model 3c APOE x PICALM x age x education	64	10	6.400000
Women	0	RPM	Model 1a APOE	64	2	32.000000
Women	0	RPM	Model 1b APOE x age	64	4	16.000000
Women	0	RPM	Model 1c APOE x age x education	64	8	8.000000
Women	0	RPM	Model 2a PICALM	64	2	32.000000
Women	0	RPM	Model 2b PICALM x age	64	4	16.000000
Women	0	RPM	Model 2c PICALM x age x education	64	7	9.142857
Women	0	RPM	Model 3a APOE x PICALM	64	4	16.000000
Women	0	RPM	Model 3b APOE x PICALM x age	64	6	10.666667
Women	0	RPM	Model 3c APOE x PICALM x age x education	64	10	6.400000
Women	1	CVLT_1	Model 1a APOE	32	2	16.000000
Women	1	CVLT_1	Model 1b APOE x age	32	4	8.000000
Women	1	CVLT_1	Model 1c APOE x age x education	32	8	4.000000
Women	1	CVLT_1	Model 2a PICALM	32	2	16.000000
Women	1	CVLT_1	Model 2b PICALM x age	32	4	8.000000
Women	1	CVLT_1	Model 2c PICALM x age x education	32	7	4.571429
Women	1	CVLT_1	Model 3a APOE x PICALM	32	4	8.000000
Women	1	CVLT_1	Model 3b APOE x PICALM x age	32	6	5.333333
Women	1	CVLT_1	Model 3c APOE x PICALM x age x education	32	10	3.200000
Women	1	CVLT_2	Model 1a APOE	32	2	16.000000
Women	1	CVLT_2	Model 1b APOE x age	32	4	8.000000
Women	1	CVLT_2	Model 1c APOE x age x education	32	8	4.000000
Women	1	CVLT_2	Model 2a PICALM	32	2	16.000000
Women	1	CVLT_2	Model 2b PICALM x age	32	4	8.000000
Women	1	CVLT_2	Model 2c PICALM x age x education	32	7	4.571429
Women	1	CVLT_2	Model 3a APOE x PICALM	32	4	8.000000
Women	1	CVLT_2	Model 3b APOE x PICALM x age	32	6	5.333333

Women	1	CVLT_2	Model 3c APOE x PICALM x age x education	32	10	3.200000
Women	1	CVLT_3	Model 1a APOE	32	2	16.000000
Women	1	CVLT_3	Model 1b APOE x age	32	4	8.000000
Women	1	CVLT_3	Model 1c APOE x age x education	32	8	4.000000
Women	1	CVLT_3	Model 2a PICALM	32	2	16.000000
Women	1	CVLT_3	Model 2b PICALM x age	32	4	8.000000
Women	1	CVLT_3	Model 2c PICALM x age x education	32	7	4.571429
Women	1	CVLT_3	Model 3a APOE x PICALM	32	4	8.000000
Women	1	CVLT_3	Model 3b APOE x PICALM x age	32	6	5.333333
Women	1	CVLT_3	Model 3c APOE x PICALM x age x education	32	10	3.200000
Women	1	CVLT_4	Model 1a APOE	32	2	16.000000
Women	1	CVLT_4	Model 1b APOE x age	32	4	8.000000
Women	1	CVLT_4	Model 1c APOE x age x education	32	8	4.000000
Women	1	CVLT_4	Model 2a PICALM	32	2	16.000000
Women	1	CVLT_4	Model 2b PICALM x age	32	4	8.000000
Women	1	CVLT_4	Model 2c PICALM x age x education	32	7	4.571429
Women	1	CVLT_4	Model 3a APOE x PICALM	32	4	8.000000
Women	1	CVLT_4	Model 3b APOE x PICALM x age	32	6	5.333333
Women	1	CVLT_4	Model 3c APOE x PICALM x age x education	32	10	3.200000
Women	1	CVLT_5	Model 1a APOE	32	2	16.000000
Women	1	CVLT_5	Model 1b APOE x age	32	4	8.000000
Women	1	CVLT_5	Model 1c APOE x age x education	32	8	4.000000
Women	1	CVLT_5	Model 2a PICALM	32	2	16.000000
Women	1	CVLT_5	Model 2b PICALM x age	32	4	8.000000
Women	1	CVLT_5	Model 2c PICALM x age x education	32	7	4.571429
Women	1	CVLT_5	Model 3a APOE x PICALM	32	4	8.000000
Women	1	CVLT_5	Model 3b APOE x PICALM x age	32	6	5.333333
Women	1	CVLT_5	Model 3c APOE x PICALM x age x education	32	10	3.200000
Women	1	CVLT_6	Model 1a APOE	32	2	16.000000
Women	1	CVLT_6	Model 1b APOE x age	32	4	8.000000

Women	1	CVLT_6	Model 1c APOE x age x education	32	8	4.000000
Women	1	CVLT_6	Model 2a PICALM	32	2	16.000000
Women	1	CVLT_6	Model 2b PICALM x age	32	4	8.000000
Women	1	CVLT_6	Model 2c PICALM x age x education	32	7	4.571429
Women	1	CVLT_6	Model 3a APOE x PICALM	32	4	8.000000
Women	1	CVLT_6	Model 3b APOE x PICALM x age	32	6	5.333333
Women	1	CVLT_6	Model 3c APOE x PICALM x age x education	32	10	3.200000
Women	1	CVLT_7	Model 1a APOE	32	2	16.000000
Women	1	CVLT_7	Model 1b APOE x age	32	4	8.000000
Women	1	CVLT_7	Model 1c APOE x age x education	32	8	4.000000
Women	1	CVLT_7	Model 2a PICALM	32	2	16.000000
Women	1	CVLT_7	Model 2b PICALM x age	32	4	8.000000
Women	1	CVLT_7	Model 2c PICALM x age x education	32	7	4.571429
Women	1	CVLT_7	Model 3a APOE x PICALM	32	4	8.000000
Women	1	CVLT_7	Model 3b APOE x PICALM x age	32	6	5.333333
Women	1	CVLT_7	Model 3c APOE x PICALM x age x education	32	10	3.200000
Women	1	CVLT_8	Model 1a APOE	32	2	16.000000
Women	1	CVLT_8	Model 1b APOE x age	32	4	8.000000
Women	1	CVLT_8	Model 1c APOE x age x education	32	8	4.000000
Women	1	CVLT_8	Model 2a PICALM	32	2	16.000000
Women	1	CVLT_8	Model 2b PICALM x age	32	4	8.000000
Women	1	CVLT_8	Model 2c PICALM x age x education	32	7	4.571429
Women	1	CVLT_8	Model 3a APOE x PICALM	32	4	8.000000
Women	1	CVLT_8	Model 3b APOE x PICALM x age	32	6	5.333333
Women	1	CVLT_8	Model 3c APOE x PICALM x age x education	32	10	3.200000
Women	1	CVLT_9	Model 1a APOE	32	2	16.000000
Women	1	CVLT_9	Model 1b APOE x age	32	4	8.000000
Women	1	CVLT_9	Model 1c APOE x age x education	32	8	4.000000
Women	1	CVLT_9	Model 2a PICALM	32	2	16.000000
Women	1	CVLT_9	Model 2b PICALM x age	32	4	8.000000
Women	1	CVLT_9	Model 2c PICALM x age x education	32	7	4.571429

Women	1	CVLT_9	Model 3a APOE x PICALM	32	4	8.000000
Women	1	CVLT_9	Model 3b APOE x PICALM x age	32	6	5.333333
Women	1	CVLT_9	Model 3c APOE x PICALM x age x education	32	10	3.200000
Women	1	CVLT_10	Model 1a APOE	32	2	16.000000
Women	1	CVLT_10	Model 1b APOE x age	32	4	8.000000
Women	1	CVLT_10	Model 1c APOE x age x education	32	8	4.000000
Women	1	CVLT_10	Model 2a PICALM	32	2	16.000000
Women	1	CVLT_10	Model 2b PICALM x age	32	4	8.000000
Women	1	CVLT_10	Model 2c PICALM x age x education	32	7	4.571429
Women	1	CVLT_10	Model 3a APOE x PICALM	32	4	8.000000
Women	1	CVLT_10	Model 3b APOE x PICALM x age	32	6	5.333333
Women	1	CVLT_10	Model 3c APOE x PICALM x age x education	32	10	3.200000
Women	1	CVLT_11	Model 1a APOE	32	2	16.000000
Women	1	CVLT_11	Model 1b APOE x age	32	4	8.000000
Women	1	CVLT_11	Model 1c APOE x age x education	32	8	4.000000
Women	1	CVLT_11	Model 2a PICALM	32	2	16.000000
Women	1	CVLT_11	Model 2b PICALM x age	32	4	8.000000
Women	1	CVLT_11	Model 2c PICALM x age x education	32	7	4.571429
Women	1	CVLT_11	Model 3a APOE x PICALM	32	4	8.000000
Women	1	CVLT_11	Model 3b APOE x PICALM x age	32	6	5.333333
Women	1	CVLT_11	Model 3c APOE x PICALM x age x education	32	10	3.200000
Women	1	CVLT_12	Model 1a APOE	32	2	16.000000
Women	1	CVLT_12	Model 1b APOE x age	32	4	8.000000
Women	1	CVLT_12	Model 1c APOE x age x education	32	8	4.000000
Women	1	CVLT_12	Model 2a PICALM	32	2	16.000000
Women	1	CVLT_12	Model 2b PICALM x age	32	4	8.000000
Women	1	CVLT_12	Model 2c PICALM x age x education	32	7	4.571429
Women	1	CVLT_12	Model 3a APOE x PICALM	32	4	8.000000
Women	1	CVLT_12	Model 3b APOE x PICALM x age	32	6	5.333333

Women	1	CVLT_12	Model 3c APOE x PICALM x age x education	32	10	3.200000
Women	1	CVLT_13	Model 1a APOE	32	2	16.000000
Women	1	CVLT_13	Model 1b APOE x age	32	4	8.000000
Women	1	CVLT_13	Model 1c APOE x age x education	32	8	4.000000
Women	1	CVLT_13	Model 2a PICALM	32	2	16.000000
Women	1	CVLT_13	Model 2b PICALM x age	32	4	8.000000
Women	1	CVLT_13	Model 2c PICALM x age x education	32	7	4.571429
Women	1	CVLT_13	Model 3a APOE x PICALM	32	4	8.000000
Women	1	CVLT_13	Model 3b APOE x PICALM x age	32	6	5.333333
Women	1	CVLT_13	Model 3c APOE x PICALM x age x education	32	10	3.200000
Women	1	RPM	Model 1a APOE	32	2	16.000000
Women	1	RPM	Model 1b APOE x age	32	4	8.000000
Women	1	RPM	Model 1c APOE x age x education	32	8	4.000000
Women	1	RPM	Model 2a PICALM	32	2	16.000000
Women	1	RPM	Model 2b PICALM x age	32	4	8.000000
Women	1	RPM	Model 2c PICALM x age x education	32	7	4.571429
Women	1	RPM	Model 3a APOE x PICALM	32	4	8.000000
Women	1	RPM	Model 3b APOE x PICALM x age	32	6	5.333333
Women	1	RPM	Model 3c APOE x PICALM x age x education	32	10	3.200000
Men	0	CVLT_1	Model 1a APOE	59	2	29.500000
Men	0	CVLT_1	Model 1b APOE x age	59	4	14.750000
Men	0	CVLT_1	Model 1c APOE x age x education	59	8	7.375000
Men	0	CVLT_1	Model 2a PICALM	59	2	29.500000
Men	0	CVLT_1	Model 2b PICALM x age	59	4	14.750000
Men	0	CVLT_1	Model 2c PICALM x age x education	59	7	8.428571
Men	0	CVLT_1	Model 3a APOE x PICALM	59	4	14.750000
Men	0	CVLT_1	Model 3b APOE x PICALM x age	59	6	9.833333
Men	0	CVLT_1	Model 3c APOE x PICALM x age x education	59	10	5.900000
Men	0	CVLT_2	Model 1a APOE	59	2	29.500000
Men	0	CVLT_2	Model 1b APOE x age	59	4	14.750000

Men	0	CVLT_2	Model 1c APOE x age x education	59	8	7.375000
Men	0	CVLT_2	Model 2a PICALM	59	2	29.500000
Men	0	CVLT_2	Model 2b PICALM x age	59	4	14.750000
Men	0	CVLT_2	Model 2c PICALM x age x education	59	7	8.428571
Men	0	CVLT_2	Model 3a APOE x PICALM	59	4	14.750000
Men	0	CVLT_2	Model 3b APOE x PICALM x age	59	6	9.833333
Men	0	CVLT_2	Model 3c APOE x PICALM x age x education	59	10	5.900000
Men	0	CVLT_3	Model 1a APOE	59	2	29.500000
Men	0	CVLT_3	Model 1b APOE x age	59	4	14.750000
Men	0	CVLT_3	Model 1c APOE x age x education	59	8	7.375000
Men	0	CVLT_3	Model 2a PICALM	59	2	29.500000
Men	0	CVLT_3	Model 2b PICALM x age	59	4	14.750000
Men	0	CVLT_3	Model 2c PICALM x age x education	59	7	8.428571
Men	0	CVLT_3	Model 3a APOE x PICALM	59	4	14.750000
Men	0	CVLT_3	Model 3b APOE x PICALM x age	59	6	9.833333
Men	0	CVLT_3	Model 3c APOE x PICALM x age x education	59	10	5.900000
Men	0	CVLT_4	Model 1a APOE	59	2	29.500000
Men	0	CVLT_4	Model 1b APOE x age	59	4	14.750000
Men	0	CVLT_4	Model 1c APOE x age x education	59	8	7.375000
Men	0	CVLT_4	Model 2a PICALM	59	2	29.500000
Men	0	CVLT_4	Model 2b PICALM x age	59	4	14.750000
Men	0	CVLT_4	Model 2c PICALM x age x education	59	7	8.428571
Men	0	CVLT_4	Model 3a APOE x PICALM	59	4	14.750000
Men	0	CVLT_4	Model 3b APOE x PICALM x age	59	6	9.833333
Men	0	CVLT_4	Model 3c APOE x PICALM x age x education	59	10	5.900000
Men	0	CVLT_5	Model 1a APOE	59	2	29.500000
Men	0	CVLT_5	Model 1b APOE x age	59	4	14.750000
Men	0	CVLT_5	Model 1c APOE x age x education	59	8	7.375000
Men	0	CVLT_5	Model 2a PICALM	59	2	29.500000
Men	0	CVLT_5	Model 2b PICALM x age	59	4	14.750000
Men	0	CVLT_5	Model 2c PICALM x age x education	59	7	8.428571

Men	0	CVLT_5	Model 3a APOE x PICALM	59	4	14.750000
Men	0	CVLT_5	Model 3b APOE x PICALM x age	59	6	9.833333
Men	0	CVLT_5	Model 3c APOE x PICALM x age x education	59	10	5.900000
Men	0	CVLT_6	Model 1a APOE	59	2	29.500000
Men	0	CVLT_6	Model 1b APOE x age	59	4	14.750000
Men	0	CVLT_6	Model 1c APOE x age x education	59	8	7.375000
Men	0	CVLT_6	Model 2a PICALM	59	2	29.500000
Men	0	CVLT_6	Model 2b PICALM x age	59	4	14.750000
Men	0	CVLT_6	Model 2c PICALM x age x education	59	7	8.428571
Men	0	CVLT_6	Model 3a APOE x PICALM	59	4	14.750000
Men	0	CVLT_6	Model 3b APOE x PICALM x age	59	6	9.833333
Men	0	CVLT_6	Model 3c APOE x PICALM x age x education	59	10	5.900000
Men	0	CVLT_7	Model 1a APOE	59	2	29.500000
Men	0	CVLT_7	Model 1b APOE x age	59	4	14.750000
Men	0	CVLT_7	Model 1c APOE x age x education	59	8	7.375000
Men	0	CVLT_7	Model 2a PICALM	59	2	29.500000
Men	0	CVLT_7	Model 2b PICALM x age	59	4	14.750000
Men	0	CVLT_7	Model 2c PICALM x age x education	59	7	8.428571
Men	0	CVLT_7	Model 3a APOE x PICALM	59	4	14.750000
Men	0	CVLT_7	Model 3b APOE x PICALM x age	59	6	9.833333
Men	0	CVLT_7	Model 3c APOE x PICALM x age x education	59	10	5.900000
Men	0	CVLT_8	Model 1a APOE	59	2	29.500000
Men	0	CVLT_8	Model 1b APOE x age	59	4	14.750000
Men	0	CVLT_8	Model 1c APOE x age x education	59	8	7.375000
Men	0	CVLT_8	Model 2a PICALM	59	2	29.500000
Men	0	CVLT_8	Model 2b PICALM x age	59	4	14.750000
Men	0	CVLT_8	Model 2c PICALM x age x education	59	7	8.428571
Men	0	CVLT_8	Model 3a APOE x PICALM	59	4	14.750000
Men	0	CVLT_8	Model 3b APOE x PICALM x age	59	6	9.833333

Men	0	CVLT_8	Model 3c APOE x PICALM x age x education	59	10	5.900000
Men	0	CVLT_9	Model 1a APOE	59	2	29.500000
Men	0	CVLT_9	Model 1b APOE x age	59	4	14.750000
Men	0	CVLT_9	Model 1c APOE x age x education	59	8	7.375000
Men	0	CVLT_9	Model 2a PICALM	59	2	29.500000
Men	0	CVLT_9	Model 2b PICALM x age	59	4	14.750000
Men	0	CVLT_9	Model 2c PICALM x age x education	59	7	8.428571
Men	0	CVLT_9	Model 3a APOE x PICALM	59	4	14.750000
Men	0	CVLT_9	Model 3b APOE x PICALM x age	59	6	9.833333
Men	0	CVLT_9	Model 3c APOE x PICALM x age x education	59	10	5.900000
Men	0	CVLT_10	Model 1a APOE	59	2	29.500000
Men	0	CVLT_10	Model 1b APOE x age	59	4	14.750000
Men	0	CVLT_10	Model 1c APOE x age x education	59	8	7.375000
Men	0	CVLT_10	Model 2a PICALM	59	2	29.500000
Men	0	CVLT_10	Model 2b PICALM x age	59	4	14.750000
Men	0	CVLT_10	Model 2c PICALM x age x education	59	7	8.428571
Men	0	CVLT_10	Model 3a APOE x PICALM	59	4	14.750000
Men	0	CVLT_10	Model 3b APOE x PICALM x age	59	6	9.833333
Men	0	CVLT_10	Model 3c APOE x PICALM x age x education	59	10	5.900000
Men	0	CVLT_11	Model 1a APOE	59	2	29.500000
Men	0	CVLT_11	Model 1b APOE x age	59	4	14.750000
Men	0	CVLT_11	Model 1c APOE x age x education	59	8	7.375000
Men	0	CVLT_11	Model 2a PICALM	59	2	29.500000
Men	0	CVLT_11	Model 2b PICALM x age	59	4	14.750000
Men	0	CVLT_11	Model 2c PICALM x age x education	59	7	8.428571
Men	0	CVLT_11	Model 3a APOE x PICALM	59	4	14.750000
Men	0	CVLT_11	Model 3b APOE x PICALM x age	59	6	9.833333
Men	0	CVLT_11	Model 3c APOE x PICALM x age x education	59	10	5.900000
Men	0	CVLT_12	Model 1a APOE	59	2	29.500000
Men	0	CVLT_12	Model 1b APOE x age	59	4	14.750000

Men	0	CVLT_12	Model 1c APOE x age x education	59	8	7.375000
Men	0	CVLT_12	Model 2a PICALM	59	2	29.500000
Men	0	CVLT_12	Model 2b PICALM x age	59	4	14.750000
Men	0	CVLT_12	Model 2c PICALM x age x education	59	7	8.428571
Men	0	CVLT_12	Model 3a APOE x PICALM	59	4	14.750000
Men	0	CVLT_12	Model 3b APOE x PICALM x age	59	6	9.833333
Men	0	CVLT_12	Model 3c APOE x PICALM x age x education	59	10	5.900000
Men	0	CVLT_13	Model 1a APOE	59	2	29.500000
Men	0	CVLT_13	Model 1b APOE x age	59	4	14.750000
Men	0	CVLT_13	Model 1c APOE x age x education	59	8	7.375000
Men	0	CVLT_13	Model 2a PICALM	59	2	29.500000
Men	0	CVLT_13	Model 2b PICALM x age	59	4	14.750000
Men	0	CVLT_13	Model 2c PICALM x age x education	59	7	8.428571
Men	0	CVLT_13	Model 3a APOE x PICALM	59	4	14.750000
Men	0	CVLT_13	Model 3b APOE x PICALM x age	59	6	9.833333
Men	0	CVLT_13	Model 3c APOE x PICALM x age x education	59	10	5.900000
Men	0	RPM	Model 1a APOE	59	2	29.500000
Men	0	RPM	Model 1b APOE x age	59	4	14.750000
Men	0	RPM	Model 1c APOE x age x education	59	8	7.375000
Men	0	RPM	Model 2a PICALM	59	2	29.500000
Men	0	RPM	Model 2b PICALM x age	59	4	14.750000
Men	0	RPM	Model 2c PICALM x age x education	59	7	8.428571
Men	0	RPM	Model 3a APOE x PICALM	59	4	14.750000
Men	0	RPM	Model 3b APOE x PICALM x age	59	6	9.833333
Men	0	RPM	Model 3c APOE x PICALM x age x education	59	10	5.900000
Men	1	CVLT_1	Model 1a APOE	17	2	8.500000
Men	1	CVLT_1	Model 1b APOE x age	17	4	4.250000
Men	1	CVLT_1	Model 1c APOE x age x education	17	8	2.125000
Men	1	CVLT_1	Model 2a PICALM	17	2	8.500000
Men	1	CVLT_1	Model 2b PICALM x age	17	4	4.250000
Men	1	CVLT_1	Model 2c PICALM x age x education	17	7	2.428571

Men	1	CVLT_1	Model 3a APOE x PICALM	17	4	4.250000
Men	1	CVLT_1	Model 3b APOE x PICALM x age	17	6	2.833333
Men	1	CVLT_1	Model 3c APOE x PICALM x age x education	17	10	1.700000
Men	1	CVLT_2	Model 1a APOE	17	2	8.500000
Men	1	CVLT_2	Model 1b APOE x age	17	4	4.250000
Men	1	CVLT_2	Model 1c APOE x age x education	17	8	2.125000
Men	1	CVLT_2	Model 2a PICALM	17	2	8.500000
Men	1	CVLT_2	Model 2b PICALM x age	17	4	4.250000
Men	1	CVLT_2	Model 2c PICALM x age x education	17	7	2.428571
Men	1	CVLT_2	Model 3a APOE x PICALM	17	4	4.250000
Men	1	CVLT_2	Model 3b APOE x PICALM x age	17	6	2.833333
Men	1	CVLT_2	Model 3c APOE x PICALM x age x education	17	10	1.700000
Men	1	CVLT_3	Model 1a APOE	17	2	8.500000
Men	1	CVLT_3	Model 1b APOE x age	17	4	4.250000
Men	1	CVLT_3	Model 1c APOE x age x education	17	8	2.125000
Men	1	CVLT_3	Model 2a PICALM	17	2	8.500000
Men	1	CVLT_3	Model 2b PICALM x age	17	4	4.250000
Men	1	CVLT_3	Model 2c PICALM x age x education	17	7	2.428571
Men	1	CVLT_3	Model 3a APOE x PICALM	17	4	4.250000
Men	1	CVLT_3	Model 3b APOE x PICALM x age	17	6	2.833333
Men	1	CVLT_3	Model 3c APOE x PICALM x age x education	17	10	1.700000
Men	1	CVLT_4	Model 1a APOE	17	2	8.500000
Men	1	CVLT_4	Model 1b APOE x age	17	4	4.250000
Men	1	CVLT_4	Model 1c APOE x age x education	17	8	2.125000
Men	1	CVLT_4	Model 2a PICALM	17	2	8.500000
Men	1	CVLT_4	Model 2b PICALM x age	17	4	4.250000
Men	1	CVLT_4	Model 2c PICALM x age x education	17	7	2.428571
Men	1	CVLT_4	Model 3a APOE x PICALM	17	4	4.250000
Men	1	CVLT_4	Model 3b APOE x PICALM x age	17	6	2.833333

Men	1	CVLT_4	Model 3c APOE x PICALM x age x education	17	10	1.700000
Men	1	CVLT_5	Model 1a APOE	17	2	8.500000
Men	1	CVLT_5	Model 1b APOE x age	17	4	4.250000
Men	1	CVLT_5	Model 1c APOE x age x education	17	8	2.125000
Men	1	CVLT_5	Model 2a PICALM	17	2	8.500000
Men	1	CVLT_5	Model 2b PICALM x age	17	4	4.250000
Men	1	CVLT_5	Model 2c PICALM x age x education	17	7	2.428571
Men	1	CVLT_5	Model 3a APOE x PICALM	17	4	4.250000
Men	1	CVLT_5	Model 3b APOE x PICALM x age	17	6	2.833333
Men	1	CVLT_5	Model 3c APOE x PICALM x age x education	17	10	1.700000
Men	1	CVLT_6	Model 1a APOE	17	2	8.500000
Men	1	CVLT_6	Model 1b APOE x age	17	4	4.250000
Men	1	CVLT_6	Model 1c APOE x age x education	17	8	2.125000
Men	1	CVLT_6	Model 2a PICALM	17	2	8.500000
Men	1	CVLT_6	Model 2b PICALM x age	17	4	4.250000
Men	1	CVLT_6	Model 2c PICALM x age x education	17	7	2.428571
Men	1	CVLT_6	Model 3a APOE x PICALM	17	4	4.250000
Men	1	CVLT_6	Model 3b APOE x PICALM x age	17	6	2.833333
Men	1	CVLT_6	Model 3c APOE x PICALM x age x education	17	10	1.700000
Men	1	CVLT_7	Model 1a APOE	17	2	8.500000
Men	1	CVLT_7	Model 1b APOE x age	17	4	4.250000
Men	1	CVLT_7	Model 1c APOE x age x education	17	8	2.125000
Men	1	CVLT_7	Model 2a PICALM	17	2	8.500000
Men	1	CVLT_7	Model 2b PICALM x age	17	4	4.250000
Men	1	CVLT_7	Model 2c PICALM x age x education	17	7	2.428571
Men	1	CVLT_7	Model 3a APOE x PICALM	17	4	4.250000
Men	1	CVLT_7	Model 3b APOE x PICALM x age	17	6	2.833333
Men	1	CVLT_7	Model 3c APOE x PICALM x age x education	17	10	1.700000
Men	1	CVLT_8	Model 1a APOE	17	2	8.500000
Men	1	CVLT_8	Model 1b APOE x age	17	4	4.250000

Men	1	CVLT_8	Model 1c APOE x age x education	17	8	2.125000
Men	1	CVLT_8	Model 2a PICALM	17	2	8.500000
Men	1	CVLT_8	Model 2b PICALM x age	17	4	4.250000
Men	1	CVLT_8	Model 2c PICALM x age x education	17	7	2.428571
Men	1	CVLT_8	Model 3a APOE x PICALM	17	4	4.250000
Men	1	CVLT_8	Model 3b APOE x PICALM x age	17	6	2.833333
Men	1	CVLT_8	Model 3c APOE x PICALM x age x education	17	10	1.700000
Men	1	CVLT_9	Model 1a APOE	17	2	8.500000
Men	1	CVLT_9	Model 1b APOE x age	17	4	4.250000
Men	1	CVLT_9	Model 1c APOE x age x education	17	8	2.125000
Men	1	CVLT_9	Model 2a PICALM	17	2	8.500000
Men	1	CVLT_9	Model 2b PICALM x age	17	4	4.250000
Men	1	CVLT_9	Model 2c PICALM x age x education	17	7	2.428571
Men	1	CVLT_9	Model 3a APOE x PICALM	17	4	4.250000
Men	1	CVLT_9	Model 3b APOE x PICALM x age	17	6	2.833333
Men	1	CVLT_9	Model 3c APOE x PICALM x age x education	17	10	1.700000
Men	1	CVLT_10	Model 1a APOE	17	2	8.500000
Men	1	CVLT_10	Model 1b APOE x age	17	4	4.250000
Men	1	CVLT_10	Model 1c APOE x age x education	17	8	2.125000
Men	1	CVLT_10	Model 2a PICALM	17	2	8.500000
Men	1	CVLT_10	Model 2b PICALM x age	17	4	4.250000
Men	1	CVLT_10	Model 2c PICALM x age x education	17	7	2.428571
Men	1	CVLT_10	Model 3a APOE x PICALM	17	4	4.250000
Men	1	CVLT_10	Model 3b APOE x PICALM x age	17	6	2.833333
Men	1	CVLT_10	Model 3c APOE x PICALM x age x education	17	10	1.700000
Men	1	CVLT_11	Model 1a APOE	17	2	8.500000
Men	1	CVLT_11	Model 1b APOE x age	17	4	4.250000
Men	1	CVLT_11	Model 1c APOE x age x education	17	8	2.125000
Men	1	CVLT_11	Model 2a PICALM	17	2	8.500000
Men	1	CVLT_11	Model 2b PICALM x age	17	4	4.250000
Men	1	CVLT_11	Model 2c PICALM x age x education	17	7	2.428571

Men	1	CVLT_11	Model 3a APOE x PICALM	17	4	4.250000
Men	1	CVLT_11	Model 3b APOE x PICALM x age	17	6	2.833333
Men	1	CVLT_11	Model 3c APOE x PICALM x age x education	17	10	1.700000
Men	1	CVLT_12	Model 1a APOE	17	2	8.500000
Men	1	CVLT_12	Model 1b APOE x age	17	4	4.250000
Men	1	CVLT_12	Model 1c APOE x age x education	17	8	2.125000
Men	1	CVLT_12	Model 2a PICALM	17	2	8.500000
Men	1	CVLT_12	Model 2b PICALM x age	17	4	4.250000
Men	1	CVLT_12	Model 2c PICALM x age x education	17	7	2.428571
Men	1	CVLT_12	Model 3a APOE x PICALM	17	4	4.250000
Men	1	CVLT_12	Model 3b APOE x PICALM x age	17	6	2.833333
Men	1	CVLT_12	Model 3c APOE x PICALM x age x education	17	10	1.700000
Men	1	CVLT_13	Model 1a APOE	17	2	8.500000
Men	1	CVLT_13	Model 1b APOE x age	17	4	4.250000
Men	1	CVLT_13	Model 1c APOE x age x education	17	8	2.125000
Men	1	CVLT_13	Model 2a PICALM	17	2	8.500000
Men	1	CVLT_13	Model 2b PICALM x age	17	4	4.250000
Men	1	CVLT_13	Model 2c PICALM x age x education	17	7	2.428571
Men	1	CVLT_13	Model 3a APOE x PICALM	17	4	4.250000
Men	1	CVLT_13	Model 3b APOE x PICALM x age	17	6	2.833333
Men	1	CVLT_13	Model 3c APOE x PICALM x age x education	17	10	1.700000
Men	1	RPM	Model 1a APOE	17	2	8.500000
Men	1	RPM	Model 1b APOE x age	17	4	4.250000
Men	1	RPM	Model 1c APOE x age x education	17	8	2.125000
Men	1	RPM	Model 2a PICALM	17	2	8.500000
Men	1	RPM	Model 2b PICALM x age	17	4	4.250000
Men	1	RPM	Model 2c PICALM x age x education	17	7	2.428571
Men	1	RPM	Model 3a APOE x PICALM	17	4	4.250000
Men	1	RPM	Model 3b APOE x PICALM x age	17	6	2.833333

Men	1	RPM	Model 3c APOE x PICALM x age x education	17	10	1.700000

Results of robust regression analyses for CVLT Recognition Total Hits performance

To evaluate whether the observed associations with CVLT Recognition Total Hits (CVLT_12) performance could be influenced by a potential ceiling effect or by influential observations, we repeated the regression analyses using robust linear models (RLM) and compared the estimates with the original ordinary least squares (OLS) models.

The robust regression analyses yielded results largely consistent with the original OLS findings. In Cluster 0, the APOE × age model showed a similar negative association between the APOE × age interaction term and recognition memory performance in both approaches (OLS: $\beta = -0.229$, $p = 0.001$; RLM: $\beta = -0.162$, $p = 0.003$), indicating that the observed association was not driven solely by extreme observations.

In Cluster 1, the effects observed in the OLS models were generally maintained after applying robust regression. Positive associations with education, as well as the age × education interaction, remained present across APOE-only, PICALM-only, and combined APOE × PICALM models. For example, in the APOE × age × education model, education remained positively associated with CVLT_12 performance (OLS: $\beta = 0.332$, $p = 0.021$; RLM: $\beta = 0.327$), and the age × education interaction showed a similar pattern (OLS: $\beta = 0.163$, $p < 0.001$; RLM: $\beta = 0.136$). Comparable patterns were observed for the PICALM × age × education and combined APOE × PICALM × age × education models.

Overall, robust regression analyses indicated that the observed CVLT_12 associations were not solely attributable to potential ceiling effects or influential observations. However, given the relatively small sample size of Cluster 1 (N = 32), these findings should be interpreted cautiously (see Table S6).

Table S6. Comparison of ordinary least squares (OLS) and robust linear regression (RLM) estimates for models predicting CVLT Recognition Total Hits (CVLT_12) performance

Cluster	Model	Predictor	OLS_beta	OLS_p	RLM_beta	RLM_SE	RLM_z	RLM_p
0	Model 1b APOE x age	const	15.581207	1.267516e-78	1.565796e+01	8.493745e-02	1.843469e+02	0.000000

0	Model 1b APOE x age	APOE	-0.396307	8.885809e-02	-3.702739e-01	1.848829e-01	-2.002748e+00	0.045204
0	Model 1b APOE x age	age_c	0.002740	9.288392e-01	4.227975e-04	2.464886e-02	1.715283e-02	0.986315
0	Model 1b APOE x age	APOE_age	-0.229210	1.389868e-03	-1.619092e-01	5.515973e-02	-2.935279e+00	0.003332
1	Model 1c APOE x age x education	const	15.749202	1.083618e-41	1.591443e+01	3.267518e-09	4.870496e+09	0.000000
1	Model 1c APOE x age x education	APOE	-0.017588	9.087808e-01	-2.514016e-10	5.631316e-09	-4.464349e-02	0.964391
1	Model 1c APOE x age x education	age_c	-0.020643	5.229572e-01	-3.550113e-02	1.180920e-09	-3.006227e+07	0.000000
1	Model 1c APOE x age x education	education_c	0.332172	2.056593e-02	3.270563e-01	4.989324e-09	6.555124e+07	0.000000
1	Model 1c APOE x age x education	APOE_age	-0.065656	2.647957e-01	-2.322039e-09	2.134000e-09	-1.088116e+00	0.276544
1	Model 1c APOE x age x education	APOE_education	-0.004602	9.087808e-01	-6.577333e-11	1.473309e-09	-4.464326e-02	0.964392
1	Model 1c APOE x age x education	age_education	0.162830	7.200152e-04	1.356932e-01	1.573311e-09	8.624691e+07	0.000000
1	Model 1c APOE x age x education	APOE_age_education	-0.017178	2.647957e-01	-6.075100e-10	5.583139e-10	-1.088116e+00	0.276544
1	Model 2c PICALM x age x education	const	15.649556	6.610240e-42	1.591511e+01	2.545311e-07	6.252719e+07	0.000000
1	Model 2c PICALM x age x education	PICALM	0.262126	8.976330e-02	6.450706e-07	4.402222e-07	1.465330e+00	0.142831
1	Model 2c PICALM	age_c	-0.030558	3.481222e-01	-3.563790e-02	9.465513e-08	-3.765026e+05	0.000000

	x age x education							
1	Model 2c PICALM x age x education	education_c	0.268993	5.041557e-02	3.244628e-01	3.880782e-07	8.360759e+05	0.000000
1	Model 2c PICALM x age x education	PICALM_age	-0.011003	8.529044e-01	-6.306046e-08	1.739066e-07	-3.626111e-01	0.716895
1	Model 2c PICALM x age x education	PICALM_education	0.068579	8.976330e-02	1.687685e-07	1.151744e-07	1.465330e+00	0.142831
1	Model 2c PICALM x age x education	age_education	0.158856	7.561642e-04	1.362162e-01	1.232461e-07	1.105237e+06	0.000000
1	Model 3c APOE x PICALM x age x education	const	15.684088	1.382644e-36	1.591529e+01	3.315350e-08	4.800485e+08	0.000000
1	Model 3c APOE x PICALM x age x education	APOE	-0.096928	6.504212e-01	-6.081313e-01	7.165546e-08	-8.486881e+06	0.000000
1	Model 3c APOE x PICALM x age x education	PICALM	0.283541	1.977210e-01	7.173551e-08	7.256217e-08	9.886075e-01	0.322855
1	Model 3c APOE x PICALM x age x education	age_c	-0.010419	7.436039e-01	-3.567343e-02	1.068388e-08	-3.338994e+06	0.000000
1	Model 3c APOE x PICALM x age x education	education_c	0.286607	4.247357e-02	3.237902e-01	4.529123e-08	7.149071e+06	0.000000
1	Model 3c APOE	APOE_PICALM	0.050835	8.709450e-01	5.691719e-01	1.050387e-07	5.418687e+06	0.000000

	x PICALM x age x education							
1	Model 3c APOE x PICALM x age x education	APOE_PICALM_age	0.111111	2.753322e-01	1.999973e-01	3.375198e-08	5.925500e+06	0.000000
1	Model 3c APOE x PICALM x age x education	APOE_PICALM_education	0.013300	8.709450e-01	1.489113e-01	2.748106e-08	5.418687e+06	0.000000
1	Model 3c APOE x PICALM x age x education	age_education	0.171105	3.778889e-04	1.363518e-01	1.396198e-08	9.765936e+06	0.000000
1	Model 3c APOE x PICALM x age x education	APOE_age_education	-0.555970	1.032754e-01	-7.644343e-01	1.112397e-07	-6.871954e+06	0.000000

===================================
CVLT_12 DISTRIBUTION
===================================

count 172.000000
mean 15.424419
std 0.979370
min 10.000000
25% 15.000000
50% 16.000000
75% 16.000000
max 16.000000
Name: CVLT_12, dtype: float64

Maximum possible observed score = 16.0
Participants with maximum score = 109
Percentage = 63.4%

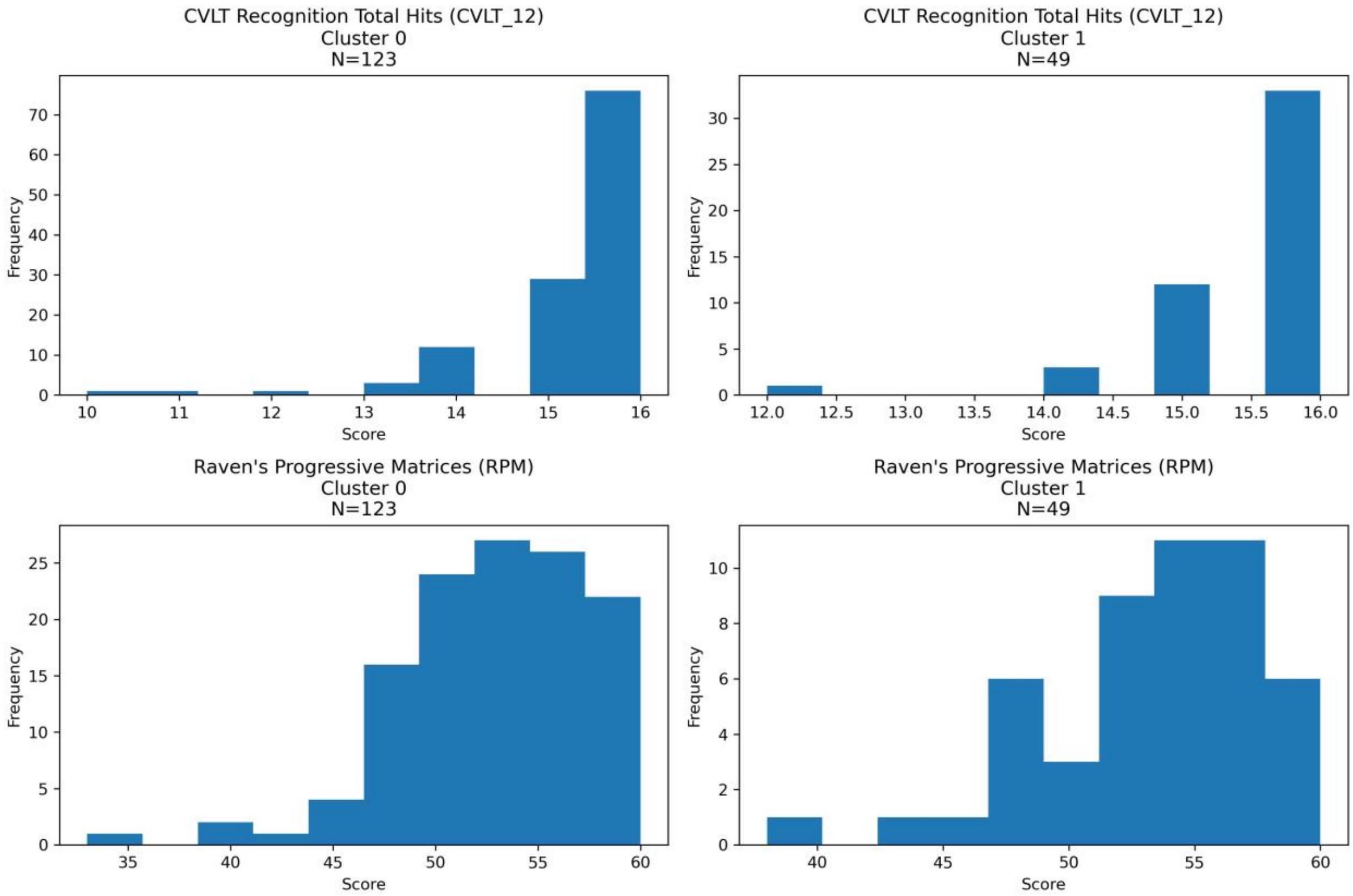

Figure S4. Distribution of CVLT Recognition Total Hits (CVLT_12) scores across personality-defined clusters. Distribution of Raven's Progressive Matrices (RPM) scores across personality-defined clusters.

Supplementary Analysis: Continuous Metatrait Regression Models

To complement the cluster-based analyses, additional ordinary least squares (OLS) regression models were performed to examine whether Stability and Plasticity, treated as continuous personality dimensions, showed independent linear associations with cognitive performance. The Big Five personality dimensions were standardized using z-scores, and metatraits were calculated according to the higher-order personality model proposed by DeYoung and colleagues. Stability was calculated as the mean of standardized Neuroticism (reversed), Agreeableness, and Conscientiousness scores, whereas Plasticity was calculated as the mean of standardized Extraversion and Openness scores.

The analyses included all available neuropsychological outcomes derived from the California Verbal Learning Test (CVLT-1 to CVLT-13) and Raven's Progressive Matrices (RPM). APOE ε4 carrier status was coded as a binary variable (1 = ε3/ε4 or ε4/ε4 carriers; 0 = non-carriers), whereas PICALM rs3851179 GG genotype status was coded as 1 for homozygous GG carriers and 0 for all remaining genotypes.

For each neuropsychological outcome, three complementary regression models were estimated using complete-case analysis.

The first model evaluated the effects of APOE status while controlling for continuous metatraits, demographic variables, sex, and relevant interaction terms. The model included APOE status, Stability, Plasticity, age, education, sex, APOE × Stability, APOE × Plasticity, APOE × age, APOE × education, and age × education interaction terms.

The second model was analogous but focused on PICALM genotype status. This model included PICALM status, Stability, Plasticity, age, education, sex, PICALM × Stability, PICALM × Plasticity, PICALM × age, PICALM × education, and age × education interaction terms.

The third model examined the combined effects of APOE and PICALM. This model included both genetic variables, Stability, Plasticity, age, education, sex, APOE × PICALM interaction, APOE × Stability, PICALM × Stability, APOE × Plasticity, PICALM × Plasticity, APOE × age, PICALM × age, APOE × education, PICALM × education, and age × education interaction terms.

Continuous predictors (age and education) were mean-centered prior to calculating interaction terms. All regression models were estimated using ordinary least squares

regression. For each predictor, regression coefficients (β), standard errors (SE), t statistics, p values, and 95% confidence intervals were extracted. In addition, model-level statistics, including explained variance (R^2), adjusted R^2, F statistics, and overall model significance, were reported.

Because the aim of this supplementary analysis was to identify specific predictors associated with cognitive performance rather than to evaluate only overall model significance, correction for multiple comparisons was applied at the coefficient level. P values for individual regression coefficients were adjusted using the Benjamini–Hochberg false discovery rate (FDR) procedure. Predictors with adjusted p values (pFDR) below 0.05 were considered statistically significant.

All analyses were conducted using Python, with ordinary least squares regression implemented using the statsmodels package.

Table S5.Complementary regression analyses testing linear associations between Stability and Plasticity metatraits and neuropsychological outcomes after false discovery rate correction (n=173)

Outcome	Model	Predictor	Beta	SE	t	p	CI_low	CI_high	R2	Adj_R2	F	Model_p	pFDR_coef
CVLT_9	PICALM	PICALM: Plasticity	-2.972557	0.913272	-3.254841	0.001383	-4.776095	-1.169019	0.157037	0.099443	2.726623	0.002957	0.012706
CVLT_9	APOE_PICALM	PICALM: Plasticity	-2.929177	0.909866	-3.219351	0.001565	-4.726514	-1.131840	0.211995	0.125568	2.452895	0.001991	0.014159

Table S6. FDR-corrected regression analyses examining interactions between personality metatraits and genetic variants (APOE and PICALM) in predicting cognitive performance (n=173).

Outcome	Model	Predictor	Beta	SE	t	p	CI_low	CI_high	R2	Adj_R2	F	Model_p	pFDR_coef
CVLT_5	PICALM	sex	2.061294	0.388775	5.302019	3.726976e-07	1.293538	2.829051	0.183860	0.128099	3.297278	0.000420	0.000006
CVLT_5	APOE	sex	2.010131	0.382886	5.249944	4.747696e-07	1.254004	2.766258	0.200857	0.146257	3.678717	0.000112	0.000007
CVLT_5	APOE_PICALM	sex	2.005615	0.394646	5.082056	1.062054e-06	1.226035	2.785194	0.212381	0.125997	2.458565	0.001941	0.000015
CVLT_1	APOE	sex	6.445966	1.310971	4.916940	2.150679e-06	3.857049	9.034882	0.169284	0.112527	2.982605	0.001240	0.000030
CVLT_1	PICALM	sex	6.360546	1.316566	4.831164	3.139940e-06	3.760581	8.960512	0.170084	0.113382	2.999596	0.001170	0.000043
CVLT_7	PICALM	sex	1.689508	0.356264	4.742291	4.625246e-06	0.985955	2.393061	0.177719	0.121539	3.163354	0.000667	0.000060
CVLT_7	APOE	sex	1.653315	0.350234	4.720597	5.080096e-06	0.961670	2.344961	0.197747	0.142935	3.607719	0.000143	0.000065

CVLT_1	APOE_PICALM	sex	6.151519	1.322519	4.651366	7.019120e-06	3.539033	8.764006	0.215694	0.129674	2.507471	0.001559	0.000088
CVLT_7	APOE_PICALM	sex	1.598944	0.356577	4.484141	1.417180e-05	0.894565	2.303322	0.228534	0.143922	2.700956	0.000648	0.000170
CVLT_2	APOE	sex	1.290856	0.297277	4.342272	2.485351e-05	0.703792	1.877921	0.150956	0.092947	2.602271	0.004487	0.000287
CVLT_2	PICALM	sex	1.265971	0.296789	4.265563	3.390393e-05	0.679870	1.852072	0.161726	0.104453	2.823755	0.002129	0.000376
CVLT_2	APOE_PICALM	sex	1.225760	0.301175	4.069923	7.472262e-05	0.630822	1.820697	0.191534	0.102863	2.160063	0.007218	0.000814
CVLT_3	APOE	sex	1.203513	0.301293	3.994498	9.839856e-05	0.608518	1.798508	0.136172	0.077153	2.307252	0.011861	0.001052
CVLT_3	PICALM	sex	1.206708	0.303610	3.974533	1.062195e-04	0.607136	1.806279	0.131112	0.071747	2.208565	0.016305	0.001115
CVLT_8	PICALM	sex	1.122153	0.299843	3.742465	2.531522e-04	0.530020	1.714285	0.119970	0.059844	1.995297	0.031941	0.002611
CVLT_3	APOE_PICALM	sex	1.137756	0.306622	3.710607	2.876949e-04	0.532057	1.743454	0.170010	0.078979	1.867601	0.024630	0.002917
CVLT_8	APOE	sex	1.090545	0.295324	3.692702	3.034557e-04	0.507336	1.673754	0.138162	0.079279	2.346374	0.010444	0.003024
CVLT_6	PICALM	sex	1.058084	0.287187	3.684301	3.128293e-04	0.490944	1.625223	0.116461	0.056095	1.929256	0.039142	0.003066
CVLT_6	APOE	sex	1.034024	0.282218	3.663917	3.367227e-04	0.476698	1.591351	0.138642	0.079792	2.355834	0.010127	0.003246
CVLT_8	APOE_PICALM	sex	1.075345	0.301671	3.564626	4.847374e-04	0.479428	1.671263	0.165724	0.074222	1.811163	0.030930	0.004597
CVLT_6	APOE_PICALM	sex	1.002077	0.288827	3.469476	6.754440e-04	0.431533	1.572622	0.163043	0.071248	1.776161	0.035560	0.006304
RPM	PICALM	age_c	-0.451742	0.140932	-3.205387	1.626726e-03	-0.730056	-0.173428	0.126009	0.066295	2.110213	0.022295	0.014493
CVLT_4	APOE	sex	0.886610	0.300658	2.948902	3.664192e-03	0.292869	1.480351	0.078535	0.015577	1.247430	0.260017	0.032157
CVLT_11	APOE	sex	-0.663858	0.235551	-2.818320	5.433290e-03	-1.129027	-0.198690	0.105418	0.044298	1.724759	0.072154	0.046982

Stability and Plasticity showed a significant positive correlation in the overall sample ($r = 0.381$, $p < 0.001$), indicating a moderate association between these two metatraits. The correlation was somewhat stronger in women ($r = 0.466$, $p < 0.001$) than in men ($r = 0.281$, $p = 0.008$), although the relationship remained significant in both groups. These findings suggest that Stability and Plasticity represent related but distinct personality dimensions rather than a single underlying construct.

Table Sx. Correlations between Stability and Plasticity metatraits

	Group	N	Pearson_r	p_value
**0**	Overall	191	0.380611	5.590259e-08
**1**	Women	103	0.466024	7.017884e-07
**2**	Men	88	0.281196	7.956426e-03

Note. Stability was calculated as (-Neuroticism + Agreeableness + Conscientiousness)/3, whereas Plasticity was calculated as (Extraversion + Openness)/2, according to the higher-order Big Five model proposed by DeYoung and colleagues (DeYoung et al., 2002; DeYoung et al., 2007).
